# Microarray analysis of *Foxa2 *mutant mouse embryos reveals novel gene expression and inductive roles for the gastrula organizer and its derivatives

**DOI:** 10.1186/1471-2164-9-511

**Published:** 2008-10-30

**Authors:** Owen J Tamplin, Doris Kinzel, Brian J Cox, Christine E Bell, Janet Rossant, Heiko Lickert

**Affiliations:** 1 Program in Developmental and Stem Cell Biology, Research Institute, The Hospital for Sick Children, 555 University Avenue, Toronto, Ontario, M5G 1X8, Canada; 2 Department of Molecular Genetics, Medical Sciences Building, 1 King's College Circle, University of Toronto, Toronto, Ontario, M5S 1A8, Canada; 3 Institute of Stem Cell Research, Helmholtz Zentrum München, German Research Center for Environmental Health (GmbH), Ingolstädter Landstr 1, 85764 Neuherberg, Germany; 4 Samuel Lunenfeld Research Institute, Mount Sinai Hospital, 600 University Avenue, Toronto, Ontario, M5G 1X5, Canada; 5 Department of Obstetrics and Gynecology, 92 College Street, University of Toronto, Toronto, Ontario, M5G 1L4, Canada

## Abstract

**Background:**

The Spemann/Mangold organizer is a transient tissue critical for patterning the gastrula stage vertebrate embryo and formation of the three germ layers. Despite its important role during development, there are still relatively few genes with specific expression in the organizer and its derivatives. Foxa2 is a forkhead transcription factor that is absolutely required for formation of the mammalian equivalent of the organizer, the node, the axial mesoderm and the definitive endoderm (DE). However, the targets of Foxa2 during embryogenesis, and the molecular impact of organizer loss on the gastrula embryo, have not been well defined.

**Results:**

To identify genes specific to the Spemann/Mangold organizer, we performed a microarray-based screen that compared wild-type and *Foxa2 *mutant embryos at late gastrulation stage (E7.5). We could detect genes that were consistently down-regulated in replicate pools of mutant embryos versus wild-type, and these included a number of known node and DE markers. We selected 314 genes without previously published data at E7.5 and screened for expression by whole mount *in situ *hybridization. We identified 10 novel expression patterns in the node and 5 in the definitive endoderm. We also found significant reduction of markers expressed in secondary tissues that require interaction with the organizer and its derivatives, such as cardiac mesoderm, vasculature, primitive streak, and anterior neuroectoderm.

**Conclusion:**

The genes identified in this screen represent novel Spemann/Mangold organizer genes as well as potential Foxa2 targets. Further investigation will be needed to define these genes as novel developmental regulatory factors involved in organizer formation and function. We have placed these genes in a Foxa2-dependent genetic regulatory network and we hypothesize how Foxa2 may regulate a molecular program of Spemann/Mangold organizer development. We have also shown how early loss of the organizer and its inductive properties in an otherwise normal embryo, impacts on the molecular profile of surrounding tissues.

## Background

The organizer is a highly specialized and transient structure that has been found in all studied vertebrates. It was originally discovered in amphibians by Spemann and Mangold in 1924 by its ability to induce much of a secondary body axis when transplanted to a host embryo [1]. These experiments were repeated with equivalent tissues in the chick (Hensen's node; [2]), zebrafish (shield; [3]), and mouse (node; [4]), demonstrating that organizer function is highly conserved and is essential for patterning of the basic body plan during gastrulation. In mouse, the early gastrula organizer (EGO) forms just anterior to the emerging primitive streak (PS) at E6.5, then gives rise to the mid-gastrula organizer (MGO) as the PS elongates towards the distal tip of the mouse embryo, and finally forms the morphologically visible node at E7.5 [5]. These organizer cell populations contribute to the axial mesoderm and notochord, and are the source of DE that displaces the embryonic visceral endoderm (VE) into the extra-embryonic region (reviewed [6]). Although the organizer has been studied for more than 80 years, there are still relatively few genes that have defined expression in this highly specialized tissue.

One important and conserved organizer-specific gene is *Foxa2*, which is first expressed in the mouse embryo at E6.5 in the EGO and the anterior VE [7-9]. As gastrulation proceeds, *Foxa2 *is expressed in the later organizer populations (MGO and node) and organizer derivatives (anterior mesendoderm (AME), notochord, DE and floor plate). *Foxa2 *is absolutely required in these tissues, as they do not form in the *Foxa2 *null embryo [10,11]. *Foxa2 *null embryos die between E7.5 and E9.5 due to embryonic patterning defects of the primary body axes. *Foxa2 *has an additional function in the VE for proper elongation of the PS, a function that can be rescued in a *Foxa2 *mutant embryo by restoration of wild-type VE [12]. As an indication of its conserved importance among vertebrates, *Foxa2 *has organizer-specific homologues in zebrafish [13,14], *Xenopus *[15], and chicken [16]. Despite its conserved and important functions, the only defined targets of Foxa2 in the early embryo are *Shh *[17,18] and *Otx2 *[19], leaving many open questions regarding the downstream targets of Foxa2 in vertebrate development. Furthermore, the lack of markers for organizer derivatives, particularly the DE, is a major obstacle in our understanding of the molecular pathways that underlie differentiation of distinct lineages in the early embryo [20]. This is of particular significance when attempting to coax embryonic stem (ES) cells into specific cell types for therapeutic applications [21,22].

A number of groups have designed screens to identify regionally specific transcripts within the mouse gastrula embryo, as well as novel molecular markers of node, AME, and DE [23-28]. Transcriptional profiling of the embryo has been done using subtractive cDNA libraries, serial analysis of gene expression (SAGE), and microarrays. These techniques, in conjunction with micro-dissection, flow cytometry, and wild-type versus mutant comparison, have highlighted the molecular differences between tissues and cell types in the gastrula. Although these screens were highly productive, they by no means reached saturation and each study contributed its own set of novel markers depending on the design and limitations of each approach. This suggested to us that there were still many more genes to be identified in the tissues derived from the mouse gastrula organizer.

In this study, we present a functionally based microarray screen to identify novel molecular markers of the node, AME, and DE. We compared total RNA from pools of wild-type and *Foxa2 *mutant embryos at E7.5. We could detect genes whose levels were significantly reduced in *Foxa2 *mutant embryos due to the absence of organizer-derived tissues. The genes we detected represent putative targets of Foxa2 or indirect targets due to the absence of organizer tissue. Based on the microarray results, we conducted whole mount *in situ *hybridization screens to identify novel gene expression patterns. Finally, we placed our results in the context of a gene regulatory network of organizer development.

## Results

### Microarray Analysis

**Figure 1:**
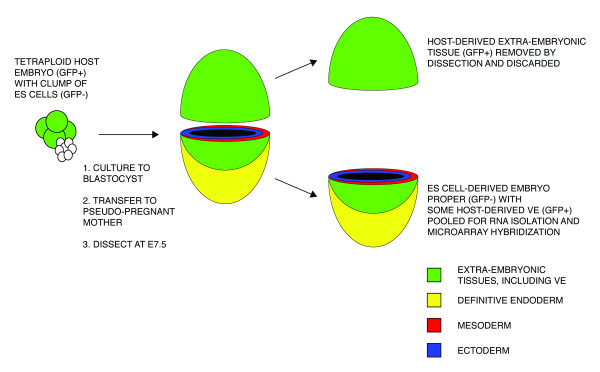
Overview of the method used to derive and collect *Foxa2 *mutant epiblasts for analysis.

To identify novel genes implicated in organizer and endoderm formation, we performed a differential gene expression comparison of wild-type and *Foxa2 *null embryos at E7.5. *Foxa2 *null embryos were derived from *Foxa2 *null ES cells [12] using the tetraploid complementation technique [29,30]. Briefly, this technique involves aggregating ES cells (GFP^-^) with wild-type tetraploid host embryos (GFP^+^), culturing the chimeric embryos to blastocyst stage, then transferring back to pseudo-pregnant recipient mothers (Figure 1). In these experiments, the ES cells contribute to the epiblast and the tetraploid host will contribute to the extra-embryonic tissues, including the VE. The embryos are then dissected at E7.5 and screened for GFP^- ^ES cell contribution to the epiblast. In our experiments, this technique has the advantage of rescuing the function of *Foxa2 *in the VE, allowing proper elongation of the PS, and analysis of Foxa2 function exclusively in the embryo proper [12]. The technique also allowed us to rapidly collect litters of entirely homozygous null embryos (~50 gastrula stage E7.25–E7.75 embryos per experiment). Furthermore, total RNA could be isolated without the need for amplification before hybridization to Affymetrix GeneChip microarrays. We initially used the U74Av2 array platform (~12,000 transcripts represented), and then to expand the scope of our study, we re-hybridized the same biological samples to the whole genome MOE430v2 array platform (~39,000 transcripts represented) when it became available. We performed the array experiments on two biological replicate pools each of *Foxa2 *null and wild-type epiblasts. We also collected a third biological replicate pool of *Foxa2 *mutant and wild-type embryos that we could use for independent quantitative validation of gene expression level changes (see below).

After normalization and filtering of the U74Av2 microarray data, we scored probe sets as consistently reduced in *Foxa2 *null embryos compared to wild-type, if they had a significant change in two out of four cross comparisons (p ≤ 0.01; see Methods for details). We hypothesized that these genes would represent direct or indirect targets of Foxa2, due to loss of activation by Foxa2 or loss of organizer-derived tissues, respectively. Our *Foxa2 *mutant microarrays also detected many up-regulated genes (data not shown), which could represent putative target genes repressed by Foxa2. There is evidence that Foxa2 acts as a repressor [31], however in our experiments these results are confounded and would be difficult to analyze. This is because a *Foxa2 *mutant epiblast derived by tetraploid complementation does not displace its wild-type VE, and when compared to a wild-type epiblast that *does *displace its VE, the mutant appears to be highly enriched for VE markers (data not shown); this makes it impossible to distinguish between genuine up-regulated genes, and genes up-regulated due to VE enrichment. Accordingly, we focused on the genes with reduced levels in *Foxa2 *mutant embryos, and could identify a number of known markers of the node, AME and DE (Table 1). A number of these known markers were also previously validated in the literature as being reduced or absent in *Foxa2 *null embryos (Table 1).

**Table 1:** Summary of node, notochord, and DE genes with reduced expression in *Foxa2 *mutants.

Expression Group	Gene Symbol	Microarray* (MGU74v2A)	Microarray* (MOE430v2)	Q-PCR*	Reduced expression in Foxa2 mutants by whole mount in situ hybridization
Known node and/or notochord markers	Foxd4	-2.80	-3.74	-4.64	Compare Figures 2A and 2D (n = 6/6) Ref: [34]
	Foxa1	-1.18	-1.56	-1.69	Ref: [10]
	T	-0.90	-0.78	-1.43	Ref: [10-12]
	Foxa2	-1.28	-0.87	--	--
	Shh	--	-1.43	--	Ref: [10-12,38]
	Car3	--	-1.55	--	--
	Cthrc1	--	-1.45	--	--
	Chrd	--	-1.49	--	--
	Tmprss2	--	-0.36	--	--
	Dynlrb2	--	-0.69	--	--

Novel node and/or notochord markers	Gal	-0.93	-1.58	-3.32	Compare Figures 2B and 2E (n = 7/7)
	Prnp	-0.65	-0.76	-0.30	Data not shown (n = 3/3)
	Pim1	-0.63	-1.68	--	--
	Smoc1	-0.20	-0.68	--	--
	Gstm5	-0.48	--	--	--
	Cyb561	-0.40	--	--	--
	1700027A23Rik	--	-1.01	--	--
	Josd2	--	-1.04	--	--
	Mlf1	--	-0.87	--	--
	1700009P17Rik	--	-1.26	--	--

Known DE markers	Sox17	-0.83	-0.87	-0.58	--
	Cer1	-1.40	-1.87	-2.32	Ref: [38]
	Foxa2	-1.28	-0.87	--	--
	Foxa1	-1.18	-1.56	-1.69	Ref: [10]
	Trh	-0.65	-0.79	--	--
	Shh	--	-1.43	--	Ref: [10-12,38]
	Gprc5c	--	-1.64	--	--
	Cyp26a1	--	-0.40	--	--
	Tmprss2	--	-0.36	--	--
	Cpn1	--	-0.79	--	--

Novel DE markers	Cldn4	-0.60	--	-0.40	Ref: (Burtscher & Lickert, 2008, manuscript submitted)
	Itga3	-0.58	--	-0.60	Compare Figures 2C and 2F (n = 4/4)
	Cpm	--	-2.45	--	--
	Efhd2	--	-0.64	--	--
	Ppp1r14a	--	-0.90	--	--

* values are log_2 _transformed ratios of *Foxa2 *mutant pools over wild type

Abbreviation: DE, definitive endoderm

**Figure 2:**
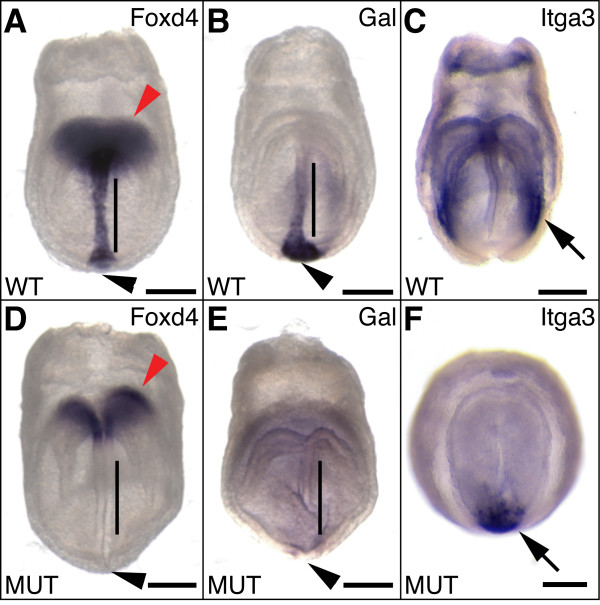
**Validation of genes reduced in the *Foxa2* mutant by whole mount *in situ* hybridization.** (A-C) Wild-type and (D-F) *Foxa2* mutant embryos at E7.75. (A, D) *Foxd4* expression is absent from node and AME, and reduced in the ANE in mutant embryos compared to wild-type. (B, E) *Gal* is completely absent from node, AME and PS in the mutant embryos compared to wild-type. (Note: the *Foxa2 *mutant embryo in (E) has some background signal due to over-staining to detect any residual *Gal* expression). (C, F) *Itga3* is severely reduced in the mutant embryo compared to wild-type and is only expressed in a small area around the APS. Black arrowheads indicate node; black lines indicate AME; red arrowheads indicate ANE; black arrows indicate DE. Scale bars are 200 μm.

We found *Foxa1 *transcript, another forkhead transcription factor that follows *Foxa2 *expression in the AME and DE [7-9], was reduced in the *Foxa2 *null embryo. This is consistent with previous data that showed *Foxa1 *is reduced in both *Foxa2 *null embryos [10] and embyroid bodies (EBs) derived from *Foxa2 *null ES cells [32]. We also found reduction of *Brachyury *(*T*) levels, which is expressed in the PS, node and AME at E7.5 [33]. This is consistent with loss of *Brachyury *expression specifically in the node and AME, but not PS, of tetraploid-derived *Foxa2 *null embryos at E7.5 [12]. One of the most highly down-regulated genes in the *Foxa2 *null data set was *Foxd4 *(formerly *fkh-2*). *Foxd4 *expression is completely lost in the axial midline of *Foxa2 *null embryos at E8.5 and is also slightly reduced in the anterior neuroectoderm (ANE) [34]. We confirmed that expression of *Foxd4 *in the node and AME at E7.5 was absent in *Foxa2 *null embryos (compare Figures 2A and 2D; n = 6/6). In addition to known markers of the node and AME, we also found DE markers *Sox17 *[35], *Trh *[25,36] and *Cer1 *[37] were reduced in *Foxa2 *null embryos, consistent with previous data that showed loss of *Foxa2 *in the epiblast leads to reduction of *Cer1 *[38]. Finally, we also detected reduction in genes strongly expressed in the DE as well as in other germ layers, such as *Arg1 *and *Cdx1 *[25].

To provide further validation of the microarray results, we performed quantitative real-time PCR (Q-PCR) on third biological replicate pools of wild-type and *Foxa2 *mutant embryos. We confirmed that the known node, AME and DE markers described above (*Foxa1*, *T*, *Foxd4*, *Sox17*, *Cer1*; Table 1 and Additional File 1) had reduced levels in the *Foxa2 *mutant embryo.

Reduction of these known markers provided evidence that our microarray-based screen could detect changes in transcripts specific to organizer-derived tissues at E7.5.

### Whole mount *in situ *hybridization screening: Phase I

Based on the presence of known node, AME or DE markers among the most highly down-regulated genes in the *Foxa2 *null embryo (Table 1), we selected further genes for expression analysis by whole mount *in situ *hybridization. In the first phase of our screen, we analyzed genes based on the criteria that they were significantly reduced in the *Foxa2 *null embryo (≥ 1.5 fold decrease, as detected by the U74Av2 array) and did not have previously published expression patterns as early as E7.5 (n = 106, after removing redundancies; Additional File 2). After completing the first round of screening, we found the frequency of novel, regionally specific patterns was 13% (n = 14/106), which was on par with previous gene expression screens at the same stage of embryonic mouse development (e.g. Sousa-Nunes and colleagues found 18%; n = 29/160 [23]). The frequency of regionally specific genes with a ≥ 1.5 fold decrease in the *Foxa2 *null mutant more than doubles if the known (n = 24) and novel (n = 14) expression patterns in the data set are considered together (29%; n = 38/131; Additional File 2).

**Figure 3:**
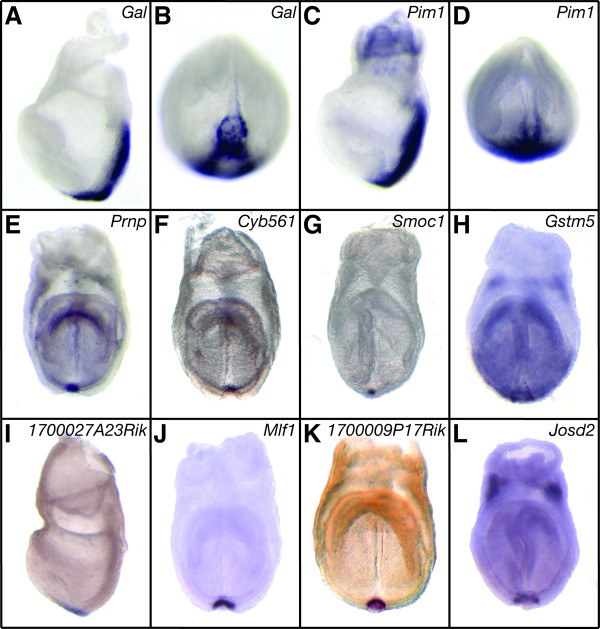
**Genes we identified that are expressed in the node at E7.5**. (A, B) *Gal *and (C, D) *Pim1 *are expressed in the PS and node. (E) *Prnp*, (F) *Cyb561*, (G) *Smoc1*, (I) *1700027A23Rik*, (J) *Mlf1*, (K) *1700009P17Rik*, and (L) *Josd2 *are all specifically expressed in the node (Note: later at E7.75, *Smoc1 *is expressed in the node and more broadly in the mesoderm; Additional File 3). (H) *Gstm5 *has widespread expression throughout the embryo, but is expressed strongly in the periphery of the node. (A, C, I) lateral view, anterior left; (B, D) distal view, anterior top; (E-H, J-L) anterior view.

In this initial phase of the screen we found three genes with novel expression domains in the node and/or AME: *Gal *(Figures 3A, 3B), *Pim1 *(Figures 3C, 3D), and *Prnp *(Figure 3E). *Gal *(*galanin*) encodes a peptide hormone that controls various biological activities [39], and is expressed in the node, AME, and PS at E7.5 (Figures 2B, 3A, and 3B). We confirmed that *Gal *was completely absent in *Foxa2 *mutant embryos (compare Figures 2B and 2E; n = 7/7). *Gal *has an intriguing expression pattern in the PS, which is first observed in the most posterior and medial region of the streak before it bifurcates, and is then expressed more laterally in the posterior mesoderm (Additional File 3). At E9.0 *Gal *is weakly expressed in the heart and posterior notochord (Additional File 3). *Pim1 *(*proviral integration site 1*) is a serine/threonine kinase [40], and is expressed in the node and PS at E7.5 (Figures 3C, 3D), after which its expression becomes widespread at E8.5 (Additional File 3). We found *Prnp*, the gene that encodes prion protein, is expressed specifically in the node at E7.75 (Figure 3E). This was much earlier in development than was previously reported, which described initial onset in the mouse embryo at E9.5 [41]. Third replicate pool validation of *Gal *and *Prnp *levels using Q-PCR confirmed their reduction in *Foxa2 *null embryos (Table 1 and Additional File 1). We also identified genes that, although widespread at E7.75, had an obvious and strong expression domain in the node (*Igfbp5 *and *Ppp1r1a*; Additional File 3).

**Figure 4:**
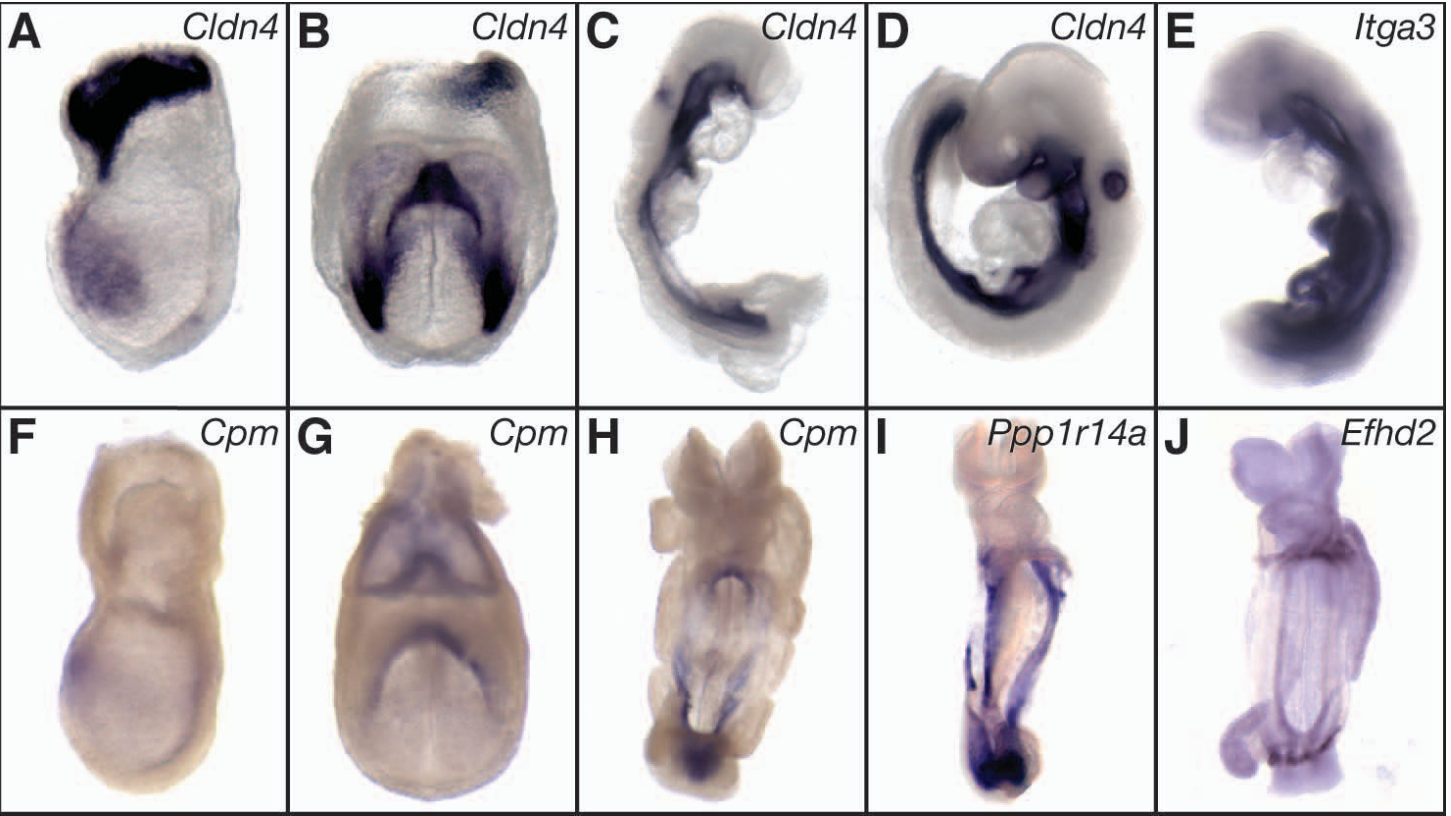
**Genes we identified that are expressed in the DE**. (A) *Cldn4 *is expressed in the anterior DE and in extra-embryonic regions at E7.25, (B) in the foregut pocket, lateral DE, and 37 extra-embryonic regions at E7.75, (C-D) and in the gut endoderm and otic vesicle from E8.5–9.0. (E) *Itga3 *is strongly expressed throughout the gut endoderm at E8.5. (F) *Cpm *is expressed in the anterior DE at E7.5, (G) strongly in the foregut pocket and throughout the DE at E7.75, (H) and in the ventral aspect of the gut at E8.5 (Note: at longer exposure, *Cpm *can be seen in more DE cells; Additional File 3). (I) *Ppp1r14a* is expressed in the ventral aspect of the gut at E8.5. (J) *Efhd2* is highly expressed in the rostral foregut and caudal hindgut at E8.5. (A, F) lateral view, anterior left; (B, G) anterior view; (C-E) lateral view; (H-J) ventral view.

The two novel DE expression patterns that were revealed in the first phase of the screen were *Cldn4 *and *Itga3*. *Cldn4 *(*claudin 4*) encodes a tight junction protein that is expressed in the anterior DE and extra-embryonic regions at E7.25, then in lateral DE and the foregut pocket at E7.75 (Figures 4A, 4B). *Cldn4 *is restricted to the gut endoderm and otic vesicle from E8.5–9.0 (Figures 4C, 4D). Reduction of *Cldn4 *has been observed in whole mount *Foxa2 *null embryos (Burtscher & Lickert, 2008, manuscript submitted). A large-scale embryonic gene expression screen previously identified *Cldn4 *and *Cldn6 *as regionally restricted genes, but only *Cldn6 *was described and shown at E9.5 as being expressed in the endoderm and otic vesicle [42]. *Cldn6 *was also one of two DE genes identified in a subtractive cDNA screen at E7.5 [23]. Furthermore, *Cldn9 *was identified in a SAGE-based screen to identify genes enriched in the DE [25]. This raises the possibility that at least part of the large *claudin *gene family may have important and therefore redundant functions in the endoderm. *Itga3 *(*integrin alpha 3*) is strongly expressed in a lateral region of the DE at E7.5 (Figure 2C). *Itga3 *is expressed throughout the notochord and gut endoderm at E8.5 (Figure 4E and data not shown). We confirmed *Itga3 *was reduced in *Foxa2 *null embryos (compare Figures 2C and 2F; n = 4/4). Interestingly, one of the four mutants examined still expressed some *Itga3 *in a small patch at the anterior primitive streak (APS; Figure 2F). This is consistent with previous results that showed endoderm-like cells form in tetraploid chimeric *Foxa2 *mutant embryos, which accumulate in the APS region due to failure of epithelialization (Burtscher & Lickert, 2008, manuscript submitted). Reduction of *Cldn4 *and *Itga3 *levels in *Foxa2 *mutant embryos was confirmed using Q-PCR (Table 1 and Additional File 1). We also found a number of genes that had weak to no staining at E7.5, but that showed endoderm expression at E8.5 (*Cd276*, *Galt*, *Gpx2*, *Pla2g7*, *Raet1e*, *Wfdc2*; Additional File 3); because the E7.5 microarray screen detected these genes as being significantly reduced, we took this to mean the *in situ *hybridization lacked sufficient sensitivity at this earlier stage.

Although the first phase of our screen identified a number of genes with expression specifically in the node at E7.5, we wondered if our ability to detect reduction in node-specific transcripts was at the limit of the microarray's sensitivity. We considered this because the node is a very small tissue at E7.5 (~100 cells [4]), and therefore must contribute only a tiny fraction of the total embryonic RNA at this stage. Given that node-specific genes could be present in the group of down-regulated genes with a less than 1.5 fold change in the *Foxa2 *mutant, we decided to examine a number of genes that fell below this threshold. We screened 46 genes that fell below the threshold, and found one additional gene expressed in the endoderm (*Igfbp3*; Additional Files 3 and 4), and three expressed in the node at E7.5; these were *Cyb561 *(*cytochrome b-561*; Figure 3F), *Smoc1 *(*SPARC related modular calcium binding 1*; Figure 3G), and *Gstm5 *(*glutathione S-transferase, mu 5*; Figure 3H). The frequency of regionally specific transcripts we found in this set was slightly lower than the first phase of the screen (9%; n = 4/46). Together these results suggested that our screening threshold was appropriately set for larger tissues in the gastrula embryo, such as the AME and DE, but that very small tissues, such as the node, were near the limit of detection in our experimental design.

### Whole mount *in situ *hybridization screening: Phase II

To expand on the screen performed using the U74Av2 arrays, we rehybridized the same biological samples to the upgraded whole genome MOE430v2 arrays. The newer arrays had much greater coverage of the mouse transcriptome, however the probe sets were also completely redesigned, so we could not directly compare overlapping data from the two platforms. We did find that the MOE430v2 arrays measured the same reduction in known node, AME and DE markers as the U74Av2 arrays (*Foxa2*, *Foxa1*, *Foxd4*, *T, Sox17*, *Cer1*, *Trh*; Table 1). The MOE430v2 arrays also demonstrated greater sensitivity in detecting reduction in known node, AME and DE markers represented on both old and new versions of the GeneChip (*Shh *[43], *Car3 *[44], *Chrd *[45], *Tmprss2 *[46], *Cpn1 *[25], *Cyp26a1 *[25,47]; Table 1). Furthermore, changes were detected in known node, AME and DE markers not represented on the older version of the array (*Cthrc1 *[48], *Dynlrb2 *[49], *Gprc5c *[24]; Table 1), demonstrating the increased potential to identify novel genes in our tissues of interest.

For the second phase of our screen, we analyzed genes detected by the MOE430v2 arrays as, 1) being significantly reduced in the *Foxa2 *mutant embryo (p ≤ 0.05 using a Wilcoxon paired rank test; Additional File 5), 2) not included in the first phase of our screen, and 3) having a potential functional role in embryogenesis based on the available annotation. We screened an additional 162 genes at E7.5 (and E8.5) and found 18 (11%; n = 18/162) had regionally specific expression patterns, consistent with the first phase of the screen (Additional File 6). Of these genes, four genes were expressed in the node: *1700027A23Rik *(Figure 3I); *Mlf1 *(*myeloid leukemia factor 1*; Figure 3J); *1700009P17Rik *(Figure 3K); and *Josd2 *(*Josephin domain containing 2*; Figure 3L). We also found three additional markers of the DE: *Cpm *(*carboxypeptidase M*), which is expressed in the anterior DE at E7.5 (Figure 4F), throughout the DE and strongly in the foregut pocket at E7.75 (Figure 4G), and then mostly in the ventral aspect of the presumptive gut at E8.5 (Figure 4H); *Ppp1r14a *(*protein phosphatase 1, regulatory (inhibitor) subunit 14A*), which is also expressed strongly in the ventral region of the gut at E8.5 (Figure 4I); and *Efhd2 *(*EF hand domain containing 2*), which is expressed strongly in the most rostral foregut and caudal hindgut at E8.5 (Figure 4J). By using an extended array platform to measure transcripts reduced in the *Foxa2 *mutant embryo, we increased the depth of our screen and found seven additional genes with novel expression domains in the node or DE.

In total, we screened 314 unique transcripts reduced in the *Foxa2 *mutant embryo. We found 10 novel expression domains in the node and 5 in the DE. These genes are expressed in regions of high Foxa2 activity and are therefore potential targets of Foxa2. Furthermore, novel node and endoderm genes are candidates for embryo patterning and notochord formation, and novel DE genes could have an additional role in organogenesis of the gut and its associated organs. Further studies will elucidate the functional roles of these genes, and whether or not they are directly downstream of Foxa2.

### Secondary phenotypes caused by the loss of organizer derivates

During our screen we found a large number of both known and novel regionally specific genes expressed outside the primary tissues affected in the *Foxa2 *mutant embryo. For example, based on the results of the U74Av2 array and first phase screen, we found genes expressed in the PS, ANE, cardiac mesoderm, and vasculature (Additional Files 2 and 3). The *Foxa2 *mutant chimeras we derived for this study using tetraploid complementation have wild-type rescued VE, which allows, an albeit smaller, PS to elongate [12]. However the APS, which is comprised of organizer tissue and gives rise to AME [5], does not form in these mutants. Given the important inductive role of the organizer in patterning the gastrula embryo (reviewed [50]), it is not surprising that we would detect molecular defects within the streak. In fact, 14% of the genes in the first phase of the screen were expressed in the PS (n = 18/131; Additional Files 2 and 3). Using Q-PCR, we confirmed that a number of these PS markers were in fact reduced in the *Foxa2 *mutant at E7.5 (*Wnt3a *[51,52], *Wnt8a *[53], *Hoxa1 *[54]; Additional File 1). This led us to conclude that our screen not only detected genes specific to the primary tissues absent in *Foxa2 *mutant embryos, but also found genes in secondary tissues affected by loss of interaction with organizer derivatives.

The ingressing mesoderm of the PS is not only an important signaling centre for the posterior of the embryo, but is also the site of mesodermal lineage specification. For example, endothelial precursors of the vasculature and cardiac mesoderm both originate within the PS [55]. We detected a number of early markers of the embryonic vasculature including *Tie1 *[56,57] and *Flt4 *[58]. We went on to confirm that *Flt4 *was expressed at E7.75 (Additional File 3), earlier than was previously reported at E8.5 [58], and is reduced in *Foxa2 *mutants by Q-PCR (Additional File 1). The cells that will make up the embryonic vasculature originate in the PS, and our data suggests that this early specification step is at least partially dependent on signals from organizer tissues.

The anterior endoderm and cardiac mesoderm develop in close proximity to each other, both at early stages of lineage specification and determination, as well as later during organogenesis (reviewed [59]). Extensive work in frog and chick embryos has explored the inductive interactions between these two tissues, although it is less clear how these mechanisms translate in the mouse embryo (reviewed [60]). There is some evidence that the anterior VE has a role in patterning cardiac precursors in the mouse [61]. In E8.5 *Foxa2 *mutant embryos derived by the same method used in this study (i.e. tetraploid complementation giving wild-type VE and a *Foxa2 *null epiblast), loss of the AME and DE does not severely affect patterning of the anterior heart field, based on the result that heart markers *Nppa *and *Wnt11 *are not greatly reduced [62]. However, von Both and colleagues also showed that the heart marker *Smpx *was partially reduced, raising the possibility that there is a subset of cardiac markers that are affected in these *Foxa2 *mutant chimeras. Consistent with this possibility, the first phase of our screen identified 6 markers of cardiac mesoderm as reduced in the *Foxa2 *mutant at E7.5 (*Actc1 *[63], *Frzb *[64], *Myl7 *[65], *Tagln *[66], *Tnnt2 *[67], *Myl1 *[68]; Additional Files 2 and 3). We went on to validate reduction of *Tnnt2 *in the *Foxa2 *mutant pool using Q-PCR (Additional File 1). This subset of cardiac markers suggests there are some genes expressed in the anterior heart field that are more dependent on interaction with DE versus VE, although further analysis will be required to understand these differential inductive roles.

The ANE, or presumptive forebrain, is highly dependent on inductive interactions with the anterior mesendoderm, definitive and visceral endoderm during gastrulation (reviewed [6]). Foxa2 has an important role in regulating downstream genes in the anterior VE, and therefore maintenance of the underlying ANE [12,19]. However, anterior organizer derivatives in the embryo proper also play a critical role in forebrain patterning events. When the AME is genetically compromised, either by loss of BMP antagonists *chordin *and *noggin *[45] or by attenuation of Nodal signal [69], severe defects in forebrain patterning result. A chimeric embryo without *Hhex *(formerly *Hex*) in the embryo proper results in loss of DE, and subsequently drastic forebrain truncation [70], further highlighting the importance of organizer derivatives in anterior patterning. Conditional inactivation of *Foxa2 *strictly in the epiblast, which leads to a phenotype similar to the *Foxa2 *tetraploid chimeras used in this study, also demonstrated that loss of organizer derivatives leads to reduction of ANE markers *Six3*, *Hesx1*, *Foxg1 *(formerly *BF1*), and *Fgf8 *[38]. We detected reduction of ANE markers *Six3 *and *Hesx1 *by microarray in the *Foxa2 *mutant pool, and using Q-PCR further validated strong reduction of *Hesx1 *(Additional File 1). We also confirmed *Foxd4 *is reduced in the ANE of *Foxa2 *mutants at E7.75 (compare Figure 2A and 2D), as was previously shown at E8.5 [34]. Together these results strengthen the model that tissue interaction within the anterior of the embryo proper is required for patterning of the forebrain, although the distinct contributions of the AME and DE will need to be investigated further.

We propose that the tetraploid *Foxa2 *mutant chimera will provide an important model for further studies on the interactions between organizer derivates and surrounding tissues in the gastrula embryo, and we are currently investigating the inductive effect of endoderm on heart mesoderm.

### Enrichment of Gene Ontology (GO) terms among genes reduced in *Foxa2 *mutants

As a first step towards understanding the functional relationships between genes that are differentially regulated in *Foxa2 *mutant embryos, we looked for enrichment of specific GO terms. We grouped genes as being expressed in the primary tissues affected in *Foxa2 *mutants (i.e. node, notochord, AME, DE), which are also regions of high Foxa2 activity, and in the secondary tissues affected but where *Foxa2 *is not expressed, as described above (i.e. PS, cardiac mesoderm, ANE, vasculature). We found genes grouped into primary and secondary *Foxa2 *mutant tissues had statistically significant GO terms (p ≤ 0.01 using GOFFA in ArrayTrack; [71]; Additional Files 1, 7, 8). GO terms such as "transcription factor activity" and "anterior/posterior pattern formation" were present in both groups (Table 2 and Additional Files 7, 8), and we took this to support the *Foxa2 *mutant phenotype having specific patterning defects due to the loss of organizer and its derivatives, and subsequent loss of interactions with surrounding tissues. As expected, within regions of high Foxa2 activity we found significant GO terms such as, "morphogenesis of an epithelium", "gastrulation", and "notochord development" (Table 2 and Additional File 7). Furthermore, among genes specific to secondary tissues we found GO terms such as, "cardiac muscle morphogenesis" and "forebrain development" (Table 2 and Additional File 8). Most importantly, the transcription factors we identified as being reduced in organizer derivatives may form the basis of a Foxa2-dependent gene regulatory network.

**Table 2:** Genes specific to affected tissues in *Foxa2 *mutants are enriched for Gene Ontology (GO) terms.

Affected tissues	GO Term	Specific Term	GO ID	P value* (Average)	E value	Gene Hits (Number)	Gene Hits (Symbols)
Primary:	Molecular function	transcription factor activity	GO:0003700	0.002335	5.08	5	Foxa1 Foxa2
-node							Foxd4
-notochord							Sox17
-anterior							T
mesendoderm	Biological process	anterior/posterior pattern formation	GO:0009952	0.000362	21.11	3	Cer1
-definitive endoderm	Biological process	morphogenesis of an epithelium	GO:0002009	0.000432	19.87	3	Foxa2 T
							Foxa1
							Foxa2
							T
	Biological process	gastrulation	GO:0007369	0.003025	24.25	2	Cer1 Foxa2
	Biological process	notochord development	GO:0030903	0.006329	157.63	1	T

Secondary:	Molecular function	transcription factor activity	GO:0003700	0.000053	4.49	10	Cdx1
							Foxb1
-primitive streak							Gbx2
-cardiac mesoderm							Hesx1
-anterior neuroectoderm							Hoxa1
-vasculature							Meis1
							Meox1
							Nkx1-2
							Six3
							Tbx6
	Biological process	anterior/posterior pattern formation	GO:0009952	0	21.77	7	Aldh1a2
							Dll1
							Gbx2
							Hoxa1
							Meox1
							Six3
							Wnt3a
	Biological process	cardiac muscle morphogenesis	GO:0055008	0.00012	116.09	2	Actc1
							Tnnt2
	Biological process	forebrain development	GO:0030900	0.000392	11.51	4	Aldh1a2
							Fabp7
							Six3
							Wnt3a

*significance for GO term enrichment: p ≤ 0.01

### Prediction of putative Foxa2 target genes

One of the difficulties of using the *Foxa2 *mutant embryo to identify genuine Foxa2 targets is the complete loss of organizer-derived tissues. This makes it difficult to distinguish between 1) genes reduced due to the absence of tissue, versus, 2) genes reduced due to the absence of their upstream activator. To begin classifying genes into these two distinct categories, and as a way to predict putative Foxa2 target genes, we used two independent methods to find conserved Foxa2 binding motifs.

We searched for Foxa2 binding motifs around all genes reduced in *Foxa2 *mutants *and *expressed in regions of Foxa2 activity (Additional Files 1, 9, 10, 11, 12). The first algorithm utilizes a precomputed database of known binding motifs found within conserved regions of mouse and human promoters (oPOSSUM; [72]). We found 9 out of 19 genes searched had conserved Foxa2 binding motifs within a 10 kb upstream and 5 kb downstream region around the transcriptional start site (Additional File 10). These represent strong candidates for Foxa2 target genes, and include both known and novel genes we identified as expressed in the node, AME and/or DE. Importantly, we found *Foxa2 *itself among this set of genes, consistent with studies in human hepatocytes that demonstrated Foxa2 binds its own promoter for autoregulation [73]. To focus more specifically on the gene regulatory program within the axial mesoderm, we also found conserved Brachyury/T binding motifs in a smaller subset of the above co-expressed genes (n = 3/19; Additional File 11). Surprisingly, one of these genes was *Cer1*, a gene expressed exclusively in the endoderm during gastrulation, suggesting Brachyury/T could have a role in repressing endoderm genes within the mesoderm.

**Figure 5:**
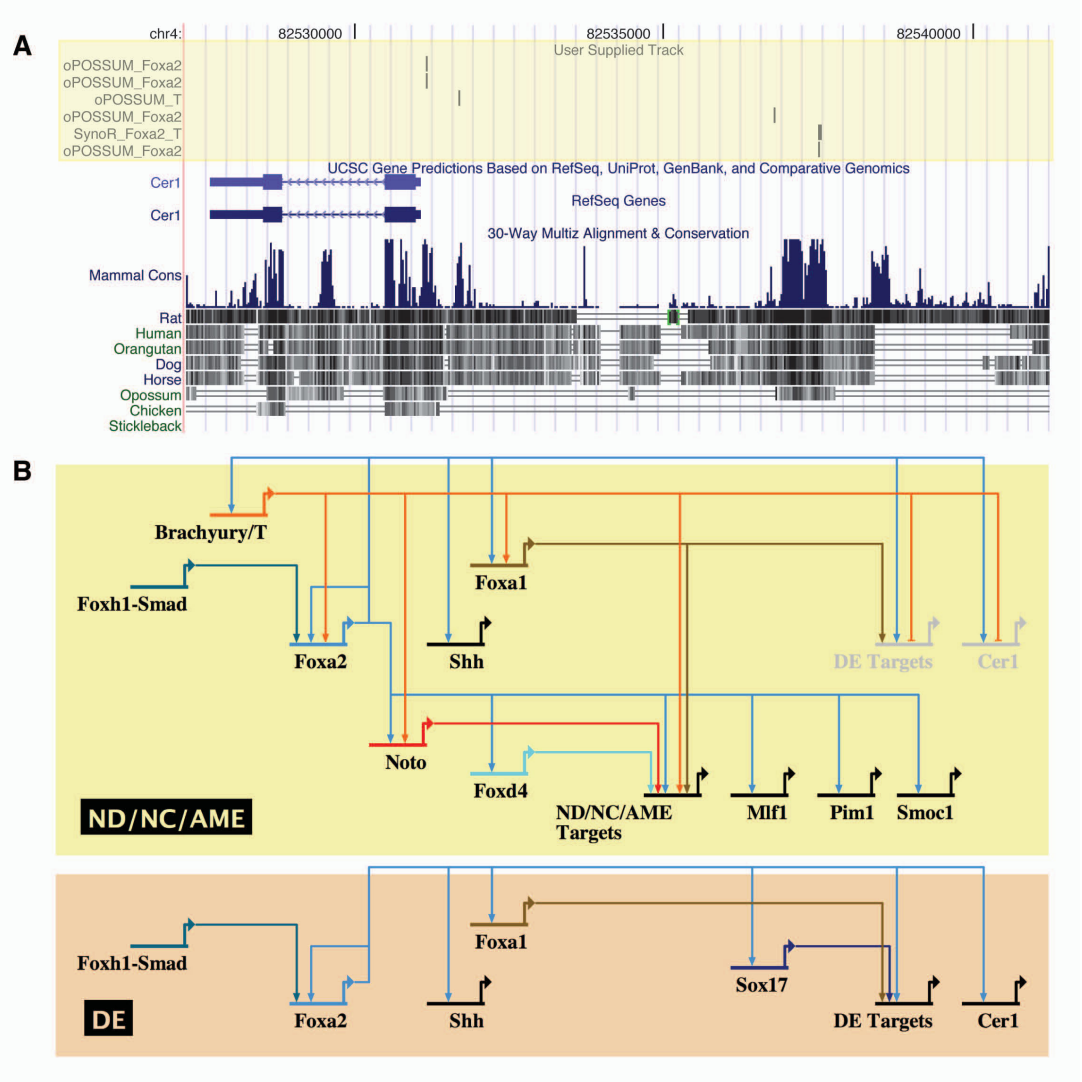
**A network model for Foxa2-dependent gene regulation in organizer derivatives**. (A) An example of binding motif prediction results visualized using the UCSC genome browser; [105]. Conserved Foxa2 and Brachyury/T binding motifs were found in the 10 kb region upstream of the endoderm-specific *Cer1 *gene. (B) A hypothetical network model based on existing genetic data (see Discussion for details and references), and binding motif predictions around putative Foxa2 target genes (Additional Files 1, 9, 10, 11, 12). Brachyury/T is active in node, notochord and axial mesendoderm (ND/NC/AME), but not in definitive endoderm (DE). The transcription factors Foxa2, Brachyury/T, Noto, Foxa1, and/or Foxd4 could regulate putative ND/NC/AME target genes; Foxa2, Foxa1, and/or Sox17 could regulate DE target genes. We propose a role for Brachyury/T in repression of DE targets in the ND/NC/AME lineages. Foxa2 likely regulates itself in both ND/NC/AME and DE. (Note: the network diagram was created using BioTapestry; [106]).

The second algorithm we used searches the entire genome for conserved regions that contain combinations of at least two DNA binding motifs (SynoR; [74]); in this case Foxa2 and Brachyury/T. The advantage of this approach is that it is not limited to a specific set of genes, or the relatively small genomic regions around genes' transcriptional start sites. We identified two of the same four genes as above (*Cer1 *and *Foxa2*) as potential targets of Foxa2 and Brachyury/T (Figure 5A; Additional File 12). The two methods we applied (oPOSSUM and SynoR) use different position weight matrix databases (JASPAR and TRANSFAC, respectively), which may explain why the predicted DNA binding motifs found by the two programs do not overlap. We combined our validated *Foxa2 *mutant microarray data, predictions of conserved Foxa2 and Brachyury/T binding motifs, as well as existing genetic data, to build a gene regulatory network model in the organizer and its derivates (Figure 5B), the details of which will be outlined in the following discussion.

## Discussion

### Microarray analysis of *Foxa2 *mutant gastrula embryos

Functionally based expression profiling of the early mouse embryo is emerging as a powerful method to elucidate the regulatory networks that underlie developmental programs. Comparison of the transcriptional profile between wild-type and mutant embryos has been done using subtractive cDNA libraries [26], and more recently using microarrays; for example, to study Wnt signaling during gastrulation using conditional β-catenin mutants [75], the pharyngeal region of *Tbx1 *mutants [76], and the mid-hindbrain organizer region in *Pax2 *mutants [77]. We have used microarrays for expression profiling of wild-type and *Foxa2 *mutant embryos at E7.5, which fail to form organizer-derived tissues, including the node, AME, and DE. We were able to identify both known and novel markers that were specific to these tissues, based on their reduction or absence in the *Foxa2 *mutant.

### Whole mount *in situ *expression screening

We found known markers of the node, AME, and DE had a ≥ 1.5 fold decrease in the *Foxa2 *mutant, and this enabled us to set a threshold for the first phase of the whole mount *in situ *hybridization screen (n = 106). We then examined a number of genes that fell below this threshold to evaluate the sensitivity of our experimental design (n = 46). We found that genes expressed in the node, because it is a small tissue and represents a small fraction of the total RNA, were difficult to detect by differential expression levels. Finally, we rehybridized our original biological samples to an updated microarray to expand the depth of our screen, and chose additional transcripts for analysis (n = 162). This second phase of our whole mount *in situ *hybridization screen more than doubled our total coverage of unique genes (n = 314). From the combination of these various approaches, we found 10 novel expression patterns in the node and 5 in the DE.

### Highly dynamic regional expression patterns within the developing primitive gut

Before this and other recent screens in the early mouse embryo [23-25,46], there were very few markers available that were specific to the definitive endoderm. Two of these screens extended their design to isolate regionally specific transcripts along the anterior-posterior axis of the gut [25,46]. Together, all of these markers will be critical for understanding the regional patterning of the gut during development, as well as for *in vitro *differentiation of ES cells into endoderm lineages for therapeutic use. Significant recent progress has also been made in understanding the fate map of gastrulation stage DE and how it gives rise to later regions of the gut endoderm [78-81]. From this data a highly dynamic model of endoderm formation is emerging.

The next challenge will be to understand the different spatial and temporal requirements of this myriad of endoderm genes. For example, there are endoderm genes that are only expressed at early stages of endoderm development (*Cer1*, [37]), and those that are uniformly expressed early on and then become highly regionalized (*Trh*, [36]). We have identified genes that later at E8.5 are restricted to the midgut and hindgut (*Ppp1r14a*; Figure 4I), as have others previously (*Sox17*, [35]; *Tmprss2*, [46]), highlighting anterior-posterior differences in the developing gut tube. Furthermore, the dorsal-ventral aspect of the primitive gut also has differential gene expression, as shown by the ventrally enriched genes we and others have identified (*Cpm*; Figure 4H; *Pyy*, [25]; *Trh*, [36]). Finally, we have also identified genes that appear to be expressed throughout many regions and stages of DE development (*Cldn4*; Figures 4A, 4B, 4C, 4D), which are structurally important for the development of the gut epithelium. A major outstanding question is whether or not these early regional expression patterns in the endoderm are predictive of later pattern in the gut and its associated organs.

### Loss of organizer induction in the *Foxa2 *mutant embryo

Our microarray analysis of *Foxa2 *mutant embryos derived by tetraploid aggregation represents a complete molecular profile of a gastrula embryo that has developed with all tissues intact, except the organizer and its derivatives. The functional nature of this screen has allowed us to detect not only primary organizer specific genes (e.g. node, notochord, and DE), but also the secondary reduction of tissues that rely on interaction with the organizer (e.g. cardiac mesoderm, PS, vascular progenitors, ANE). Importantly, all of these secondary tissues still form in the *Foxa2 *mutant, so it is not their specification that is compromised. In fact, the first studies that either genetically or physically ablated the organizer in the mouse were surprising because of how well the embryo was still patterned [10,11,82,83]. Significantly, there appear to be subsets of genes within these secondary tissues that are more reliant on organizer interactions than others; for example, we observed reduction in various cardiac mesoderm markers, whereas others have shown that there are other cardiac markers that are not reduced in these mutants [62]. This is suggestive of genetic programs that are more dependent on either a regulative or determinative mode of development. The analysis of the *Foxa2 *mutant we present here may point to a future direction in organizer research – the molecular basis of how the organizer functions to subtly refine its adjacent tissues.

### Building a network of Foxa2-dependent gene regulation in organizer derivatives

Although our understanding of the gene regulatory networks involved in vertebrate development is at an early stage, significant progress has been made in sea urchin [84], *Drosophila *[85], and ascidian embryos [86]. These invertebrate models provide an invaluable framework for predicting how more complex organisms must also organize genetic networks. Our interest is focused on specification of cell lineages that originate in the mouse gastrula organizer, and in particular how Foxa2 functions as a regulator within these tissues. Foxa2 is expressed early in the organizer and then throughout its development, as this population gives rise to axial mesoderm (notochord), DE (gut), and ventral neuroectoderm (floor plate) [5]. This raises the interesting question of how Foxa2 maintains regulation in distinct but closely associated lineages that segregate during gastrulation. To begin addressing this complex problem, we have built a putative gene regulatory network based on potential Foxa2 target genes (experimentally validated by either Q-PCR or whole mount *in situ *hybridization in *Foxa2 *mutants) that can be used as a testable model in future studies.

Studies in sea urchin embryos have shown that gene regulatory networks specify cell lineages in a number of ways [87], which include: 1) a means for initial acquisition of identity, 2) feedback loops to stabilize a regulatory state, 3) exclusion of alternate states, 4) production of a signal that is required for itself and adjacent cells, and 5) lineage-specific activation of genes. We present our network model with these concepts in mind.

A delicate balance between Wnt and Nodal pathways is required for the establishment and function of the vertebrate organizer (reviewed [50]). There is evidence that the Wnt/β-catenin pathway indirectly regulates *Foxa2 *through Tead proteins that bind a node-specific *Foxa2 *upstream enhancer [88]. Previous genetic studies have shown that Nodal signal, mediated in the nucleus through a Foxh1-Smad complex, is also upstream of *Foxa2 *in organizer lineages [89,90]. Our microarray results confirm this, as *Foxh1 *levels are not changed in *Foxa2 *mutants; a result that was further validated using Q-PCR (Additional File 1). Conversely, we also validated that *Foxa2 *levels are reduced in *Foxh1 *mutants at E7.5 (data not shown). Nodal-Foxh1-Smad is likely one of the mechanisms that organizer cells use to interpret their environmental cues for initial acquisition of identity.

Once an initial identity has been established in a cell lineage it must be stabilized. One mechanism for this is a positive feedback loop involving transcriptional regulators that reinforce each other's expression [87]. We found evidence for this using Foxa2 and Brachyury/T binding motif predictions; *Brachyury/T *may be regulated by Foxa2, and *Foxa2 *may be regulated by itself and Brachyury/T (Figure 5B; Additional File 12). Consistent with this idea, we have previously found that Foxa2 is expressed in AME precursors in the epiblast, which upregulate Brachyury/T protein after epithelial-to-mesenchymal transition (Burtscher & Lickert, 2008, manuscript submitted). This AME population stays positive for Foxa2 and Brachyury/T protein during the development of the axial mesoderm and anterior endoderm populations. Also, Foxa2 and Brachyury/T likely regulate *Foxa1*, another axial mesendoderm transcription factor. The presence of all three factors in a cell's nucleus would help "lock in" this particular fate. Furthermore, *Foxa2 *genetically interacts with a number of other transcription factors (i.e. *Noto *[91], *Lhx1 *[92], *Otx2 *[93], *Gsc *[94]) and further investigation may reveal that feedback loops work to reinforce cell fates in these contexts as well.

Another important step in specifying a given lineage is the exclusion of alternate cell fates. For example, the homeodomain transcription factor *Noto *is critical for promoting axial mesoderm fate (notochord) and repressing paraxial mesoderm fate (somite) [91,95]. Also, β-catenin has a role in promoting axial mesendoderm towards an endoderm fate, while repressing mesoderm fate [96]. Synergism between Foxa2 and Brachyury/T has been demonstrated in both ascidian and *Xenopus *for promotion of notochord fate [97,98], yet surprisingly we found conserved Foxa2 and Brachyury/T binding motifs associated with the endoderm gene *Cer1 *(Figure 5; Additional File 12). As Brachyury/T is not expressed in the endoderm, it would suggest that Brachyury/T might actively repress endoderm fate within the axial mesoderm. Studies in ascidian embryos have shown that the converse is true; that *Brachyury/T *is repressed in endoderm lineages (by β-catenin), which has the effect of inhibiting notochord fate [99]. There is evidence that Foxa2 can function as a repressor [31], so it is possible that Foxa2 may also work to exclude alternate fates. It will be interesting to explore further how Foxa2 and Brachyury/T interact with each other and their downstream targets, both positively and negatively, to sort out the multiple lineages that are produced during gastrulation.

A given cell lineage in the developing embryo does not develop in isolation, and proper specification usually involves production of a signal for maintenance of itself and patterning of adjacent tissues [87]. In the notochord, Shh is the most likely candidate for this lineage-specific signal. It is thought to have an autocrine function, in that the notochord is formed but not maintained in its absence, as well as a paracrine function, as it is necessary and sufficient for patterning of the adjacent ventral neural tube [43,100]. Foxa2 is a key regulator of *Shh *in both the notochord and the floor plate [17,18], connecting the production of a notochord-specific signal with our emerging Foxa2-dependent gene regulatory network.

The culmination of lineage specification is the activation of a host of genes that enable cells to perform their designated function. The notochord's two primary functions during embryogenesis are signaling and structural (reviewed [101]), which in the mouse embryo can be broadly assigned to earlier and later stages, respectively. Our *Foxa2 *mutant screen was performed at E7.5, which we expect is too early to detect disruption of the structural genes expressed later in differentiation of mature notochord. Our binding motif predictions identified novel node genes *Mlf1*, *Pim1*, and *Smoc1 *as potential downstream targets of Foxa2 in the notochord, as well as *Cer1 *in the endoderm (Figure 5B; Additional File 12). We could detect reduced *Shh *levels, and therefore one of the direct signaling targets of Foxa2. However, because of the early stage of our screen, we would predict that detectable Foxa2 targets would be in the top tier of the axial mesendoderm gene regulatory network, and few of the notochord differentiation genes would have been activated. This is supported by the identification of a number of other transcription factors among our group of putative Foxa2 targets (Additional File 12).

## Conclusion

We have conducted functional microarray and expression pattern screens based on the *Foxa2 *mutant embryo. These screens detected differentially regulated genes because of the absence of a critical transcription factor and phenotypic loss of organizer-derived tissues in the early embryo. These data have provided not only novel regionally specific gene expression patterns in the node, AME and DE, but also putative downstream targets of Foxa2 and potential new genes regulating organizer biology. This data has allowed us to build a model of the gene regulatory network involved in Spemann/Mangold organizer formation.

Note Added in Proof

While this manuscript was in preparation, Schweickert and colleagues identified *Galanin *in a screen for asymmetrically expressed genes, based on its expression pattern in the heart. They also noted its expression in the node, notochord and PS between E7.5 and E8.5 [102].

## Methods

### Microarrays

Tetraploid embryos were derived as previously described [29,30] using *Foxa2 *null ES cells [12]. Wild-type embryos were collected from timed matings of ICR mice. Wild-type or tetraploid embryos were collected at E7.5 and staged accordingly [103]. Embryos were between mid-streak and head-fold stages and the extra-embryonic regions were removed by dissection. The embryo proper, including extra-embryonic tetraploid-derived VE, was retained and multiple samples were pooled for total RNA isolation using Trizol reagent (Invitrogen). Numbers of E7.5 embryos collected and pooled were as follows: wild-type 1 (n = 53), wild-type 2 (n = 50), *Foxa2 *null 1 (n = 53), *Foxa2 *null 2 (n = 48) (Additional File 13 details the staging of embryos). Total RNA from each of the four embryo pools was processed for Affymetrix U74Av2 GeneChip microarrays, as previously described [75]. The same hybridization mixtures of these biological samples were later used for hybridization to the upgraded Affymetrix MOE430v2 GeneChip microarrays. All microarray data has been submitted to the Gene Expression Omnibus (GEO) at NCBI (accession number: GSE5424).

### Data analysis

Affymetrix MAS 5.0 software was applied to the U74Av2 GeneChip data to target normalize the global expression level to 1000, and provide present, absent and fold change calls in cross-comparisons of all replicate mutant samples over the two replicate wild-type samples. Next probe sets with absent calls in all samples were removed from the data set. As well, all samples with illogical calls were also removed, for example a probe set with an increase call that was called absent in the sample with the higher level of expression. Lastly, we filtered the data to include only probe sets that showed a statistical change call in two or more (2/4) of the cross comparisons.

MOE430v2 GeneChips were analyzed as follows in the R statistical programming language (R Core Development Team, 2006; Additional File 5). Probeset summaries were calculated according to the MAS 5.0 algorithm. Logarithmic summary values were normalized by the loess smoother [104] applied to the M-A scale transform. Statistical testing for differential expression between mutant and wild-type was performed on single probe level taking the duplicate chips into account employing the Wilcoxon paired rank test. The *p*-value threshold was adjusted by either family-wise error rate (FWER, Bonferroni procedure) or the false discovery rate (FDR, Bejamini-Hochberg technique). Significant probesets were tested for enrichment of GO terms by the hypergeometric distribution testing with the annotation as available from the GO consortium on March 25^th^, 2005.

### Whole mount *in situ *hybridization

Whole mount *in situ *hybridization on E7.5–9.0 mouse embryos was performed as previously described [96], however digestion with proteinase K was replaced with a 20 minute 3% hydrogen peroxide incubation step. Antisense RNA *in situ *probes were transcribed accordingly from sequence-verified cDNA clones (see Methods and Additional Files 1, 2, 4, 6). Described expression patterns are representative of at least three or more stage-matched embryos.

### Quantitative real-time PCR

A third biological replicate pool each of wild-type (n = 47) and *Foxa2 *null (n = 33) embryos were collected, as above, for independent validation of gene expression levels using quantitative real-time PCR. Total RNA was isolated using Trizol reagent (Invitrogen) and provided the template for subsequent cDNA synthesis (Qiagen QuantiTect Rev. Transcription Kit). Quantitative real-time PCR was performed using the Roche LightCycler 480 and SYBR green reagent. See Additional File 1 for primer sequences and details. Transcripts were measured as a ratio of *Foxa2 *null levels compared to wild-type and normalized to endogenous *Hprt *levels as a reference.

## Abbreviations

DE: Definitive Endoderm; EGO: early gastrula organizer; MGO: mid-gastrula organizer; PS: primitive streak; VE: visceral endoderm; AME: anterior mesendoderm; ANE: anterior neuroectoderm; ES: embryonic stem; APS: anterior primitive streak; SAGE: serial analysis of gene expression; GFP: green fluorescent protein; EB: embryoid body; ND: node; NC: notochord; GO: gene ontology.

## Authors' contributions

OJT and DK shared equally in conducting the screen and identifying novel gene expression patterns. OJT conducted additional validation, bioinformatics experiments, and drafted the manuscript. BJC conceived and performed the initial microarray and data analysis. CEB performed some whole mount *in situ *hybridization, imaging, and database compilation. JR supervised the study and finalized the manuscript. HL conceived of the study, designed the overall project, supervised the study, and finalized the manuscript. All authors have approved the final manuscript.

## Supplementary Material

Additional file 1 **Supplementary materials and methods: ** page 2 abbreviations used in supplementary tables. Page 3 Primers for generating IVT Templates. Page 4 Q-PCR methods. Page 5 Q-PCR primers. Page 6 Gene Ontology analysis using GOFFA. Page 7 oPOSSUM methods Page 8 SynoR methods. Click here for file

Additional file 2 **Supplementary Table 1.** List of genes screened by whole mount *in situ *hybridization that had a ≥ 1.5 fold decrease in *Foxa2 *mutants, as detected by the Affymetrix U74Av2 array. Click here for file

Additional file 3 **Supplementary Figures.** Additional whole mount *in situ* images organized alphabetically by current MGI gene symbol. Click here for file

Additional file 4 **Supplementary Table 2.** List of genes screened by whole mount *in situ *hybridization that did not meet the 1.5 fold decrease threshold in *Foxa2 *mutants, as detected by the Affymetrix U74Av2 array. Click here for file

Additional file 5 **Analysis of Foxa expression data by Heiko Lickert.** Report and details of Affymetrix MOE430v2 GeneChip data analysis. Click here for file

Additional file 6 **Supplementary Table 3.** List of genes screened by whole mount *in situ *hybridization that were significantly reduced in *Foxa2 *mutants, as detected by the Affymetrix MOE430v2 array. Click here for file

Additional file 7 **Supplementary Table 4.** Gene Ontology (GO) terms significantly enriched (p ≤ 0.01) among genes expressed in the primary tissues affected in Foxa2 mutants. Click here for file

Additional file 8 **Supplementary Table 5.** Gene Ontology (GO) terms significantly enriched (p ≤ 0.01) among genes expressed in the secondary tissues affected in Foxa2 mutants. Click here for file

Additional file 9 **Supplementary Table 6.** oPOSSUM output: TF motifs identified in promoters of genes reduced in Foxa2 mutants and expressed in regions of Foxa2 activity. Click here for file

Additional file 10 **Supplementary Table 7.** oPOSSUM output: putative target genes with conserved Foxa2 binding motifs. Click here for file

Additional file 11 **Supplementary Table 8.** oPOSSUM output: putative target genes with conserved Brachyury/T binding motifs. Click here for file

Additional file 12 **Supplementary Table 9.** Summary of conserved Foxa2 and T binding motif predictions around putative Foxa2 target genes. Click here for file

Additional file 13 **Starting material for *Foxa2 *expression profiling.** Details of the numbers and stages of embryos collected for the screen. Click here for file
