# Supplementary material for: Microarray analysis of Foxa2 mutant mouse embryos reveals novel gene expression and inductive roles for the gastrula organizer and its derivatives
Source: BMC Genomics. 2008 Oct 30;9:511. doi: 10.1186/1471-2164-9-511 (PMC2605479; doi:10.1186/1471-2164-9-511)
Supplement: Additional file 1 — Supplementary materials and methods: page 2 abbreviations used in supplementary tables. Page 3 Primers for generating IVT Templates. Page 4 Q-PCR methods. Page 5 Q-PCR primers. Page 6 Gene Ontology analysis using GOFFA. Page 7 oPOSSUM methods Page 8 SynoR methods. [file 1471-2164-9-511-S1.pdf]

Tamplin et al. 2008
Supplementary Materials and Methods.

Table of Contents:

Page 2 Abbreviations used in Supplementary Tables 1-3

Page 3 Primers for generating IVT Templates

Page 4 Q-PCR methods

Page 5 Q-PCR primers

Page 6 Gene Ontology analysis using GOFFA

Page 7 oPOSSUM methods

Page 8 SynoR methods

ABBREVIATIONS USED IN SUPPLEMENTARY TABLES:

ADE	ANTERIOR DEFINITIVE ENDODERM
AL	ALLANTOIS
AME	ANTERIOR MESENDODERM
ANE	ANTERIOR NEUROECTODERM
BA	BRANCHIAL ARCHES
BAL	BASE OF ALLANTOIS
CH	CHORION
CM	CARDIAC MESODERM
EN	ENDODERM
EP	EPIBLAST
EXE	EXTRA-EMBRYONIC
FB	FOREBRAIN
HB	HINDBRAIN
HG	HINDGUT
IFT	INFLOW TRACT
LPM	LATERAL PLATE MESODERM
MB	MIDBRAIN
ME	MESODERM
MG	MIDGUT
NA	NOT AVAILABLE
NC	NOTOCHORD
ND	NODE
NT	NEURAL TUBE
OFT	OUTFLOW TRACT
OV	OTIC VESICLE
PA	PARAXIAL MESODERM
PE	PRIMITIVE ENDODERM
PP	PRECHORDAL PLATE
PS	PRIMITIVE STREAK
PSM	PRESOMITIC MESODERM
RH	RHOMBOMERE
RS	REGIONALLY-SPECIFIC
SO	SOMITES
ST	SEPTUM TRANSVERSUM
SU	STRONGLY UBIQUITOUS
TB	TAILBUD
VA	VARIOUS OTHER TISSUES
VE	VESSELS
WN	WEAK TO NO STAINING
WS	WIDESPREAD
WU	WEAKLY UBIQUITOUS

Description of columns in Supplementary Tables (Additional Files) 1-3:

U74Av2/MOE430v2 probe	Affymetrix GeneChip probeset ID number
Gene Symbol	Current MGI gene symbol, if available
Clone ID	EST used to generate anti-sense probe for in situ and/or reference to relevant published expression data
Clone Vector	vector backbone for reference to PCR protocol (see below, p. 2) and polymerase used to transcribe anti-sense probe
Expression at E7.5	brief description of expression pattern (see abbreviations above)
Expression at E8.5	brief description of expression pattern (see abbreviations above)
Average signal log ratio	of duplicate Foxa2 null pools compared to wild-type
Internal Lab ID #	internal reference for organizer of clones
E7.5 image available	reference to image in Supplementary Figures, Additional File 12
E8.5 image available	reference to image in Supplementary Figures, Additional File 12
E9.0 image available	reference to image in Supplementary Figures, Additional File 12

Primers and PCR Programs for IVT templates; polymerases for antisense riboprobes:

Vector (antisense polymerase)	Primer Name	Primer Sequence	PCR Program
pBluescript-Lion (SP6)	Lion-5	AGCGTGGTCGCGGCCGAGGT	94°C 3’15”<br>95°C 45”<br>56°C 45”<br>72°C 1’<br>30 cycles<br>72°C 4’
	Lion-3-Sp6	ATTTAGGTGACACTATAGAATCGAGCGGCCGCCCGGGCAGGT	
pSPORT1 (SP6)<br>pCMV-SPORT6 (T7)<br>pCMV-SPORT2 (T7)<br>pT7T3D-Pac1 (T3)	TM-M13 FWD	GTAAAACGACGGCCAGT	96°C 2’<br>96°C 10”<br>52°C 5”<br>72°C 4’<br>30 cycles<br>72°C 4’
	TM-M13 REV	CAGGAAACAGCTATGAC	
pBluescript-modified (T3)	RZPD-M13 FWD	GCTATTACGCCAGCTGGCGAAAGGGGGATGTG	94°C 3’15”<br>95°C 45”<br>68°C 45”<br>72°C 4’<br>30 cycles<br>72°C 4’
	RZPD-M13 REV	CCCCAGGCTTTACACTTTATGCTTCCGGCTCG	
pME18s-Fl3 (T7)	SuganoF1-Sp6	ATTTAGGTGACACTATACAACTGCTCCTCAGTGGATGTTGCCTTTAC	94°C 3’15”<br>95°C 45”<br>56°C 45”<br>72°C 4’<br>30 cycles<br>72°C 4’
	SuganoR1-T7	TAATACGACTCACTATAGGACAGGTTCAGGGGGAGGTGTGG	
pSPORT1 (SP6)<br>alternate protocol	pSPORT1 FWD	GCTATTACGCCAGCTGGCGAAAGGGGGATGTG	94°C 2’<br>94°C 30”<br>65°C 30”<br>72°C 2’<br>30 cycles<br>72°C 5’
	pSPORT1 REV	CCCCAGGCTTTACACTTTATGCTTCCGGCTCG	
pCMV-SPORT6 (T7)<br>alternate protocol	pCMV-SPORT6 FWD	ACAAAGATCCCAAGCTAGCAG	94°C 2’<br>94°C 30”<br>60°C 30”<br>72°C 3’<br>30 cycles<br>72°C 5’
	pCMV-SPORT6 REV	TTGACCTCCATAGAAGACACC	
pBSII SK- (T7)	pBSII SK- FWD	GTTTTCCCAGTCACGACGTT	
	pBSII SK- REV	TGTGGAATTGTGAGCGGATA	
pT7T3D (T3)<br>alternate protocol	pT7T3D FWD	GTTTTCCCAGTCACGACGTT	
	pT7T3D REV	TGTGGAATTGTGAGCGGATA	
Ank3 clone (T7)	pCMV-SPORT6 FWD	ACAAAGATCCCAAGCTAGCAG	
	Ank3 REV	CCTAAACCCTGTGTGCCTGT	

Quantitative PCR to validate expression levels in *Foxa2* mutant embryo pool:
- transcripts were measured as a ratio of *Foxa2* null pool levels compared to wild-type pool
- levels were normalized to endogenous *Hprt* levels
- 16/20 markers tested, 80% confirmed the microarray results
- 1 known ubiquitous marker unchanged (Foxh1)
- 9 primary tissue markers reduced (see below)
- 6 secondary tissue markers reduced (see below)

cDNA	Normalized Ratio	Normalized Ratio STD	log ratio base 2
Foxd4	0.04	0.01	-4.64385619
Gal	0.1	0.02	-3.321928095
Foxa1	0.31	0.09	-1.689659879
T	0.37	0.07	-1.434402824
Prnp	0.81	0.13	-0.304006187
Cer1	0.2	0.05	-2.321928095
Itga3	0.66	0.2	-0.59946207
Sox17	0.67	0.15	-0.577766999
Cldn4	0.76	0.21	-0.395928676
Hesx1	0.08	0.01	-3.64385619
Wnt3a	0.34	0.12	-1.556393349
Tnnt2	0.44	0.07	-1.184424571
Hoxa1	0.57	0.08	-0.810966176
Wnt8a	0.69	0.29	-0.535331733
Flt4	0.8	0.11	-0.321928095
Foxh1	0.91	0.27	-0.13606155
Foxa2*	0.29	0.77	-1.785875195
Actc1*	0.89	0.2	-0.168122759
Smoc1*	4.65	1.2	2.217230716
Tie1*	3.18	0.96	1.669026766

*Q-PCR of these transcripts did not confirm the microarray results (i.e. large STD or increase instead of decrease)

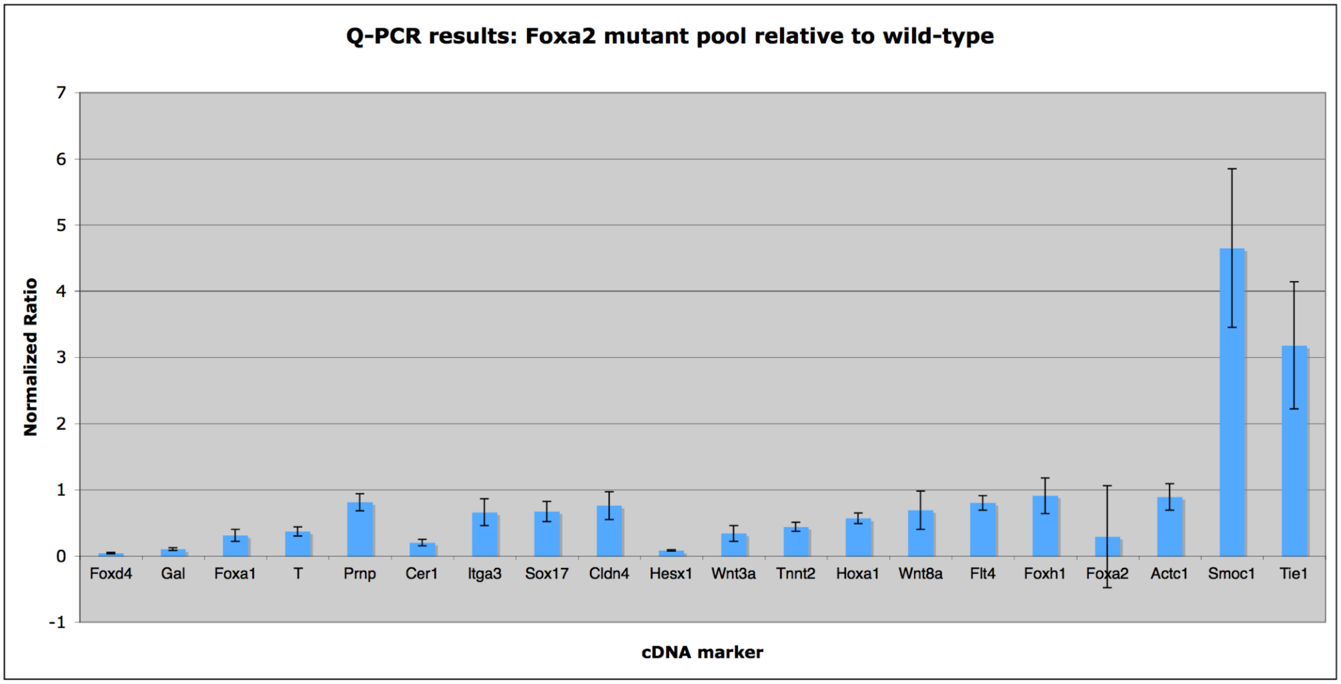

Primers used for Quantitative PCR (all T_m ~60°C):

Primer Name	Primer Sequence
OT-Foxd4-FWD-Q-PCR	CGGAAGAAGAGGATGACGAG
OT-Foxd4-REV-Q-PCR	GAGGAACCCGGAGGAGTTAC
OT-Gal-FWD-Q-PCR	GAGAGGTTGGACCCTGAACA
OT-Gal-REV-Q-PCR	GGTCTCCTTTCCTCCACCTC
OT-Foxa1-FWD-Q-PCR	CATGAGAGCAACGACTGGAA
OT-Foxa1-REV-Q-PCR	TGTTGCTGACAGGGACAGAG
OT-T-FWD-Q-PCR	TCCCGAGACCCAGTTCATAG
OT-T-REV-Q-PCR	TTCTTTGGCATCAAGGAAGG
OT-Prnp-FWD-Q-PCR	GCATTCTGCCTTCCTAGTGG
OT-Prnp-REV-Q-PCR	GGTTCGCCATGATGACTGAT
OT-Cer1-FWD-Q-PCR	GTCATCCTGCCCATCAAAAG
OT-Cer1-REV-Q-PCR	ATTTGCCAAAGCAAAGGTTG
OT-Itga3-FWD-Q-PCR	TGTCTTCCACGGCTTCTTCT
OT-Itga3-REV-Q-PCR	TCATGGCAATGACGATAGGA
OT-Sox17-FWD-Q-PCR	CAGAACCCAGATCTGCACAA
OT-Sox17-REV-Q-PCR	GCTTCTCTGCCAAGGTCAAC
OT-Cldn4-FWD-Q-PCR	GGGGATCATCCTGAGTTGTG
OT-Cldn4-REV-Q-PCR	CACTGCATCTGACCTGTGCT
OT-Hesx1-FWD-Q-PCR	ACAGACCCTGGACAGACACC
OT-Hesx1-REV-Q-PCR	CTTTCTTCTGGCCTTGGATG
OT-Wnt3a-FWD-Q-PCR	ATGGCTCCTCTCGGATACCT
OT-Wnt3a-REV-Q-PCR	GGGCATGATCTCCACGTAGT
OT-Tnnt2-FWD-Q-PCR	CTGAGACAGAGGAGGCCAAC
OT-Tnnt2-REV-Q-PCR	TTCTCGAAGTGAGCCTCGAT
OT-Hoxa1-FWD-Q-PCR	GCCCTGGCCACGTATAATAA
OT-Hoxa1-REV-Q-PCR	GAGCTGCTTGGTGGTGAAAT
OT-Wnt8a-FWD-Q-PCR	CCATCATGTACGCAGTCACC
OT-Wnt8a-REV-Q-PCR	GCCCTGTTGTTGTGAAGGTT
OT-Flt4-FWD-Q-PCR	CCCAGCCATGTACAGAAGGT
OT-Flt4-REV-Q-PCR	GGCTGGAGTCAGAGGAGTTG
OT-Foxh1-FWD-Q-PCR	GACGACTATGAGGGCTGGAA
OT-Foxh1-REV-Q-PCR	AGCAGGAATCAGGCTCACAT
OT-Foxa2-FWD-Q-PCR	CCCGGGACTTAACTGTAACG
OT-Foxa2-REV-Q-PCR	TTGCTCACGGAAGAGTAGCC
OT-Actc1-FWD-Q-PCR	CGATATCCGCAAAGACCTGT
OT-Actc1-REV-Q-PCR	GCTGGAAGGTGGACAGAGAG
OT-Smoc1-FWD-Q-PCR	AAGAGCATAGAGGCCGATGA
OT-Smoc1-REV-Q-PCR	CCTGAACCATGTCTGTGGTG
OT-Tie1-FWD-Q-PCR	CAGGCACAGCAGGTTGTAGA
OT-Tie1-REV-Q-PCR	GTGCCACCATTTTGACACTG
OT-Hprt-FWD-Q-PCR	CAGGCCAGACTTTGTTGGAT
OT-Hprt-REV-Q-PCR	TTGCGCTCATCTTAGGCTTT

Gene Ontology analysis using GOFFA (available through ArrayTrack software):

Select data type:	Gene name
Select array type:	Affy_Mouse430_2 (i.e. the reference genes; *N*=total number of genes on the chip)
Input Data:	primary or secondary gene lists, as below (*M*=number of genes in input set; m_i=subset of *M* that belongs to a GO term *i*)
Output Data:	select terms with p≤0.01 as indicative of statistically significant finding

Genes expressed in the primary tissues affected in Foxa2 *mutants (M=20;* m_i*=17):*

1700009P17Rik
1700027A23Rik
Cer1
Cldn4
Cpm
Cyb561
Foxa1
Foxa2
Foxd4
Gal
Gstm5
Josd2
Mlf1
Nptx2
Pim1
Prnp
Smoc1
Sox17
T
Trh

Genes expressed in the secondary tissues affected in Foxa2 *mutants (M=41;* m_i*=40):*

Actc1
Actc1
Alcam
Aldh1a2
Arg1
Cdc14b
Cdkn1c
Cdx1
Cnn2
Col1a1
Crabp1
Dll1
Efhd2
Fabp7
Fgfbp3
Flt4
Foxb1
Frzb
Gbx2
Hesx1
Hoxa1
Igfbp3
Meis1
Meox1
Mgst1
Myl1
Myl7
Nkx1-2
Pak1
Pcdh19
Pcsk1n
Ripk3
Scd2
Six3
Tagln
Tbx6
Tie1
Tmsb4x
Tnnt2
Wnt3a
Wnt8a

Transcription factor binding motif prediction method 1:

oPOSSUM (Ho Sui et al., 2005)

http://www.cisreg.ca/cgi-bin/oPOSSUM/opossum

STEP 1: Enter a list of co-expressed genes	STEP 2: Select transcription factor binding site matrices	STEP 3: Select parameters
Species:<br>Mouse<br><br>Gene ID type:<br>MGI Symbol<br><br>Paste gene IDs:<br>1700009P17Rik<br>1700027A23Rik<br>Cer1<br>Cldn4<br>Cpm<br>Cyb561<br>Foxa1<br>Foxa2<br>Foxd4<br>Gal<br>Gstm5<br>Josd2<br>Mlf1<br>Nptx2<br>Pim1<br>Prnp<br>Smoc1<br>Sox17<br>T<br>Trh	JASPAR CORE Profiles<br>select by taxonomic supergroup:<br>vertebrate	Level of conservation:<br>Top 10% of conserved regions (min. conservation 70%)<br><br>Matrix match threshold:<br>80%<br><br>Amount of upstream / downstream sequence:<br>10000 / 5000<br><br>Number of results to display:<br>All<br><br>Sort results by:<br>Z-score<br><br>SUBMIT

Analysis Results
Selected Parameters
Conservation level: *Top 10% of conserved regions (min. conservation 70%)*
Matrix match score: *80%*
Upstream sequence length: *10000*
Downstream sequence length: *5000*
Number of genes submitted: *20*
Number of genes included: *19*
Number of genes excluded: *1*
Target Genes
Analyzed: *Gal Cyb561 Nptx2 Foxa2 Foxa1 Pim1 Gstm5 Cpm Cer1 Josd2 Sox17 T Prnp Trh 1700027A23Rik Mlf1 Foxd4 1700009P17Rik Smoc1*
Excluded: *Cldn4*

(Note: see Additional Files 6-8 for relevant oPOSSUM output tables)

Transcription factor binding motif prediction method 2:

SynoR (Ovcharenko and Nobrega, 2005)

http://synor.dcode.org/

**STEP 1: List transcription factors**	**STEP 2: Specify distance limitations between neighboring binding sites**	**STEP 3: Select genomes**
BRACH_01 (Brachyury/T)<br>HNF3B_01 (Foxa2/Hnf3-beta)	at least 4 bps, but not more than 200 bps	base genome: mouse (mm9)<br>comparison genome(s): human (hg18)

Note: raw data filtered for genes down-regulated in *Foxa2* mutants and co-expressed with *Foxa2* (see gene list above used for oPOSSUM)—results are summarized in Additional File 9.
