# Supplementary material for: Microarray analysis of Foxa2 mutant mouse embryos reveals novel gene expression and inductive roles for the gastrula organizer and its derivatives
Source: BMC Genomics. 2008 Oct 30;9:511. doi: 10.1186/1471-2164-9-511 (PMC2605479; doi:10.1186/1471-2164-9-511)
Supplement: Additional file 3 — Supplementary Figures. Additional whole mount in situ images organized alphabetically by current MGI gene symbol. [file 1471-2164-9-511-S3.pdf]

Tamplin et al. 2008
Supplementary Figures

Pages 1-17

Additional whole mount in situ images organized alphabetically by current MGI gene symbol.
Supplementary Tables 1-3 indicate whether or not a screened transcript has an image included and its stage.

File annotation is as follows:

'gene symbol_stage_view/orientation.extension'

Some images have an additional note for relative length of time the *in situ* colour reaction was developed:

'gene symbol_stage_view/orientation(_long/short).extension'

Scale bars, if included, are all 200μm, except the following, which are 500μm:

1810037I17Rik_E9.0
Atm_E9.0
BC003993_E8.5
Cd276_E8.5
Cldn4_E8.5
Cldn4_E9.0
Cyb561_E8.5
Fabp7_E9.0
Galt_E9.0
Gpx2_E8.5
Gstm5_E8.5
Hrmt1l2_E9.0
Ina_E9.0
Meis1_E8.5
Meis1_E9.0
Mfap2_E8.5
Ppp1r1a_E9.0
Pts_E8.5
Pura_E8.5
Pygb_E8.5
Rap2b_E8.5
Rbmx_E8.5
Scn1a_E8.5
Slc1a5_E8.5
Smoc1_E9.0
Snx5_E8.5
Tpbg_E9.0
Trh_E9.0
Wfdc2_E8.5

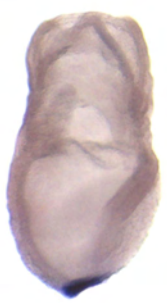

1700009P17Rik_E7.5_lat.jpg

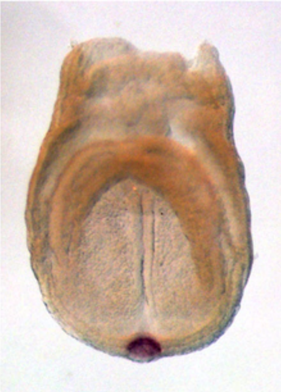

1700009P17Rik_E7.75_ant.jpg

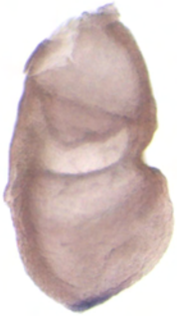

1700027A23Rik_E7.5_lat.jpg

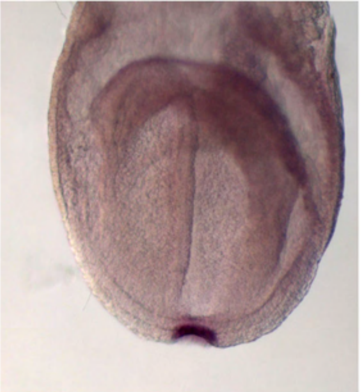

1700027A23Rik_E7.75_ant.jpg

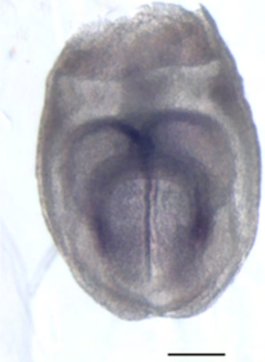

1810037I17Rik_E7.75_ant.jpg

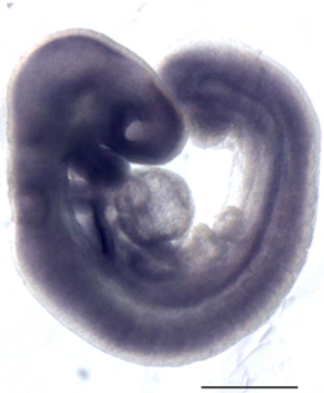

1810037I17Rik_E9.0_lat.jpg

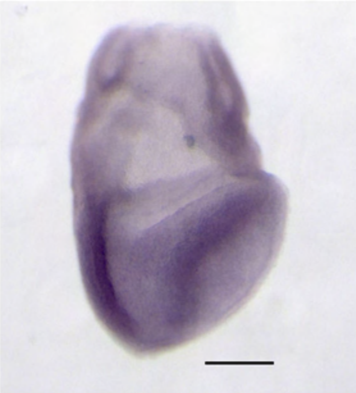

2610524G07Rik_E7.5_lat.jpg

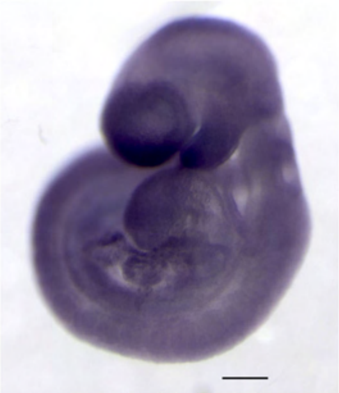

2610524G07Rik_E9.0_lat.jpg

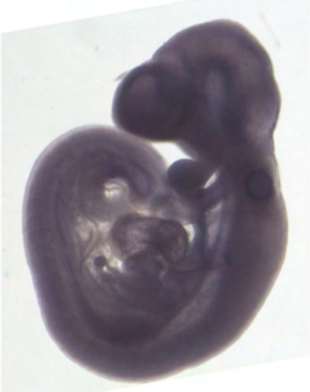

2700038N03Rik_E9.5_lat.jpg

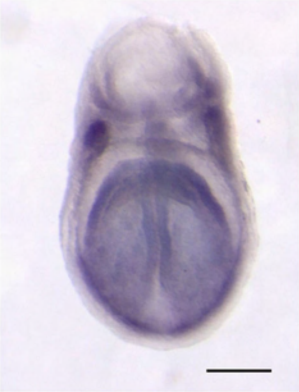

2810040C05Rik_E7.5_ant.jpg

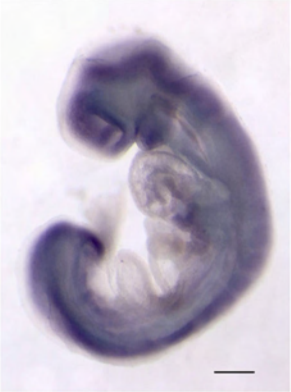

2810040C05Rik_E8.5_lat.jpg

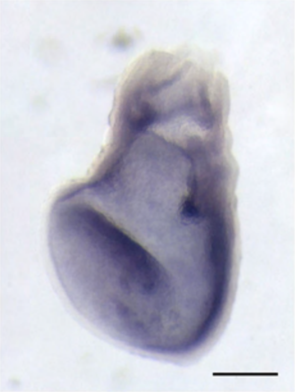

3732412D22Rik_E7.5_lat.jpg

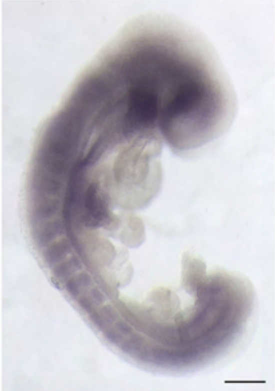

3732412D22Rik_E8.5_lat.jpg

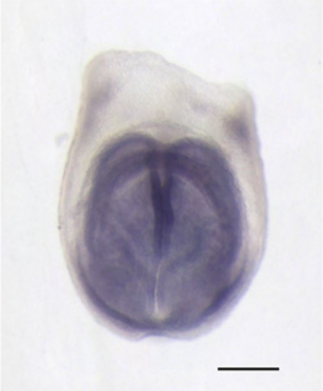

4833420G17Rik_E7.5_ant.jpg

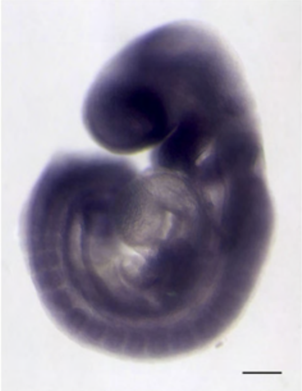

4833420G17Rik_E9.0_lat.jpg

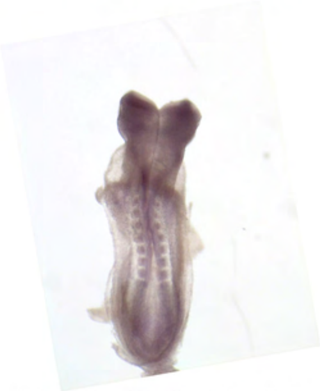

4921506J03Rik_E8.5_dorsal.jpg

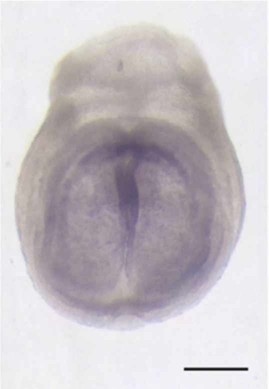

6330403K07Rik_E7.5_ant.jpg

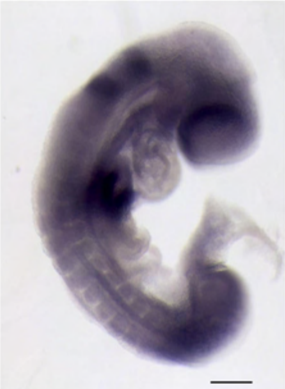

6330403K07Rik_E8.5_lat.jpg

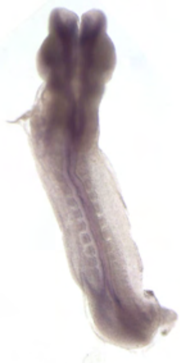

Agpat1_E8.5_dorsal.jpg

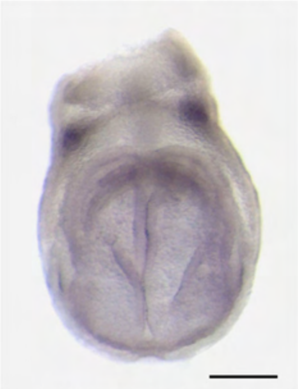

AI506816_E7.75_ant.jpg

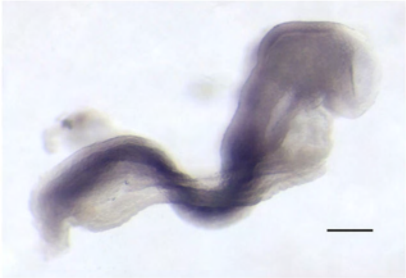
AI506816_E8.5_lat.jpg

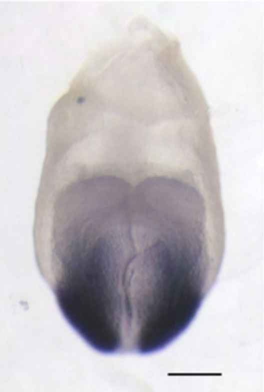
Aldh1a2_E7.75_ant.jpg

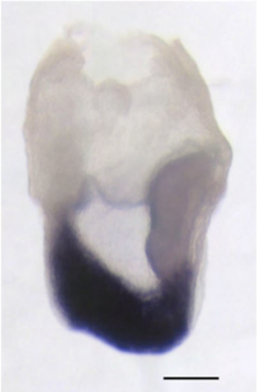
Aldh1a2_E7.75_lat.jpg

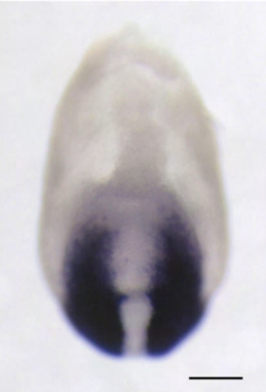
Aldh1a2_E7.75_post.jpg

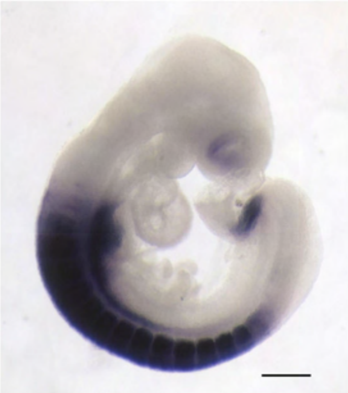
Aldh1a2_E8.5_lat.jpg

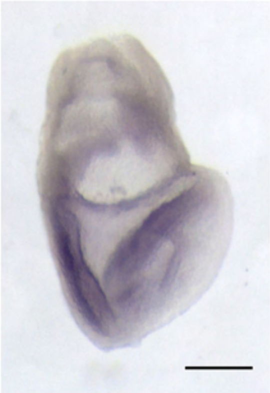
Ank3_E7.5_lat.jpg

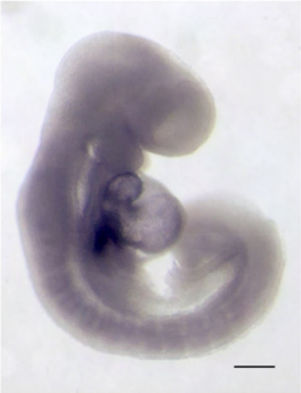
Ank3_E8.5_lat.jpg

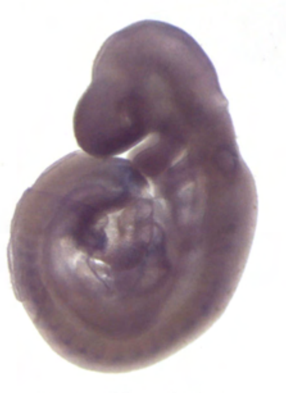
Anxa5_E9.5_lat.jpg

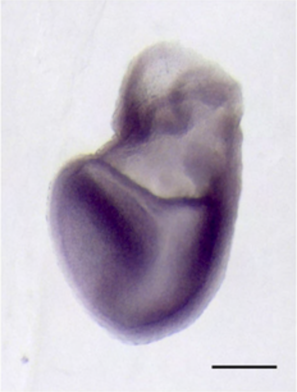
Ap4s1_E7.5_lat.jpg

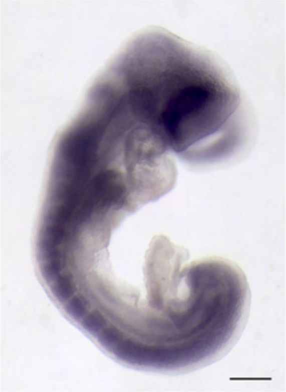
Ap4s1_E8.5_lat.jpg

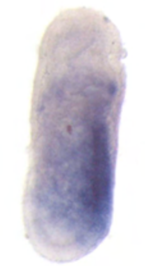
Aplnr_E7.5_lat.jpg

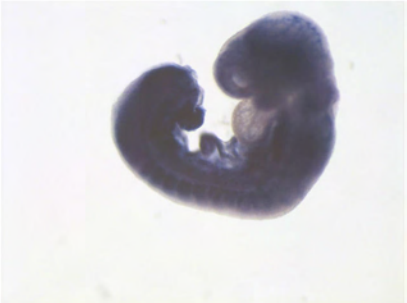
Aplnr_E9.0_lat.jpg

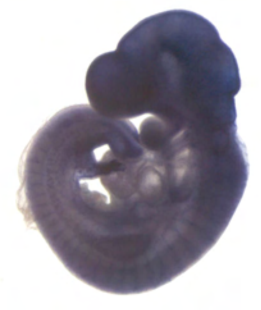
Aplnr_E9.5_lat.jpg

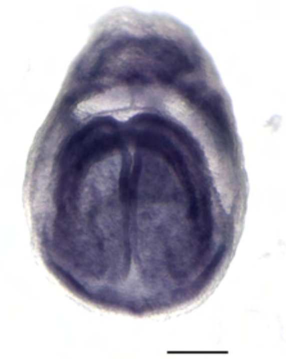
App_E7.75_ant.jpg

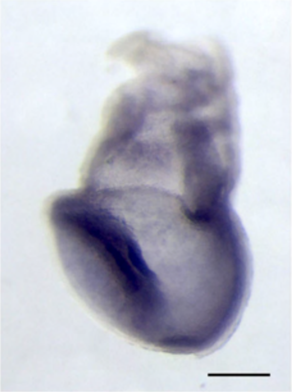
App_E7.75_lat.jpg

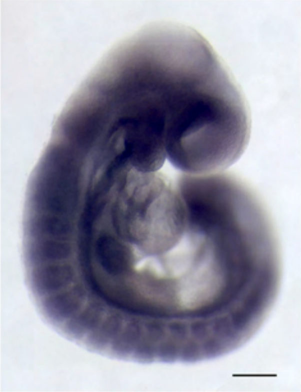
App_E9.0_lat.jpg

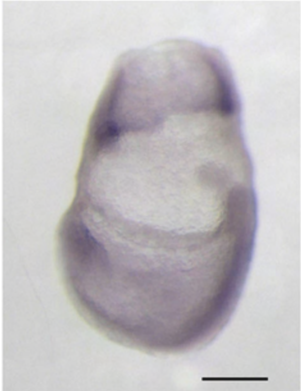
Arcn1_E7.5_lat.jpg

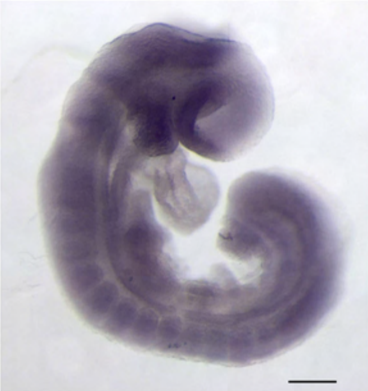
Arcn1_E8.5_lat.jpg

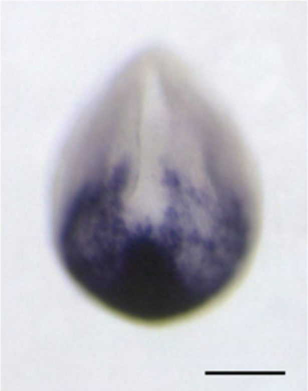
Arg1_E7.5_distal.jpg

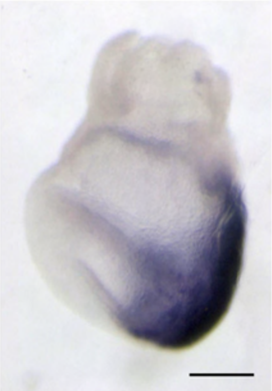
Arg1_E7.5_lat.jpg

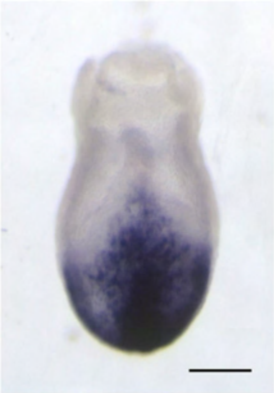
Arg1_E7.5_post.jpg

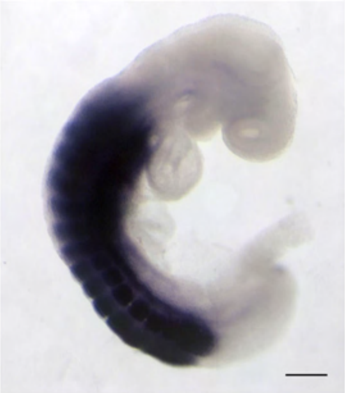
Arg1_E8.5_lat.jpg

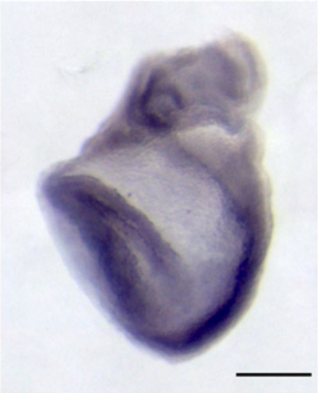
Arl6ip1_E7.5_lat.jpg

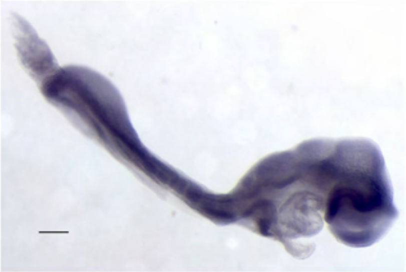
Arl6ip1_E8.5_lat.jpg

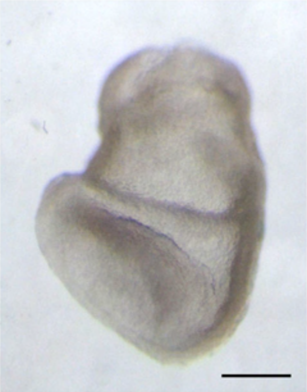
Astn1_E7.5_lat.jpg

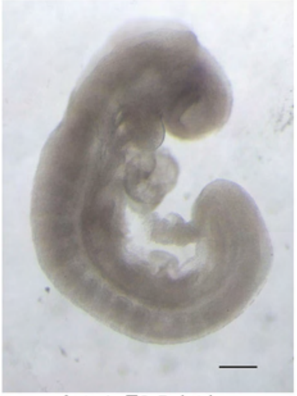
Astn1_E8.5_lat.jpg

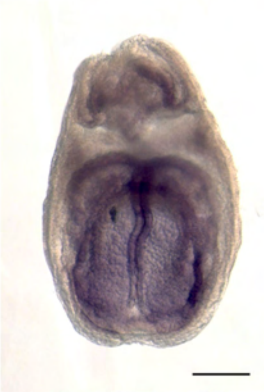
Atm_E7.75_lat.jpg

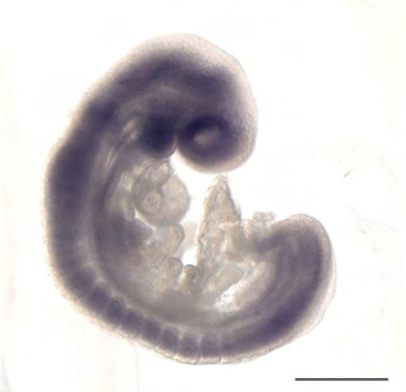
Atm_E9.0_lat.jpg

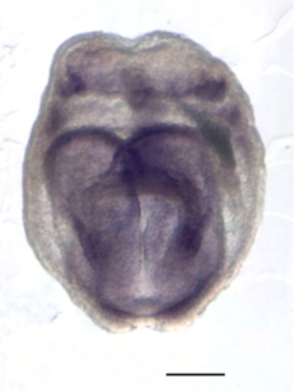
BC003993_E7.75_ant.jpg

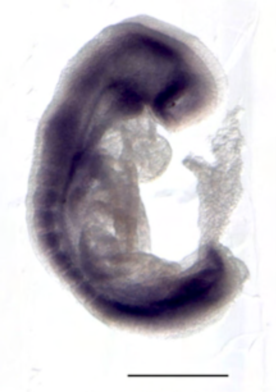
BC003993_E8.5_lat.jpg

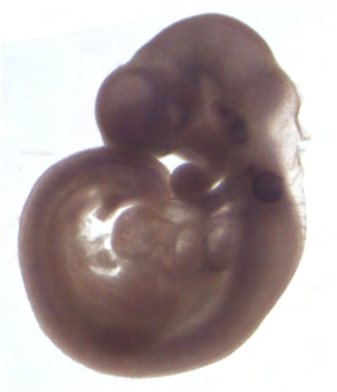
BC067047_E9.5_lat.jpg

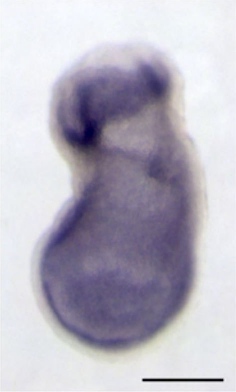
Bcl2l2_E7.25_lat.jpg

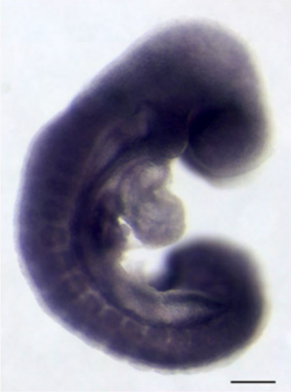
Bcl2l2_E8.5_lat.jpg

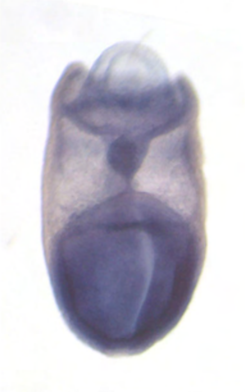
Brwd1_E7.5_ant.jpg

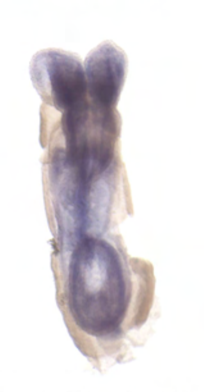
Brwd1_E8.5_dorsal.jpg

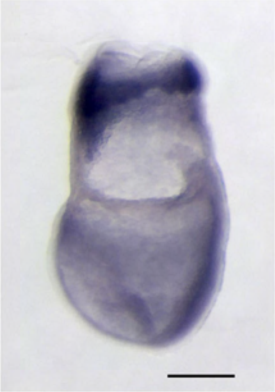
C77545_E7.5_lat.jpg

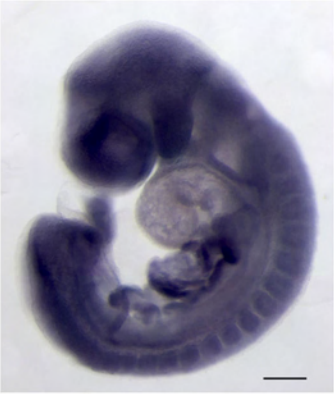
C77545_E8.5_lat.jpg

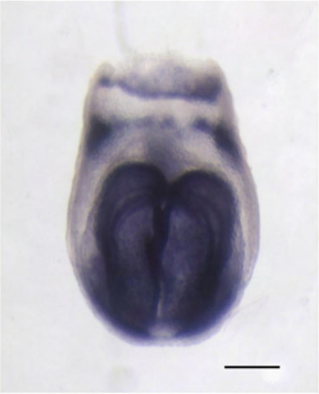
Cbx3_E7.5_ant.jpg

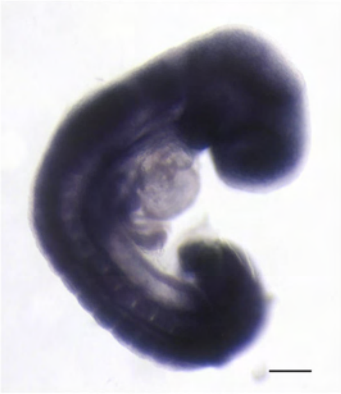
Cbx3_E8.5_lat.jpg

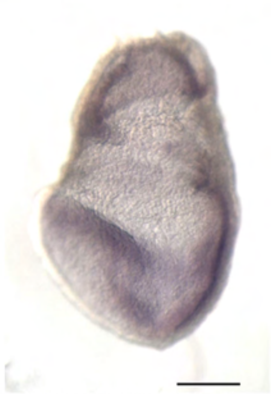
Cd276_E7.5_lat.jpg

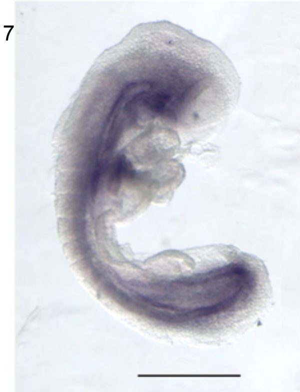
Cd276_E8.5_lat.jpg

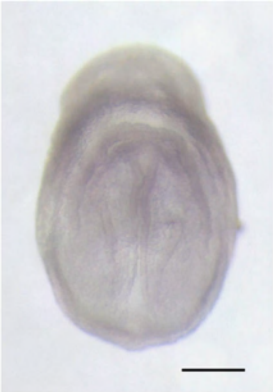
Cd59a_E7.5_ant.jpg

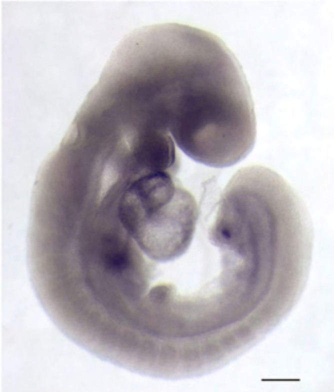
Cd59a_E9.0_lat.jpg

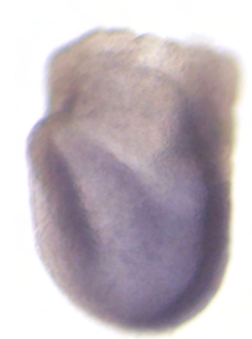
Cdc14b_E7.5_lat.jpg

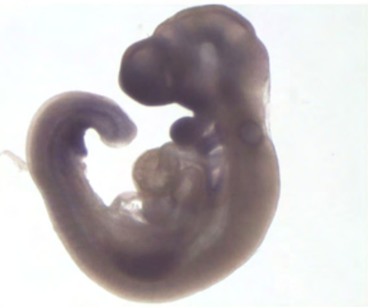
Cdc14b_E9.5_lat.jpg

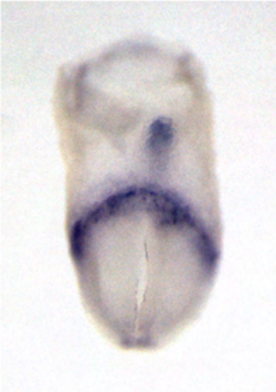
Cdkn1c_E7.5_ant.jpg

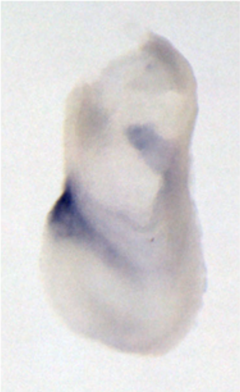
Cdkn1c_E7.5_lat.jpg

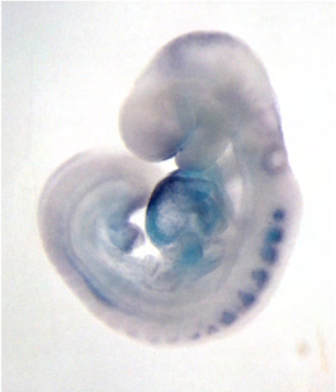
Cdkn1c_E9.0_lat.jpg

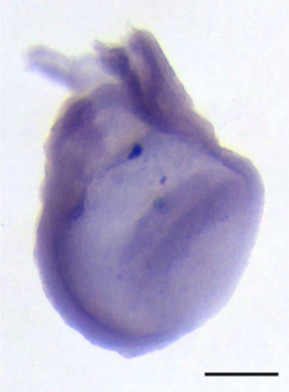
Cklfsf8_E7.5_lat.jpg

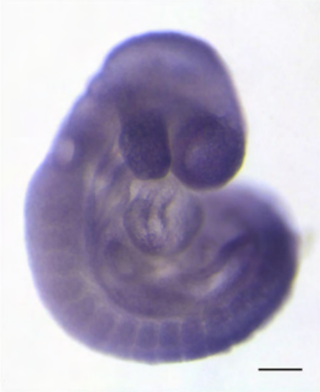
Cklfsf8_E9.0_lat.jpg

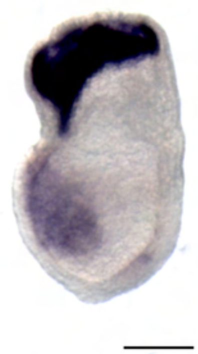
Cldn4_E7.5_lat.jpg

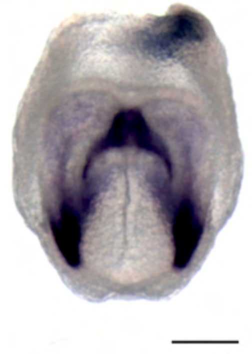
Cldn4_E7.75_ant.jpg

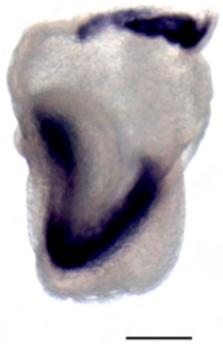
Cldn4_E7.75_lat.jpg

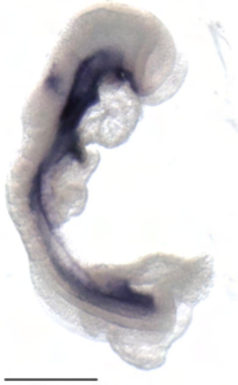
Cldn4_E8.5_lat.jpg

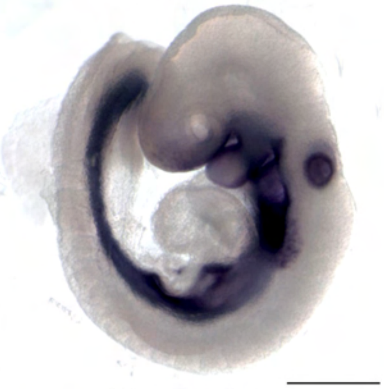
Cldn4_E9.0_lat.jpg

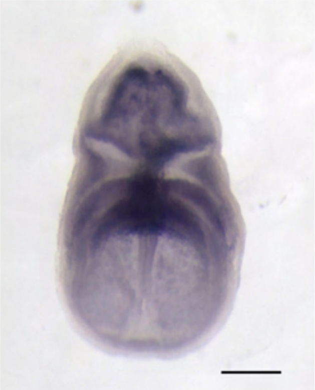
Col1a1_E7.5_ant.jpg

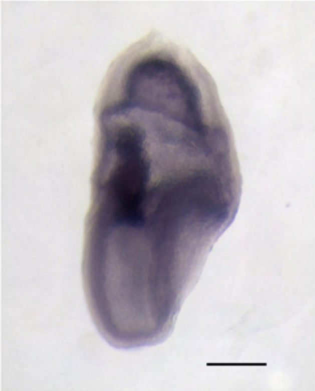
Col1a1_E7.5_lat.jpg

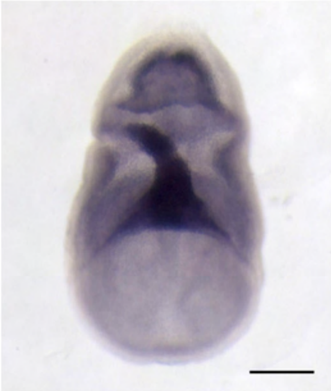
Col1a1_E7.5_post.jpg

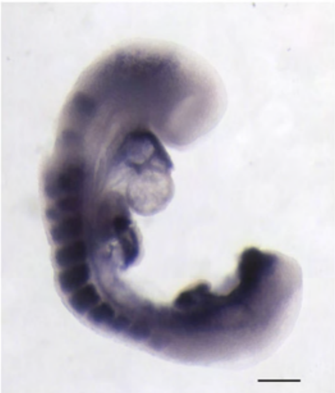
Col1a1_E8.5_lat.jpg

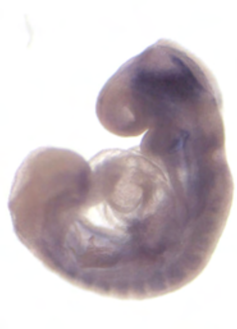
Colec12_E9.0_lat.jpg

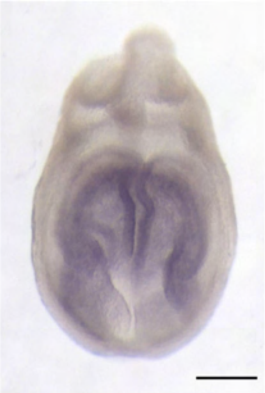
Cox7b_E7.5_ant.jpg

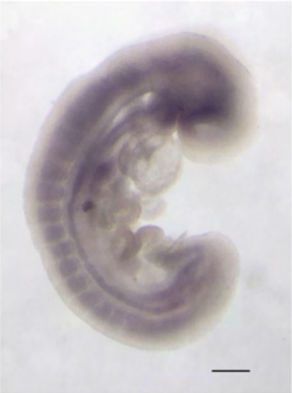
Cox7b_E8.5_lat.jpg

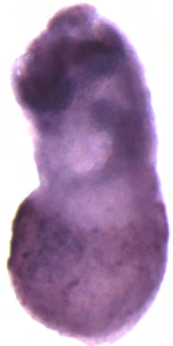
Cpm_E7.5_lat_long.jpg

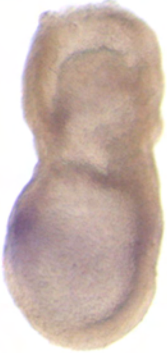
Cpm_E7.5_lat_short.jpg

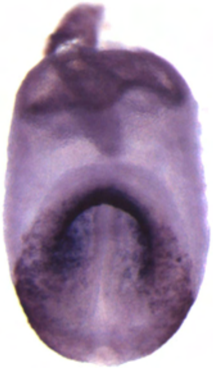
Cpm_E7.75_ant_long.jpg

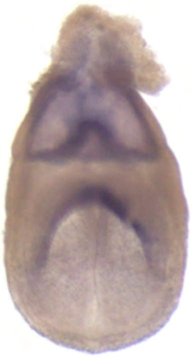
Cpm_E7.75_ant_short.jpg

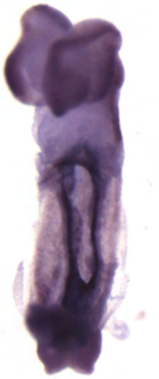
Cpm_E8.5_ventral_long.jpg

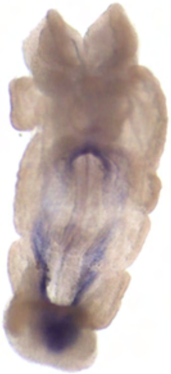
Cpm_E8.5_ventral_short.jpg

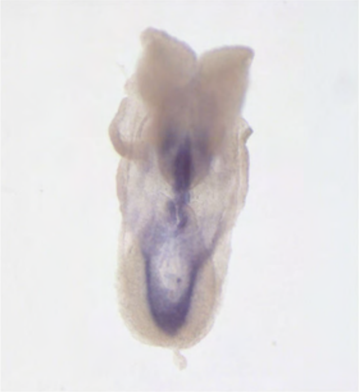
Cpn1_E8.5_dorsal.jpg

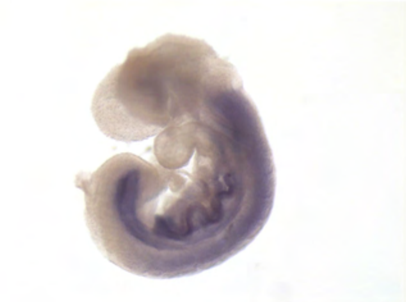
Cpn1_E9.0_lat.jpg

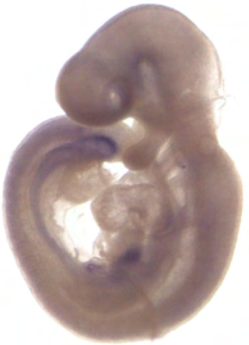
Cpn1_E9.5_lat.jpg

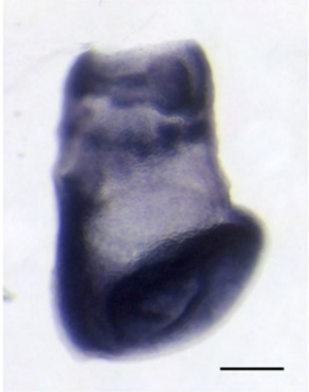
Csnk2a1_E7.5_lat.jpg

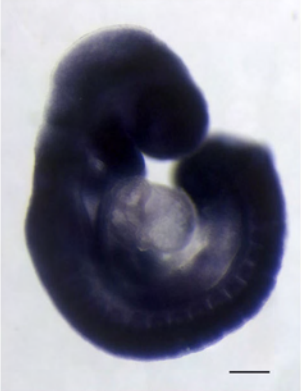
Csnk2a1_E9.0_lat.jpg

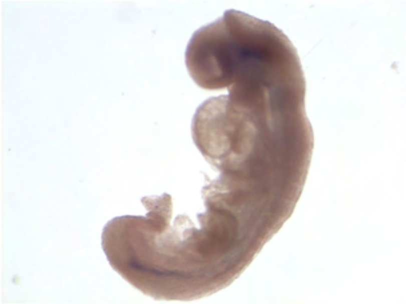
Cthrc1_E90_lat.jpg

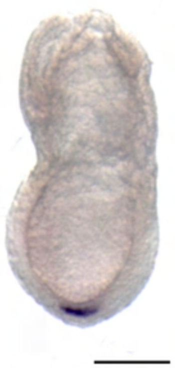
Cyb561_E7.5_lat.jpg

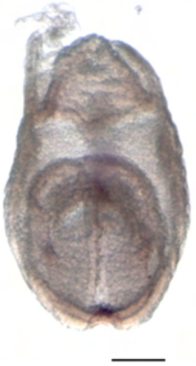
Cyb561_E7.75_ant.jpg

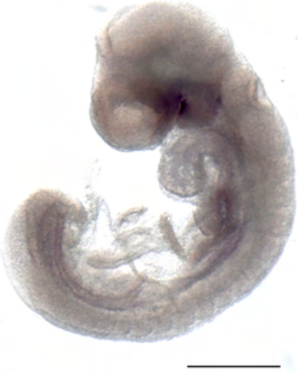
Cyb561_E8.5_lat.jpg

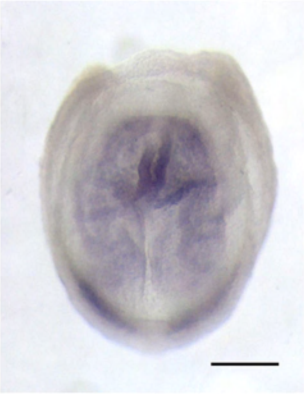
Cyp51_E7.75_ant.jpg

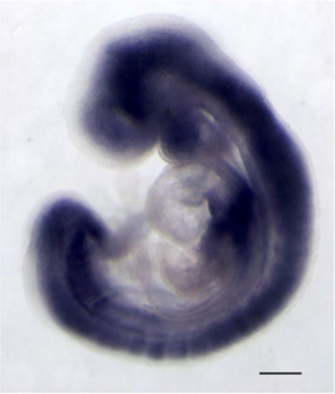
Cyp51_E8.5_lat.jpg

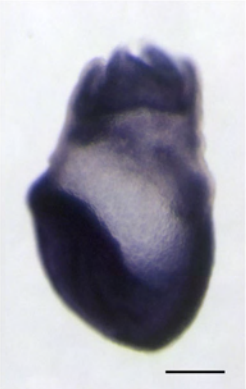
Ddx6_E7.5_lat.jpg

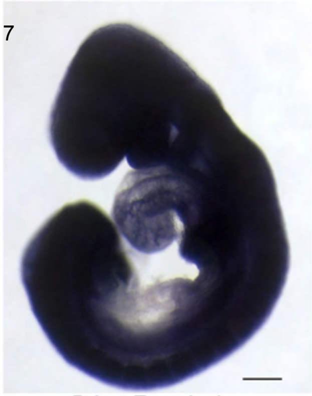
Ddx6_E9.0_lat.jpg

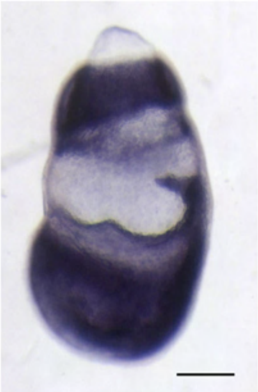
Defcr6_E7.5_lat.jpg

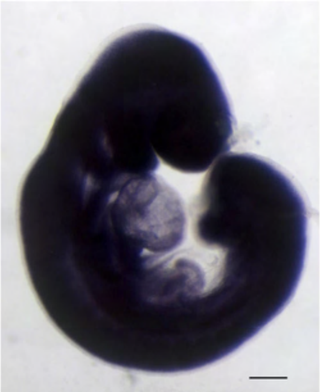
Defcr6_E9.0_lat.jpg

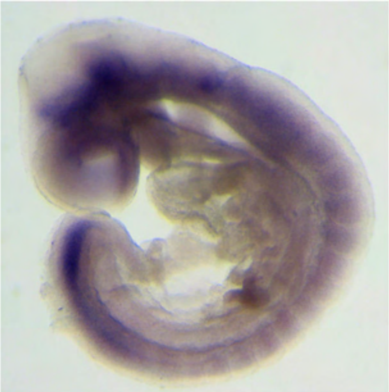
Edg2_E9.0_lat.jpg

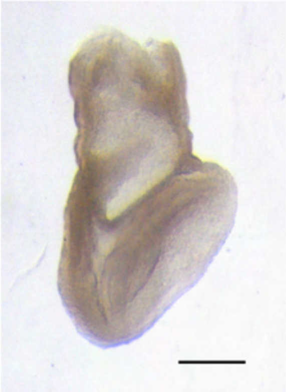
Efhd1_E7.5_lat.jpg

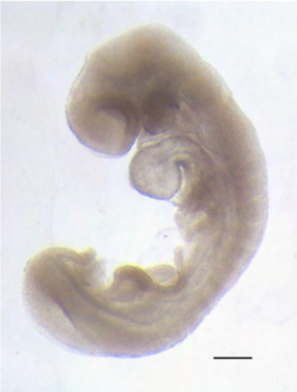
Efhd1_E8.5_lat.jpg

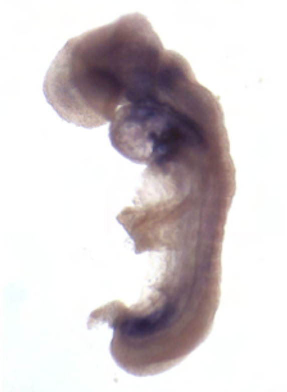
Efhd2_E8.5_lat_b.jpg

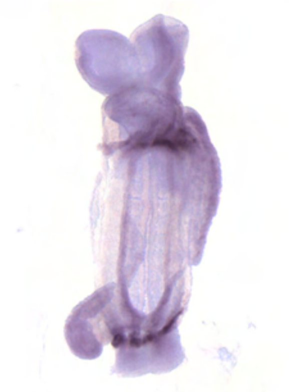
Efhd2_E8.5_ventral.jpg

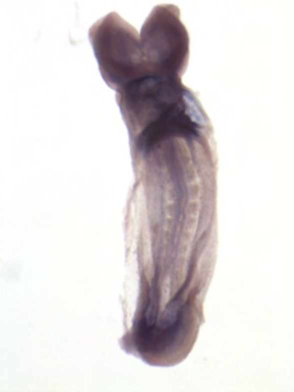
Efhd2_E8.5_ventral_b.jpg

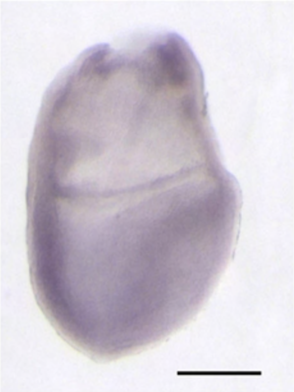
Esd_E7.5_lat.jpg

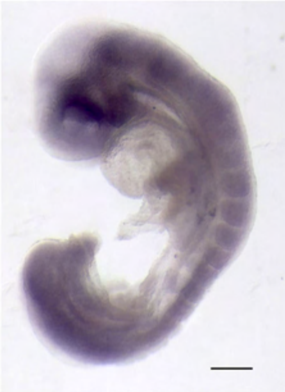
Esd_E8.5_lat.jpg

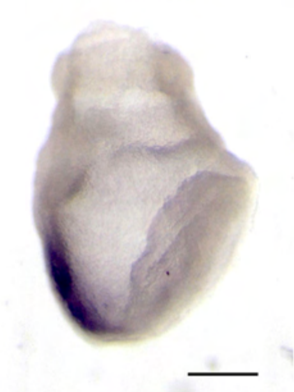
Fabp7_E7.5_lat.jpg

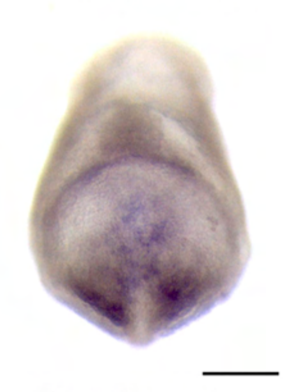
Fabp7_E7.5_post.jpg

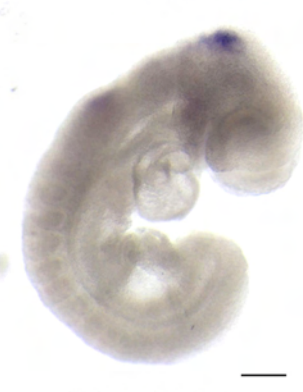
Fabp7_E8.5_lat.jpg

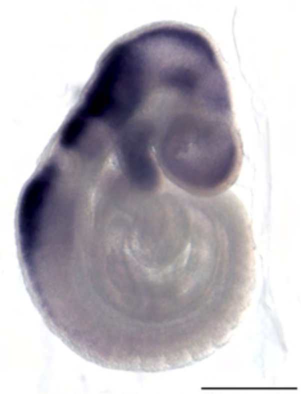
Fabp7_E9.0_lat.jpg

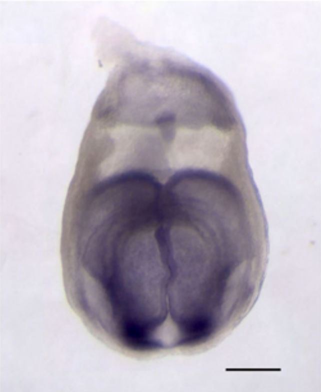
Fgfr2_E7.75_ant.jpg

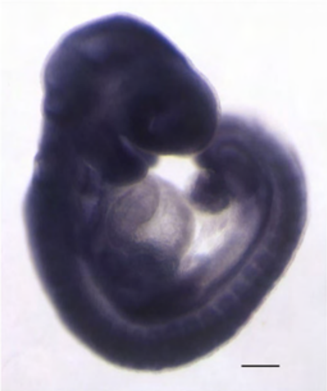
Fgfr2_E9.0_lat.jpg

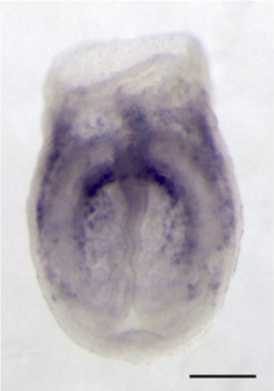
Flt4_E7.75_ant.jpg

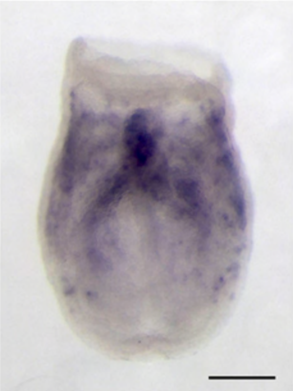
Flt4_E7.75_post.jpg

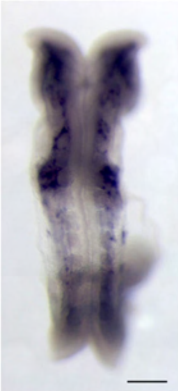
Flt4_E8.5_dorsal.jpg

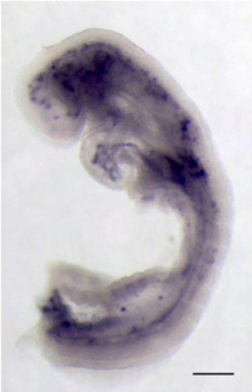
Flt4_E8.5_lat.jpg

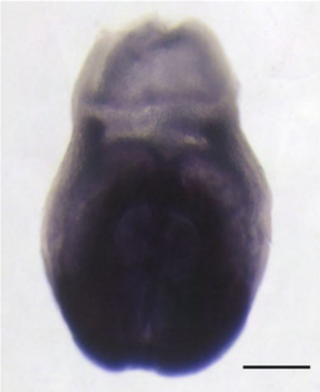
Fstl1_E7.75_ant.jpg

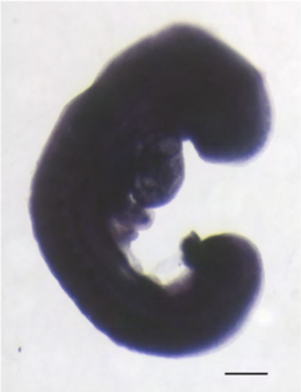
Fstl1_E8.5_lat.jpg

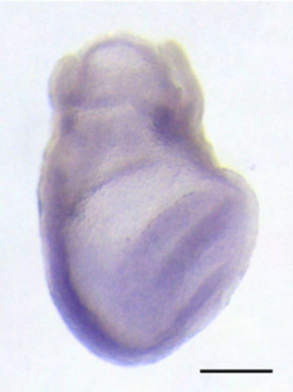
Fv4_E7.5_lat.jpg

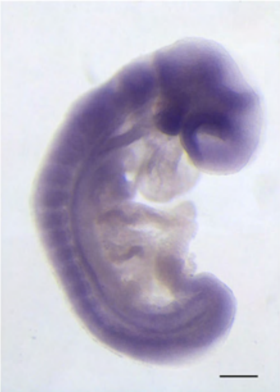
Fv4_E8.5_lat.jpg

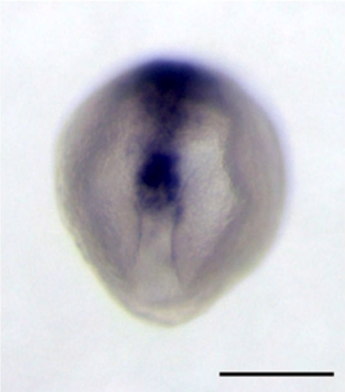
Gal_E7.5_distal.jpg

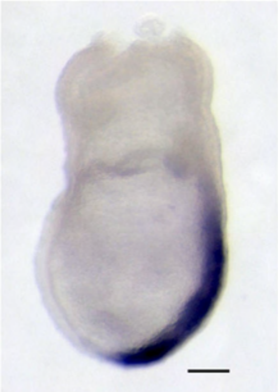
Gal_E7.5_lat.jpg

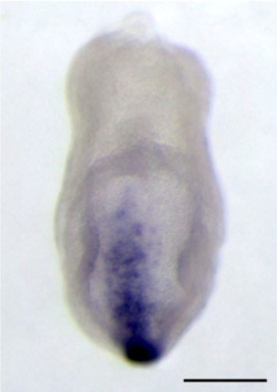
Gal_E7.5_post.jpg

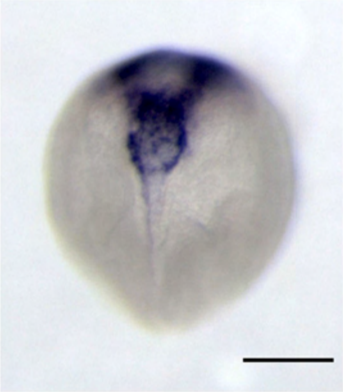
Gal_E7.75_distal.jpg

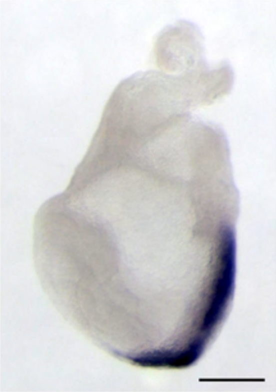
Gal_E7.75_lat.jpg

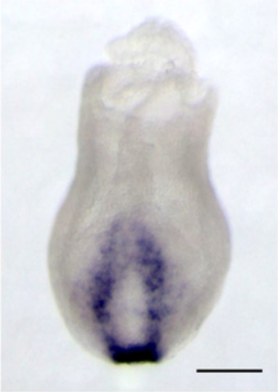
Gal_E7.75_post.jpg

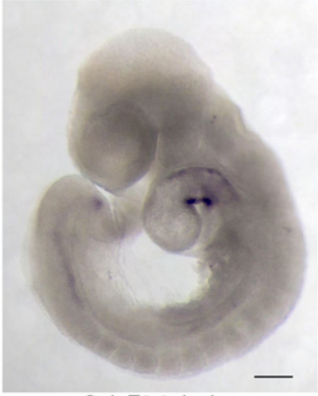
Gal_E9.0_lat.jpg

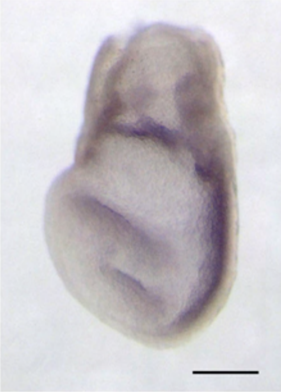
Galt_E7.5_lat.jpg

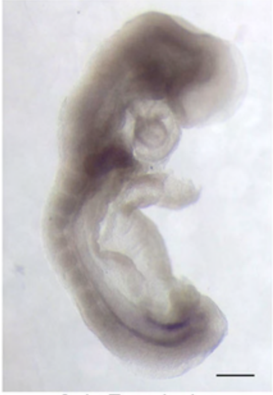
Galt_E8.5_lat.jpg

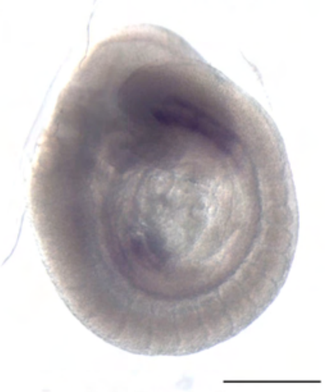
Galt_E9.0_lat.jpg

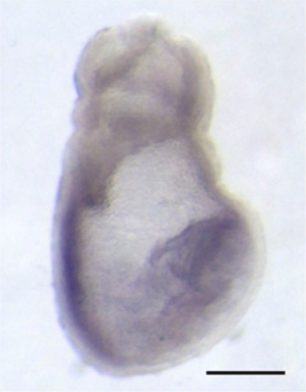
Gas5_E7.5_lat.jpg

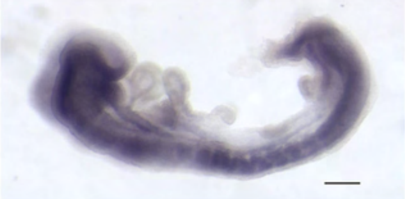
Gas5_E8.5_lat.jpg

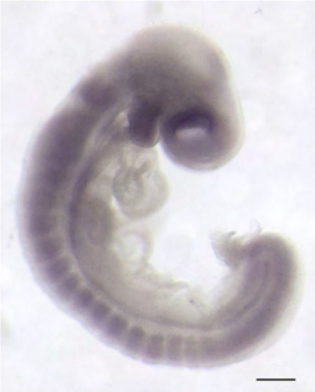
Gas5_E9.0_lat.jpg

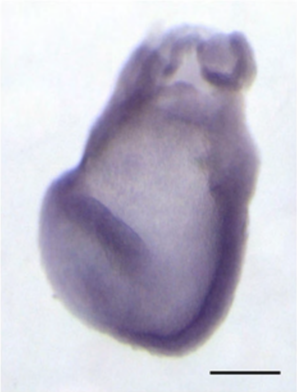
Gna14_E7.5_lat.jpg

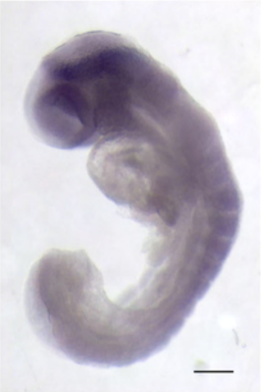
Gna14_E8.5_lat.jpg

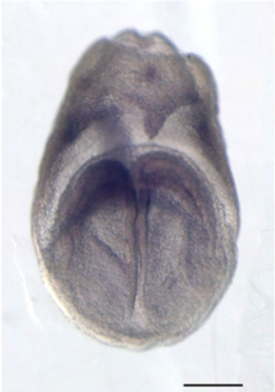

Gpx2_E7.75_ant.jpg

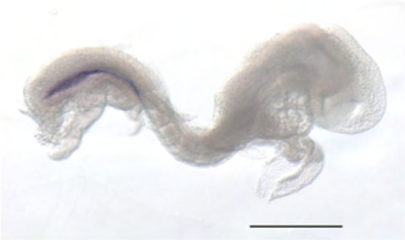

Gpx2_E8.5_lat.jpg

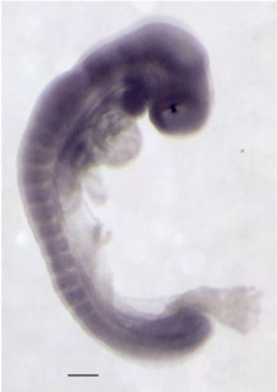

Gpx2_E9.0_lat.jpg

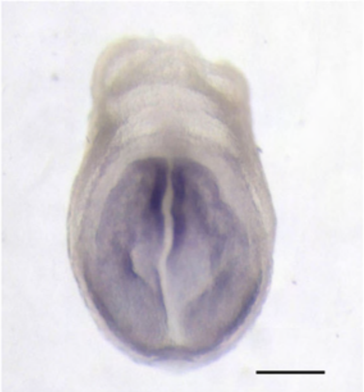

Gsta4_E7.5_ant.jpg

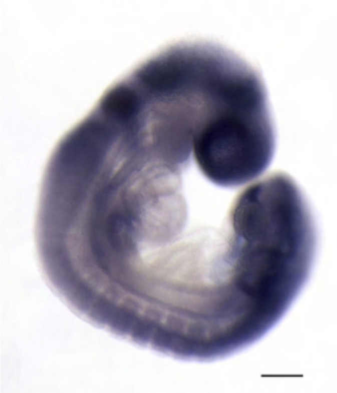

Gsta4_E8.5_lat.jpg

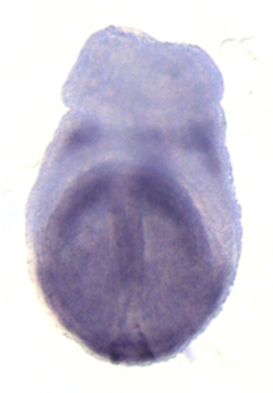

Gstm5_E7.75_ant.jpg

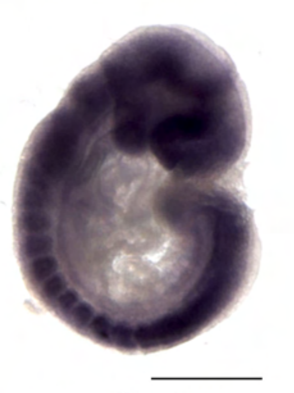

Gstm5_E8.5_lat.jpg

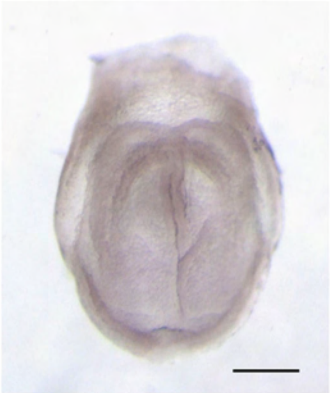

H13_E7.5_ant.jpg

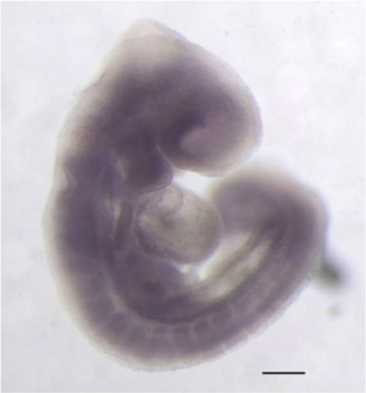

H13_E8.5_lat.jpg

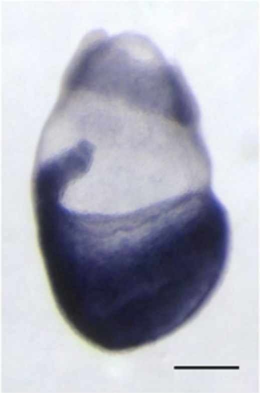

H1fx_E7.5_lat.jpg

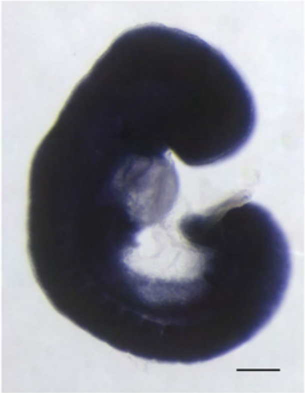

H1fx_E8.5_lat.jpg

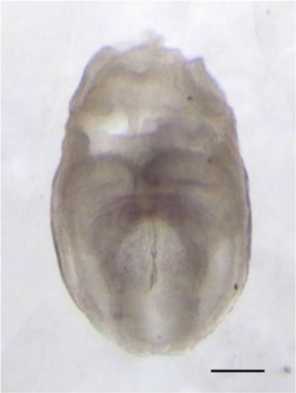

H2-K1_E7.75_ant.jpg

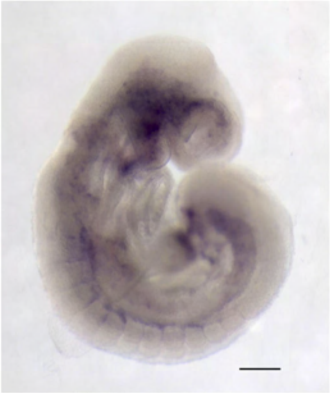

H2-K1_E8.5_lat.jpg

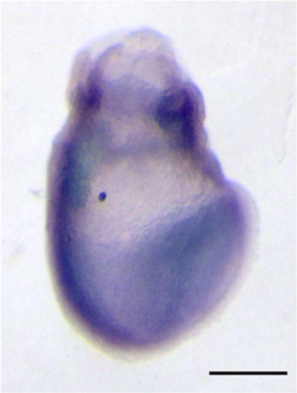

Hrmt1l2_E7.5_lat.jpg

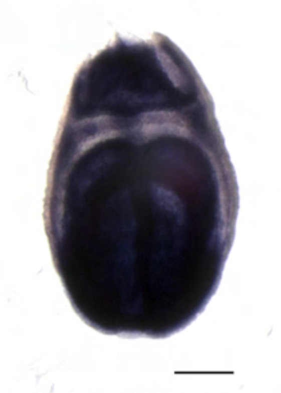

Hrmt1l2_E7.75_ant.jpg

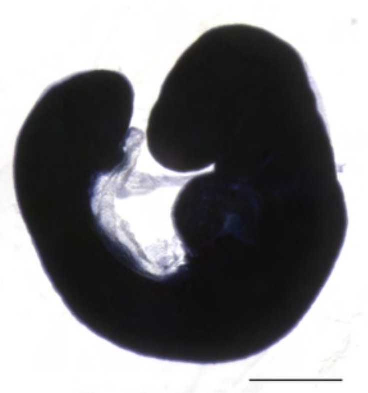

Hrmt1l2_E9.0_lat.jpg

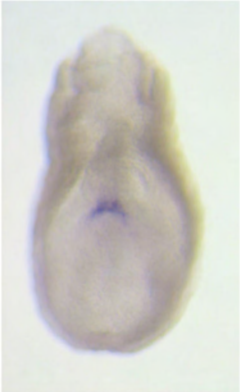

Igfbp3_E7.5_post.jpg

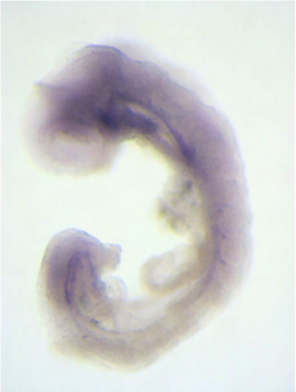

Igfbp3_E8.5_lat.jpg

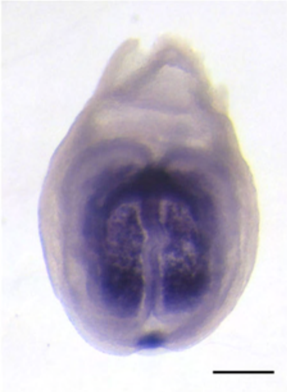

Igfbp5_E7.75_ant.jpg

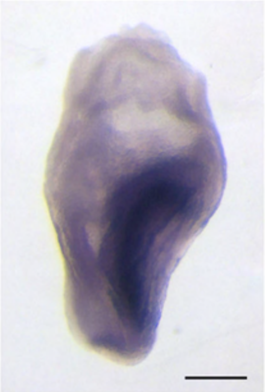

Igfbp5_E7.75_lat.jpg

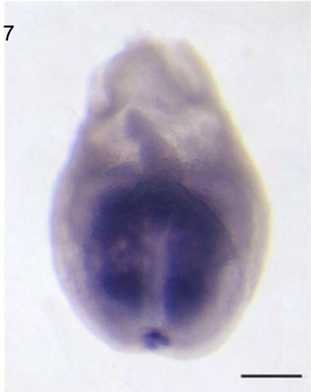
Igfbp5_E7.75_post.jpg

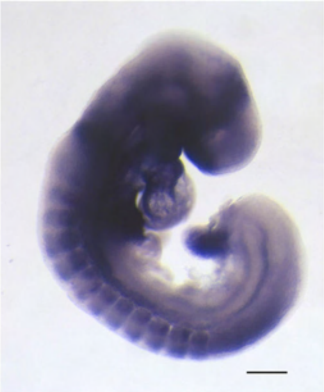
Igfbp5_E8.5_lat.jpg

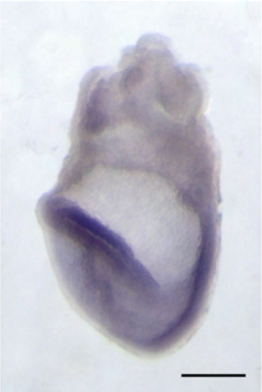
Igsf4a_E7.5_lat.jpg

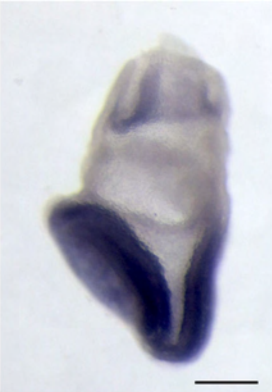
Igsf4a_E7.75_lat.jpg

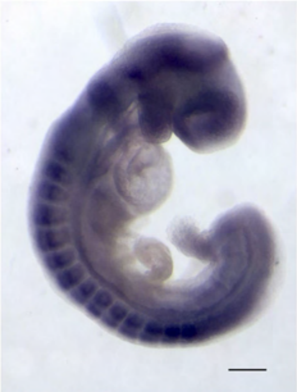
Igsf4a_E8.5_lat.jpg

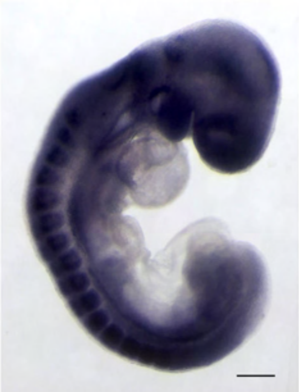
Igsf4a_E9.0_lat.jpg

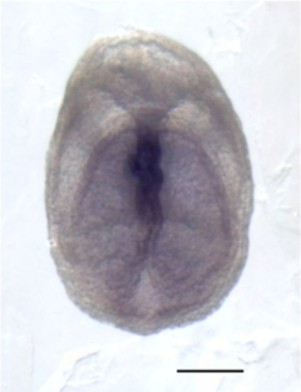
Ina_E7.5_ant.jpg

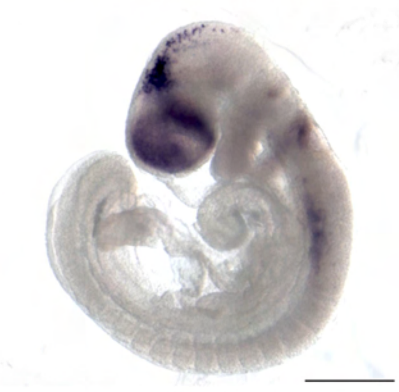
Ina_E9.0_lat.jpg

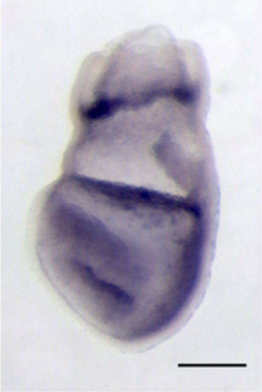
Inpp1_E7.5_lat.jpg

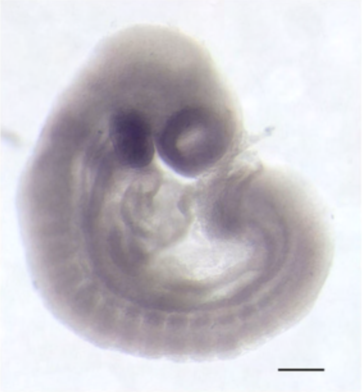
Inpp1_E8.5_lat.jpg

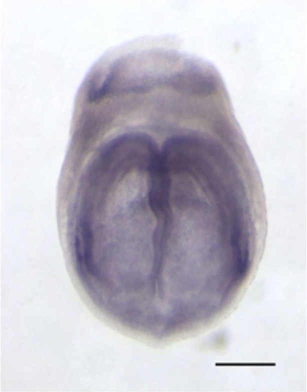
Itga3_E7.75_ant.jpg

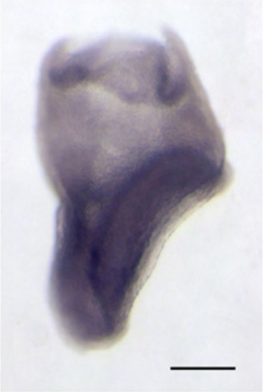
Itga3_E7.75_lat.jpg

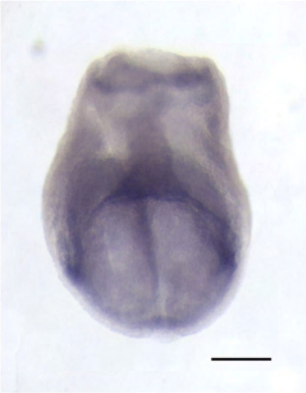
Itga3_E7.75_post.jpg

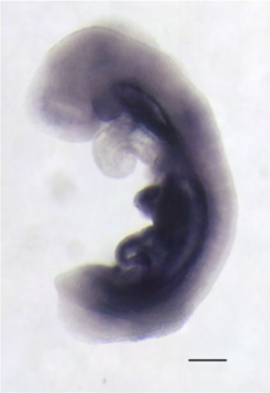
Itga3_E8.5_lat.jpg

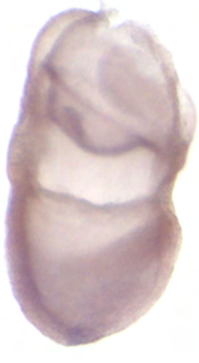
Josd2_E7.5_lat.jpg

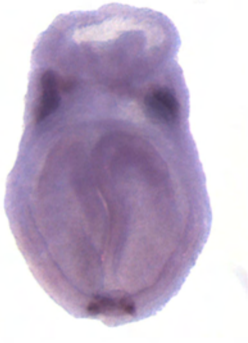
Josd2_E7.75_ant.jpg

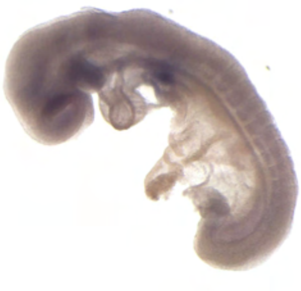
Josd2_E8.5_lat.jpg

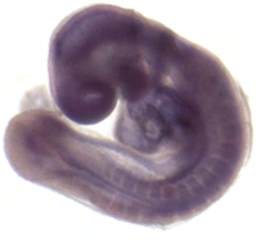
Josd2_E9.0_lat.jpg

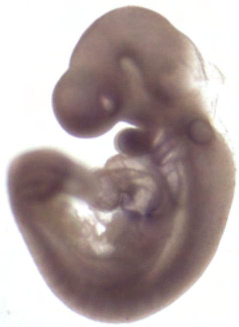
Josd2_E9.5_lat.jpg

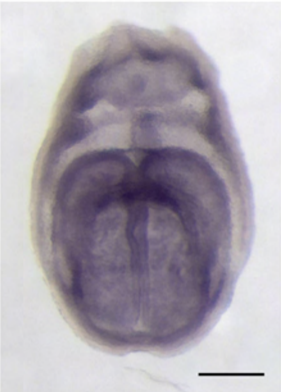
Kctd12_E7.75_ant.jpg

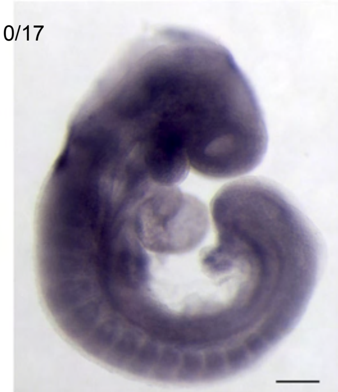
Kctd12_E8.5_lat.jpg

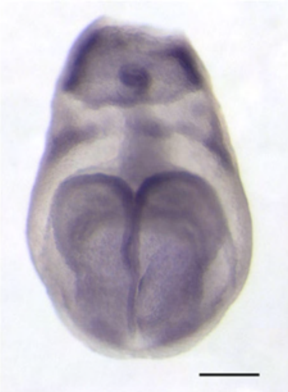
Kitl_E7.75_ant.jpg

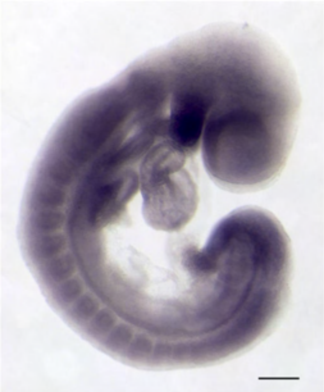
Kitl_E8.5_lat.jpg

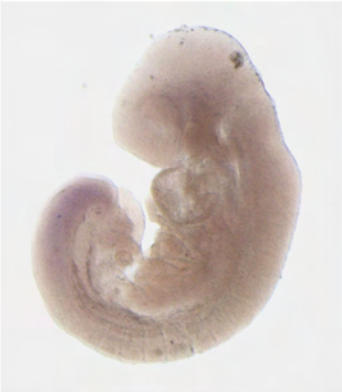
Limch1_E9.0_lat.jpg

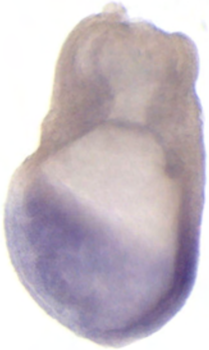
Lrig3_E7.5_lat.jpg

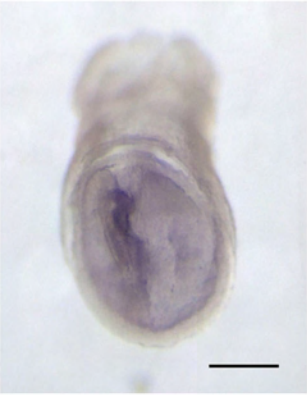
Lrrc54_E7.5_ant.jpg

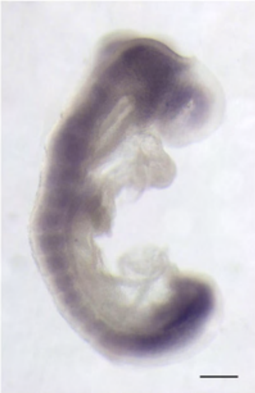
Lrrc54_E8.5_lat.jpg

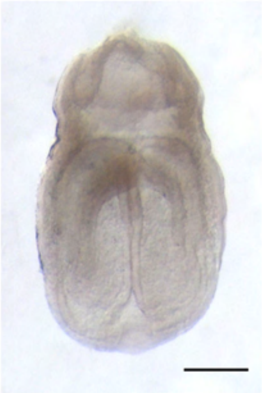
Mapre1_E7.75_ant.jpg

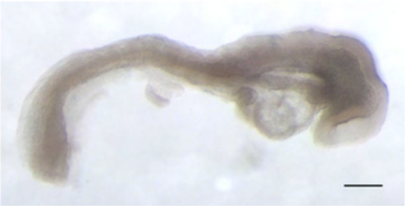
Mapre1_E8.5_lat.jpg

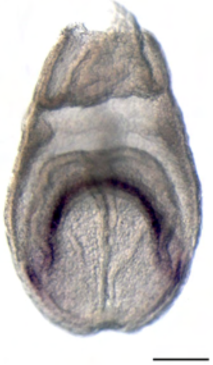
Meis1_E7.75_ant.jpg

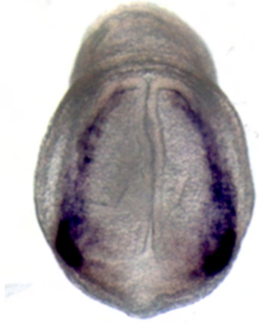
Meis1_E7.75_ant_b.jpg

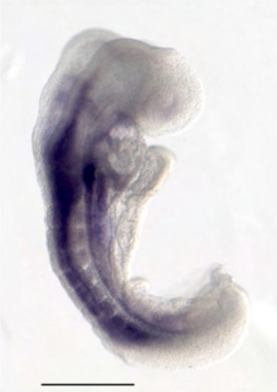
Meis1_E8.5_lat.jpg

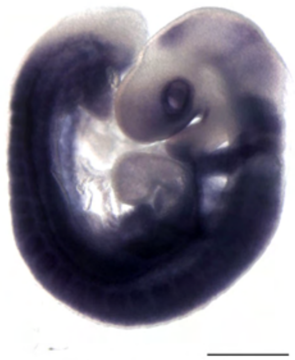
Meis1_E9.0_lat.jpg

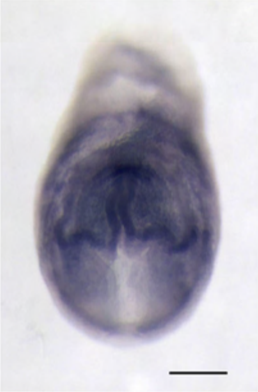
Mest_E7.75_ant.jpg

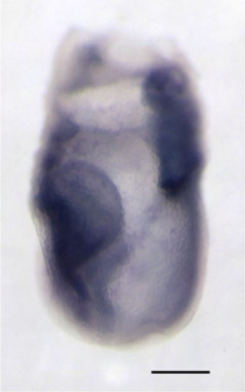
Mest_E7.75_lat.jpg

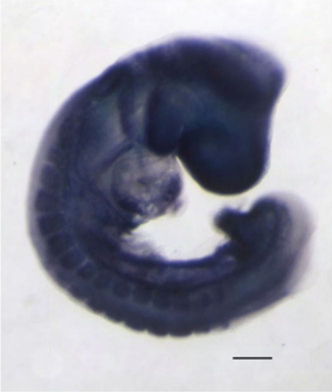
Mest_E8.5_lat.jpg

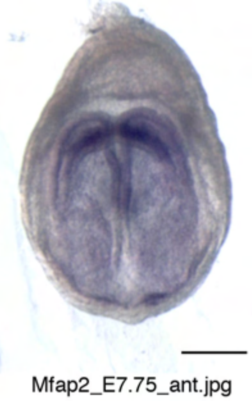
Mfap2_E7.75_ant.jpg

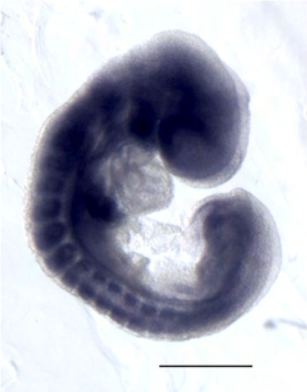
Mfap2_E8.5_lat.jpg

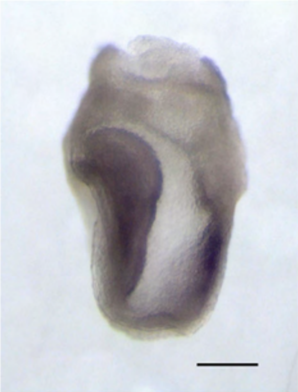
Mgst1_E7.75_lat.jpg

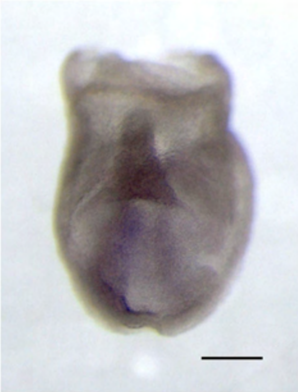
Mgst1_E7.75_post.jpg

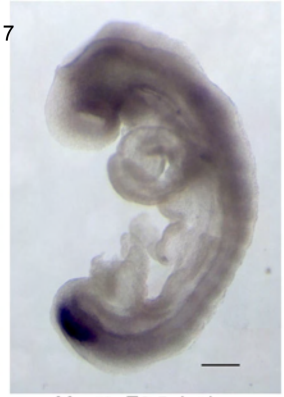
Mgst1_E8.5_lat.jpg

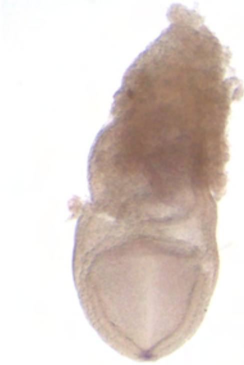
Mlf1_E7.5_ant.jpg

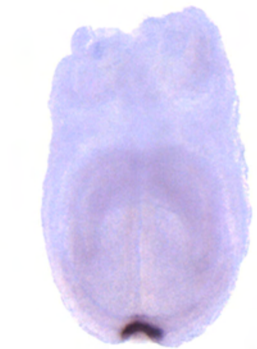
Mlf1_E7.75_ant.jpg

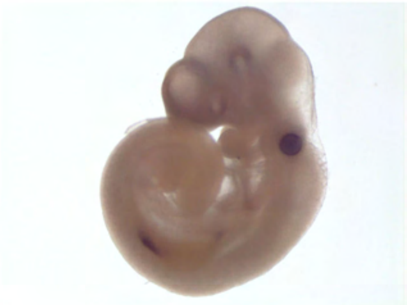
Mlf1_E9.5_lat.jpg

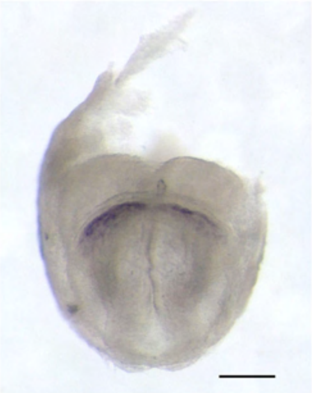
Myl1_E7.75_ant.jpg

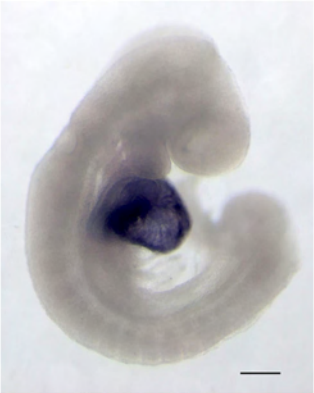
Myl1_E9.0_lat.jpg

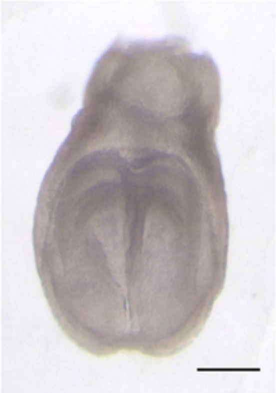
Myl4_E7.5_ant.jpg

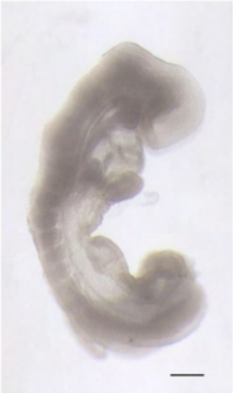
Myl4_E8.5_lat.jpg

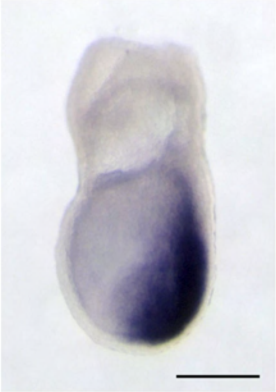
Nkx1-2_E7.5_lat.jpg

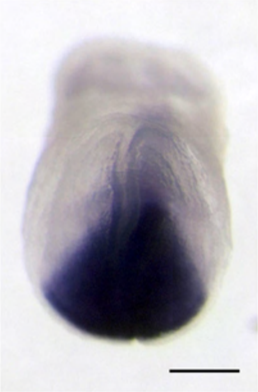
Nkx1-2_E7.75_ant.jpg

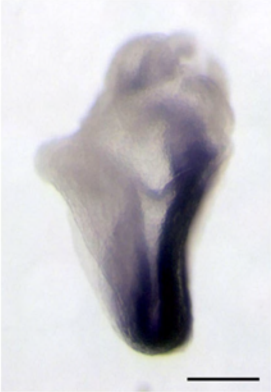
Nkx1-2_E7.75_lat.jpg

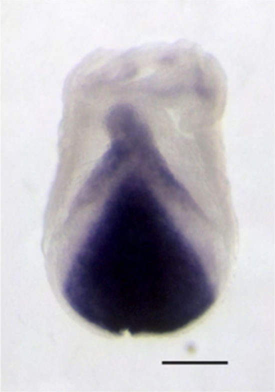
Nkx1-2_E7.75_post.jpg

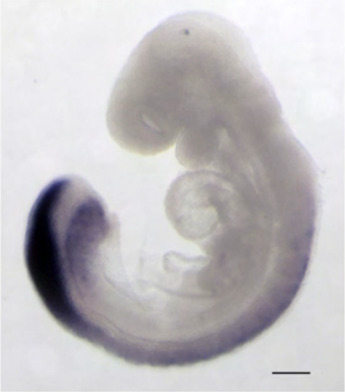
Nkx1-2_E9.0_lat.jpg

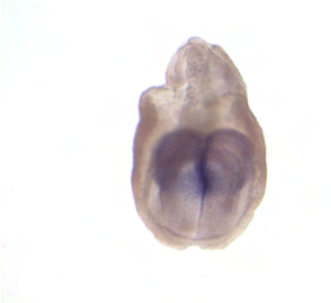
Nptx2_E7.75_ant.jpg

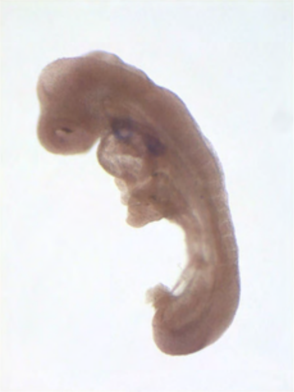
Nptx2_E8.5_lat.jpg

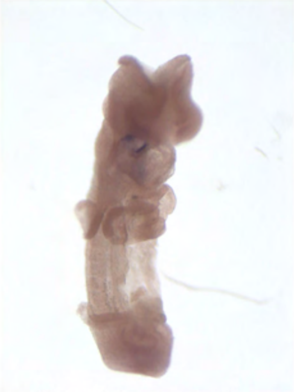
Nptx2_E8.5_ventral.jpg

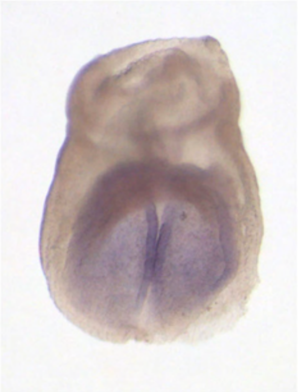
Pcsk1n_E7.75_ant.jpg

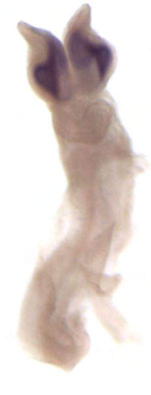
Pcsk1n_E8.5_ventral.jpg

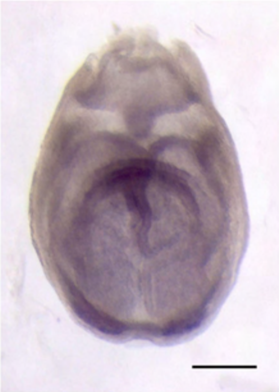
Pcsk5_E7.75_ant.jpg

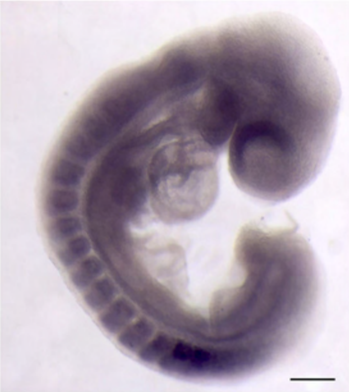
Pcsk5_E8.5_lat.jpg

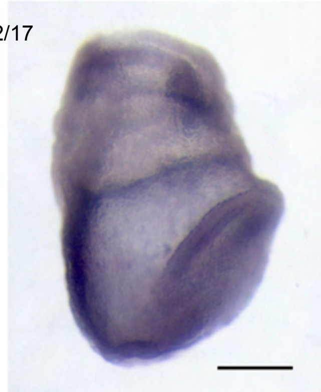
Pdia4_E7.5_lat.jpg

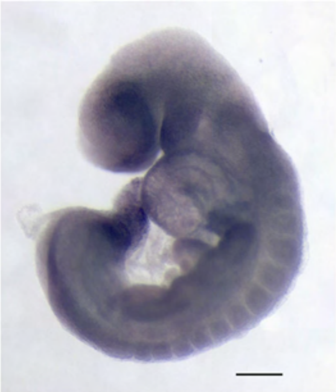
Pdia4_E8.5_lat.jpg

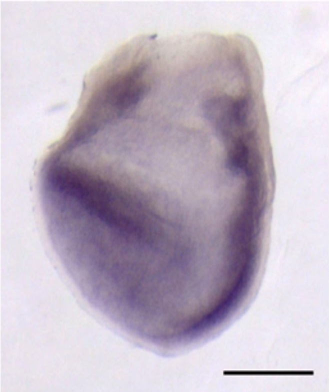
Pgm2_E7.5_lat.jpg

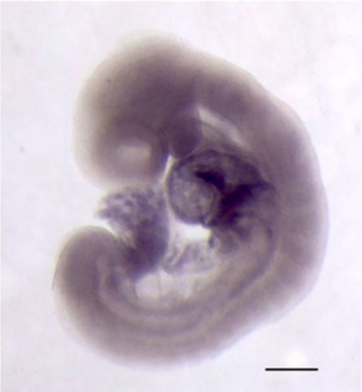
Pgm2_E8.5_lat.jpg

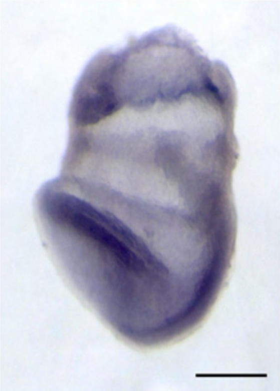
Phldb1_E7.5_lat.jpg

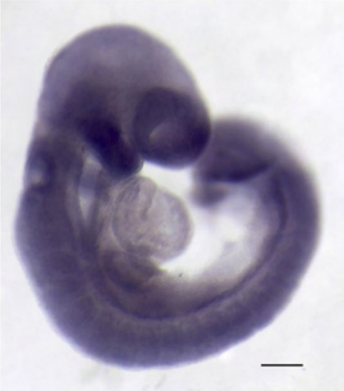
Phldb1_E9.0_lat.jpg

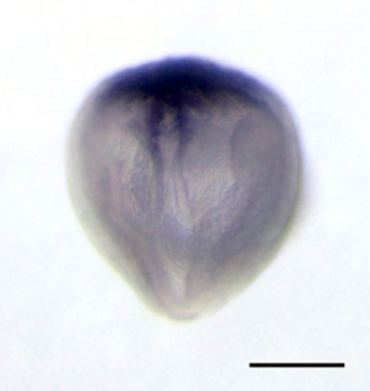
Pim1_E7.5_distal.jpg

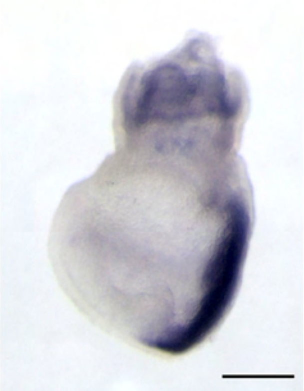
Pim1_E7.5_lat.jpg

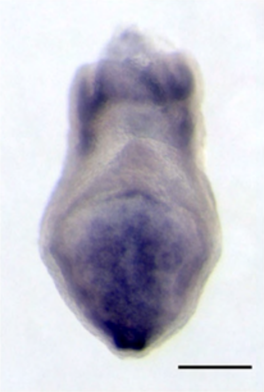
Pim1_E7.5_post.jpg

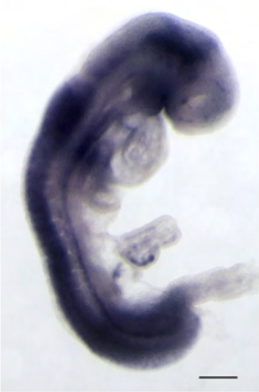
Pim1_E8.5_lat.jpg

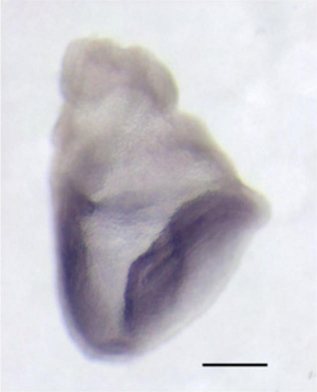
Pkd2_E7.5_lat.jpg

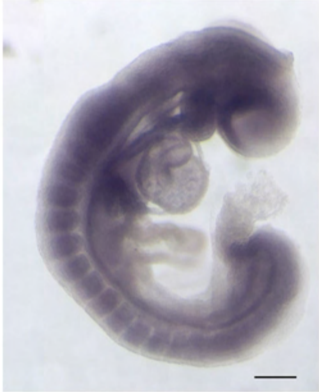
Pkd2_E8.5_lat.jpg

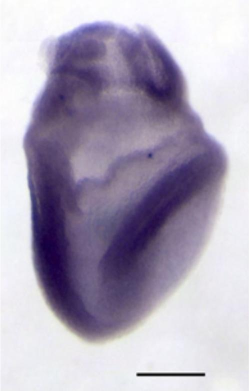
Pkig_E7.5_lat.jpg

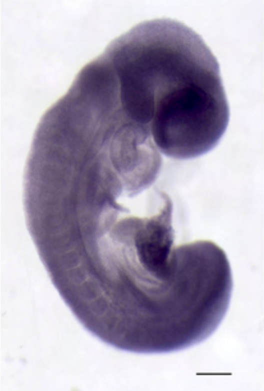
Pkig_E8.5_lat.jpg

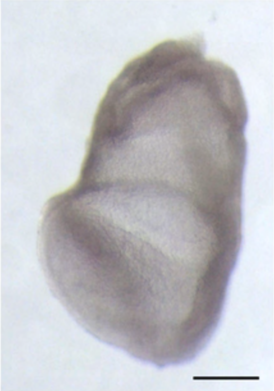
Pla2g7_E7.5_lat.jpg

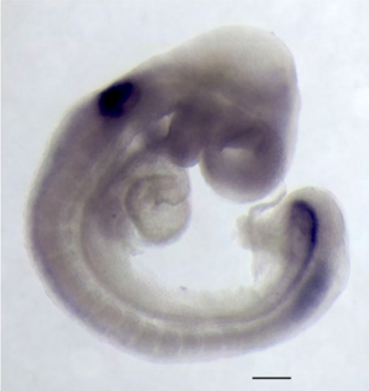
Pla2g7_E9.0_lat.jpg

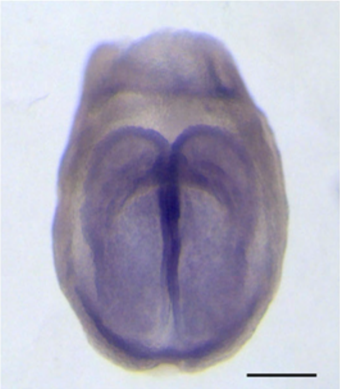
Plekha1_E7.5_ant.jpg

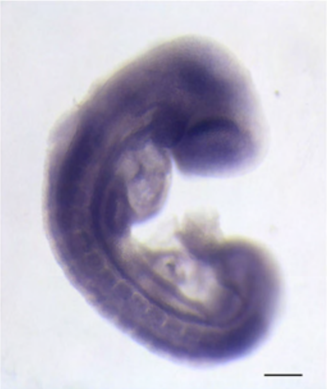
Plekha1_E8.5_lat.jpg

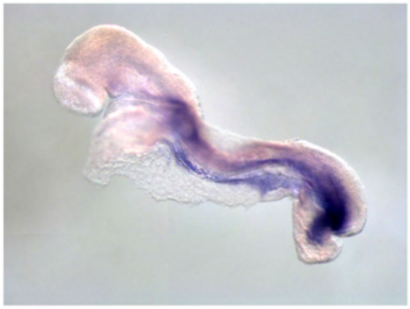
Ppp1r14a_E8.5_lat_a.jpg

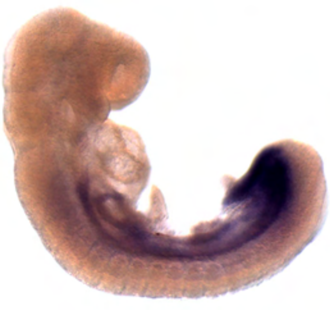
Ppp1r14a_E8.5_lat_c.jpg

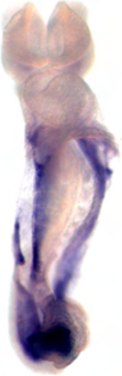
Ppp1r14a_E8.5_ventral_a.jpg

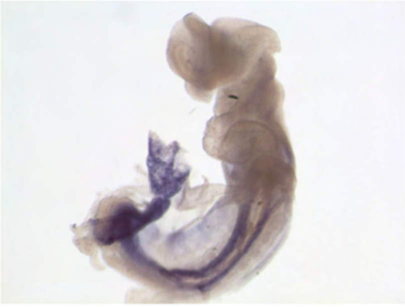
Ppp1r14a_E8.5_ventral_b.jpg

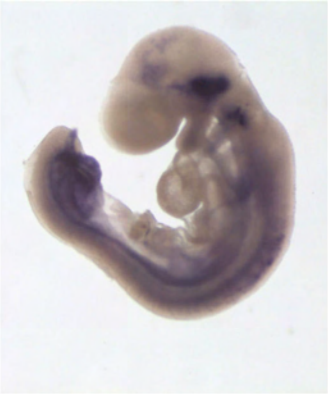
Ppp1r14a_E9.0_lat.jpg

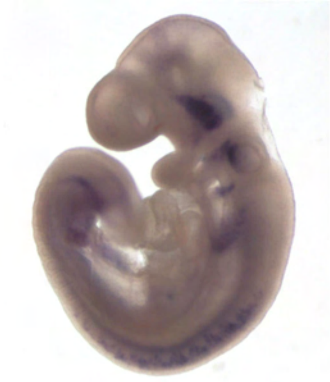
Ppp1r14a_E9.5_lat.jpg

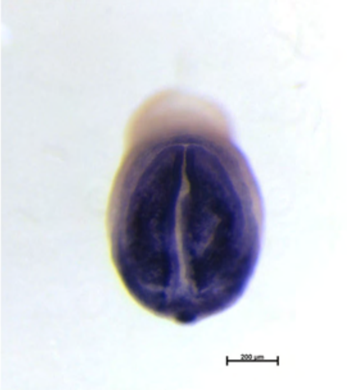
Ppp1r1a_E7.75_ant.jpg

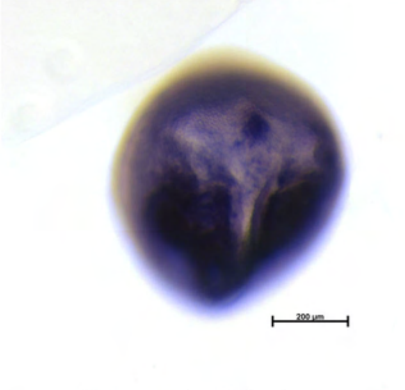
Ppp1r1a_E7.75_distal.jpg

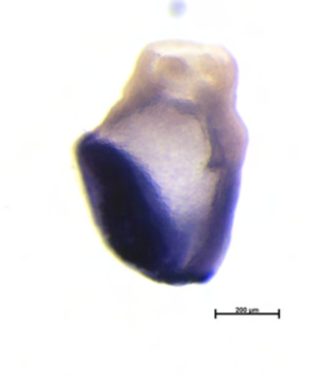
Ppp1r1a_E7.75_lat.jpg

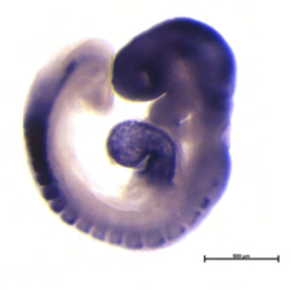
Ppp1r1a_E9.0_lat.jpg

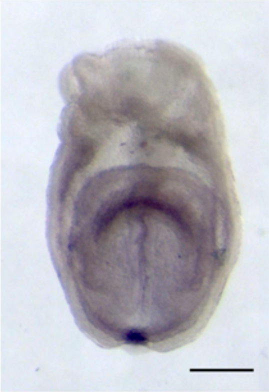
Prnp_E7.75_ant.jpg

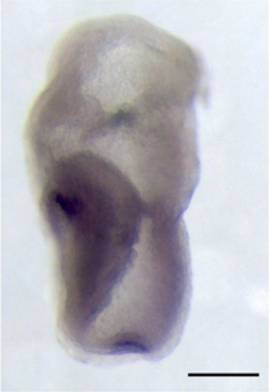
Prnp_E7.75_lat.jpg

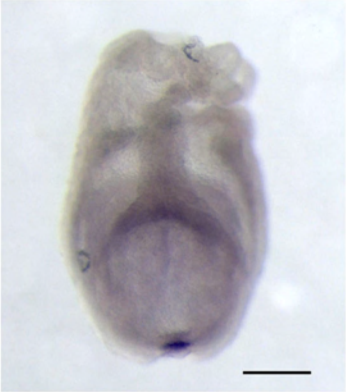
Prnp_E7.75_post.jpg

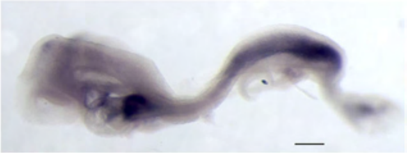
Prnp_E8.5_lat.jpg

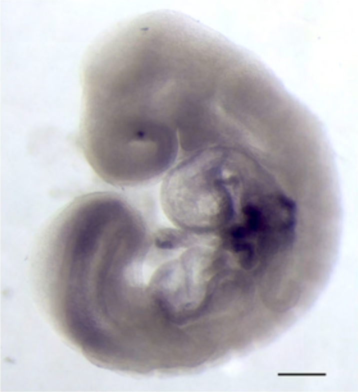
Prnp_E9.0_lat.jpg

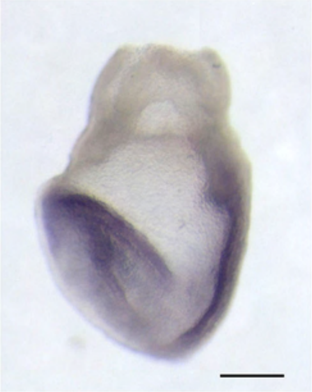
Prss19_E7.5_lat.jpg

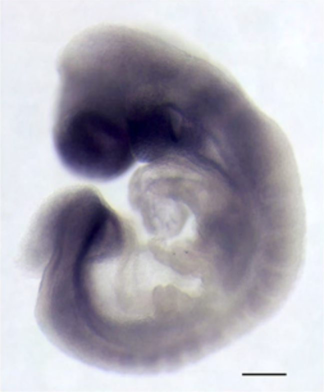
Prss19_E8.5_lat.jpg

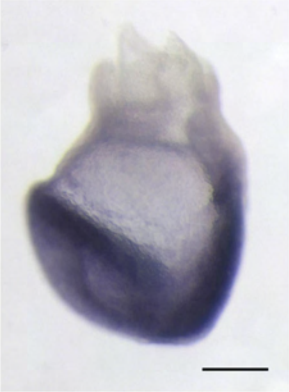
Ptgis_E7.5_lat.jpg

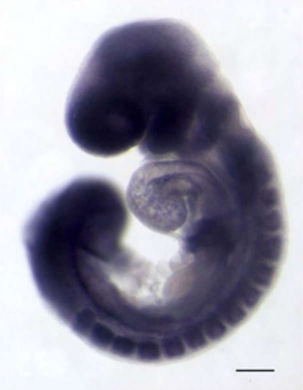
Ptgis_E9.0_lat.jpg

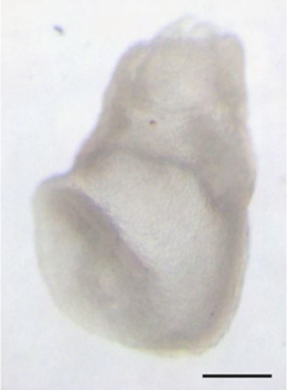
Ptprd_E7.5_lat.jpg

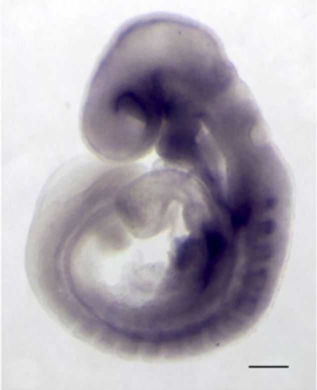
Ptprd_E9.0_lat.jpg

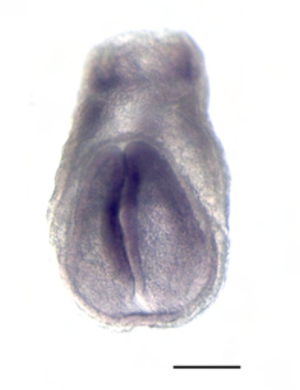
Pts_E7.75_ant.jpg

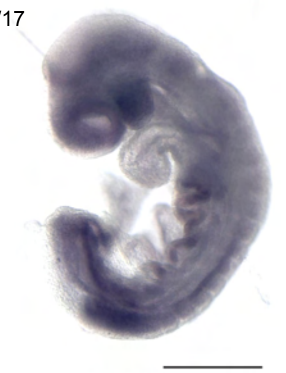
Pts_E8.5_lat.jpg

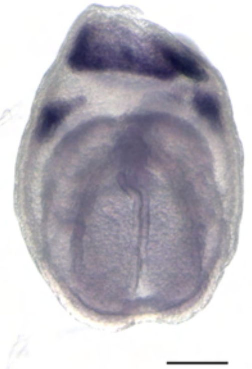
Pura_E7.75_ant.jpg

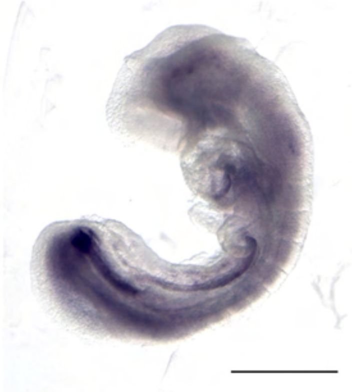
Pura_E8.5_lat.jpg

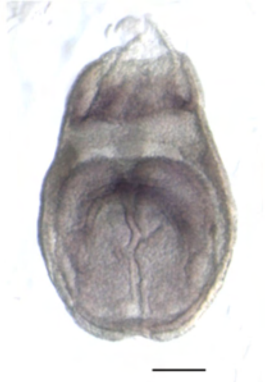
Pygb_E7.75_ant.jpg

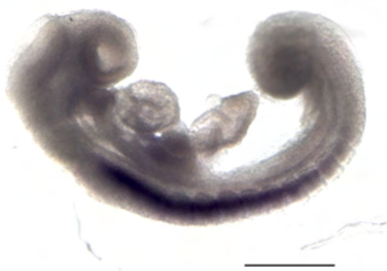
Pygb_E8.5_lat.jpg

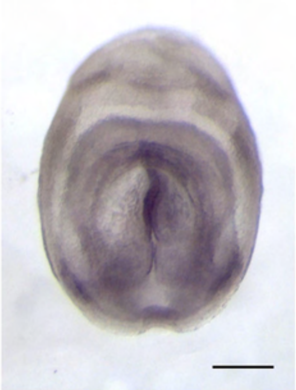
Raet1e_E7.75_ant.jpg

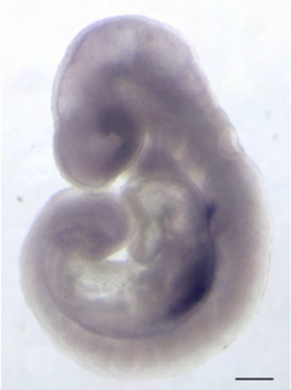
Raet1e_E9.0_lat.jpg

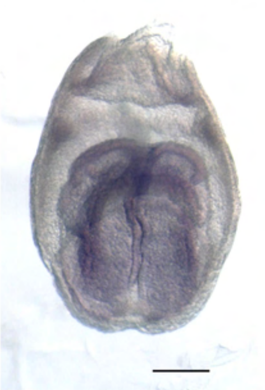
Rap2b_E7.75_ant.jpg

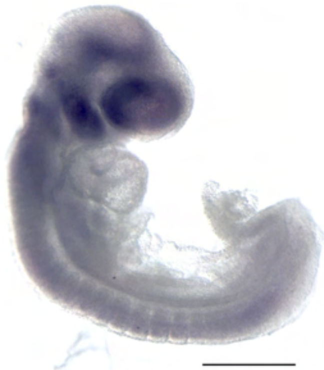
Rap2b_E8.5_lat.jpg

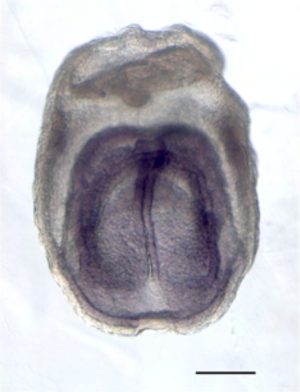
Rbmx_E7.75_ant.jpg

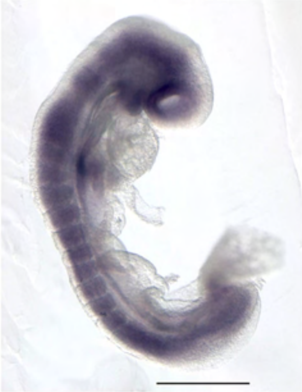
Rbmx_E8.5_lat.jpg

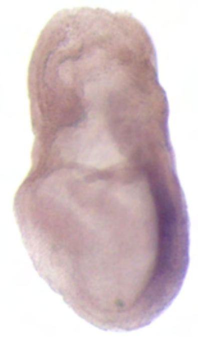
Ripk3_E7.5_lat.jpg

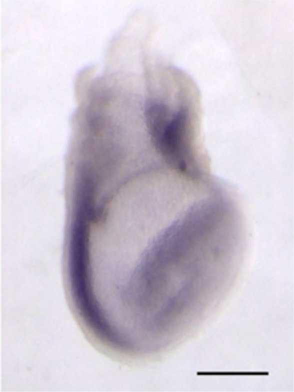
Rnaset2_E7.5_lat.jpg

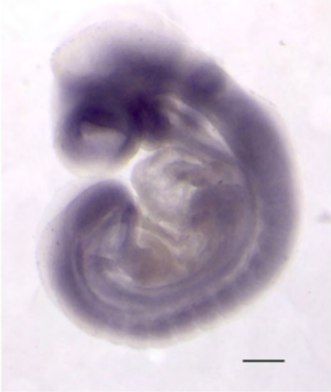
Rnaset2_E8.5_lat.jpg

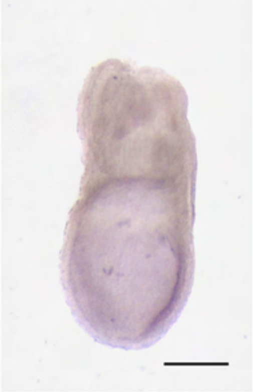
Rpl7l1_E7.5_lat.jpg

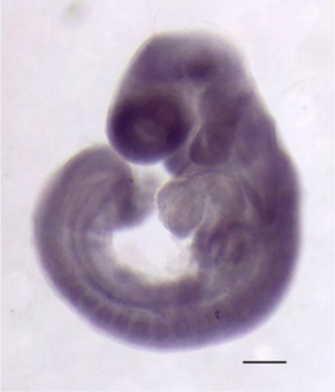
Rpl7l1_E9.0_lat.jpg

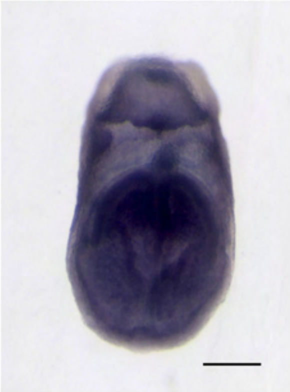
Rshl2_E7.5_ant.jpg

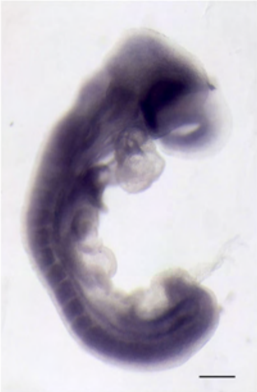
Rshl2_E8.5_lat.jpg

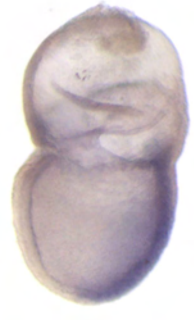
Rspo3_E7.5_lat.jpg

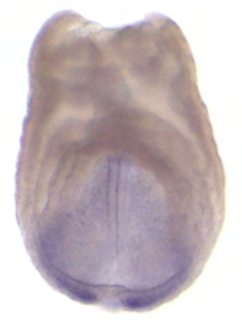
Rspo3_E7.75_ant.jpg

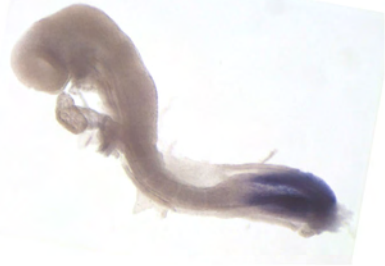

Rspo3_E8.5_lat.jpg

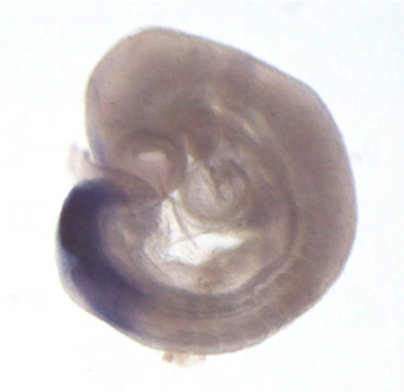

Rspo3_E9.0_lat.jpg

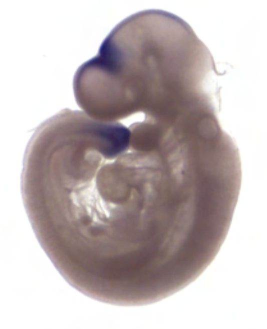

Rspo3_E9.5_lat.jpg

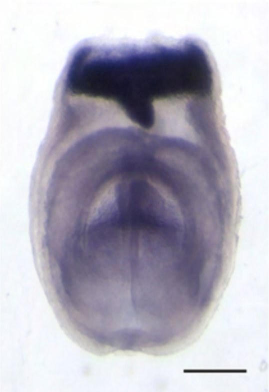

S100a11_E7.75_ant.jpg

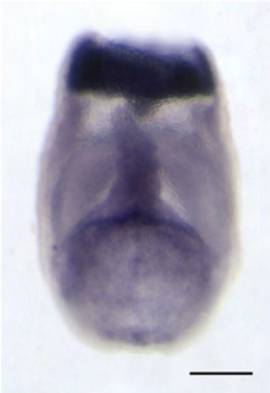

S100a11_E7.75_post.jpg

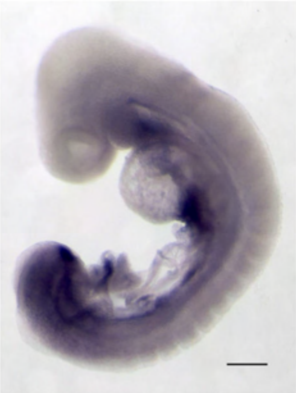

S100a11_E8.5_lat.jpg

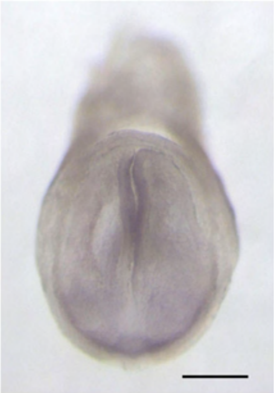

Scn1a_E7.5_ant.jpg

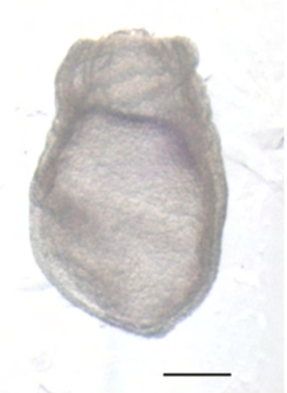

Scn1a_E7.5_lat.jpg

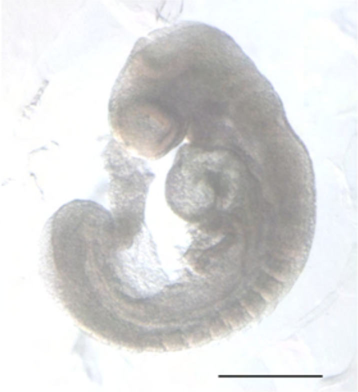

Scn1a_E8.5_lat.jpg

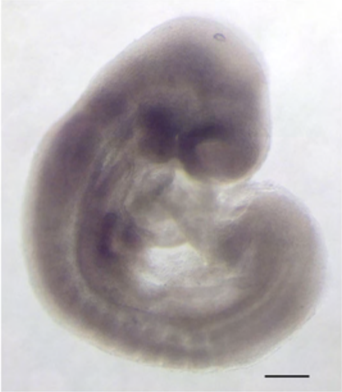

Scn1a_E9.0_lat.jpg

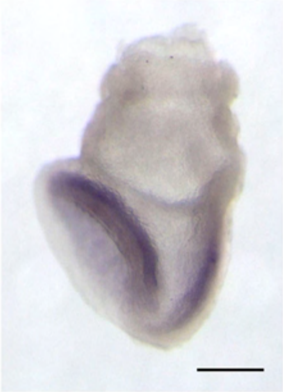

Sgne1_E7.5_lat.jpg

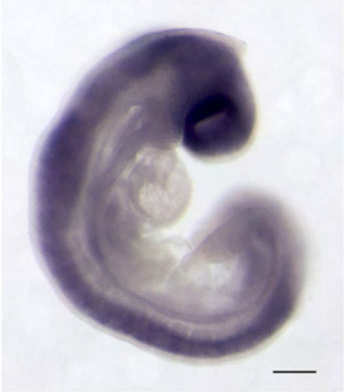

Sgne1_E8.5_lat.jpg

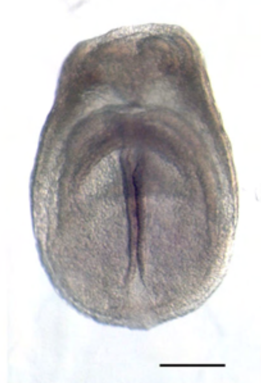

Slc1a5_E7.75_ant.jpg

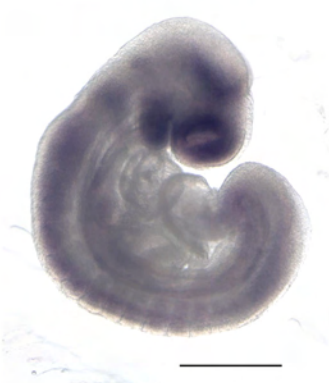

Slc1a5_E8.5_lat.jpg

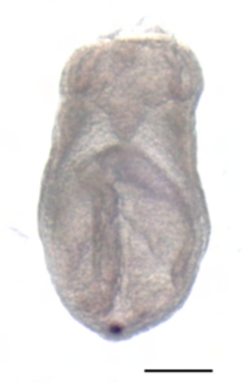

Smoc1_E7.5_ant.jpg

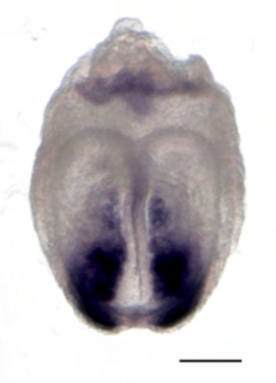

Smoc1_E7.75_ant.jpg

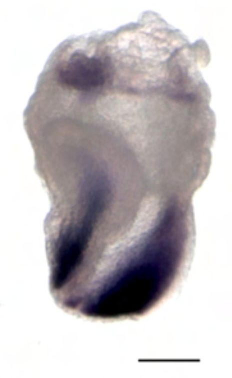

Smoc1_E7.75_lat.jpg

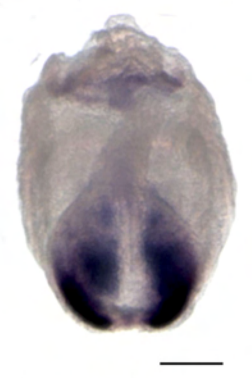

Smoc1_E7.75_post.jpg

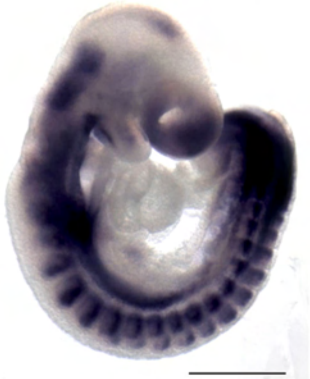

Smoc1_E9.0_lat.jpg

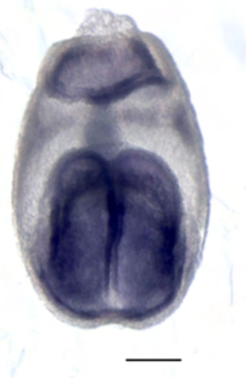

Snx5_E7.75_ant.jpg

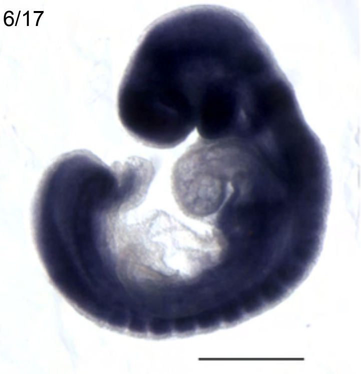
Snx5_E8.5_lat.jpg

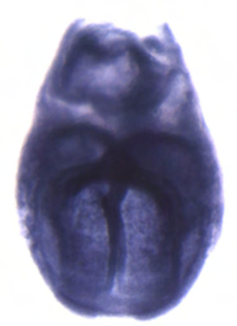
Sparc_E7.75_ant.jpg

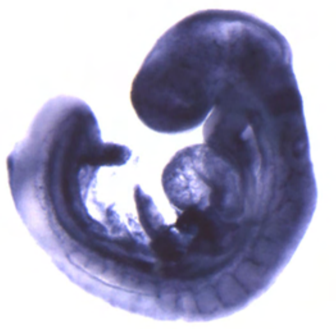
Sparc_E9.0_lat.jpg

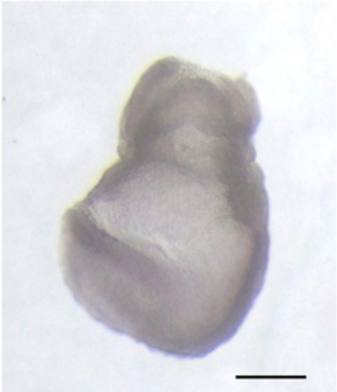
Stt3a_E7.5_ant.jpg

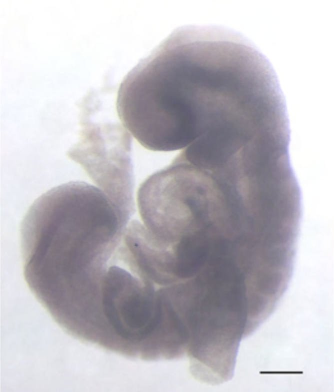
Stt3a_E8.5_lat.jpg

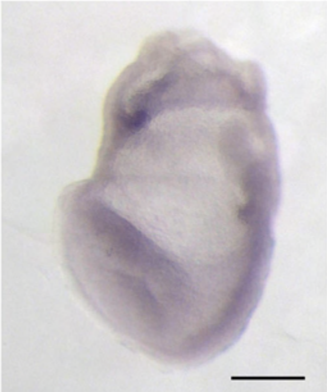
Tgds_E7.5_lat.jpg

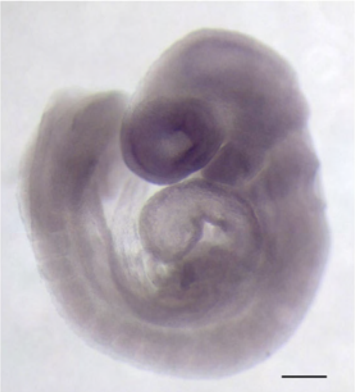
Tgds_E8.5_lat.jpg

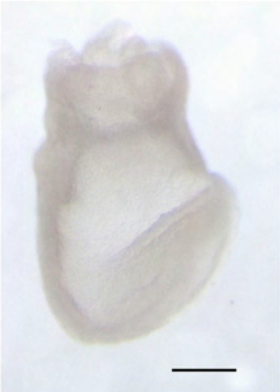
Tmem68_E7.5_lat.jpg

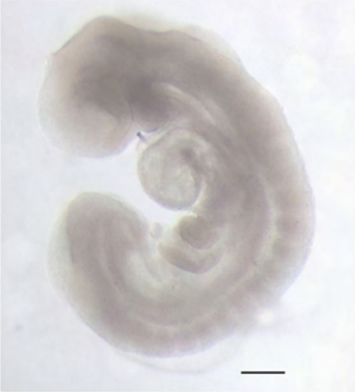
Tmem68_E8.5_lat.jpg

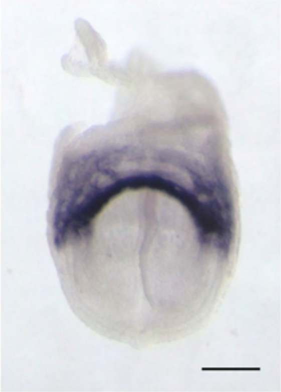
Tnnt2_E7.75_ant.jpg

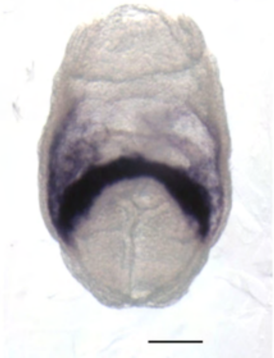
Tnnt2_E7.75_ant_b.jpg

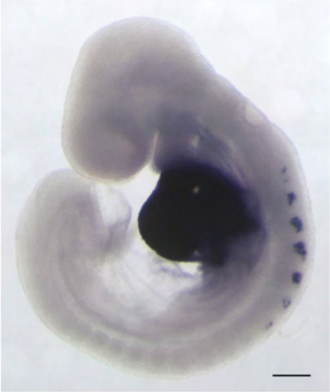
Tnnt2_E9.0_lat.jpg

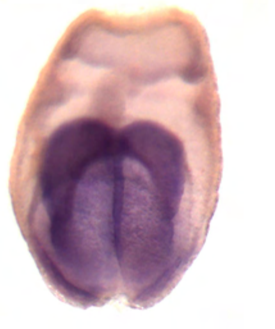
Tpbg_7.75_ant.jpg

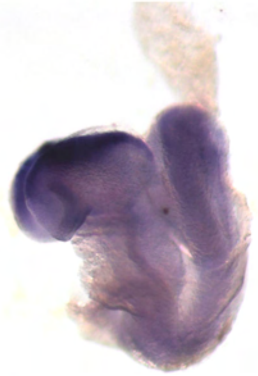
Tpbg_8.5_lat.jpg

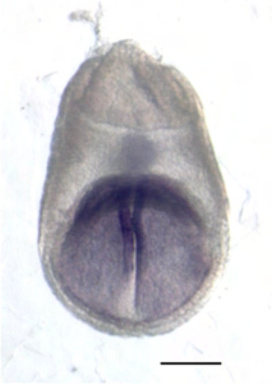
Tpbg_E7.5_ant.jpg

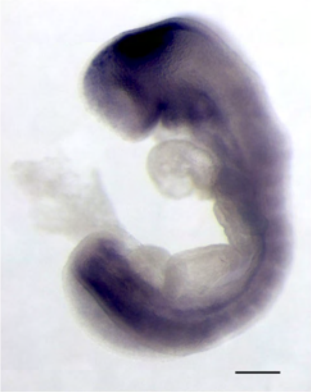
Tpbg_E8.5_lat.jpg

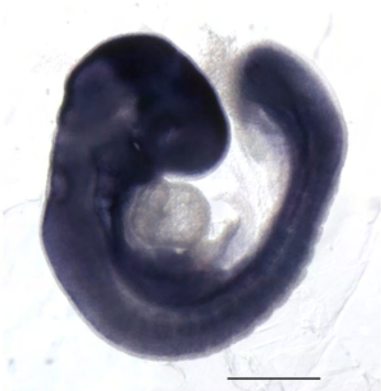
Tpbg_E9.0_lat.jpg

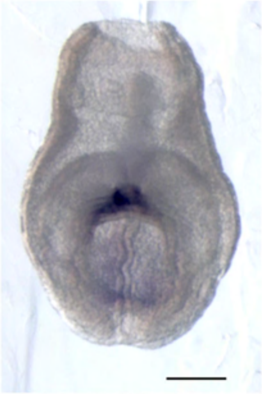
Trh_E7.75_ant.jpg

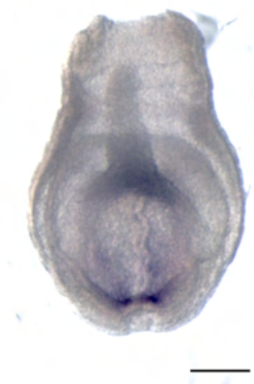
Trh_E7.75_post.jpg

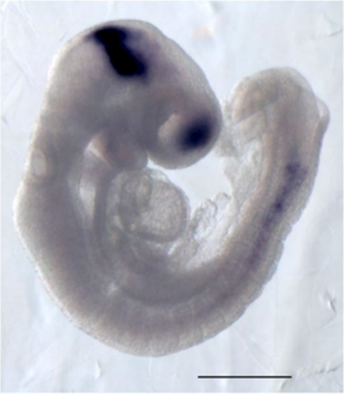
Trh_E9.0_lat.jpg

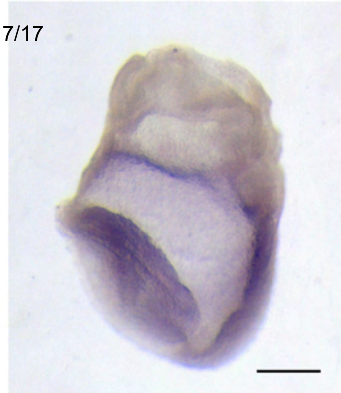
Trove2_E7.5_lat.jpg

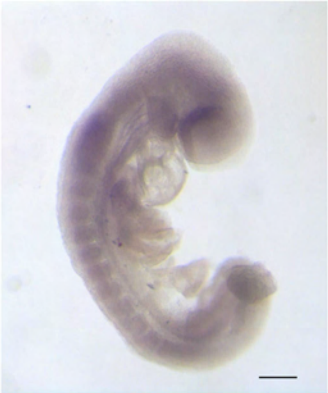
Trove2_E8.5_lat.jpg

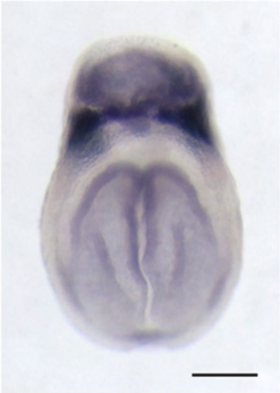
Tspan33_E7.5_ant.jpg

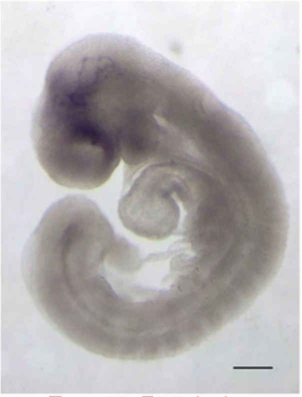
Tspan33_E8.5_lat.jpg

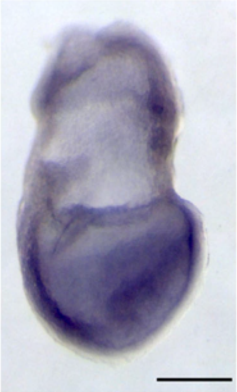
Tubb4_E7.5_lat.jpg

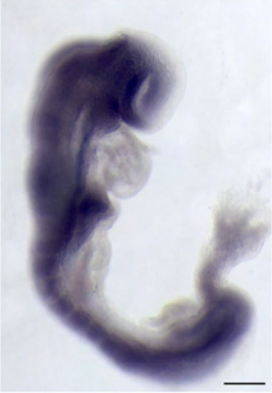
Tubb4_E8.5_lat.jpg

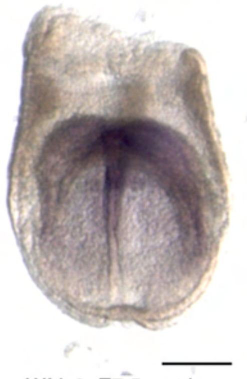
Wfdc2_E7.5_ant.jpg

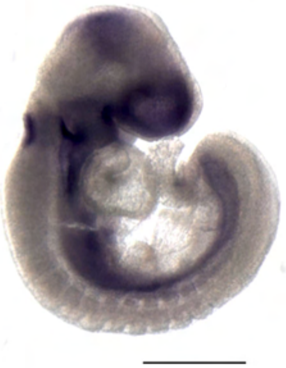
Wfdc2_E8.5_lat.jpg

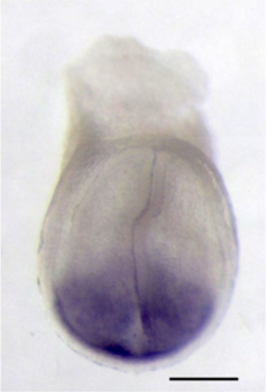
Wnt8a_E7.5_ant.jpg

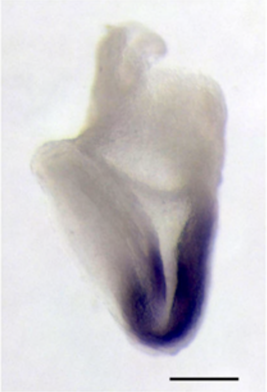
Wnt8a_E7.5_lat.jpg

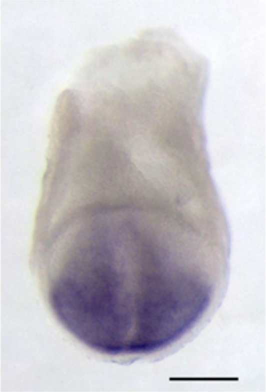
Wnt8a_E7.5_post.jpg

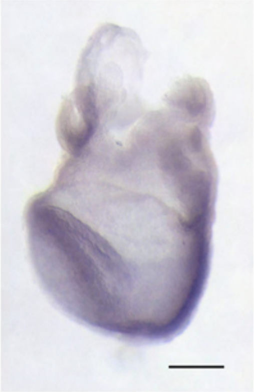
Zranb1_E7.5_lat.jpg

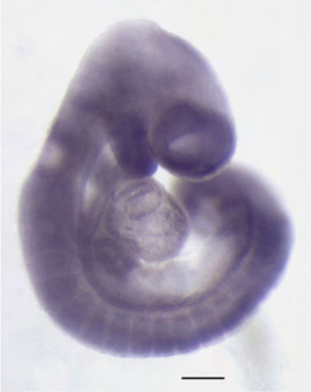
Zranb1_E8.5_lat.jpg
