# Supplementary material for: Microarray analysis of Foxa2 mutant mouse embryos reveals novel gene expression and inductive roles for the gastrula organizer and its derivatives
Source: BMC Genomics. 2008 Oct 30;9:511. doi: 10.1186/1471-2164-9-511 (PMC2605479; doi:10.1186/1471-2164-9-511)
Supplement: Additional file 5 — Analysis of Foxa expression data by Heiko Lickert. Report and details of Affymetrix MOE430v2 GeneChip data analysis. [file 1471-2164-9-511-S5.pdf]

Analysis of *Foxa* expression data by Heiko Lickert

Michael T. Mader
m.mader@gsf.de
GSF Research Center, Institute of Stem Cell Research,
Ingolstädter Landstr. 1, 85764 Neuherberg, Germany

April 11, 2005

Contents

1 Preprocessing **2**

2 Quality and Reproducability Control **3**

3 Gene-wise testing **16**
3.1 Wilcoxon paired rank tests on chip designs separately (single probes) . . 16
3.2 MG-U74 17
3.3 MOE4302 66
3.4 Testing on the combined dataset (probesets) 103

4 Mapping MG-U74 data on MOE4302 data **104**

5 Conclusions **104**

All analytical procedures and algorithms are implemented in R-code (Ihaka and Gentleman, 1996). The necessary R-packages are available from http://cran.r-project.org and http://mips.gsf.de/mips/staff/mader/software. If you have a local installation of R and BioConductor you may want to use vExplorer ore openVignette to work through this Sweave-based vignette.

The experiment consists of four extracts

- two from Foxa mutant embryos E7.5
- two from wt embryos (E7.5)

Hybridizations were performed twice. The first time in 2002/2003 (sample-dependent) on MG-U74 A Affymetrix GeneChips and the second time in March 2004 (one day for all four extracts) on MOE4302 Affymetrix GeneChips.

The experiment is a classical two-group comparison (mutant vs. wildtype) with no additional parameters varied. However, an additional goal of the study is to confirm results on a rather early GeneChip platform with recent GeneChips.

1 Preprocessing

The following files were used for this analysis:

Filename	Hybridization Date	Platform	Genotype
U74A2_021903_HL03T_LH.CEL	20.02.2003	MG-U74	wt
U74A2_021903_HL04T_LH.CEL	20.02.2003	MG-U74	Foxa2
U74A2_102402_HW01T_LH.CEL	14.10.2002	MG-U74	wt
U74A2_102402_HW02T_LH.CEL	14.10.2002	MG-U74	Foxa2
ISF_HL_A21.CEL	16.03.2005	MOE4302	Foxa2
ISF_HL_A22.CEL	16.03.2005	MOE4302	Foxa2
ISF_HL_wt1.CEL	16.03.2005	MOE4302	wt
ISF_HL_wt2.CEL	16.03.2005	MOE4302	wt

Table 1: Files and annotations used for the analysis

No filtering (including ceiling/flooring) was applied so far.

Preprocessing steps:

- Calculation of probeset summaries (according to the three most popular algorithms (MAS 5.0, dChip (Li and Wong, 2001), RMA (Bolstad et al., 2003))
- Log-scale Transformation (log, basis 2)
- Normalization (lmp; nonlinear transformation employing the loess smoother (Cleveland et al., 1992), (Cleveland, 1979), (Cleveland, 1981))

The preprocessing was performed separately for the MG-U74 and MOE4302 chips. The table headings in the following sections are comprised of:

- hybridization date
- separating dot
- genotype

2 Quality and Reproducability Control

Variance and center measures are in tables 2 and 3.

Scatterplot matrices of the raw and normalized log-transformed data of the `MG-U74` study are presented in figures 1 and 2, respectively. Fig. 3 and 4 are for the `MOE4302` hybridizations.

	20.02.2003.wt	20.02.2003.Foxa2	14.10.2002.wt	14.10.2002.Foxa2
Mean	5.47	5.47	5.47	5.47
Median	5.56	5.57	5.57	5.58
Var	6.88	6.89	6.89	6.89
SD	2.62	2.62	2.62	2.63
Altmann's FD	0.21	0.21	−0.10	0.22

Table 2: Quality measures (variation and center) of the MG-U74 data

	16.03.2005.Foxa2	16.03.2005.Foxa2	16.03.2005.wt	16.03.2005.wt
Mean	5.06	5.06	5.06	5.06
Median	4.99	5.00	5.00	4.99
Var	6.23	6.23	6.23	6.23
SD	2.50	2.50	2.50	2.50
Altmann's FD	0.24	0.21	0.27	0.18

Table 3: Quality measures (variation and center) of the MOE4302 data

Hierarchical clustering can be used to find unexpected (dis)similarities between the samples. However, every single hierarchical clustering algorithm is prone to detect local instead of the global maximum. Hence, different methods should be compared. We present two very distinct methods, namely agglomerative and divisive clustering. In the agglomerative approach the clustering starts with separate samples and step-by-step tries to fuse clusters. Contrarily, divisive clustering starts with one large cluster and splits it iteratively up until the algorithm ends with every sample in a separate cluster. Figures 5 and 6 present results on the raw data whilst figures 7 and 8 are from normalized data. The analogs for the `MOE4302` hybridizations are in fig. 9-12.

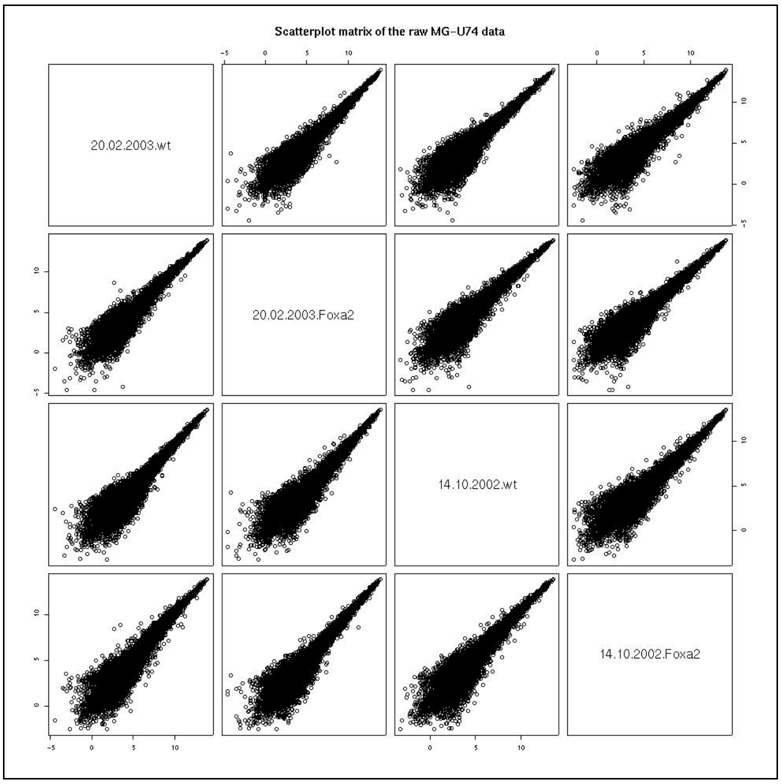

Figure 1: Scatterplot matrix of the raw and log-transformed data from MG-U74

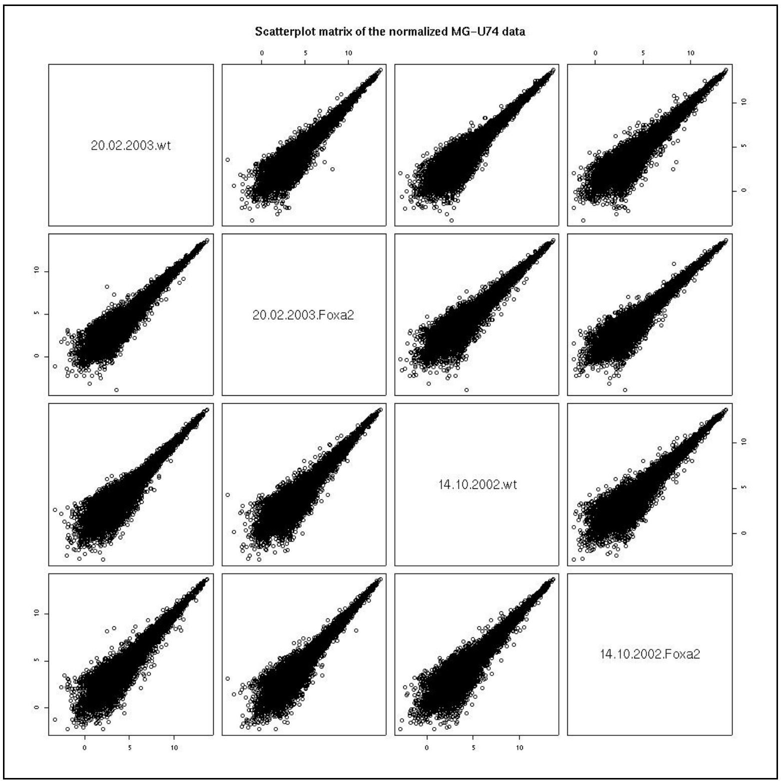

Figure 2: Scatterplot matrix of the normalized and log-transformed data from MG-U74

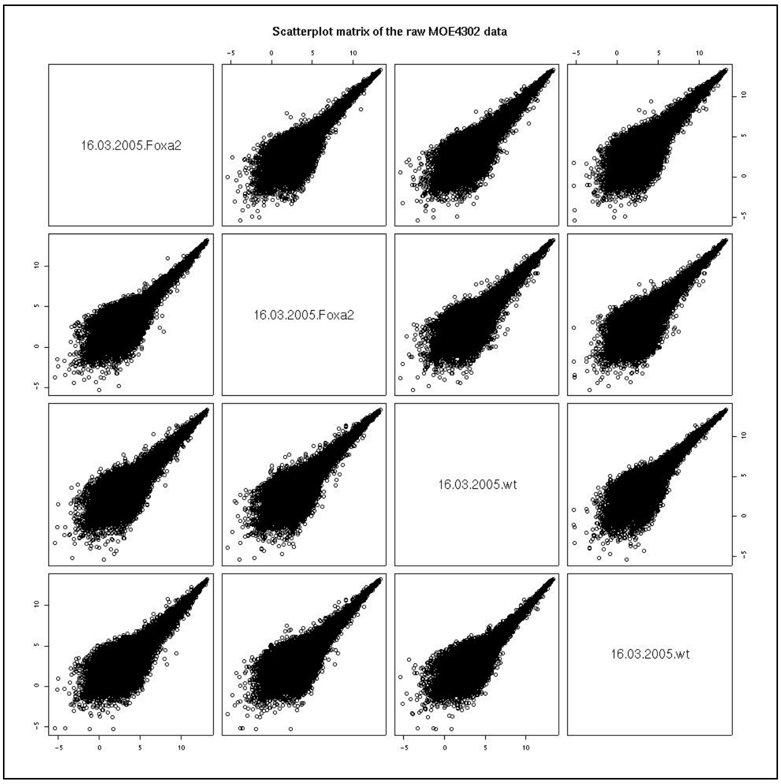

Figure 3: Scatterplot matrix of the raw and log-transformed data from MOE4302

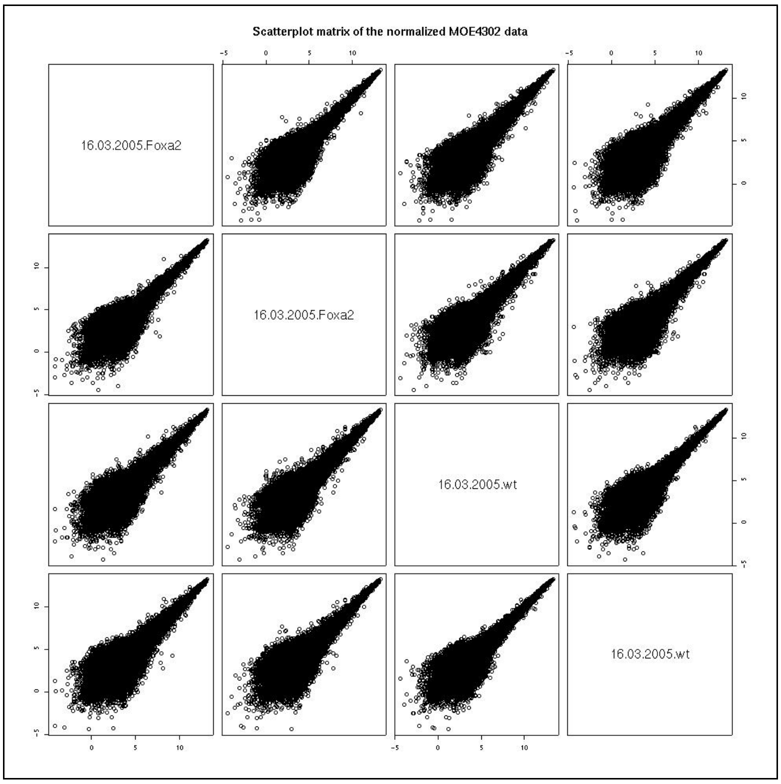

Figure 4: Scatterplot matrix of the normalized and log-transformed data from MOE4302

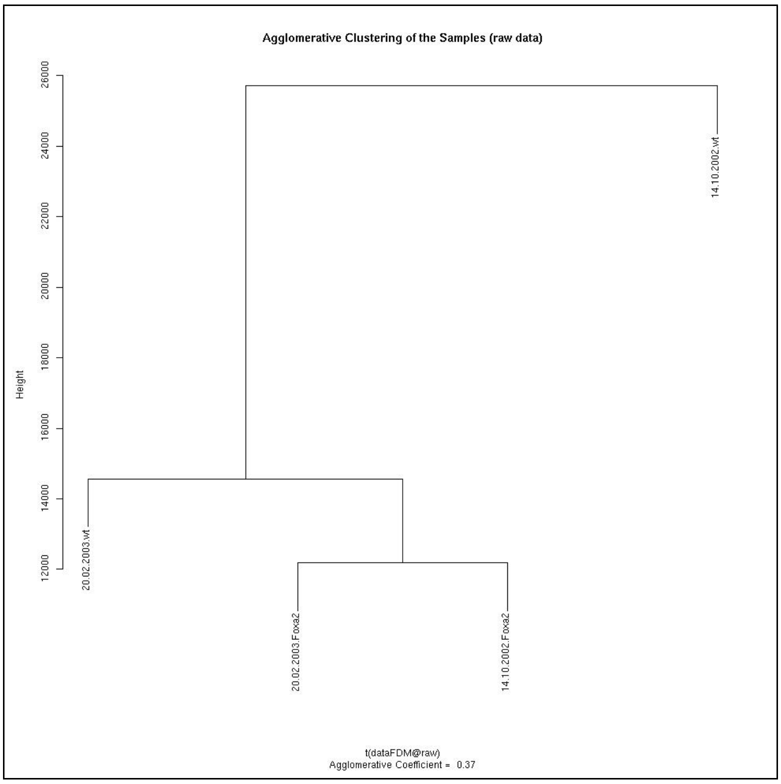

Figure 5: Agglomerative Clustering on raw log-scaled data for MG-U74

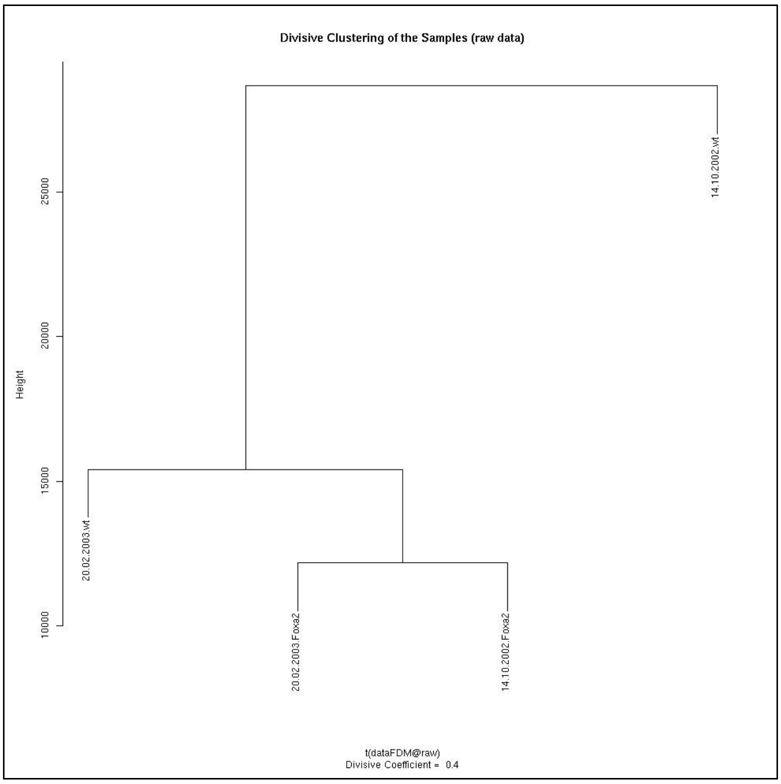

Figure 6: Diana Clustering on raw log-scaled data for MG-U74

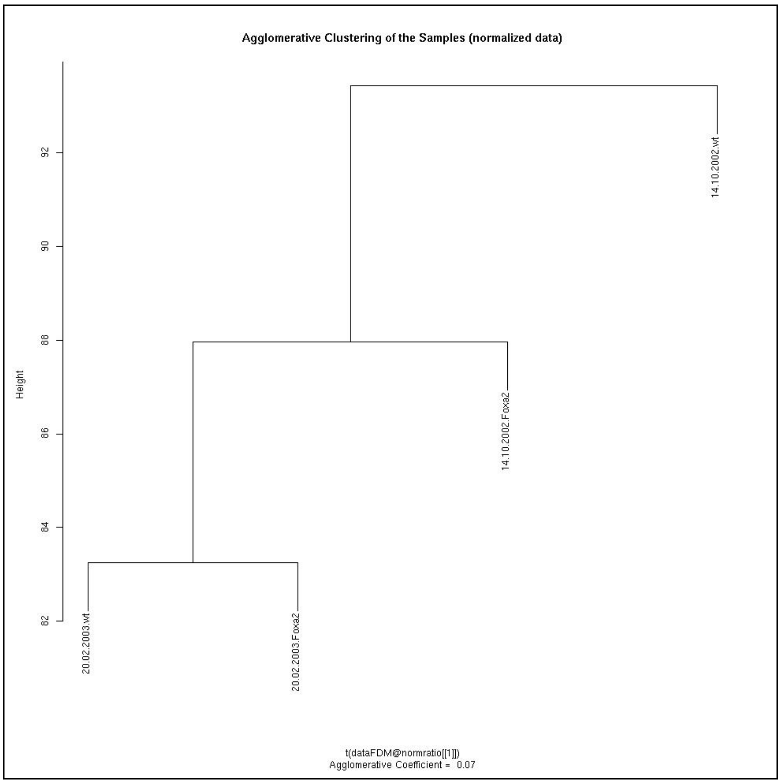

Figure 7: Agglomerative Clustering on normalized log-scaled data for MG-U74

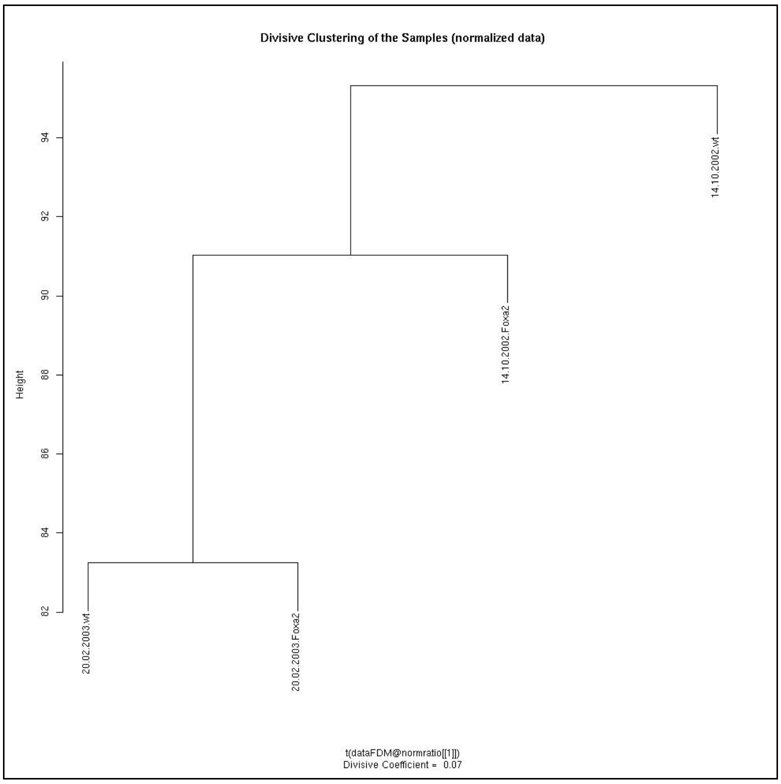

Figure 8: Diana Clustering on normalized log-scaled data for MG-U74

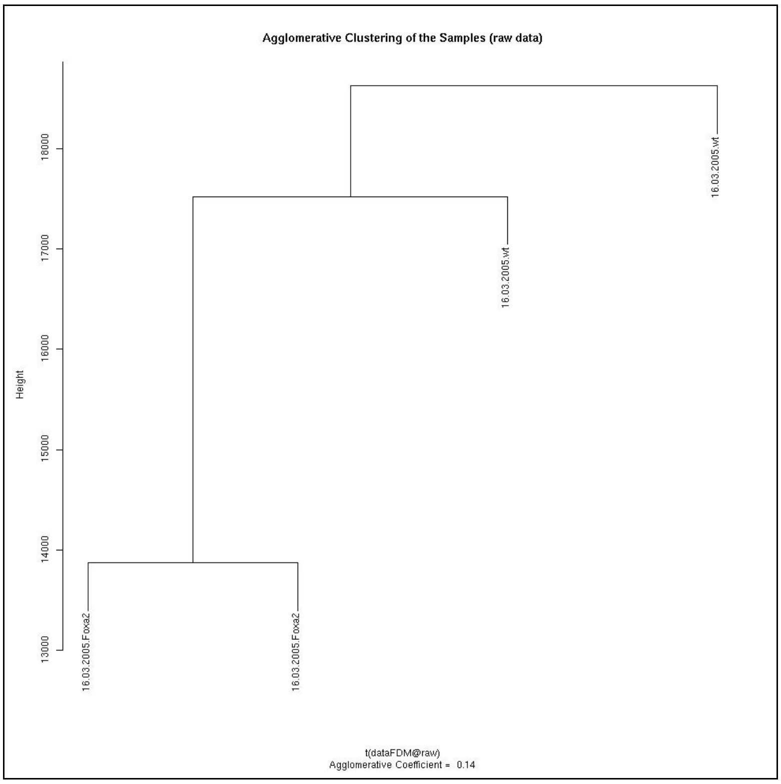

Figure 9: Agglomerative Clustering on raw log-scaled data for MOE4302

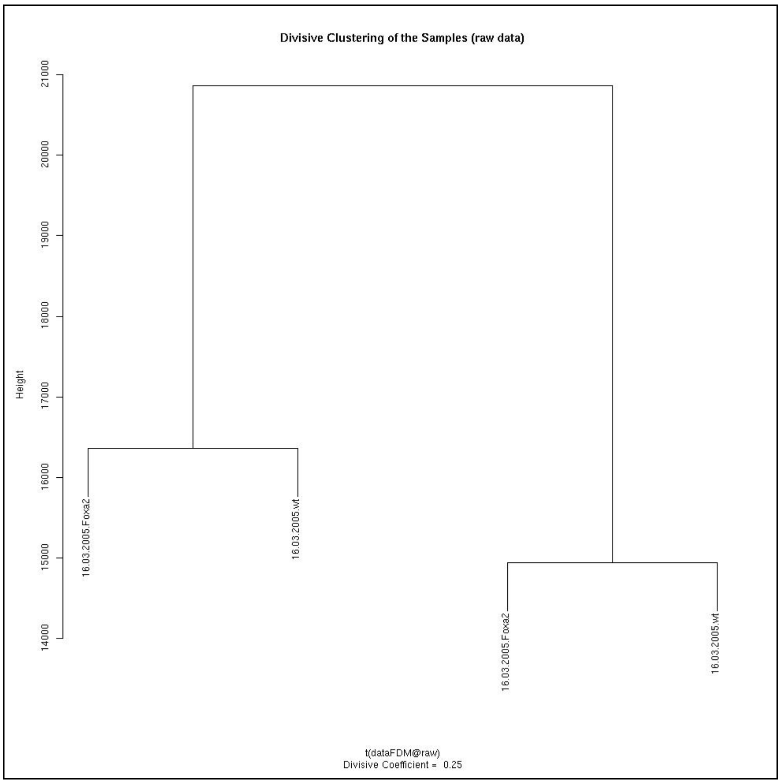

Figure 10: Diana Clustering on raw log-scaled data for MOE4302

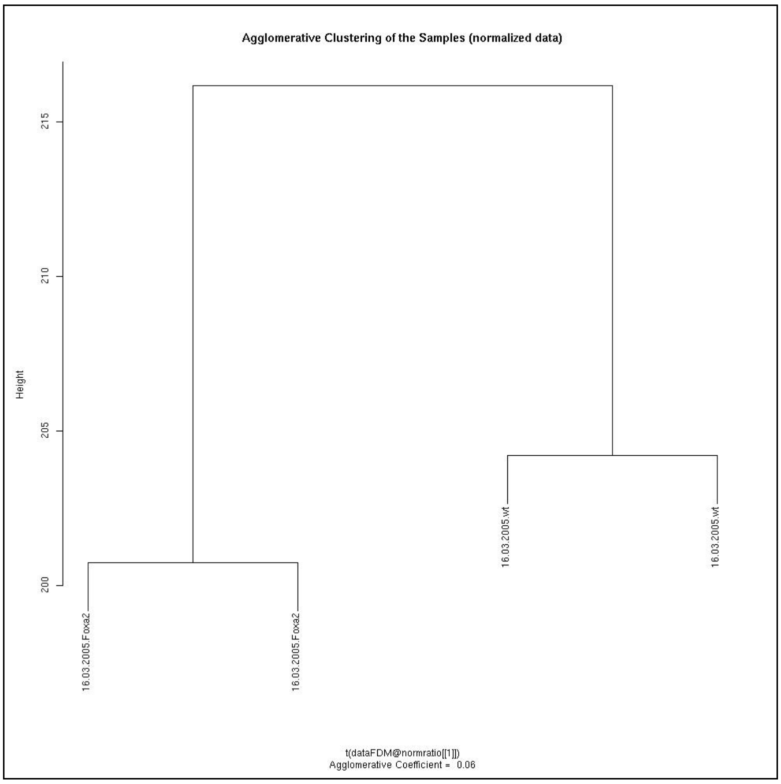

Figure 11: Agglomerative Clustering on normalized log-scaled data for MOE4302

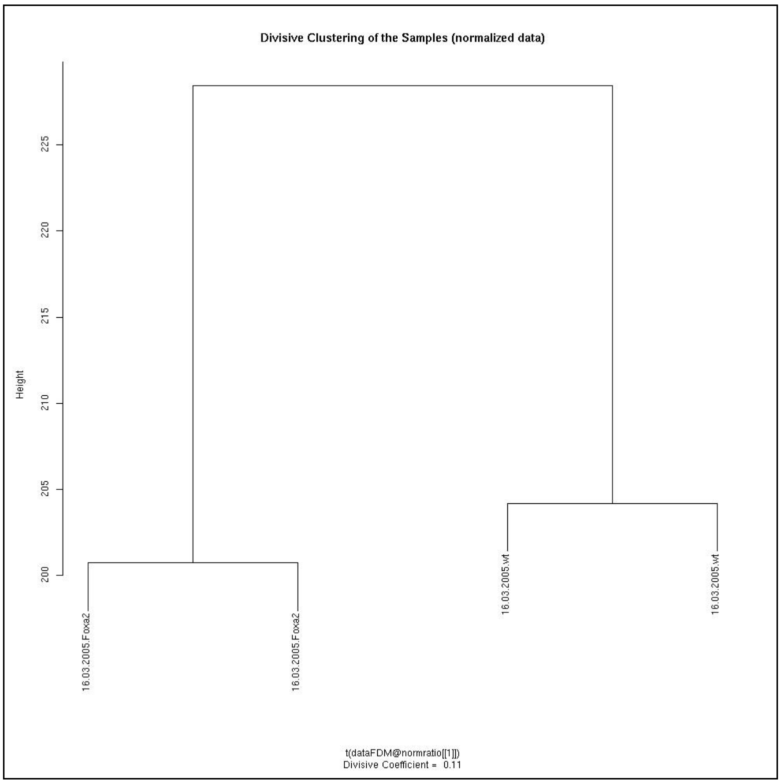

Figure 12: Diana Clustering on normalized log-scaled data for MOE4302

3 Gene-wise testing

3.1 Wilcoxon paired rank tests on chip designs separately (single probes)

Since the current dataset consists of two replicates only, we perform a Wilcoxon paired rank test on the 22 or so (2 chips $*$ 11 probes) probes.

Probesets (genes) are called significantly differentially expressed if the have a p-value ≤ 0.05 (i.e. 5% significance level).

Different multiple testing procedures are applied [1]:

- none (`raw`)
- Bonferrroni (`bonf`)
- Benjamini-Hochberg (`BH`, (Benjamini and Hochberg, 1997))

Using the (over-pessimistic) Bonferroni correction revealed 1 (MOE4302) and 601 (MG-U74) significant probesets. The less stringent Benjamini-Hochberg False Discovery Rate correction found 4199 and 3256 (MG-U74 and MOE4302, respectively) significantly differential probesets/genes (see tab. 4 and 5). Out of these 962 are in common (23% and 30%, respectively).

[1] Controlling the false discovery rate or family-wise error rate is essential because we would expect 624 or 2255 (MG-U74 and MOE4302, respectively) false positive genes without correction

3.2 MG-U74

Affy ID	GeneSymbol	BHcorrected Pval	Mean Expression
100004_at	5930412E23Rik	0.0000098	6.6662276
100005_at	Traf4	0.0006008	8.0116606
100006_at	Cdh11	0.0000248	4.8792883
100007_at	Irf2bp1	0.0101358	8.0941660
100014_at	Tlk2	0.0017358	6.6539897
100020_at	Slc4a2	0.0096015	7.6707923
100023_at	Mybl2	0.0030382	7.9556409
100024_at	Shrm	0.0122205	8.1180267
100026_at	Bcat1	0.0038649	8.9806685
100027_s_at	Pex14	0.0154561	8.3720051
100029_at	Pex14	0.0001911	9.3192290
100030_at	Upp	0.0096031	7.1405391
100032_at	D030041N15Rik	0.0006434	7.2001727
100033_at	Msh2	0.0000150	9.2039891
100037_at	Ddx18	0.0000026	7.9521699
100041_at	3010027G13Rik	0.0000163	9.4339150
100046_at	Mthfd2	0.0081474	9.0650285
100048_at	Rap1a	0.0004548	5.2163079
100050_at	Idb1	0.0000015	8.9895124
100056_at	Fbxw2	0.0037500	7.1779438
100057_at	2510027N19Rik	0.0004548	7.3264645
100062_at	Mcm3	0.0000163	9.1070283
100065_r_at	Gja1	0.0005229	8.0243812
100066_at	Gart	0.0000405	9.6177089
100072_at	Ciao1−pending	0.0013419	7.7517029
100073_at	2510005D08Rik	0.0000895	7.5823129
100074_at	2400003B06Rik	0.0000708	7.0147543
100078_at	Apoa4	0.0001044	7.9180238
100079_at	Ndufb9	0.0019703	8.9316759
100081_at	Stip1	0.0000063	9.3203527
100084_at	Vil2	0.0030388	8.9872437
100086_at	Lrpap1	0.0002384	8.3711013
100088_at	Ppp1cb	0.0000044	8.6980397
100089_at	Ppic	0.0001749	7.9885491
100093_at	Pctk1	0.0004548	7.9775591
100094_at	Supt5h	0.0000150	9.2316971
100095_at	Scarb1	0.0000163	8.2105635
100099_at	Smpd1	0.0083955	7.7039427
100101_at	Snrpa	0.0000238	9.2220664
100112_at	Cxcl12	0.0000016	7.1904660
100113_s_at	Kifap3	0.0002965	6.9180876
100115_at	2610028H07Rik	0.0000126	8.0038585
100116_at	2810417H13Rik	0.0000293	9.6448770
100123_f_at	Itgb1	0.0000088	10.6159002
100124_r_at	Itgb1	0.0000040	8.3994062
100125_at	Pa2g4	0.0003074	8.6103725
100126_at	Chrac1	0.0065145	7.7697454
100128_at	Cdc2a	0.0030388	10.1546971
100130_at	Jun	0.0002565	4.3361073
100131_at	Sgne1	0.0143004	3.6884494
100133_at	Fyn	0.0016284	5.7833594
100136_at	Lamp2	0.0096031	5.2891042
100138_f_at	Rbm14	0.0005607	8.9239628
100139_at	Pcsk1n	0.0000345	6.5134013
100142_at	Dgcr2	0.0000126	8.3454953
100144_at	Ncl	0.0000345	12.4482189
100148_at	Ctcf	0.0000013	8.2245088
100156_at	Mcm5	0.0000010	11.0642887
100213_f_at	Rpl41	0.0020982	13.5760691
100289_at	Efna3	0.0019703	8.0829814
100290_f_at	6230416J20Rik	0.0001911	6.6231661
100296_at	Tex2	0.0072900	5.9638229
100297_at	Wdr26	0.0001127	5.6825524
100306_at	2700007P21Rik	0.0000044	5.2681272
100320_at	Kpna4	0.0006217	5.1581370
100321_f_at	2210418O10Rik	0.0043501	3.6238369
100323_at	Amd1	0.0000048	9.4096740
100324_g_at	Amd1	0.0000093	6.7245167
100331_g_at	Prdx2	0.0000048	11.5132186
100332_s_at	Prdx6	0.0000163	9.6255096
100343_f_at	Tuba1	0.0002565	12.2121604
100344_at	BC026585	0.0081474	7.9406382
100345_f_at	Vamp8	0.0002574	8.4655455
100348_at	X83313	0.0000026	7.7909235
100352_at	Hspa4	0.0007377	5.9164181
100353_g_at	Hspa4	0.0000013	8.9768837
100380_at	H3f3a	0.0000318	10.4370830
100400_at	4921531G14Rik	0.0000050	5.8523029
100401_at	Son	0.0132266	7.0171054
100403_at	Mylc2a	0.0000106	6.8859643
100405_at	Cbx3	0.0000069	7.6709558
100407_at	Gal	0.0001128	7.0005362

100408_at	Pole3	0.0000827	6.7107067
100410_at	C330027G06Rik	0.0007377	7.7954645
100413_at	Zap3−pending	0.0001528	8.3671329
100416_at	Melk	0.0007375	3.5357511
100417_at	AI643885	0.0000058	6.4243987
100429_at	Ppox	0.0018496	5.2413631
100432_f_at	Mdfi	0.0006008	5.3991926
100433_r_at	Mdfi	0.0139359	5.5585162
100442_at	Tbrg4	0.0000008	7.5379522
100443_at	Bcat2	0.0000536	7.5677698
100451_at	Hsf1	0.0007895	6.9977553
100457_at	Glg1	0.0000008	8.6627219
100458_at	Napb	0.0005413	4.4462862
100459_at	Rad50	0.0065145	7.9475078
100460_at	Sh2bp1	0.0000013	7.8482779
100461_at	Polr2j	0.0096031	8.8896152
100462_at	Arf6	0.0013419	4.8256593
100464_at	3110043O21Rik	0.0003484	5.5628095
100468_g_at	Lyl1	0.0015268	4.4924735
100469_at	Nfya	0.0058140	7.1667795
100472_at	Enah	0.0000558	7.9388746
100475_at	Trim25	0.0068924	7.1160661
100479_at	Dnmt3a	0.0000138	7.7405417
100482_at	BC023040	0.0000019	8.6239177
100489_at	Pde7a	0.0086090	7.0055094
100491_at	Slc16a2	0.0066999	5.8627999
100492_at	Ap2a2	0.0000967	8.5982547
100493_at	Hsd11b2	0.0018496	7.0967630
100495_at	Dvl2	0.0005605	7.5670652
100497_at	Stx3	0.0118937	3.2722591
100499_at	Stx3	0.0000026	8.0254924
100509_at	Rnf19	0.0000037	6.7420978
100512_at	Uchl5	0.0000126	7.3697259
100513_at	Ddef1	0.0090939	7.1696446
100514_at	Gna13	0.0000967	7.2531836
100515_at	Furin	0.0005607	8.0651480
100522_s_at	Wbp5	0.0010320	10.7853363
100523_r_at	Wbp5	0.0000457	7.6974344
100525_at	Htf9c	0.0112863	7.7997828
100526_f_at	Adam3	0.0082210	1.6376345
100527_at	D11Ertd99e	0.0007377	8.6552453
100528_at	Ube2h	0.0041010	6.4791728
100534_at	Tsnax	0.0000008	8.2013116
100535_at	E130105L11Rik	0.0000022	7.6097140
100538_at	Sod1	0.0000116	10.3090360
100539_at	Bach−pending	0.0002384	7.1065990
100540_at	Lta4h	0.0000559	9.6839688
100543_s_at	Brd7	0.0000009	8.5366865
100544_at	Brd7	0.0000013	6.0717312
100553_at	Trim27	0.0000405	9.7737152
100554_at	Pdlim1	0.0000193	7.1476513
100555_at	Dscr1	0.0000040	7.8034111
100557_g_at	2310046H11Rik	0.0000008	9.8924026
100559_at	Dhx16	0.0000708	7.2233490
100560_at	Pafah1b1	0.0011775	7.2685219
100561_at	Iqgap1	0.0000040	8.4261346
100565_at	Gnpda1	0.0000116	7.2193653
100566_at	Igfbp5	0.0000013	7.5014616
100568_at	Abce1	0.0000009	9.4752231
100569_at	Anxa2	0.0000106	7.1787368
100571_at	Laptm4b	0.0038649	9.9196864
100572_at	Tm9sf2	0.0086090	7.6049144
100573_f_at	Gpi1	0.0000105	8.0254399
100574_f_at	Gpi1	0.0000082	11.5959241
100575_at	Ard1	0.0000090	8.1283867
100576_at	Pafah1b3	0.0086090	8.5818796
100577_at	Snrpd1	0.0000150	7.1194467
100578_at	Impdh2	0.0000163	10.6616934
100579_s_at	Clta	0.0112863	9.4385096
100581_at	Cstb	0.0000248	9.3080164
100582_at	Snx17	0.0090939	6.9149151
100584_at	Anxa4	0.0038649	5.8371435
100587_f_at	5730403B10Rik	0.0002859	6.2432086
100595_at	Ptp4a2	0.0132266	8.6863697
100597_at	Gyg1	0.0003303	5.3543695
100601_at	Itgb5	0.0038649	8.3647486
100602_at	Rps15	0.0065133	2.0274632
100603_at	Dncic2	0.0000476	7.1761923
100606_at	Prnp	0.0006658	7.7908801
100608_at	Sptlc1	0.0000012	8.2444111
100612_at	Rrm1	0.0000034	9.3991912
100613_at	Fkbp8	0.0000019	9.1595072
100616_at	Cenpa	0.0025288	7.8061682
100617_at	Slc25a5	0.0086090	3.9431403
100618_f_at	Slc25a5	0.0000023	9.0004476
100622_at	Prdx6	0.0009032	9.9975185
100626_at	Odf2	0.0083725	5.5218341

100633_at	2810484M10Rik	0.0065133	7.2219118
100635_at	Sara	0.0003678	9.6125732
100636_at	Eif4ebp1	0.0000138	8.4201304
100684_at	Prkcsh	0.0132266	9.8236126
100686_at	Rps2	0.0000098	13.0356020
100694_at	Rplp1	0.0146787	13.0952064
100706_f_at	Sfmbt2	0.0000318	6.6261744
100708_at	H3f3b	0.0006008	11.1395154
100711_at	Rpl10a	0.0000559	12.0886107
100713_at	LOC170938	0.0017914	5.6596619
100717_at	U90926	0.0146787	7.2326542
100718_at	Ptma	0.0000053	12.0262548
100720_at	Pabpc1	0.0002385	12.1535790
100727_at	Rpl28	0.0007895	12.4765280
100732_at	Rps8	0.0000828	12.5601372
100733_at	Psma2	0.0054898	10.6910732
100734_at	Rpl3	0.0000345	12.3538354
100753_at	Atp5a1	0.0001528	11.2785783
100756_r_at	Tyms	0.0139338	7.1531890
100758_at	Rps28	0.0000023	12.3911782
100772_g_at	Blnk	0.0166877	3.9488642
100775_at		0.0000559	8.4603674
100780_at	Rps4x	0.0000248	12.6813418
100828_at		0.0096031	6.0288414
100878_at	Strn3	0.0119030	7.9142516
100882_at	Defb1	0.0003678	1.8961749
100885_at	Nek2	0.0003303	7.9669225
100886_f_at	Mrpl45	0.0000031	8.0378926
100889_at	BC042901	0.0018496	8.4851731
100890_at	Chaf1b	0.0001528	7.6402051
100891_at	Mcsp	0.0106982	4.7786618
100892_at	Ndufaf1	0.0000178	6.9982807
100894_at	Orc5l	0.0000210	7.5812492
100895_at	D16Bwg1547e	0.0034293	7.0203378
100900_at	Hcfc1	0.0000015	9.5479804
100901_at	Hcfc1	0.0000516	6.4703381
100902_at	2610019F03Rik	0.0000895	6.2008174
100903_at	Adprtl2	0.0000013	8.6828075
100904_at	D14Ertd209e	0.0032287	7.3392349
100905_at	1700056O17Rik	0.0000150	6.2988014
100910_at	Surf2	0.0026894	8.1355630
100912_at	2410012M04Rik	0.0000318	6.5911316
100914_at		0.0000210	6.4408643
100917_at	2410080P20Rik	0.0077067	6.7419933
100923_at	Myo10	0.0002058	7.9392479
100924_at	Gata3	0.0050322	5.5486920
100925_at	2700089E24Rik	0.0034279	3.4525614
100928_at	Fbln2	0.0000210	6.9329206
100929_at		0.0013416	5.9457594
100931_at	Arsa	0.0000031	6.6366348
100935_at	Tcfl4	0.0020982	6.9463117
100941_at	Miz1	0.0001217	7.2671849
100946_at	BC020447	0.0011775	5.2074217
100951_at	Pkd2	0.0050332	7.4964431
100953_at	Timeless	0.0002759	8.7219329
100954_at	Hrb	0.0000374	6.7592611
100955_at	2700084L22Rik	0.0000828	6.5982598
100957_at	Ssbp1	0.0014318	8.5259420
100958_at	X83327	0.0002759	8.1558015
100959_at	S100a13	0.0002216	6.3652519
100963_at	Brp17	0.0146765	6.3128638
100964_at	Vti1b	0.0112845	7.8025224
100967_at	Slc27a2	0.0070862	3.4543874
100968_at	Cstf3	0.0020982	7.5766037
100970_at	Akt1	0.0000106	7.4268022
100977_at	D530020C15Rik	0.0000010	7.5079415
100978_at	Frg1	0.0000037	7.8046733
100979_at	Rnf138	0.0000021	7.9968900
100980_at	Rock1	0.0000009	6.4569914
100981_at	Ifit1	0.0011028	1.2122802
100983_at	Prep	0.0006008	6.8744586
100984_at	Atf1	0.0002759	8.1849678
100985_at	Siah1a	0.0000116	7.0420555
100987_f_at	Hspbp1−pending	0.0112863	7.8540536
100990_g_at	Itgb1bp1	0.0024639	6.3219120
100992_at	Edr1	0.0000044	8.7496538
100996_at	AU041707	0.0001911	3.5809674
100998_at	H2−Ab1	0.0025282	4.1739558
101002_at	Oazin	0.0000069	8.9789849
101003_at	Sfrs3	0.0000318	6.6622565
101004_f_at	Sfrs3	0.0000163	9.5497332
101007_at	Mknk2	0.0001911	8.1223533
101008_at	Tcerg1	0.0000009	7.8894908
101011_at	Cct4	0.0000967	11.1966045
101013_at	Oaz1	0.0000028	11.5464316
101016_at	Arf1	0.0000023	11.6539530
101017_at	Cdk4	0.0009656	8.6722067

101019_at	Ctsc	0.0000010	7.6434499
101024_i_at	Sprr2a	0.0107577	5.4646439
101025_f_at	Sprr2a	0.0074951	6.5601960
101028_i_at	Actc1	0.0001887	4.3696243
101030_at	Arhb	0.0000439	7.4728135
101035_at	Api5	0.0001647	8.6614127
101036_at	Tomm20−pending	0.0043541	6.5393135
101039_at	Col4a2	0.0002058	8.9428362
101040_at	Capn2	0.0000090	6.9143787
101044_at	Alad	0.0000021	8.2987610
101050_at	0610038L10Rik	0.0162695	6.9674584
101053_at	2610031L17Rik	0.0000210	9.1625936
101055_at	Ppgb	0.0061552	8.2338898
101056_at	Rdx	0.0000559	9.6980740
101057_at	A430005L14Rik	0.0000069	8.1756884
101059_at	Ndn	0.0081474	7.8576062
101060_at	Grp58	0.0000293	8.8669667
101062_at	Hmox2	0.0132266	5.5323493
101063_at	Tncc	0.0005607	4.4390255
101064_at	Plrg1	0.0035330	8.8441791
101065_at	Pcna	0.0000439	10.5227360
101067_at	2010005E08Rik	0.0000178	8.6722847
101069_g_at	Mkrn1	0.0002759	8.7945856
101070_at	Mkrn1	0.0003425	6.3630412
101072_at	AA792569	0.0000163	9.9490222
101073_at	Lrp1	0.0015272	8.1682787
101079_at	Nxf1	0.0002759	7.7122508
101081_at	Ctbp1	0.0000075	9.3671189
101082_at	Mod1	0.0139359	5.8750395
101083_s_at	Lsm2	0.0000138	9.3213773
101084_f_at	Dpm3	0.0020982	7.4381783
101086_f_at	Cnbp	0.0003678	10.7817679
101088_f_at	Cnbp	0.0000116	10.2332188
101091_at	Ubl1	0.0003949	10.2280412
101093_at	Col4a1	0.0000106	9.9017438
101094_at	Hig1−pending	0.0000228	6.8866277
101095_at	Mfap2	0.0012165	7.0667888
101096_s_at	Hs1bp1	0.0011025	8.3483987
101101_at	Ppp2cb	0.0002385	8.8587206
101102_at	Igbp1	0.0000072	7.6326536
101104_at	Dscr3	0.0000040	7.7632599
101105_at	Banf1	0.0000016	10.7411242
101106_at	G3bp2−pending	0.0000331	9.2071979
101107_at	Calu	0.0012165	8.7551378
101108_at	Nasp	0.0000345	8.9939084
101111_at	Arha	0.0068912	5.6565377
101112_g_at	Arha	0.0000163	8.5198087
101113_at	Arha	0.0000048	8.5551762
101129_at	Rpl5	0.0026894	12.5297285
101135_at	Calcr	0.0023037	3.4929330
101137_at	Rps3	0.0000019	12.7470436
101148_at	Prkcl	0.0034293	5.5789009
101151_at	Rcvrn	0.0004878	2.4294125
101158_at	Zfp2	0.0096031	4.6441169
101165_at		0.0090939	7.5715819
101179_at		0.0011775	8.8578524
101180_at	Atm	0.0000130	7.0034025
101186_at	Ppnr−pending	0.0119030	4.9255297
101207_at	Ppia	0.0000708	13.1876826
101212_at	Rps7	0.0000023	12.2754623
101214_f_at	Gapd	0.0006892	12.6425363
101221_at		0.0011023	5.1304965
101227_at		0.0022326	3.7562857
101254_at	Ran	0.0000163	12.9408058
101288_at	Fv4	0.0083924	1.8633843
101289_f_at	Klk21	0.0022331	3.8308350
101291_at	Srm	0.0001087	5.6497435
101294_g_at	G6pdx	0.0000766	7.0228964
101295_s_at	Clns1a	0.0011025	7.9773362
101344_at	Cckbr	0.0081474	6.8793288
101346_at	−−−	0.0027725	4.5086245
101350_g_at	Plk	0.0046129	9.5397226
101356_at	Tk2	0.0000736	5.5920422
101357_at	Ap2a1	0.0101358	6.8124696
101359_at	Lamb2	0.0001127	5.3536670
101363_at	Adora2a	0.0096015	6.2915217
101366_f_at	1200009I24Rik	0.0000828	6.0693173
101367_at	Dctn1	0.0000248	8.0273291
101368_at	Pem	0.0000010	9.1241320
101370_at	Kpna1	0.0000422	7.1265650
101371_at	Cpsf4	0.0112845	8.3198825
101372_at	Trip13	0.0000079	7.4700764
101381_at	Rabep1	0.0011772	5.2310118
101385_at	Mbd3	0.0014314	9.3921575
101392_at	6330407G04Rik	0.0000058	7.0316297
101398_at	Stxbp2	0.0005607	7.7162237
101401_at	Bet1	0.0077080	5.4246802

101404_at	2310061I09Rik	0.0012986	5.5299970
101409_at	Lgtn	0.0051821	7.8212362
101413_at	4432411E13Rik	0.0135384	3.1299411
101414_at	Lmnb2	0.0000766	7.1842825
101416_f_at	Bat4	0.0000374	7.5511707
101422_at	Fnbp4	0.0000040	7.0040261
101430_at	Sox4	0.0036411	7.8651313
101437_at	Stk2	0.0011818	5.9824232
101439_at	Arl6	0.0000476	7.6553839
101444_at	Gt(ROSA)26asSor	0.0000559	8.3338195
101445_at	Dnmt1	0.0000828	9.8526748
101446_at	Tpd52l1	0.0000040	6.1585524
101448_at	Hgs	0.0001044	9.3612336
101456_at	Zfp106	0.0000150	7.7154373
101458_at	Wee1	0.0000318	7.2429251
101459_at	Chd1	0.0000016	8.7990589
101461_f_at	Pja1	0.0000090	10.0877168
101462_r_at	Pja1	0.0054888	4.1675859
101464_at	Timp1	0.0119030	7.1044376
101468_at	Pfc	0.0007893	4.6135687
101475_at	Bmi1	0.0024645	7.0533352
101476_at	Pabpn1	0.0000034	9.7302906
101481_at	Rnf34	0.0003810	7.0279046
101482_at	Ppp1cc	0.0000895	9.5721546
101484_at	Nbr1	0.0018496	8.1511062
101485_at	Ddx48	0.0036404	10.3664319
101486_at	Psmb10	0.0000345	7.2983617
101489_at	Amd1	0.0000026	10.0404143
101490_at	1810010A06Rik	0.0002473	7.7414808
101498_at	Impa1	0.0001774	7.3374801
101499_at	Ilk	0.0000318	7.5684523
101501_r_at	Impact	0.0000439	4.6866578
101506_at	Snrpa1	0.0000053	8.6169604
101509_at	Vbp1	0.0000605	10.0582079
101510_at	Psme1	0.0015268	7.6741542
101514_at	Trappc3	0.0031318	6.2855272
101516_at	Cd59a	0.0062624	6.8237923
101517_at	Tex261	0.0038649	7.3137579
101519_at	Srp14	0.0011775	9.3156004
101520_at	Spata6	0.0051821	6.1014089
101521_at	Birc5	0.0032287	9.0063028
101523_at	2610510D13Rik	0.0000053	11.5591480
101524_at	2610510D13Rik	0.0000142	5.6642448
101525_at	Ndufb10	0.0000193	8.6467484
101526_at	Msx1	0.0000037	8.3633981
101527_at	Tcea1	0.0000026	9.1869703
101528_at	Tcea1	0.0000011	8.6301148
101529_g_at	Tcea1	0.0000032	8.2400085
101530_at	Snrp116−pending	0.0001128	9.3283633
101536_at	Ncor1	0.0000048	7.7432846
101540_at	Tdg	0.0000015	9.6617720
101542_f_at	Ddx3x	0.0000069	9.9889325
101543_f_at	Tuba6	0.0000967	12.7930898
101546_at	Neu1	0.0146787	6.4024226
101548_at	Synj2bp	0.0046129	4.3187920
101555_at	Rac1	0.0006436	11.1885789
101557_at	Bckdk	0.0000034	8.8964297
101558_s_at	Psmb5	0.0000021	10.0290186
101560_at	Emb	0.0001217	11.0933662
101561_at	Mt2	0.0001128	10.1648960
101562_at	Hsp70−4	0.0000040	8.0187306
101564_at	Cnot7	0.0000028	6.2798855
101568_at	1700024N20Rik	0.0000248	7.0184701
101573_f_at	Rpl27a	0.0048900	12.5904836
101577_at	Rps6	0.0000023	12.8064187
101579_at	Srp9	0.0000318	7.5136028
101580_at	Cox7b	0.0010320	9.8124847
101581_at	Ube3a	0.0000090	6.2218815
101582_at	LOC230737	0.0023770	7.3906953
101583_at	Btg2	0.0003074	7.4331593
101585_at	Pgrmc1	0.0081474	8.9094583
101589_at	Hmgn2	0.0000163	9.5946605
101590_at	Lamp2	0.0000754	7.2629685
101593_at	Crip2	0.0112863	5.8587133
101601_at	DXErtd223e	0.0023759	3.3195070
101607_at	C80068	0.0003425	5.5985409
101617_s_at		0.0054888	6.1170580
101634_at	Npm1	0.0000048	12.6417935
101648_at	Foxd4	0.0000150	5.3637500
101664_at	Rps3a	0.0000967	12.8272351
101665_at	Nr5a1	0.0090939	8.3969731
101676_at	Gpx3	0.0030388	9.0389778
101680_at	Rpl27a	0.0000318	11.8489768
101687_r_at		0.0086379	3.9349523
101694_f_at	Myst2	0.0005231	5.8894585
101697_f_at	BC024683	0.0025081	6.6137902
101707_at	Aldh1a2	0.0000106	6.6833964

101741_at	Psmb5	0.0139359	7.7577610
101763_at	Gpr50	0.0086090	7.7870895
101769_at	Pcdha10	0.0036411	5.7793182
101781_f_at		0.0019703	8.1060386
101787_f_at		0.0000269	8.9149925
101834_at	Mapk3	0.0012572	7.4625081
101836_at	Ppm1b	0.0020246	4.5829845
101837_g_at	Ppm1b	0.0000559	7.6588024
101856_at	2900008M13Rik	0.0072900	6.0536105
101858_at	Rfng	0.0061552	7.5051025
101860_at	Gmfb	0.0003948	4.6734285
101861_at	Sgce	0.0003949	7.6291718
101865_at	Pip5k2a	0.0119030	5.3012307
101866_at	Arfrp1	0.0003678	6.9261817
101867_at	Gpam	0.0018488	7.2228497
101876_s_at	H2−T10	0.0114071	4.7859813
101877_at	Slc31a1	0.0016284	8.1999413
101883_s_at	Xlr3a	0.0000439	7.0461352
101888_at	Rora	0.0079236	1.4132563
101890_f_at	Zrf2	0.0000015	8.9526992
101892_f_at	Fts	0.0000345	5.7559065
101894_s_at	Fts	0.0052280	5.2660027
101899_at	F2	0.0074951	4.8303708
101902_at	Rbpsuh	0.0000069	6.2470760
101905_at	Itch	0.0007129	7.4418250
101906_at	Smc4l1	0.0000021	9.8067232
101913_at	T25545	0.0065145	8.9595856
101914_at	D1Ertd396e	0.0003187	7.3342125
101916_at	Llglh2	0.0013416	8.1269176
101921_at	Rab4a	0.0000018	8.0687518
101926_at	Pim2	0.0000044	9.3294683
101929_at	6720463E02Rik	0.0000150	7.7598619
101931_at	Ptdss1	0.0007377	8.3448233
101936_at	Clk4	0.0000044	6.8882130
101937_s_at	Clk4	0.0000439	5.7822996
101938_at	Pabpc2	0.0081474	5.0666234
101942_at	Sfrs14	0.0019703	7.4442042
101945_g_at	Lypla1	0.0154561	5.0714429
101946_at	Lypla1	0.0000116	7.4240972
101947_at	Nakap95−pending	0.0000654	6.9037336
101948_at	Lamb1−1	0.0009030	6.8083256
101954_at	H2afz	0.0001128	11.1141170
101955_at	Hspa5	0.0000150	11.2938882
101957_f_at	Adprt1	0.0000193	8.5044992
101958_f_at	Tfdp1	0.0000010	9.0078648
101959_r_at	Tfdp1	0.0000012	10.4478054
101960_at	D10Wsu52e	0.0000558	8.4938558
101961_at	Bub3	0.0000708	10.1072677
101962_at	2610007K22Rik	0.0000026	9.7821093
101963_at	Ctsl	0.0000009	10.5084150
101964_at	Tkt	0.0000053	10.3967444
101971_at	2500002L14Rik	0.0000090	8.4922318
101972_at	Kdap	0.0139359	2.1295723
101976_at	Cops4	0.0008732	8.1680051
101977_at	Nudt3	0.0000106	8.2321335
101979_at	Gadd45g	0.0004548	5.9489601
101982_at	Vasp	0.0004878	8.4375352
101984_at	Atox1	0.0125493	9.6292074
101989_at	Uqcrc1	0.0001044	9.9817147
101992_at	Psmb6	0.0054898	10.0139111
101996_at	Ptpn2	0.0043501	6.9154428
101997_at	Apg12l	0.0032287	7.4202543
101998_at	4833420G17Rik	0.0002759	6.2330536
102001_at	Rrm2	0.0000008	10.1877581
102002_at	Ubqln2	0.0000058	8.4061219
102003_at	AW049900	0.0004545	7.3656365
102014_at	Homer3	0.0112863	6.0665293
102017_at	Prpf4b	0.0004876	6.4564152
102018_at	Cnot3	0.0000012	7.8254904
102019_at	Mrpl13	0.0023037	8.5639503
102026_s_at	Chkl	0.0009334	5.4434592
102030_at	4833408C14Rik	0.0001126	4.3832071
102031_at	Rnaseh1	0.0054898	4.8228446
102032_at	Twsg1	0.0058140	7.3304672
102035_at	Tpmt	0.0002565	6.1427492
102037_at	Mapre2	0.0013416	5.7871400
102039_at	Gtf2h4	0.0000053	8.5510691
102046_at	Nssr	0.0000210	7.0590353
102047_at	Nmt1	0.0006436	8.9481156
102052_at	1300003D03Rik	0.0028592	5.0917169
102054_at	Ankfy1	0.0000895	7.9719283
102057_r_at	2610002J02Rik	0.0091499	4.5385716
102058_at	Mrpl9	0.0048900	6.6677983
102060_at	Golga4	0.0139318	7.7260942
102061_at	Mlf1	0.0005803	4.4392951
102062_at	Smarcc1	0.0014318	9.1815714
102069_at	Mtf2	0.0000605	8.1806249

102070_at	Col9a3	0.0115874	6.6471569
102071_at	1500034J01Rik	0.0001417	8.4921035
102072_g_at	1500034J01Rik	0.0000058	8.3142292
102093_f_at	Sfrs4	0.0090939	7.1404050
102098_at		0.0065133	11.9024548
102109_at	Rpl13	0.0000031	13.0863827
102118_at	Ankrd17	0.0098654	4.3579485
102120_f_at	Hpcal1	0.0035330	6.3435496
102124_f_at	Cox4a	0.0000292	5.8358819
102126_at	Rps12	0.0000098	12.9363166
102130_f_at		0.0003188	7.7930630
102131_f_at	Rnf20	0.0022331	7.0699502
102134_f_at	Atp5g2	0.0032287	8.4490811
102141_f_at	4933434E20Rik	0.0041010	6.4207274
102144_f_at		0.0000081	7.1881985
102152_f_at	Igh−VS107	0.0063303	6.9689279
102161_f_at	H2−D1	0.0096031	6.6534795
102163_at		0.0003188	7.2473170
102171_r_at	Nr1i3	0.0154561	6.1480546
102193_at	4933424N09Rik	0.0001417	7.4758266
102194_at	2810432D09Rik	0.0011023	8.1083488
102195_at	Map4k4	0.0086090	7.0500293
102202_s_at	Mpv17	0.0098526	5.9403056
102208_at	Siat10	0.0004878	4.7813202
102210_at	Dtnb	0.0081474	6.4497332
102211_r_at	AI605202	0.0002778	3.0210107
102218_at	Il6	0.0162625	0.7052189
102220_at	Utf1	0.0000028	8.0118050
102228_at	Lat	0.0026888	6.2887744
102234_at	1810037I17Rik	0.0000034	4.9595861
102235_at	Lmyc1	0.0011772	7.3171699
102247_at	Ubr1	0.0038618	4.7666235
102248_f_at	Cask	0.0000106	7.2075823
102252_at	Pfdn2	0.0000895	8.5712960
102257_at	Pknox1	0.0132266	6.1057178
102258_at	Stra6	0.0065145	4.2130413
102259_at	Ywhag	0.0034293	5.7267912
102268_at	1700021P10Rik	0.0000724	7.6501314
102271_at	D11Bwg0280e	0.0009656	7.8952877
102277_at	Zfp26	0.0023770	6.6281237
102283_at	Tiam1	0.0043541	6.4258916
102286_at	Araf	0.0056487	6.9992074
102304_f_at	Zfp61	0.0000023	5.1289237
102308_at	Tulp3	0.0020982	8.6859310
102309_at	Zfp326	0.0000082	7.2822587
102315_at	Tex292	0.0000008	9.4802419
102317_at	Vamp4	0.0081446	6.5576640
102319_at	Snx12	0.0006658	7.3250000
102320_at	Snx12	0.0013419	6.7498290
102321_at	Adcy6	0.0013416	6.8172817
102322_at	Ugdh	0.0000021	9.3049918
102324_at	Dnajc4	0.0112845	5.4819097
102328_at	Casp8	0.0015272	4.7098345
102329_at	Cideb	0.0068924	5.6612271
102331_at	St5	0.0053329	6.1023488
102335_at	Kcnk1	0.0000015	8.1072309
102336_at	Rw1−pending	0.0000605	8.3021003
102340_at	Mina	0.0003425	5.9691090
102343_at	425O18−1	0.0009656	7.8408487
102344_s_at	Tcea3	0.0005050	4.9497431
102346_at	Ercc3	0.0001313	6.5504320
102348_at		0.0000269	8.7657695
102352_at	Galnt9	0.0014318	6.0304413
102360_at	Mthfr	0.0011025	6.3807322
102364_at	Jund1	0.0006008	10.7855922
102370_at	retsdr2−pending	0.0011775	7.7987412
102375_at	Smyd5	0.0000317	7.6799886
102379_at	Rassf1	0.0003424	5.2067352
102381_at	Facl4	0.0000019	7.0969278
102384_at	2610209L14Rik	0.0000063	5.8455446
102385_at	2610318G08Rik	0.0000048	8.7189003
102387_at	MGC6735	0.0086090	8.9635288
102395_at	Pmp22	0.0000228	8.3554014
102398_at	Rxrb	0.0077080	8.5681601
102399_at	Rbpms	0.0000138	8.7631188
102401_at	Irf1	0.0003732	5.2744313
102402_at	Gbas	0.0000023	8.2036599
102409_at	2010003I05Rik	0.0001417	9.2622939
102412_at	AW541137	0.0017358	9.0535085
102427_at	3830421F13Rik	0.0000026	7.9701204
102476_f_at	Xpo7	0.0000163	8.4636936
102478_f_at	Fbxw4	0.0041010	5.9347011
102599_at	Tpt1	0.0000011	13.0628193
102624_at	Stc2	0.0001313	6.1977175
102625_f_at	Zfp54	0.0083740	3.4433659
102627_at	Igf2bp1	0.0000048	10.2708606
102632_at	Calmbp1	0.0000605	6.8625159

102639_at	Chst2	0.0061541	5.3171552
102646_at	Murr2	0.0000374	6.9852520
102647_g_at	Murr2	0.0000016	8.7674241
102649_s_at	Raet1a	0.0115892	6.2575305
102656_at	Itga4	0.0139695	2.3264888
102665_at	Trh	0.0000293	8.0461877
102667_at	Wnt3a	0.0002473	5.8617374
102670_at	Vps54	0.0006892	7.2708409
102686_at	BC024816	0.0051821	8.0139027
102697_at	Pitpnb	0.0014318	7.4682959
102709_at	Hira	0.0058140	4.7333081
102713_at	Gata4	0.0081460	7.0417082
102734_at	Birc3	0.0000037	6.5173121
102735_at	Tex9	0.0061541	2.7402194
102752_at	Shyc	0.0002216	8.3208169
102753_at	Men1	0.0150607	7.1509027
102754_at	Birc6	0.0083740	7.1417206
102758_at	Rbm6	0.0000605	6.1042312
102759_at	Pik3r2	0.0001217	6.9783165
102761_at	Grpel2	0.0026074	6.5109236
102764_at	Trap1a	0.0011023	7.9484238
102765_at	Cops7b	0.0006892	7.5603849
102767_at	Gng12	0.0000374	7.1014014
102771_at	Setdb1	0.0000766	8.3831230
102772_at	Abl1	0.0002058	8.0906796
102774_at	Egf	0.0115874	2.6389848
102776_at	4833420O05Rik	0.0000895	7.2419164
102781_at	Ccnl2	0.0000058	8.4900971
102786_at	Clcn3	0.0000019	8.9704234
102788_s_at	Pitx2	0.0002565	5.2594353
102790_at	Jtb	0.0001313	8.9780607
102792_at	Ung	0.0043501	8.8778790
102794_at	Cxcr4	0.0006892	5.9118224
102795_at	Mesp1	0.0006434	7.6609335
102796_at	Npm3	0.0002473	6.8718863
102807_at	9230112O05Rik	0.0000177	8.0173058
102809_s_at	Lck	0.0077080	6.4287300
102811_at	Ext1	0.0004355	7.2495532
102819_at	Nap1l2	0.0132226	4.9199813
102821_s_at	Ran	0.0000082	12.5587415
102827_at	Nek7	0.0005231	7.4838065
102833_at	Cbx2	0.0054898	8.4107608
102835_at	Ap2a2	0.0009030	7.5838748
102839_at	Plscr1	0.0006008	7.3815368
102841_at	−−−	0.0032287	6.2195979
102852_at	Cdh2	0.0000374	7.8408123
102853_at	Cspg6	0.0000008	8.2228279
102863_at	9130423L19Rik	0.0077080	5.6758915
102866_at	Tead4	0.0146966	6.4030129
102869_at	Efna2	0.0059813	6.3035272
102870_at	5930418K15Rik	0.0000269	8.4705386
102872_f_at	Zfp51	0.0003678	5.1417916
102878_at	Rad52	0.0014784	6.7091860
102890_at	Snta1	0.0015272	7.2161044
102891_at	Wrn	0.0038649	5.9824098
102893_at	Pou2f1	0.0000069	7.8228489
102894_g_at	Pou2f1	0.0008164	7.9255516
102895_at	4921518A06Rik	0.0000075	7.0303230
102911_at	Brca2	0.0000655	7.6078984
102912_at	5430432P15Rik	0.0000193	7.9146006
102915_at	F2rl1	0.0006008	7.1343229
102922_at	1110020B03Rik	0.0096015	5.9731769
102925_at	Dusp9	0.0090939	8.5231042
102927_s_at	Hdh	0.0068912	7.3649947
102928_at	Hdh	0.0011025	8.8716826
102933_at	Plxna3	0.0001416	7.2421397
102936_at	B4galt6	0.0017358	6.5593628
102956_at	Msx2	0.0000476	7.2302371
102959_at	Tle4	0.0000170	4.8983017
102960_at	Rga	0.0128821	6.7918583
102964_at	6230400O18Rik	0.0000044	7.7681380
102965_at	AI481105	0.0032280	5.0445805
102969_at	BC023037	0.0000374	7.4492718
102972_s_at	Dab1	0.0070875	5.2778189
102976_at	Brca1	0.0001044	6.8808903
102980_at	AW536594	0.0000439	6.9139433
102981_at	Gabpa	0.0154538	5.0430360
102982_at	B230308N11Rik	0.0000012	8.6999724
102983_at	Madh1	0.0000034	7.6788966
102984_g_at	Madh1	0.0000008	8.5401068
102985_at	Srpk1	0.0004548	7.8595686
102988_at	Inppl1	0.0106982	8.3812864
102990_at	Col3a1	0.0102466	−0.3049079
102996_at	Ell	0.0000075	7.1879097
103001_at	Vegfb	0.0000516	8.0800813
103010_at	2610005L07Rik	0.0000012	5.9083941
103011_at	Sin3a	0.0000439	9.0537036

103013_at	Usf2	0.0132266	7.4362031
103018_at	Vdp−pending	0.0101375	7.2979322
103020_s_at	Map3k1	0.0005607	7.3252585
103021_r_at	Map3k1	0.0011389	4.5229352
103027_at	1810030O07Rik	0.0000008	7.8585441
103034_at	Ccne1	0.0010320	8.4742069
103035_at	Tap1	0.0021644	4.5220929
103036_at	G22p1	0.0000053	7.5234188
103047_at	Pxmp3	0.0005607	6.9403019
103048_at	Nmyc1	0.0000126	8.6101680
103054_at	Polr2a	0.0058140	9.5816571
103056_at	6230425C22Rik	0.0000075	8.3023779
103057_at	Pold1	0.0000019	9.2117067
103063_at	Zfp62	0.0000075	6.5258853
103064_at	Chek1	0.0000026	7.8472894
103065_at	Slc20a1	0.0001417	8.0850466
103067_at	Rala	0.0000044	7.7237206
103069_at	2700022J23Rik	0.0083740	6.6051481
103071_at	2810429C13Rik	0.0000439	7.6821159
103073_i_at	Taf9	0.0152216	9.8707304
103074_f_at	Taf9	0.0000177	8.5029749
103075_at	Pou5f1	0.0000008	10.4985085
103078_at	BC014685	0.0000210	7.7320204
103079_at	4432409D24Rik	0.0000178	8.3983702
103080_at	Samhd1	0.0001911	6.4624224
103081_at	Baz1b	0.0000069	9.2639629
103082_at	E230022H04Rik	0.0000588	8.0201235
103085_at	Hebp1	0.0007377	5.2301874
103092_at	1110032A10Rik	0.0001128	7.8551497
103094_at	Serf1	0.0026888	6.2540949
103099_f_at	Cgbp−pending	0.0002812	9.4361592
103201_at	Ttk	0.0000044	8.5306264
103203_f_at	4432406C08Rik	0.0000116	6.6933244
103204_r_at	4432406C08Rik	0.0002058	6.7305633
103207_at	Pola1	0.0000021	7.5309384
103212_at	BC006933	0.0041010	8.6363186
103217_at	Cflar	0.0036411	5.0533485
103220_at	5730494G16Rik	0.0002384	7.6894118
103222_at	Eps8	0.0059813	4.4927088
103228_at	Mtmr7	0.0000044	5.7782345
103232_at	6430517J16Rik	0.0000011	8.3812811
103236_at	Ring1	0.0004548	4.9965399
103242_at	Ap1g1	0.0000053	8.1419722
103258_at	Ly75	0.0000008	7.6672530
103260_at	1110060D06Rik	0.0131765	6.4138819
103261_at	Gspt2	0.0030388	6.5908211
103263_at	Epc1	0.0000023	7.8806959
103269_f_at	Zfp125	0.0016284	7.0273613
103270_at	Gtse1	0.0006892	7.6105688
103273_s_at	Abcc8	0.0105380	4.2915032
103275_at	Atp6v0a1	0.0001647	6.8275097
103276_at	Large	0.0000210	5.9882673
103277_s_at	Tnrc11	0.0000193	8.4110550
103281_at	Cd2ap	0.0015268	5.9074441
103285_at	Mbd4	0.0002759	6.5991359
103295_at	9130229H14Rik	0.0043501	7.1344063
103300_at	Abcb7	0.0004548	7.8392732
103301_i_at	Sox3	0.0120958	7.8746582
103309_at	Frap1	0.0000457	7.6454484
103312_f_at	2610101J03Rik	0.0000318	7.6364709
103315_at	3110054G10Rik	0.0000210	8.0426756
103317_at	Coch	0.0000024	4.1857414
103321_at	B230364F10	0.0002058	6.5765088
103322_at	Rpia	0.0030388	7.2862100
103327_at	Prrx2	0.0112863	6.7180415
103330_at	Spnr	0.0013419	8.1305575
103340_at	Rhced	0.0086076	4.3812336
103341_at	Ctps	0.0000828	9.9185589
103342_at	Eed	0.0000023	8.2303159
103343_at	5430432M24Rik	0.0086090	5.6224958
103345_at	Spna2	0.0025288	8.6790488
103346_at	Clk2	0.0005231	8.3921243
103348_at	1110018F06Rik	0.0018496	7.1309863
103350_at	Psmd7	0.0000008	9.8868658
103354_at	Mrps31	0.0000013	8.1606493
103357_at	Slc2a2	0.0001044	7.6510836
103359_at	LOC228019	0.0000053	7.1435097
103363_at	Tbn	0.0000766	6.7841579
103364_f_at	5730494M16Rik	0.0019703	8.2494673
103370_at	Lin7c	0.0000075	8.9548113
103371_at	H2−Ke4	0.0017358	9.5555547
103375_at	2810406K24Rik	0.0000048	7.7130868
103377_at	Lrp2	0.0001774	8.2558427
103379_at	Fbxo3	0.0000170	6.2991532
103381_at	1810024J13Rik	0.0001217	8.6446161
103388_at	P42pop−pending	0.0162695	8.0529470
103392_at	Adcy7	0.0125493	4.9412468

103393_at	Pspc1	0.0000895	7.2883154
103397_at	Hrb	0.0000040	6.9500420
103400_at	AI316828	0.0041010	8.4130618
103404_at	1110033A15Rik	0.0002385	8.8533570
103406_at	Mbdin−pending	0.0000228	9.2180148
103412_at	3732412D22Rik	0.0065121	5.4925473
103413_at	2310058A11Rik	0.0009656	7.9316244
103414_at	Stk19−ps1	0.0162695	7.2438671
103415_at	Wrnip1	0.0000274	6.7388424
103416_at	Mapk6	0.0000026	9.5859883
103418_at	Rfc4	0.0088930	7.4784279
103420_at	Emd	0.0000210	8.0416241
103421_at	Sdfr2	0.0005413	6.5950932
103428_at	Pold3−pending	0.0003678	8.4007978
103429_i_at	AL024210	0.0000055	6.9720773
103436_at	Gtpbp1	0.0000028	7.5577442
103440_at	Gabpa	0.0000018	7.1060808
103441_at	Csnk2a1	0.0007895	7.3848881
103442_at	LOC216820	0.0011775	7.7572873
103444_at	E130315B21Rik	0.0000044	6.5284549
103455_at	AI414418	0.0000034	5.2701742
103457_at	Rev3l	0.0068924	7.0164269
103459_at	Ermelin−pending	0.0001417	6.4781185
103460_at	Rtp801−pending	0.0000090	8.7336687
103463_at	−−−	0.0088470	0.8381201
103466_at		0.0046129	6.9500518
103475_s_at	Ate1	0.0142988	4.5303107
103477_at	Cdx1	0.0000009	7.4830960
103479_at	D930033N23Rik	0.0125493	6.6404025
103481_at	8430426H19Rik	0.0000075	7.7468870
103485_at	BC030046	0.0000967	7.2537437
103496_at	Dri1	0.0122205	5.9393203
103497_at	BC025546	0.0000053	5.6556799
103498_at	Gcgr	0.0036411	5.6339313
103500_at	Orc4l	0.0077054	6.3720940
103501_at	Pura	0.0005607	6.5372041
103502_at	AA409510	0.0001313	6.4644952
103503_at	Plcg2	0.0000766	6.3755850
103506_f_at	Dsc2	0.0039805	5.4082235
103508_at	5830443C21Rik	0.0006436	5.4092714
103524_at	Cdan1	0.0000558	7.0417662
103525_at	2810036L13Rik	0.0000063	9.1894326
103530_at	Fancg	0.0022336	4.9592914
103532_at	Eomes	0.0000019	5.2078256
103537_at	Eif2ak3	0.0090879	5.1566643
103538_at	Tbx3	0.0000210	5.1935151
103541_at	Tcte2	0.0162671	4.2868870
103545_at	2610019E17Rik	0.0125493	5.8089111
103551_at	Pepp2−pending	0.0000193	7.6494523
103552_at	AI481320	0.0004239	5.4880986
103553_at	2410041F14Rik	0.0003949	7.4003201
103555_at	2010012F05Rik	0.0139359	9.3780157
103557_at	5730409F24Rik	0.0014318	7.2987628
103559_at	Prkaca	0.0000011	8.3944848
103560_at	2210402C18Rik	0.0008446	5.9053476
103563_at	4930534K13Rik	0.0038649	6.2867750
103565_at	1810009A15Rik	0.0008446	9.3282052
103567_at	2010313D22Rik	0.0000735	7.4036516
103573_at	Pip5k1a	0.0119030	4.5675718
103574_at	4833406P10Rik	0.0139359	5.1429583
103578_at	Tom1	0.0000053	7.7163883
103581_at	Cte1	0.0000318	6.5579386
103584_at	5830471E12Rik	0.0000040	8.3354432
103585_at	Spg4	0.0000126	6.5647170
103591_at	Taf6	0.0132246	5.7397888
103598_at	Dhx9	0.0000269	8.5506937
103605_g_at	Rgs19	0.0032273	6.8449782
103606_r_at	Rgs19	0.0000228	7.7544363
103617_at	Daf1	0.0079209	4.1483495
103619_at	Cyb5m−pending	0.0000023	7.6940797
103620_s_at	Smn	0.0000111	9.0449783
103625_at	Afg3l1	0.0000008	8.1850876
103628_at	Lef1	0.0020982	8.5247274
103630_at	Lars	0.0013419	8.9171245
103631_at	2810407K09Rik	0.0034293	6.2818275
103635_at	2610025M23Rik	0.0041002	5.9178473
103637_at	Naga	0.0132266	6.7175197
103639_at	Ifit2	0.0026894	3.4958782
103642_at	G3bp−pending	0.0000559	10.7497394
103654_at	Nsbp1	0.0000345	8.0381020
103657_i_at	Mtf2	0.0119235	7.7414152
103658_r_at	Mtf2	0.0033269	4.7934251
103664_r_at	2810452K22Rik	0.0101375	6.6814239
103665_at	Elovl6	0.0007895	7.0289987
103667_at		0.0000060	8.6112061
103672_at	2410141M05Rik	0.0009032	8.5619928
103674_f_at	Eif2s3y	0.0000116	6.4037834

103678_at	9330180L10Rik	0.0000058	8.6994222
103680_at	1300011P19Rik	0.0014504	7.3107020
103681_at	Atp6v0a2	0.0046129	7.9557922
103682_at	Rmp−pending	0.0000516	6.4528493
103683_at	Dhodh	0.0000008	7.7040834
103694_at	LOC227699	0.0000075	8.1208688
103695_f_at	C330007P06Rik	0.0000413	8.2271217
103696_r_at	C330007P06Rik	0.0109853	2.8147420
103697_at	AW061234	0.0086076	6.4706593
103701_at	D16Ertd480e	0.0007375	6.7347320
103704_at	2010305K11Rik	0.0000116	6.3747258
103712_at	R3hdm	0.0000345	7.5160571
103713_at	Usp9x	0.0002058	7.9320366
103716_at	4933409L06Rik	0.0020982	6.6397652
103717_at	Wwp2−pending	0.0000155	6.4026274
103720_at	Rest	0.0023770	7.6513424
103727_at	Hrb	0.0154493	6.4181181
103731_at	Tsc1	0.0008446	6.3904769
103736_at	2500002E12Rik	0.0005229	6.3677806
103738_at	MGC47434	0.0090924	7.8450826
103744_at	A930014C21Rik	0.0115892	5.4983215
103745_at	Snx13−pending	0.0013419	4.8319037
103746_at	D11Ertd530e	0.0001647	6.6753801
103748_at	4933407C03Rik	0.0004548	6.4385832
103753_at	3110065C23Rik	0.0070489	8.3472574
103754_at	1100001F19Rik	0.0015272	7.6542781
103755_at	Sh3d19	0.0135751	5.9998779
103756_at	BC023829	0.0000374	8.3211889
103760_at	2810004N23Rik	0.0046129	6.6416407
103761_at	Crtr1−pending	0.0000258	6.4344351
103762_at	C76800	0.0000116	7.9930469
103763_at	Ash1l	0.0061541	6.0321031
103766_at	Sema5a	0.0009023	4.6838904
103768_at	6720480F16Rik	0.0000476	6.9503759
103771_at	1110061N23Rik	0.0006436	7.8501835
103773_at	1110020K19Rik	0.0000605	6.9796723
103774_at	9130019P20Rik	0.0000967	6.3187779
103776_at	AI593864	0.0001364	5.6626408
103779_at	Mkl1	0.0032287	6.2768632
103780_at	1700021F05Rik	0.0000035	5.1307276
103781_at	Stx4a	0.0031318	7.0797732
103783_at	Xpr1	0.0004239	8.0422549
103784_at		0.0007377	5.9818867
103786_at	D5Ertd135e	0.0005607	4.8704367
103790_at	D16Ertd36e	0.0003484	8.2507213
103791_at	Narg1	0.0008446	7.2759807
103796_at	6230400I06Rik	0.0000516	6.4292634
103797_at	Cdc7	0.0000047	6.6303795
103799_at	Mtmr9	0.0119030	6.5903729
103805_at	Nbn	0.0008341	6.2531635
103806_at	Lrp5	0.0000895	5.0139536
103807_at	Wiz	0.0000345	7.6326591
103808_at	Golga5	0.0001647	5.7984936
103814_at	Pex11b	0.0012165	6.8064231
103818_at	Slc7a7	0.0000210	8.3287167
103821_at	Cdc6	0.0046129	8.7294759
103823_at	Top3b	0.0028592	7.2638075
103828_at	Vps52	0.0000069	6.7165823
103830_at	Snai1	0.0000292	7.4116079
103832_at	Tfip11	0.0015272	5.4325225
103835_f_at	Hpcal1	0.0059813	7.7476694
103836_at	Wbp4	0.0065133	6.1873123
103840_at	Rad17	0.0001417	6.4382079
103841_at	Zfp64	0.0000765	6.1757908
103842_at	Ddx3y	0.0000019	6.1073090
103843_at	Gnao	0.0154561	6.8278619
103848_at	Crkl	0.0001911	7.3342344
103852_at	Osrf−pending	0.0046129	6.6852713
103853_at	Grwd1	0.0027725	7.7829515
103854_at	Arl2	0.0002759	7.3037493
103858_at	Eral1	0.0000017	7.2429802
103859_at	Eral1	0.0007377	6.5170889
103863_at	5630401J11Rik	0.0007895	8.9285393
103868_at	Nufip1	0.0001002	5.6595160
103869_at	Thbs3	0.0154561	7.4320235
103873_i_at	2310015N07Rik	0.0004733	7.5761814
103875_at	Ngrn−pending	0.0000040	7.2485572
103879_at	BC024806	0.0001911	7.3876094
103881_at	Sid6306−pending	0.0000655	7.1448191
103885_at	1500019O16Rik	0.0000967	8.0633870
103888_at	Rbpms	0.0000015	9.5463463
103889_at	Ira1−pending	0.0000009	7.1349865
103891_i_at	Ell2−pending	0.0001968	6.6946908
103892_r_at	Ell2−pending	0.0004044	4.3716419
103893_at	6030410I24Rik	0.0051821	7.8402570
103894_at	Shkbp1	0.0004548	8.4023593
103895_at	AW549877	0.0001528	6.8219134

103900_at	Centg3	0.0004877	7.1979735
103901_at	4930451A13Rik	0.0026074	5.4058528
103908_at	C330016H24Rik	0.0000967	6.8936883
103909_at	Ccs	0.0017914	6.1339156
103912_at	2410091N08Rik	0.0054898	6.8850458
103913_at	Sec61a2−pending	0.0054898	8.2972919
103914_at	1110033E03Rik	0.0000132	7.0523633
103922_f_at	1500005G05Rik	0.0000023	7.0853673
103923_at	Tm7sf1	0.0112863	5.6616476
103924_at	D8Ertd319e	0.0000069	8.6422794
103926_at	Eif4g1	0.0000021	7.9492980
103927_at	−−−	0.0002124	7.9554292
103930_at	N4bp1−pending	0.0000011	7.5498084
103932_at	Gm83	0.0000069	8.4257497
103939_at	2610509I15Rik	0.0023759	4.1214001
103944_at	Rad51l1	0.0063268	4.9529280
103945_at	Cfc1	0.0023770	6.2201578
103947_at	Ppp6c	0.0000708	7.0091557
103949_at	Ihh	0.0038649	5.8821169
103950_at	Aqr	0.0074951	4.7158211
103953_at	Sec22l1	0.0002384	6.6382122
103957_at	Trfr	0.0068888	6.0441556
103958_g_at	Trfr	0.0003425	9.0767192
103959_at	Phf13	0.0005607	6.7296048
103964_at	Esrra	0.0031318	6.8553591
103969_at	Mll5	0.0000048	6.8708190
103981_at	U2af1−rs2	0.0000422	7.0102749
103989_at	4432417F03Rik	0.0009337	6.1063106
103990_at	Fosb	0.0000063	6.8949784
103991_at	Akp5	0.0098654	5.4437556
103994_at	Eif2c2	0.0000008	9.2570878
103995_at	Fgfbp1	0.0005050	1.9740147
103997_at	Epor	0.0038649	6.0630390
104002_at	Zfp275	0.0036411	6.6126268
104006_at	Eps15	0.0006432	4.7888872
104007_at	Slc25a15	0.0000116	7.6928392
104015_at	Metap1	0.0000044	8.8144439
104017_at	Facl4	0.0086090	4.3249516
104019_at	Ubl4	0.0000317	5.6494420
104022_at	2810453H10Rik	0.0046129	7.1570208
104031_at	Ptch	0.0011025	6.2926946
104033_at	Mgea6	0.0000069	8.3463548
104035_at	A930019L04Rik	0.0004239	6.6663888
104036_at	Dpp7	0.0011775	7.1091126
104037_at	BC016198	0.0000405	5.0561463
104038_at	4933434E20Rik	0.0058118	7.7647498
104041_at	1810009A16Rik	0.0000018	9.5195444
104044_at	1300006N24Rik	0.0000318	7.6866807
104046_at	Arih1	0.0000228	7.8483151
104048_at	Cars	0.0065145	6.9904955
104050_at	Zfr	0.0000008	8.7842367
104052_at	1810011K17Rik	0.0000293	8.1855103
104056_at	D16Bwg1543e	0.0007895	7.9095002
104057_at	Grpel1	0.0003678	7.4812030
104058_at	1110018J12Rik	0.0000439	8.0894143
104059_at	5830451P18Rik	0.0000193	7.9837239
104063_at	Srcasm	0.0065145	6.9764934
104067_at	Tubgcp3	0.0000138	7.0931123
104069_at	Scr59−pending	0.0000063	9.9681017
104070_at	Pcaf	0.0002860	3.5943926
104071_at	Kpnb2b−pending	0.0000008	8.8443861
104074_at	0610025L06Rik	0.0013419	6.6021289
104077_at	1110049G11Rik	0.0000034	7.7040019
104078_g_at	1110049G11Rik	0.0000082	7.8116648
104079_at	Dyrk1a	0.0028592	7.3008421
104080_at	Pdap1	0.0001646	9.8875416
104082_at	Rab12	0.0000053	6.8836450
104086_at	1200014D15Rik	0.0014784	3.6694193
104089_at	2810026P18Rik	0.0000026	7.9391074
104090_at	Cdc23	0.0072900	7.0638278
104091_at	9430079M16Rik	0.0162648	7.9370127
104092_at	Usp31	0.0002058	8.4568871
104094_at	Ril−pending	0.0139137	6.2657382
104096_at	Orc4l	0.0004878	7.1059930
104097_at	Bub1	0.0000037	8.4370496
104101_at	Slc9a8	0.0000559	7.3132853
104102_at	Prss25	0.0000258	7.1512806
104105_at	Xpo6	0.0001313	9.1110560
104106_at	Sbno1	0.0000069	6.3175154
104108_at	Rab6ip1	0.0009032	6.7095434
104109_at	Fbxo21	0.0115892	7.6715689
104110_at	BC031407	0.0000008	9.7114021
104115_at	Psme4	0.0000150	8.7725203
104117_at	2310040A13Rik	0.0070875	5.2647631
104118_at	2810037C14Rik	0.0000009	8.4553082
104119_at	AW060714	0.0000238	6.8377297
104120_at	5330431N19Rik	0.0037507	7.3612852

104125_at	Rnf12	0.0001774	7.7586340
104126_at	Cstf2t−pending	0.0009656	7.6805256
104128_at	AW556797	0.0009656	7.8974134
104132_at	Noc4	0.0000008	8.9677860
104135_at	Arl3	0.0010320	7.0788269
104138_at	Pafah2	0.0010664	5.4894947
104139_at	P4ha1	0.0002058	7.7106639
104141_at	D15Wsu75e	0.0013419	7.7035186
104142_at	2810013E07Rik	0.0000967	6.7485454
104145_at	Tcof1	0.0001217	8.9147944
104150_at	2810008P14Rik	0.0022336	7.4518241
104153_at	Ivd	0.0058140	6.6878612
104154_at	Trp53	0.0034293	9.1400760
104155_f_at	Atf3	0.0072887	5.5974557
104156_r_at	Atf3	0.0139338	5.7224019
104158_at	Skiip	0.0000019	7.8529202
104161_at	Cpsf2	0.0001127	6.4239381
104163_at	Ipo8	0.0000126	7.8404472
104164_at	1300019N10Rik	0.0101375	7.5819297
104173_at	Ms4a1	0.0101325	5.0362844
104176_at	5530400K14Rik	0.0022336	5.5693634
104179_at	AI788669	0.0000019	6.0574434
104183_at	D2Ertd435e	0.0018496	7.0721181
104189_at	Traf6	0.0000116	6.7224443
104192_at	Tpr	0.0001774	5.4291307
104193_at	2810485I05Rik	0.0000736	7.9779340
104196_at	6330503C17Rik	0.0106982	7.6397923
104200_at	Slc5a6	0.0034279	5.9883155
104202_at	hephaestin	0.0002058	5.4276967
104212_at	Lrpprc	0.0000331	9.1763845
104214_at	Slc7a8	0.0072900	5.9560040
104218_s_at	2310032M22Rik	0.0001527	4.7936082
104220_at	Madh6	0.0001171	7.6303362
104221_at	Slc7a5	0.0000023	9.1078467
104225_at		0.0086090	8.7493397
104231_at		0.0004878	8.7430842
104234_at	Mrps25	0.0040931	6.8965199
104237_at	2700061N24Rik	0.0032280	6.5786303
104241_at	2210408E11Rik	0.0008446	7.7123429
104242_f_at	4930578F06Rik	0.0011775	7.7302813
104244_at	AU024026	0.0146787	7.0512125
104246_at	LOC228790	0.0006436	8.7745192
104247_at	1500041L05Rik	0.0004390	7.2190990
104248_at	0610038P07Rik	0.0018496	8.8805529
104249_g_at	0610038P07Rik	0.0000075	8.7881763
104259_at	Cbx5	0.0000009	8.0142458
104260_at	AW112037	0.0006008	6.8256566
104261_at	LOC228790	0.0003949	7.0478952
104263_at	9330177P20Rik	0.0010320	5.0453027
104267_at	Slc23a2	0.0012569	6.2850482
104270_at	Adrbk1	0.0002759	6.5473627
104272_s_at	Map3k4	0.0000013	7.3303797
104275_g_at	Trp53	0.0006892	8.5048821
104277_at	Alg2	0.0061541	8.0680305
104279_at	1810060D16Rik	0.0004878	10.0326015
104282_at	AW112037	0.0096031	4.1385971
104286_at	Slc38a4	0.0000008	8.6361553
104287_at	Smt3ip1−pending	0.0001911	9.6098346
104288_at	Cul4a	0.0000009	8.1911071
104290_at	Casp8ap2	0.0000012	8.0383028
104293_at	1810045K06Rik	0.0162695	8.6768219
104296_at	1110031M08Rik	0.0011025	7.4153895
104297_at	Ipo11	0.0000210	8.0162706
104298_at	2310044G17Rik	0.0004548	6.6124332
104300_at	Iqgap1	0.0051821	8.5586937
104301_at	2410018G20Rik	0.0154538	7.4819557
104302_f_at	1810029F08Rik	0.0024645	6.5464057
104303_i_at	1500004O14Rik	0.0005149	8.2740432
104305_at	Rarsl	0.0000034	8.5839062
104306_at	Dpp3	0.0000028	8.6257566
104312_at	1110013B16Rik	0.0009646	6.6887005
104313_at	2610020G18Rik	0.0030388	8.9105459
104315_at	B230365D05Rik	0.0000106	7.8800166
104316_at	Gna13	0.0016284	5.8738416
104317_at	Cse1l	0.0043501	9.5343976
104322_at	Ckap2	0.0000016	7.9631549
104327_at	AI265322	0.0000163	7.7244748
104330_g_at	Smarcf1	0.0003949	7.6581595
104331_at	Smarcf1	0.0000248	10.3928714
104332_at	1190005P08Rik	0.0015272	8.1622155
104337_f_at	1200008D14Rik	0.0000708	7.7349398
104338_r_at	1200008D14Rik	0.0019694	6.4796769
104339_at	Pygo2−pending	0.0011775	7.9861225
104341_at	2310014H01Rik	0.0000210	8.5105702
104342_i_at	Pla2g12	0.0045806	5.6604101
104343_f_at	Pla2g12	0.0000077	7.6653657
104349_at	2410133M08Rik	0.0001911	7.7726073

104350_at	Shc1	0.0004878	7.5757552
104352_at	Brd4	0.0000476	8.9024221
104358_at	D10Ertd610e	0.0006658	7.9094056
104362_at	B230113M03Rik	0.0093423	7.8566962
104364_at	Mapkapk5	0.0002216	7.5507854
104366_at	BC039093	0.0000075	7.5716761
104367_at	Eps15	0.0000058	7.0932773
104372_at	0910001L24Rik	0.0024513	6.5156900
104373_at	Aprin	0.0003188	5.7000728
104375_at	Spock2	0.0083740	6.8318011
104376_at	Hdac5	0.0036411	7.3040837
104378_at	MGC68232	0.0001416	5.3245654
104383_at	Crmp1	0.0154561	7.9242132
104386_f_at	Itgav	0.0150607	8.4516299
104389_at	1700017B05Rik	0.0000422	7.8844552
104390_at	Anp32a	0.0023094	9.4917444
104391_s_at	Clcn7	0.0006008	7.2806705
104396_at	Znrf2	0.0016276	5.6708491
104399_at	Cstf2	0.0000559	8.2889976
104402_at	AI426938	0.0001417	6.2067896
104403_at	2700078E11Rik	0.0032280	7.5162694
104404_at	5730533P17Rik	0.0001646	6.1289933
104405_at		0.0054898	5.7931590
104408_s_at	Sox18	0.0077040	4.9697585
104410_at	Midn	0.0009656	8.9373609
104412_at	Gnai1	0.0081474	6.0406832
104413_at	AI834976	0.0002777	6.5092046
104419_at	F730017H24Rik	0.0001774	5.9258795
104423_at	2810047L02Rik	0.0003187	7.3808206
104427_at	Mtmr13	0.0011775	7.0821150
104432_at	Arhn	0.0005607	6.0804140
104433_at	1110012E06Rik	0.0023770	7.4575977
104437_at	Zfp30	0.0001170	4.5486340
104444_at	9430098E02Rik	0.0051821	5.6558782
104446_f_at	4933428G09Rik	0.0065145	5.7846295
104448_at	Mttp	0.0000828	5.1299461
104453_at	2310079P12Rik	0.0013416	6.9736690
104454_at	Pms2	0.0000178	6.5562681
104463_at	3830408P06Rik	0.0001313	8.1396592
104464_s_at	Kdelr3	0.0002759	6.7226648
104468_at	Incenp	0.0000031	6.4948544
104473_at	AI838661	0.0139338	7.7191921
104475_at	6030458H05	0.0000082	7.1180350
104480_at	Dsg2	0.0063303	7.6022256
104486_at	A2m	0.0086090	4.2921152
104494_at	4921515A04Rik	0.0115856	5.3983292
104503_at	6030436C20Rik	0.0010320	6.7074189
104509_at	Ch25h	0.0016043	3.6793709
104513_at	2410004N09Rik	0.0001528	8.1318828
104523_at	E430036I04Rik	0.0068924	4.3008793
104527_at	Rad51	0.0001774	8.2029761
104531_at	Prkcd	0.0005607	7.8283710
104532_at	2610100K07Rik	0.0004547	8.0873235
104533_at	Pim1	0.0166877	8.1981223
104535_at	Yme1l1	0.0046129	6.1363308
104536_at	Madh2	0.0000036	7.0457862
104537_at	0610042C05Rik	0.0000012	5.6870852
104538_at	Ptgis	0.0000075	7.3952810
104545_at	Csnk2a1	0.0004238	6.8697825
104547_at	Dhfr	0.0011025	5.8956387
104549_at	Aqr	0.0000008	8.1907413
104550_at	Cyp2s1	0.0048900	5.4043424
104554_at	Nr2f6	0.0000210	8.2492263
104557_at	Pitpnb	0.0000344	7.8934485
104558_at	5730409O11	0.0015272	6.8744833
104559_at	BC003940	0.0068924	8.7636548
104560_at	Mrs3/4−pending	0.0158559	7.1143897
104561_at	2700084A09Rik	0.0000766	7.2541485
104562_at	5730403M16Rik	0.0143009	6.1103324
104567_at	3110052F15Rik	0.0000015	6.4477017
104568_at	Mll	0.0000318	6.4835914
104569_at	Mll	0.0000185	7.4564207
104572_at	MGC38585	0.0006436	6.7227819
104573_at	1110025L05Rik	0.0001647	8.7932166
104574_at	5730453I16Rik	0.0000405	8.0809421
104576_at	Ski	0.0017354	8.1270004
104578_f_at	Actn1	0.0000069	9.5877679
104579_r_at	Actn1	0.0011025	7.4516474
104581_at	Zdhhc6	0.0040994	3.7695884
104582_g_at	Zdhhc6	0.0001417	7.4981048
104583_at	Zdhhc6	0.0146787	7.9440031
104586_at	5730507C05Rik	0.0036411	7.9088820
104589_at	Rmp−pending	0.0000009	7.9808940
104592_i_at	Mef2c	0.0067288	1.2280955
104593_at	1700023O11Rik	0.0038649	6.1600301
104595_at	Stag2	0.0002385	7.3468356
104598_at	Dusp1	0.0000015	7.1985602

104600_at	1300019H17Rik	0.0000389	6.6852684
104601_at	Thbd	0.0142981	2.7262332
104602_at	Waspip	0.0032253	4.9044003
104604_at	Zfp96	0.0009656	5.3192138
104608_at		0.0150607	4.7853156
104609_at	AI465155	0.0003949	7.1974496
104610_at	LOC226169	0.0001313	7.4780631
104611_at	Wdr26	0.0061552	6.6100618
104612_g_at	Wdr26	0.0001911	6.5932510
104614_at	Gpc1	0.0002208	8.7498236
104616_g_at	Galt	0.0041010	6.8313680
104618_at	Rbbp9	0.0015272	6.3049573
104620_at	2010300G19Rik	0.0000058	5.9954118
104623_at	Tle3	0.0001044	8.8141192
104624_at	MGC38812	0.0000537	8.8853472
104625_at	Dnajb6	0.0000011	7.4421689
104626_at	Cklfsf8	0.0081474	7.4778658
104627_at	Cds2	0.0000828	7.6143721
104633_at	Dab2	0.0000018	8.6233389
104634_at	Lims1	0.0000012	7.7293294
104639_i_at	4930553M18Rik	0.0000130	9.0793380
104640_f_at	4930553M18Rik	0.0001246	9.4534123
104643_at	BC037006	0.0077080	7.2361623
104644_at	Kif4	0.0000121	7.3752329
104645_at	Klf7	0.0146765	5.7653714
104648_at	Pacs1	0.0051821	6.0476693
104651_at	C330035N22Rik	0.0154538	6.3811120
104661_at		0.0146787	6.2954215
104663_at	Pip5k1b	0.0001774	7.2566848
104672_at	Frzb	0.0000144	7.0637699
104681_at	Diap1	0.0000011	7.3075491
104682_at	Tuba8	0.0052301	7.0632959
104683_at	Ankrd17	0.0000026	7.9789681
104688_at	Map4k1	0.0119030	5.9834622
104690_at	Polm	0.0021644	7.4221372
104698_at	Gata6	0.0019703	7.1748547
104699_at	5330434F23Rik	0.0096870	6.3676218
104701_at	Bhlhb2	0.0005607	2.6644183
104706_at	Pex7	0.0022336	5.6524200
104709_at	Sec23a	0.0038649	8.2291127
104711_at	Vps4a	0.0000126	7.4117737
104712_at	Myc	0.0001178	5.9439746
104713_at	2410002M20Rik	0.0004239	8.2619914
104715_at	Ubap2	0.0003425	9.7863152
104717_at	D5Ertd689e	0.0000008	9.2270880
104719_at	Slc12a7	0.0002216	7.9857115
104725_at	Arhq	0.0065133	5.1832967
104730_at	4833432B22Rik	0.0001647	7.9025753
104735_at	AW538430	0.0088914	4.0372032
104738_at	Zrf2	0.0000010	9.2303698
104740_at	D1Ertd251e	0.0000293	7.1759819
104744_at	2610319K07Rik	0.0000495	8.0597526
104745_at	Arl6ip2	0.0000069	7.9896139
104749_at	Rnf20	0.0002058	6.1125460
104754_at	Sfrs4	0.0000708	8.3427502
104758_at	2410001E19Rik	0.0000051	6.7033986
104760_at	Ifrd2	0.0068924	8.9215457
104761_at	Antxr2	0.0125474	5.3862309
104766_at	Nola1	0.0000012	10.6418618
160064_at	Stx7	0.0000053	7.5974655
160065_s_at	Csrp1	0.0101375	7.2419619
160066_at	Limd1	0.0083740	6.8495595
160067_at	2310057D15Rik	0.0001313	7.3867743
160068_at	Sap30	0.0000010	7.6491868
160069_at	Gmnn	0.0000057	8.0880799
160071_at	Rnasep2−pending	0.0007236	7.5192546
160072_at	Rfc3	0.0002966	6.8824978
160074_at	Ddc	0.0104128	6.6317995
160076_at	Mtx2	0.0001128	8.3093743
160079_i_at	Wwp4−pending	0.0005374	6.2374061
160081_at	Rpl44	0.0014318	8.4020896
160082_s_at	Arf4	0.0000248	9.2700693
160083_at	Lpl	0.0048900	4.7740820
160084_at	Odc	0.0000040	9.1511252
160089_at	Lamp1	0.0000012	11.1418078
160090_f_at	Aldo1	0.0000895	12.0220169
160091_at	Pgam1	0.0000034	11.6360801
160092_at	Ifrd1	0.0000028	7.0581780
160094_at	Arpc4	0.0022336	9.1281373
160096_at	Spop	0.0015272	7.7817904
160098_s_at	Cryab	0.0000075	7.7634593
160101_at	Hmox1	0.0009656	7.8689401
160102_at	Cct8	0.0000013	9.9407637
160103_at	Axot	0.0000053	8.2182579
160107_at	Hprt	0.0000008	9.5263505
160110_at	Wwp2−pending	0.0000476	7.7220070
160111_at	1500010B24Rik	0.0017358	7.6227937

160112_at	1190006E07Rik	0.0001217	5.2084117
160113_at	Brap	0.0003188	7.0202620
160114_at	Gdi3	0.0000010	10.3447562
160121_at	Galk2	0.0014314	6.6871663
160123_at	Php14−pending	0.0013419	9.2004089
160124_r_at	Atp6v1c1	0.0000796	7.5855613
160125_at	D11Ertd497e	0.0000374	7.0936486
160126_at	Map2k1ip1	0.0000106	7.1803797
160127_at	Ccng1	0.0000193	5.4770137
160129_at	Eef1d	0.0000150	10.2080695
160130_at	Wdr26	0.0000016	6.0507105
160135_at	D16Ertd502e	0.0081460	6.2666979
160140_at	Tbce	0.0119030	6.1159688
160141_r_at	Sfrs1	0.0000345	5.4943376
160146_r_at	Polr2c	0.0000058	7.1657022
160150_f_at	Cnn3	0.0054898	10.0176735
160151_i_at	1200009B18Rik	0.0001716	5.7391039
160153_at	Dnajc7	0.0002966	8.3025190
160156_at	0910001A06Rik	0.0000075	7.0114847
160158_at	Gpc3	0.0041010	10.5294287
160159_at	Ccnb1	0.0000011	9.4374902
160160_at	Dedd	0.0009656	4.9047912
160161_at	Gtrgeo22	0.0146787	7.1339845
160163_at	AA415817	0.0043501	7.5476247
160164_at	Ube2v1	0.0008446	9.5867211
160166_r_at	2810409H07Rik	0.0018289	6.8289570
160167_at	Nup62	0.0029472	9.5748422
160169_at	Plaa	0.0000020	7.2140660
160172_at	Gtl2	0.0000248	9.5300049
160174_at	0610041O14Rik	0.0000248	7.6872776
160176_at	Hirip5	0.0002759	6.4560372
160177_at	2610203K23Rik	0.0000012	8.9413811
160180_at	Canx	0.0038649	6.2888225
160182_at	Sfrs6	0.0000193	10.3434785
160183_f_at	3930401E15Rik	0.0000516	9.2642644
160184_at	1200007D18Rik	0.0068924	7.1380463
160188_at	4933436C10Rik	0.0002058	8.2372902
160192_at	Rbmxrt	0.0000011	9.6380005
160196_at	Smap1	0.0000317	7.0152805
160197_at	1110058B13Rik	0.0021644	7.6218782
160199_at	Hnrpc	0.0000034	7.6554964
160200_at	3230401D17Rik	0.0003810	6.0354517
160202_at	Atp6ip2	0.0005050	6.4225220
160203_at	5330419I01Rik	0.0000012	9.0497343
160204_at	3110013H01Rik	0.0018492	4.5298615
160205_f_at	Rnf11	0.0041010	7.3505187
160206_at	2610510H01Rik	0.0009656	7.2996839
160208_at	1810061H24Rik	0.0000008	9.4432719
160209_at	Rnf44	0.0032280	8.8337630
160218_at	2310056P07Rik	0.0000021	8.5779763
160220_at	Zfp110	0.0000090	5.9006009
160221_at	Lgals2	0.0012572	6.9833974
160223_at	2610511O17Rik	0.0001617	6.6492856
160225_at	Gtf2b	0.0002134	8.1283140
160226_at	Gfm	0.0028592	7.1521228
160227_s_at	Bysl	0.0002058	9.1221662
160228_at	1110019C08Rik	0.0001417	6.5972756
160230_at		0.0072900	9.3420262
160231_at	0610012A19Rik	0.0000008	8.6247045
160233_at	Ak3l	0.0007377	5.4214572
160234_at	Usp1	0.0000009	8.6284450
160236_at	9630044O09Rik	0.0011025	5.2896371
160237_at	Ndufa6	0.0012658	7.6012117
160239_at	2400006A19Rik	0.0000009	9.8003478
160242_at	B430214A04Rik	0.0011775	6.6462250
160244_at	Fem1a	0.0009032	6.5235558
160246_at	E130304C20Rik	0.0000116	6.0612866
160247_at	Ube2v2	0.0008164	6.6065199
160248_at	D3Ertd330e	0.0101375	9.1572038
160249_at	Tpd52	0.0001982	6.8185192
160251_at	Ierepo4−pending	0.0000021	8.9704831
160252_at	Cltc	0.0000019	10.5311557
160255_at	1110004P15Rik	0.0002215	7.9604390
160257_at	Fkbp1a	0.0000359	10.4469071
160262_at	Mtch2	0.0000021	9.3141698
160264_s_at	1500036F01Rik	0.0014504	6.2923511
160265_at	Eif5	0.0000150	8.3398218
160266_r_at	1110064N10Rik	0.0001911	8.9850496
160267_at	0610009E20Rik	0.0043541	7.4646116
160268_at	1110061A19Rik	0.0000248	7.9356714
160269_at	Gfer	0.0054898	8.5534426
160270_at	Lman1	0.0001044	8.7759501
160271_at	0610007C21Rik	0.0050332	7.1855920
160272_at	Cbx3	0.0132266	9.5639754
160273_at		0.0000008	9.5783539
160277_at	Stx12	0.0004548	7.6050101
160278_at	2810428I15Rik	0.0000009	7.9812057

160279_at	AA408582	0.0000150	9.1707994
160281_at	2700023B17Rik	0.0000210	8.5763281
160282_at	4931406I20Rik	0.0001313	8.9743169
160283_at	2410005K20Rik	0.0000193	10.4439238
160285_at	Dhx40	0.0039805	6.9965703
160286_at	Dek	0.0000036	9.7396743
160287_at	Map1lc3	0.0000018	8.1863335
160288_at	Map1lc3	0.0000058	8.2851712
160289_s_at	Dlst	0.0000374	7.8293709
160290_at	Ide	0.0001774	6.9758207
160293_at	2700038L12Rik	0.0008446	9.0149368
160295_at	Slc25a11	0.0009656	8.0897550
160296_at	Wsb2−pending	0.0000558	6.0031188
160297_at	MGC36453	0.0000210	8.1615187
160299_at	Rwdd1	0.0005607	8.7919318
160300_at	Tff1	0.0068924	7.8056234
160301_at	Riok3	0.0001774	7.3706782
160305_at	Psmd11	0.0000026	8.7854368
160308_at	Msn	0.0000048	8.1040769
160309_at	Map3k7ip2	0.0000015	8.9048758
160310_at	D19Bwg1357e	0.0000090	9.2812857
160313_at	A730098D12Rik	0.0000163	8.7906620
160314_at	2010317E03Rik	0.0000248	10.3094840
160317_at	Rab34	0.0036411	7.4378345
160318_at	Stub1	0.0068924	9.3017177
160319_at	Sparcl1	0.0005229	4.2927816
160320_at	Sorbs1	0.0006217	5.7987325
160321_at	Zfp216	0.0000063	8.3254913
160322_at	1190006A08Rik	0.0139359	7.8881907
160323_at	Sra1	0.0104128	7.5392069
160326_at	Cdv3	0.0000015	9.8015987
160327_at	Dctn6	0.0000895	7.3326571
160328_at	Prss15	0.0000405	8.2643393
160329_at	1110049F12Rik	0.0090939	7.6860477
160330_at	Chordc1	0.0001044	8.5295013
160335_at	Gclm	0.0000028	6.8848899
160337_at	1300017C10Rik	0.0000008	9.2595784
160338_at	1100001D10Rik	0.0000163	7.5788464
160339_at	Leftb	0.0001128	7.2765458
160341_at	Jtv1−pending	0.0000178	10.0067078
160344_at	Npc2	0.0023770	9.2428931
160345_at	Mrpl34	0.0000248	9.6132099
160347_at	D15Ertd785e	0.0001528	8.0049677
160348_at	2610024N24Rik	0.0000063	8.8704829
160349_at	0610010E05Rik	0.0043501	7.6956874
160350_at	Gstz1	0.0000016	6.6020342
160351_at	Rnpep	0.0001528	6.6089955
160352_at	Pcbp4	0.0038649	8.6719692
160353_i_at	Mapkapk2	0.0002675	4.6849495
160355_at	Maz	0.0007377	7.2458451
160356_at	0610011D08Rik	0.0017358	7.8687619
160360_at	Sep15−pending	0.0111567	10.2737474
160361_at	Trappc4	0.0005231	8.2182907
160362_at	Mat2a	0.0011775	9.7122417
160364_at	Silg41	0.0000028	10.1085385
160365_at	Eif2s2	0.0000066	7.1918295
160369_at	Dhcr24	0.0125474	5.4191556
160370_at		0.0007373	4.7976747
160371_at	Arl6ip1	0.0005607	10.0913700
160374_r_at	Ptbp2	0.0016272	3.9740862
160375_at	Car3	0.0000163	5.4583004
160377_at	Tardbp	0.0000374	9.1890018
160380_at	Becn1	0.0002135	8.3367940
160381_at	Mrpl50	0.0000439	6.2579450
160382_at	1110014J01Rik	0.0001841	7.3714128
160384_at	Bat1a	0.0004239	11.0826977
160385_at	5730591C18Rik	0.0000009	9.6027390
160388_at	Sc4mol	0.0000031	6.0031606
160392_at	B430110G05Rik	0.0096031	5.7858126
160393_at	4930555L11Rik	0.0000193	6.8942099
160394_at	D930014A20Rik	0.0004239	6.0511042
160395_at	D11Ertd603e	0.0000293	9.0833506
160397_at	Ik	0.0000537	7.4541720
160398_at	2210008F15Rik	0.0001774	6.3447854
160399_r_at	H2afy	0.0115892	5.9655134
160400_at	2810422B04Rik	0.0014318	8.2948814
160401_r_at	Cnot7	0.0061552	2.1821633
160402_at	Tceb2	0.0058140	11.2682458
160403_at	Selk−pending	0.0043493	7.6194818
160410_at	Vps16	0.0000374	6.9253974
160411_at	1810063B05Rik	0.0168101	3.5168551
160412_at	Pdcd2	0.0002385	6.3038817
160413_at	Nsg2	0.0021615	3.3694541
160414_at	1810073N04Rik	0.0017358	8.0639740
160417_at	Kif5b	0.0001527	7.6134161
160422_at	Ruvbl2	0.0018492	9.8741437
160423_at	Mrps2	0.0018496	7.1011826

160424_f_at	Fdps	0.0016284	8.5998545
160425_at	2410017I18Rik	0.0026894	6.3491495
160427_at	Hrb2	0.0000967	9.4226671
160428_at	Suclg2	0.0001911	8.7865073
160429_at	Nxt1	0.0000558	8.6159452
160430_at	Catnb	0.0041010	10.8769582
160431_at	Mrpl12	0.0102503	9.0696890
160434_at	2310004K06Rik	0.0017354	6.3926281
160439_at	Polg	0.0000098	6.8792588
160440_at	Madh4	0.0000075	9.0648725
160442_at	Cct2	0.0046129	7.7152748
160445_at	2610005H11Rik	0.0000228	7.5376324
160447_at	Hiat1	0.0000126	7.7130148
160448_at	6030457N17Rik	0.0025288	8.3219351
160449_at	Dr1	0.0096031	7.7672947
160451_at	MGC18745	0.0001647	9.5772008
160454_at	D11Moh35	0.0081474	6.8400834
160455_s_at	D10Ertd749e	0.0000082	9.9105491
160456_at	1100001J08Rik	0.0139359	5.5295958
160457_at	9130413I22Rik	0.0000269	11.1027579
160461_f_at	2310057H16Rik	0.0000069	9.1661256
160462_f_at	Tubb3	0.0026894	10.0748765
160466_at	Rae1	0.0000090	8.0478530
160468_at	Mtpn	0.0033262	7.3402187
160470_at	Mrps5	0.0074951	5.9552584
160471_at	Slbp	0.0000013	8.4987181
160473_at	Nme4	0.0000193	8.1489573
160476_f_at		0.0003421	12.0780777
160480_at	Ptprs	0.0000318	7.2644337
160482_at	Acaa1	0.0001774	7.8017455
160483_at	Tcf4	0.0000075	6.9689654
160487_at	Myl4	0.0002778	4.6744625
160488_at	Msh3	0.0023770	6.9534776
160490_at	Def8	0.0000031	7.1757504
160492_at	Ddx18	0.0000126	9.1498849
160493_at	Cd63	0.0090939	9.0739203
160495_at	Ahr	0.0002384	3.7884891
160496_s_at	Mcm3	0.0000026	10.1577600
160499_at	Tra1	0.0003678	11.2820932
160503_at	Fbl	0.0009656	10.6840508
160507_at	0610013E23Rik	0.0061552	5.3712384
160511_at	Cxcl12	0.0000210	5.2925365
160514_at	Rp9h	0.0028592	7.4354756
160515_at	Parn	0.0028592	7.2936943
160517_at	Lmnb1	0.0000026	8.5806537
160518_at	1110060F11Rik	0.0036411	8.2183803
160520_at	Yap	0.0000269	9.7934311
160521_at	2610020N02Rik	0.0000015	7.2545443
160529_r_at	Vdac2	0.0001911	8.0715832
160531_at	Grcc2f	0.0000178	9.8170410
160534_at	Psmc5	0.0002216	8.9451005
160536_at	Hras1	0.0139338	7.2799302
160538_at	Cdk4	0.0017358	10.9822707
160539_at	Sfrs1	0.0000008	8.2479746
160540_at	Bag1	0.0054888	8.0661865
160543_at	Snx3	0.0000016	10.5003692
160544_at	Fabp5	0.0001127	7.6003259
160550_i_at	Magoh	0.0000222	9.7044042
160551_at	Vdac3	0.0000008	8.8473671
160552_at	Ap1s1	0.0051821	8.3227242
160554_at	Eif3s6	0.0000828	8.0126462
160555_r_at	Snrpb	0.0000033	11.9532978
160557_at	Tssc4	0.0032280	8.2985142
160558_at	Akt2	0.0112845	7.5747709
160562_at	Cct7	0.0001647	10.9591006
160569_at	2310008M10Rik	0.0046129	9.9125331
160571_at	Idh1	0.0004090	6.6611972
160572_at	Dhx15	0.0021644	6.1358528
160573_at	Hccs	0.0023037	6.8476412
160575_at	Cggbp1	0.0000345	8.2268564
160576_at	4930563P03Rik	0.0038649	5.2560635
160578_at	Pdpk1	0.0006008	7.6047107
160581_at	Apg16l−pending	0.0004548	7.0472192
160585_at	2810470K21Rik	0.0000040	6.5131937
160588_at	Zfp131	0.0003949	7.3294943
160592_at	Tmc6	0.0163667	6.7163316
160593_at	Snx9	0.0068924	8.3205644
160596_at	1110030J09Rik	0.0015272	6.3281669
160602_at	Pde6d	0.0003548	6.5485147
160603_at	Pparbp	0.0008446	7.1550917
160605_s_at	4833420O05Rik	0.0004878	4.2670861
160607_at	Pard3	0.0046120	7.7592275
160611_at	Cyp4v3	0.0072874	2.5412837
160615_at	Pias3	0.0081460	5.2390232
160616_at	Whsc2h	0.0000163	8.9041743
160617_at	Klf13	0.0054898	7.0637901
160621_at	Mrps22	0.0002966	6.6220815

160624_at	Kpna6	0.0000861	6.9754975
160625_f_at	LOC228019	0.0000559	6.8758588
160626_at	Myef2	0.0011025	8.4404484
160628_at	Gcat	0.0009654	7.1945514
160632_at	Prkcn	0.0000163	6.4888182
160633_at	Refbp1	0.0000069	9.3715763
160636_at	AI854251	0.0101358	5.9944093
160637_at	Mocs2	0.0006006	5.8250867
160640_at	E430002F06Rik	0.0028592	6.4853492
160641_at	Pfkfb3	0.0074745	5.6904331
160643_at	Hdac2	0.0000019	9.3078366
160646_at	Gsr	0.0000069	8.3093443
160652_at	Ctps2	0.0014318	6.7128441
160653_at	Tomm40	0.0001647	8.5982964
160654_at	2310073E15Rik	0.0000126	6.9074220
160657_at	Ilf3	0.0000098	8.9149957
160659_at	2310057G13Rik	0.0000106	6.7231077
160662_r_at	Gata6	0.0086076	4.3617751
160664_at	1200004M23Rik	0.0061552	7.3419483
160666_at	Actr6−pending	0.0038649	6.7235082
160668_at	Ogfr	0.0000178	8.6420578
160669_at	2600017A12Rik	0.0001128	8.0261692
160671_at	Cln8	0.0028592	7.0473928
160676_at	1810012N18Rik	0.0000098	9.0310615
160677_at	BC031407	0.0005231	6.4579078
160681_at	Papola	0.0001416	6.8834367
160682_at	6430706D22Rik	0.0000106	8.4363756
160685_at	D5Ertd363e	0.0070875	6.2315549
160686_at	5730555F13Rik	0.0000828	8.1305221
160688_at	Golph3	0.0000439	7.8576103
160691_at	4930511A21Rik	0.0019703	5.3897023
160693_at	Pip5k2c	0.0011775	6.3540601
160696_at	Tia1	0.0000016	6.8223726
160698_s_at	Prkcd	0.0004239	8.1260622
160699_at	2610036L13Rik	0.0146787	7.4359346
160702_at	BC031468	0.0054898	6.4697307
160703_at	Pdip46−pending	0.0022336	8.0109509
160704_at	1110067D22Rik	0.0009032	5.5745084
160707_at	interferon gamma receptor 2	0.0000967	7.1864627
160710_at	Vdu1−pending	0.0000026	6.3555181
160712_r_at	E130012A19Rik	0.0096031	8.2337775
160714_at	Gab1	0.0000008	7.1988250
160715_at	AW146242	0.0125455	5.1416802
160718_at	Capn7	0.0000026	6.7982951
160719_at	A230072I16Rik	0.0011025	7.0320896
160723_at	1500001M20Rik	0.0022336	7.2304836
160727_at	2410002F23Rik	0.0016284	8.2421227
160734_at	Ap3s1	0.0000228	7.8249456
160736_at	4932432N11Rik	0.0106982	5.5687577
160737_at	Lss	0.0000345	6.2509464
160739_at	Prkwnk1	0.0000047	6.6698950
160742_at	Plod3	0.0026894	7.2648210
160743_at	Pole3	0.0019703	7.7150590
160744_r_at	Ctrl	0.0130512	5.7547634
160745_at	Gcn5l2	0.0006434	7.3932029
160746_at	Rock1	0.0018492	5.3503098
160750_at	Prpsap2	0.0000163	7.1439886
160751_i_at	Crnkl1	0.0001541	7.2665950
160755_at	Kif2c	0.0000090	8.7621392
160757_at	Rnf38	0.0058140	7.4178182
160758_at	Sec13l−pending	0.0000193	8.1708089
160759_at	Rfc2	0.0008164	8.6081844
160762_at	Abr	0.0026894	6.7261386
160764_at	Fbxw1b	0.0000116	7.6512781
160766_at	Cd2bp2	0.0086076	5.9071435
160768_at	D030056L22	0.0000345	7.2664578
160769_at	Flana−pending	0.0003187	6.2680845
160770_at	Mvd	0.0000331	6.4569980
160772_i_at	Slu7−pending	0.0060337	6.8298058
160773_at	D4Wsu132e	0.0001774	5.5935194
160775_at	Tulp4	0.0000476	7.3903045
160777_at	AA408451	0.0002058	6.4199293
160779_at	D19Ertd703e	0.0001313	8.9518491
160780_at	Tcf3	0.0000967	8.4742119
160783_at	D14Ertd436e	0.0026888	5.6652334
160784_r_at	2610039C10Rik	0.0034293	4.8552507
160785_at	5530401C11Rik	0.0001128	6.7183721
160787_at	1500010J02Rik	0.0000766	7.1778046
160788_at	Pes1	0.0000058	7.7355403
160789_at	9530090G24Rik	0.0009980	7.4414373
160790_s_at	Btrc	0.0146966	6.1525085
160791_at	Luc7a−pending	0.0014784	6.9587713
160792_at	Snap25bp	0.0002215	6.9620681
160798_at	Nrbf1	0.0019703	5.6518355
160800_at	transcription factor 3	0.0004877	6.9863320
160801_at	2310009N05Rik	0.0061552	6.7432448
160802_at	Ppan	0.0015272	8.0326418

160803_at	D0H8S2298E	0.0000016	6.7605335
160804_at	Eif2b5	0.0000345	8.2560907
160805_s_at	Mpdu1	0.0011775	7.7629785
160806_at	Stk39	0.0001217	5.9412517
160807_at	Agpat3	0.0006436	6.0358676
160809_at	Tollip	0.0049219	6.4697344
160811_at	MGC56855	0.0000031	7.4280486
160812_at	Gga2	0.0000163	9.3812346
160820_at	Igsf8	0.0046129	7.5121454
160821_r_at	Ppp6c	0.0125493	7.2361563
160823_at	Nedd1	0.0000895	6.7649560
160824_at	1110037N09Rik	0.0000021	5.7383316
160825_at	5730484M20Rik	0.0096031	8.3572883
160826_at	Nsmaf	0.0012572	6.2491004
160827_at	Rin2	0.0132266	6.3470013
160831_at	AI838661	0.0005231	6.1338895
160832_at	Ldlr	0.0000476	7.3592922
160840_at	Arhgef3	0.0046129	3.9940057
160842_at	Kin	0.0016284	6.0450420
160844_at	Pts	0.0000439	5.8094284
160845_at	2600001J17Rik	0.0112863	7.9962120
160847_at	Trnt1	0.0000058	8.7736532
160848_at	Zhx1	0.0050322	6.2026686
160849_at	Dlgh1	0.0015272	8.4605158
160850_at	Fpgs	0.0000058	7.4508038
160853_at	Ptdss2	0.0154561	7.9420521
160854_at	Map3k7	0.0002885	6.2094092
160856_at	Slc10a3	0.0013419	6.3112457
160857_at	Efnb2	0.0041010	7.9501296
160858_at	1110046L09Rik	0.0001217	6.2544550
160864_at	Rtkn	0.0020256	6.0691504
160866_at	Dffa	0.0065145	7.8620829
160874_r_at	Ruvbl1	0.0146787	7.1264889
160875_at	Psmb1	0.0132266	9.2767421
160876_at	Bcap29	0.0000828	5.9386362
160878_at	Bop1	0.0002966	8.1223174
160879_at	Terf1	0.0003188	5.0072745
160880_at	Mapk8ip3	0.0001647	7.1134723
160882_at	Ogg1	0.0019703	5.7938506
160885_at	2700010L10Rik	0.0000293	9.8523207
160888_at	Reps1	0.0033931	7.3793299
160889_at	8430437G11Rik	0.0011020	5.6602869
160890_at	Lbr	0.0000011	9.3850773
160894_at	Cebpd	0.0117890	4.9166737
160896_at	Rcn	0.0031852	8.1632476
160897_at	BC005662	0.0061552	7.2737308
160900_at	Gkap42−pending	0.0003808	5.4611140
160901_at	Fos	0.0000008	6.1892645
160904_at	B230317C12Rik	0.0112863	6.5156515
160906_i_at		0.0089414	9.2229857
160911_at	Sos1	0.0005605	5.4305864
160919_r_at	Fgfr3	0.0012162	2.9866709
160920_at	Bcl2l2	0.0096031	6.6282981
160921_at	Acas2l	0.0043501	4.3276462
160923_at	Abp1	0.0068912	6.1363794
160924_at	BC028953	0.0006436	7.6336771
160925_at	Nras	0.0026894	7.3757833
160929_at	Mettl3	0.0036397	5.8349064
160930_at	Sacm1l	0.0011775	6.4499116
160931_at	Dnm1l	0.0000044	6.9159978
160935_at	4921528E07Rik	0.0162648	4.1272243
160936_at	Tram1	0.0007893	7.9934050
160942_at	Nptxr	0.0000828	5.5761476
160949_at	Parg	0.0000010	7.7874647
160959_at	Rcbtb1	0.0003678	7.7968301
160961_at	LOC244668	0.0000708	6.7024430
160962_at	Bag2	0.0019703	8.0564062
160964_at	D16Bwg1494e	0.0003188	8.0196676
160970_at	Odf2	0.0014318	7.6602823
160973_at	C330027C09Rik	0.0000026	7.7009666
160976_at	Diap1	0.0000011	7.2320864
160977_at	Arhgef5	0.0000765	4.8528290
160981_at	C77170	0.0125455	5.8164596
160982_at	4921526G09Rik	0.0001983	6.0881367
160984_r_at	D3Ertd250e	0.0115874	2.0702457
160988_r_at	D3Ertd330e	0.0022336	6.2091900
160990_r_at	BC016198	0.0000159	5.6753404
160997_at	E130303B06Rik	0.0000345	6.7560895
161000_i_at	2610201A12Rik	0.0000055	8.1714320
161003_at	Kif3b	0.0002058	5.2142295
161009_at	5630401M14Rik	0.0000019	6.8301229
161014_at	Terf2ip	0.0000116	5.6024257
161024_at	A430081P20Rik	0.0016284	6.4511925
161038_at	Prps2	0.0009596	4.7738348
161044_at	D030051N19Rik	0.0006436	7.1612983
161046_at	Crlf1	0.0010320	5.7311173
161056_at	−−−	0.0096031	7.3668747

161060_i_at	2310061O04Rik	0.0000642	7.7098477
161061_r_at	Posh−pending	0.0028930	3.7734268
161068_at	NYSAR35	0.0000023	5.7688747
161072_at	Enk−pending	0.0000015	7.6904735
161076_at	MGC36997	0.0000293	7.9647813
161077_f_at	Smarcd2	0.0004819	8.7353444
161082_r_at	1500005I02Rik	0.0090909	4.9345764
161083_at		0.0150607	4.4115477
161096_at	C530030I18Rik	0.0115856	6.0928404
161104_at	9430099J10Rik	0.0000405	7.7010049
161111_f_at	Dhh	0.0101358	6.1360602
161112_at		0.0020328	5.8225067
161122_f_at	Ndufab1	0.0001774	9.0939388
161126_at	−−−	0.0004548	6.5549610
161127_i_at	Rpl24	0.0000941	8.0063605
161129_r_at	Bag2	0.0058140	8.7224619
161135_f_at	2410004C24Rik	0.0094695	6.1729356
161147_f_at	1110046L09Rik	0.0000178	7.7052602
161169_f_at	1810030O07Rik	0.0139338	6.5913434
161214_r_at	MGC47434	0.0166082	7.0016567
161235_f_at	Bat4	0.0000177	6.6648084
161238_f_at	Cct3	0.0002759	8.1492286
161247_f_at	Dgcr2	0.0003188	6.0157829
161250_at	Ipo4	0.0005231	5.6006912
161253_i_at	Plod3	0.0079705	4.9956425
161255_at	Vdac2	0.0034286	3.6333245
161270_i_at	Prkwnk1	0.0000490	6.6939340
161284_r_at	Txnrd3−pending	0.0044711	7.4984620
161286_f_at	Arsa	0.0021639	3.6436739
161287_f_at	Mybbp1a	0.0001127	8.4024716
161294_f_at	Clu	0.0066987	2.9776614
161311_at		0.0058140	3.2179298
161317_r_at		0.0013416	3.8798090
161327_f_at	Rpl10a	0.0001417	11.0816795
161332_f_at	2610036L13Rik	0.0022336	7.2429836
161337_f_at	Wars	0.0006892	6.4044049
161342_r_at	2310046H11Rik	0.0000116	8.4646328
161346_f_at	Prss19	0.0162695	4.3167121
161362_at		0.0074951	1.7242975
161367_f_at	Hps1	0.0077080	6.7944764
161377_at	Emr1	0.0106965	1.8568853
161389_f_at	2210409M21Rik	0.0070875	5.5828437
161396_f_at	B230113M03Rik	0.0114976	7.3206762
161400_f_at	Rpn1	0.0068924	8.0392506
161401_f_at	Aldh3a2	0.0146787	4.2803063
161407_i_at	Inpp1	0.0075529	8.1745564
161409_f_at	Dntt	0.0125493	5.1681704
161414_f_at	Smarcd2	0.0146787	6.1449461
161424_f_at	1110049F12Rik	0.0054898	6.8721478
161432_f_at	Sart3	0.0000516	7.2687645
161433_f_at	Piga	0.0002135	6.0432798
161437_f_at	4930578F06Rik	0.0135731	4.6515194
161439_f_at	Ap1m1	0.0017358	6.5479228
161444_f_at	1110055N21Rik	0.0034286	8.1387331
161458_at	F7	0.0077080	7.8865269
161466_r_at	Asb3	0.0002965	3.5598172
161468_f_at	Hand1	0.0000116	7.1238154
161475_f_at	1700007F21Rik	0.0090939	0.8811497
161476_at	Psap	0.0006658	6.6321636
161480_i_at		0.0084697	10.9029539
161482_f_at	Prph1	0.0158536	2.7410748
161487_f_at		0.0025282	7.2215176
161498_at		0.0132266	8.6111768
161499_f_at		0.0017358	5.5153183
161510_f_at	Scn1b	0.0035323	8.1042089
161514_at	Cct5	0.0046129	7.7033861
161519_f_at		0.0000116	5.9728951
161548_r_at	Ryk	0.0132266	3.9289496
161572_r_at	Ercc2	0.0086090	3.1715437
161613_at	6330407G04Rik	0.0154561	6.1907835
161633_r_at	Gstm1	0.0158559	6.1565384
161639_f_at	Gast	0.0134574	3.9865528
161648_at	Crry	0.0063314	7.3890919
161651_f_at	Orc2l	0.0038422	3.4062162
161657_f_at	Rpl10	0.0002216	10.1858915
161695_f_at	Slc6a4	0.0077067	4.7295139
161698_f_at	BC019977	0.0009032	8.2168823
161745_f_at	Hspa4	0.0065145	7.2179031
161757_f_at	Kpnb3	0.0023770	7.5040868
161767_r_at	Mrps18a	0.0019876	7.5945481
161770_f_at	D1Ertd161e	0.0000228	6.2423131
161774_f_at	Lypla1	0.0021644	7.1852465
161787_f_at	Ris2	0.0000559	8.2482234
161796_r_at	Kcnq1	0.0000967	5.9705254
161829_at	Nes	0.0050322	2.3977088
161847_r_at	2210016L21Rik	0.0125455	6.4595982
161857_r_at	AI428936	0.0139359	5.3033909

161872_f_at	1110049G11Rik	0.0000895	6.0815749
161889_f_at	Aldo1	0.0001647	10.5283629
161897_f_at	Prps1	0.0019703	5.5170412
161898_i_at	Mtx1	0.0101393	7.5758068
161906_f_at	2410022L05Rik	0.0001363	5.9811970
161926_r_at	P4hb	0.0139359	8.4631013
161951_f_at	D130005A03	0.0020982	6.1223967
161980_f_at	Bag3	0.0001020	6.7997344
161983_f_at	Rnh1	0.0026291	5.0496741
161988_f_at	Lrpap1	0.0006005	4.5418717
161990_f_at	Diap1	0.0030388	7.7951073
161997_f_at	Aldh2	0.0012423	5.6710081
162004_i_at	Lig1	0.0085360	3.8400368
162031_f_at	A530057M15Rik	0.0115874	6.8636586
162032_f_at	Pkm2	0.0034924	11.2634736
162033_f_at	D2Wsu81e	0.0074951	7.7373223
162041_f_at	Ly64	0.0006892	7.1249079
162077_f_at	Scd2	0.0054898	9.5172188
162089_r_at	Tln	0.0010741	9.6702311
162091_f_at	Sf3b2	0.0014784	8.1002273
162094_f_at	Wtap	0.0004877	6.2333002
162121_at	Krt1−10	0.0068900	1.5139821
162125_f_at	Ubc	0.0009032	8.2380894
162138_s_at	Nptxr	0.0046120	6.6498559
162145_r_at	Pgs1−pending	0.0089664	3.8961605
162157_f_at	Stk16	0.0154538	3.6910381
162171_f_at	Fgfr2	0.0053319	4.4742047
162172_f_at	Nedd4	0.0035101	4.8789354
162177_f_at	Rpl3	0.0038649	6.3378009
162188_f_at	P4ha2	0.0004236	6.2306225
162234_f_at	Cxcl12	0.0024615	5.8362762
162245_f_at	Myo6	0.0048900	6.6588537
162275_f_at	Upf3b	0.0139318	5.8318944
162279_f_at	Cct6a	0.0000010	9.4971097
162299_f_at	Pbp	0.0000605	8.2320475
162313_f_at	Galnt3	0.0004044	5.8375652
162335_at		0.0117971	3.2267813
162379_r_at	Vim	0.0069671	6.1833256
162399_f_at		0.0000292	8.6651861
162400_f_at	Mms19l	0.0047487	4.7646179
162401_f_at	1300017C10Rik	0.0009334	5.3541964
162412_r_at		0.0143539	0.8274277
162417_at	1500001M20Rik	0.0054898	6.2523342
162426_f_at	Scamp1	0.0005803	8.1964140
162460_f_at	Igbp1	0.0072900	5.6067832
162467_r_at	AI256456	0.0110917	5.0033063
162486_f_at	Dscr3	0.0000331	7.9205762
162489_i_at	Ddx47	0.0079705	7.6324930
162499_f_at	Ube2d2	0.0139338	5.8913982
92180_at	H1fx	0.0028592	9.0237577
92182_at	Rufy1	0.0012165	5.6732939
92189_at	1810045K17Rik	0.0003678	6.8311010
92191_at	2810410A08Rik	0.0000019	6.4112412
92202_g_at	Zfp145	0.0047478	5.5354588
92208_at	C1qdc1	0.0098654	6.4976031
92211_at	AF013969	0.0002058	6.6686170
92216_at	Madh7	0.0000126	6.2945509
92220_s_at	Bin1	0.0119030	7.2730478
92225_f_at	Rpo1−2	0.0001255	7.9510903
92226_at	Ddx50	0.0000069	7.8537475
92230_at	4930472G13Rik	0.0006217	4.1842835
92233_at	1810007M14Rik	0.0000031	7.7950264
92239_at	Elavl3	0.0086090	4.0891577
92243_at	B930007L02Rik	0.0000011	8.7848310
92244_at	Exo1	0.0000083	6.6262289
92247_at	Arhgap5	0.0000765	5.5609071
92254_at	Myo5b	0.0018332	6.0319524
92257_at	Clock	0.0036411	5.3440167
92259_at	Tbl3	0.0112863	7.7670733
92262_at	Wig1	0.0025288	7.4581735
92263_at	Grcb	0.0047487	4.5101324
92269_r_at		0.0000150	6.7941333
92270_at	Tro	0.0000766	8.1474668
92272_at	SRY−box containing gene 3	0.0008446	6.2193998
92275_at	Tcfap2c	0.0043501	7.1363381
92278_at	Ercc2	0.0043501	7.4691327
92279_at	CGI−141−pending	0.0032287	6.3247091
92280_at	Strm	0.0041010	7.2792354
92282_at	2610304G08Rik	0.0000009	7.6188060
92288_at	Ap1g1	0.0000010	6.0543434
92290_at	Siah1a	0.0025282	7.0632531
92292_at	Slc2a3	0.0028592	9.7274419
92294_at	2810410P22Rik	0.0000210	6.9775323
92302_at	Sos2	0.0132266	4.7169516
92304_at	Piga	0.0001044	6.9552681
92311_s_at	Pik3c2a	0.0158559	4.0072733
92317_at	Elavl2	0.0000930	5.3428028

92325_at	Ptk7	0.0012572	7.2625371
92338_f_at		0.0000090	6.0537576
92339_at	Taf1a	0.0005414	6.3977291
92345_g_at	Smarca3	0.0026894	6.4736526
92360_at	Stam	0.0004548	7.9400460
92375_at	1810015P09Rik	0.0003948	7.1224887
92383_at	Dyrk1a	0.0011775	5.9635142
92384_at	Xpa	0.0004708	5.2665687
92394_f_at	Crebl1	0.0006008	6.3623349
92408_at	C130065N10Rik	0.0050728	4.9233210
92409_at	Zfp260	0.0112863	5.6760415
92410_at	Rad23a	0.0000185	6.7737637
92411_at	Hs1bp3−pending	0.0002208	7.4373507
92412_s_at	Spag5	0.0000221	5.4643623
92423_at	Pard6a	0.0034293	6.1075817
92424_at	AI839920	0.0028592	6.5715016
92428_at	Asna1	0.0000034	9.5643555
92434_at	Traip	0.0019703	6.7514985
92440_at	Irf6	0.0003810	5.4640264
92443_i_at	Zfp1	0.0000125	7.8749630
92444_f_at	Zfp1	0.0000009	7.9247364
92456_at	Taf1c	0.0000037	7.0630799
92458_at	Orc1l	0.0001044	7.1483061
92468_at	Gbif−pending	0.0016284	6.2044598
92477_at	Spin	0.0002058	6.8455152
92478_at	Stag1	0.0004390	6.9965254
92480_f_at	Zfp118	0.0000476	5.4977189
92486_at	Slc22a9	0.0048900	4.1765824
92487_at	Sox7	0.0119030	5.4694297
92492_at	Ak3l	0.0066999	4.4538560
92500_at	Odz3	0.0000063	6.5416863
92502_at	Plagl1	0.0025288	3.6295219
92503_at	Hus1	0.0001171	6.0289017
92506_at	Crtl1	0.0053319	3.0776155
92507_at	Utrn	0.0009978	5.2334054
92513_at	Stag2	0.0002659	8.7261605
92517_at	Rbms2	0.0005229	6.6628192
92521_at	Atbf1	0.0000828	6.6498657
92529_s_at	Arnt	0.0000026	5.4805890
92539_at	S100a10	0.0000040	8.6166610
92540_f_at	Srm	0.0000008	9.9658952
92544_f_at	Psma3	0.0058140	11.2244093
92547_at	Hip2	0.0010317	7.3508422
92548_g_at	Hip2	0.0043501	7.6088971
92549_at	Pkig	0.0002759	7.7320115
92551_at	Lig1	0.0001217	8.7538514
92553_at	Es10	0.0029889	9.1482965
92554_at	Ctbp2	0.0000516	8.7760655
92562_at	Nfe2l2	0.0000344	7.8413605
92565_at	1110005A23Rik	0.0000023	8.7017355
92567_at	Col5a2	0.0005604	6.0574961
92568_at	Tfb2m	0.0054888	5.6123454
92569_f_at	Nol5	0.0000026	9.1039148
92571_at	Hspa4	0.0000374	8.9340307
92572_at	Tnfaip1	0.0020967	5.5075299
92573_at	Ppp2r1a	0.0001044	10.2449465
92577_f_at	Rpl37	0.0000895	12.8004125
92578_at	Scye1	0.0000011	9.4556683
92579_at	Ssb	0.0000009	6.8738587
92580_at	Hars	0.0162695	8.2156066
92582_at	Slc1a7	0.0000009	5.6567276
92584_at	2410003M04Rik	0.0052290	6.8168655
92585_at	Map2k1	0.0000724	7.7809217
92586_at	Glud	0.0058140	7.6179909
92589_at	Psph	0.0042231	8.4162238
92595_r_at	Fech	0.0155106	2.6083561
92596_at	Cacybp	0.0000009	8.0556001
92597_s_at	Atp6v1b2	0.0000028	6.0870565
92598_at	Atp6v1b2	0.0000228	9.8224269
92603_at	Atp6v0d1	0.0000015	8.5593546
92607_at	Mest	0.0000011	8.0080684
92610_at	Rdbp	0.0004239	8.6847968
92611_at	Gpiap1	0.0000193	10.0536103
92614_at	Idb3	0.0001217	9.1950095
92615_at	−−−	0.0004878	9.7355396
92616_at	Ube1x	0.0000063	9.8494860
92621_at	Pcbp2	0.0000009	11.2121482
92622_at	2210013K02Rik	0.0001044	8.8518577
92623_at	D3Jfr1	0.0001647	9.6217138
92624_r_at	Lor	0.0043493	6.9170903
92625_at	Nme2	0.0043501	11.3598698
92628_at	Rpl36	0.0004878	11.6906185
92629_f_at	Hdgf	0.0016284	11.2673534
92631_f_at	Calm3	0.0019352	7.7505447
92632_at	Calm3	0.0000008	8.7587478
92633_at	Ctsz	0.0000248	8.4414870
92637_at	Pfkl	0.0000011	10.9429337

92638_at	Ppp2ca	0.0000063	6.7587003
92639_at	Stk6	0.0000178	8.5812076
92640_at	Vps35	0.0000016	9.7246037
92642_at	Car2	0.0000116	8.7865877
92643_at	Nf2	0.0000018	7.8166235
92644_s_at	Myb	0.0000008	6.4325144
92646_at	Mrpl23	0.0000269	8.8114901
92647_at	Rbbp4	0.0001417	10.2108097
92648_at	Stxbp3	0.0005607	6.2914912
92653_at	D530037H12Rik	0.0018496	4.5942728
92654_at	MGC62420	0.0007373	6.2497585
92655_at	Gcnt1	0.0086090	6.2738402
92660_f_at	Ube2e1	0.0000476	7.5533247
92678_at	Ddx25	0.0068924	6.5423042
92681_at	Magel2	0.0000193	6.0675960
92697_at	Foxa1	0.0002385	5.5855385
92699_at	Slc7a9	0.0001527	6.5798696
92703_at	2310032M22Rik	0.0036404	7.1416214
92708_at	2810457M08Rik	0.0000178	7.1455082
92724_at	Hnrpa1	0.0000126	7.2224675
92746_at	Nat2	0.0043501	4.4581174
92754_at	Fdxr	0.0053874	5.8282462
92756_r_at	Sct	0.0034293	8.8507001
92758_at	Dusp2	0.0128821	6.0400748
92766_at	Hand1	0.0000031	8.7942511
92767_at	Bmpr1a	0.0054898	6.7192306
92769_at	Psmd3	0.0001313	9.5625720
92771_at	Zfp207	0.0000021	9.2869873
92773_at	Ier5	0.0000015	6.7575720
92774_at	Pabpc4	0.0008732	5.9770062
92775_at	−−−	0.0000037	10.4270701
92777_at	Cyr61	0.0051821	5.3287083
92781_at	Tmpo	0.0125493	5.3381028
92782_at	Tmpo	0.0001417	9.0334986
92787_at	Ankrd10	0.0000009	9.3116318
92788_f_at	Cetn3	0.0002720	9.3510050
92789_r_at	Cetn3	0.0023749	5.3331665
92790_at	Kpna2	0.0000439	9.3831142
92794_f_at	Nme1	0.0000053	8.1936900
92795_at	Mtap4	0.0000040	6.1960553
92796_at	Akp2	0.0000060	6.9910637
92797_at	Cul3	0.0000248	8.9448595
92798_at	Atp5c1	0.0000163	9.9525027
92800_i_at	Atp5c1	0.0000089	11.2012533
92804_at	Polr2h	0.0006892	8.8914541
92806_at	1110004D19Rik	0.0036411	7.4926051
92807_at	Txn1	0.0034293	11.2752545
92808_f_at	Fkbp4	0.0001911	11.4507522
92809_r_at	Fkbp4	0.0000178	9.7962742
92810_at	Pdk3	0.0003949	8.5307453
92816_r_at	Eif4a1	0.0001528	8.5523069
92817_at	1190002L16Rik	0.0000655	9.4332548
92818_at	Ube1c	0.0002384	6.9609944
92828_at	Dpm1	0.0000828	7.1926439
92829_at	Hspe1	0.0025288	11.3002866
92831_at	Sfxn1	0.0001313	9.2320580
92834_at	Rpl13a	0.0020977	10.7193114
92838_at	Fscn1	0.0000106	11.0437455
92839_f_at	Snrpb2	0.0000374	4.8826597
92840_at	3110079L04Rik	0.0029889	7.9777938
92841_f_at	Chgb	0.0090909	3.2434806
92845_at	Oxct	0.0000019	7.2506367
92847_s_at	chromogranin B	0.0083740	7.0590250
92848_at	Oat	0.0009656	9.0224210
92850_at	Rrbp1	0.0062624	8.7585912
92854_at	Rab11a	0.0000248	8.8906295
92855_at	Sui1−rs1	0.0011025	9.9638086
92870_at	Sel1h	0.0002574	6.2355090
92872_at	1200016B17Rik	0.0043501	7.5751351
92879_at	Ppm1g	0.0000106	9.9200852
92882_at	Rab1	0.0000018	7.4800141
92884_at		0.0000058	10.8117836
92887_at	Ddah2	0.0004035	9.1909542
92888_s_at	Styx	0.0000011	6.5313076
92907_at	Ocln	0.0061541	4.1292407
92915_s_at	Hoxb7	0.0008732	5.6300658
92918_at	F7	0.0143009	4.4335512
92927_at	Etv1	0.0000344	4.9135222
92930_at	Dlx5	0.0077067	5.8987237
92931_at	Dll1	0.0061552	7.8630519
92933_at	Pou2f3	0.0081446	6.0794997
92934_at	Zfp90	0.0001276	5.8795014
92956_at	Notch3	0.0048891	7.4996696
92957_at	Fgf3	0.0154561	6.4399693
92961_at	Lhx1	0.0065145	6.1011494
92963_at	MGC32441	0.0158559	7.5018572
92979_at	Etv4	0.0003949	6.2343551

92992_i_at	5730497N03Rik	0.0121291	3.1621042
92996_at	Sox17	0.0002385	7.6159656
92998_at	Vav2	0.0003678	5.7145419
93002_r_at	Tdgf1	0.0003949	4.2307939
93008_at	Lsm4	0.0000008	10.1370297
93013_at	Idb2	0.0000058	9.7991828
93014_at	Atp5l	0.0016284	10.2774337
93016_at	Ywhaq	0.0139359	5.2834393
93017_at	Sdcbp	0.0016280	4.9579412
93019_at	H2afx	0.0146787	10.9723005
93020_at	Rex3	0.0000374	9.5612673
93021_at	LOC406217	0.0000150	11.0587023
93023_f_at	Hist2h3c2	0.0000116	8.0437807
93025_at	Ndfip1	0.0000069	8.4166412
93026_at	Mgst1	0.0125493	4.3346021
93029_at	Idh3g	0.0001313	8.8139494
93030_at	Rps27a	0.0000150	12.0064526
93033_at	Ube2e3	0.0089967	5.2975976
93041_at	Mcm4	0.0000293	9.9234029
93042_at	Bzrp	0.0036411	8.7948675
93043_at	Sdfr1	0.0000044	7.7145689
93045_at	Abcd3	0.0096031	6.8952362
93046_at	Nup50	0.0000058	9.3397607
93047_at	Nup50	0.0001891	7.9347350
93048_at	Clpp	0.0000895	8.7031199
93054_at	1110054N06Rik	0.0016284	7.4041620
93055_at	1110054N06Rik	0.0000293	6.5726247
93056_g_at	1110054N06Rik	0.0039805	6.4094609
93057_at	LOC218490	0.0000967	10.7548867
93058_at	Eif1a	0.0000031	8.6195562
93062_at	Mrpl39	0.0001087	7.8476870
93063_at	App	0.0001417	9.3997986
93066_at	Grn	0.0003074	7.4546432
93067_f_at		0.0007895	8.1396333
93069_at	Ube2d2	0.0006892	8.6364346
93070_at	Kpnb3	0.0000008	10.0051737
93071_at	Trim28	0.0000138	12.6211405
93076_at	Csnk1a1	0.0000106	9.0942904
93080_at	Bscl2	0.0008446	7.8399873
93081_at	Rbbp7	0.0000098	11.1331947
93082_at	Awp1−pending	0.0002565	9.1593306
93084_at	Slc25a4	0.0000008	10.5379735
93089_at	Eif4a2	0.0000010	10.1922086
93093_at	Mcl1	0.0036411	8.0993344
93094_at	Cdr2	0.0006892	6.6299818
93095_at	Hmgb1	0.0002385	9.7181433
93096_at	3010002H13Rik	0.0159285	4.5721753
93097_at	Arg1	0.0000044	5.8383152
93100_at	Acta2	0.0000359	5.2797914
93101_s_at	Nedd4	0.0000605	9.8603628
93102_f_at	Actg2	0.0119030	7.1666027
93111_at	Kpnb1	0.0000374	10.3638777
93112_at	Mcm2	0.0008446	9.8122205
93116_at	Prkacb	0.0000015	8.2714535
93117_at	Hnrpa2b1	0.0000021	11.3267429
93118_at	Hnrpa2b1	0.0000106	6.7824399
93119_at	Cox5b	0.0002759	10.0148823
93120_f_at	H2−K	0.0001417	7.5332317
93126_at	Ckb	0.0000090	8.0984880
93130_at	2600005C20Rik	0.0000011	8.7699488
93133_at	Slc7a3	0.0028592	7.4455113
93138_at	2410012H22Rik	0.0000708	6.4670463
93143_at	1190005I06Rik	0.0000201	5.9311577
93144_at	AI317237	0.0000318	7.8739571
93145_at		0.0016276	5.5452918
93164_at	Rnf2	0.0000069	6.4111664
93169_at	9630054P07Rik	0.0000020	8.0265764
93174_at	6030411F23Rik	0.0005231	7.8946289
93180_at	AI506816	0.0000011	6.2135999
93183_at	BC028278	0.0038649	7.3099295
93184_at	Tbl2	0.0015272	7.0079540
93190_at		0.0000022	7.3907748
93195_at	Mfhas1	0.0026894	5.5675934
93200_f_at	D1Pas1	0.0166830	4.3019568
93203_f_at	3230402J05Rik	0.0006656	3.8256731
93208_at	Try3	0.0146743	3.4373725
93211_at	Dnajc5	0.0010662	7.0903661
93215_at	Tnfaip1	0.0000016	6.0818052
93217_at	2610017J04Rik	0.0003425	7.2148829
93218_at	Swap70	0.0083940	4.4155236
93219_at	Acp1	0.0016280	6.5393173
93228_at	Hells	0.0000516	8.4594153
93235_at	BB128963	0.0036389	5.4001751
93236_s_at	Tyms	0.0000028	8.3953674
93240_f_at	Upf3b	0.0006890	7.6480769
93246_at	Narg1	0.0000150	8.1816631
93250_r_at	Hmgb2	0.0000063	8.7951716

93251_at	Nipsnap1	0.0162695	7.3016060
93253_at	Mapk1	0.0012560	6.7350524
93254_at	Mapk1	0.0007377	9.1517948
93255_at	Ralbp1	0.0004547	8.5430300
93256_at	C130038N16Rik	0.0000170	6.8936060
93257_at	Ddx1	0.0003678	9.2411391
93261_at	Lgmn	0.0000098	9.0198503
93264_at	Srebf1	0.0000013	8.5377044
93267_at	Rnpc2	0.0000766	8.6545304
93268_at	Glo1	0.0000009	8.3685561
93269_at	Glo1	0.0022331	9.0436938
93270_at	Gars	0.0000048	9.7143427
93272_at	Stk16	0.0018960	7.5066070
93274_at	Clk	0.0000318	9.1136296
93276_at	Hn1	0.0006008	9.2889410
93277_at	Hspd1	0.0000210	12.0174100
93278_at	Scp2	0.0006892	6.5498553
93281_at	Rcn2	0.0048900	7.8819997
93283_at	Aprt	0.0000439	6.0222991
93285_at	Dusp6	0.0048900	7.7347196
93288_at	Arpc2	0.0000012	10.0655274
93290_at	Pnp	0.0000116	9.2791146
93293_at	Calm2	0.0000476	12.1151440
93294_at	Ctgf	0.0000895	5.6602315
93295_at	Cct5	0.0000026	11.4273567
93301_at	1300007B12Rik	0.0000828	6.5165706
93305_f_at	Vamp8	0.0132266	7.0731316
93306_at	Mapre1	0.0002216	9.8534072
93309_at	Fin14	0.0000037	8.1492589
93310_at	Psmc6	0.0000011	10.8859576
93311_at	Clk3	0.0112863	7.2315700
93312_at	Ube2g1	0.0013419	8.6700247
93315_at	Map2k3	0.0047487	7.4061175
93316_at	Osbpl1a	0.0000019	6.4617310
93318_at	Ninj1	0.0025288	4.0163625
93319_at	Rasa3	0.0096031	5.3599679
93323_at	Plp2	0.0000439	10.8026179
93324_at	Zfp36l1	0.0096031	8.1643591
93325_at	Polr2e	0.0000559	8.1765409
93326_at	Tm4sf2	0.0128821	8.5440854
93327_at	1300011C24Rik	0.0095999	7.0769026
93337_at	Vps4b	0.0000766	8.0573553
93339_at	Mdm4	0.0000090	7.3943891
93340_f_at	Copb2	0.0000013	7.4369741
93341_r_at	Copb2	0.0122205	9.3074285
93342_at	Mki67ip	0.0000027	8.0163997
93346_at	Pgk1	0.0000023	11.8720238
93354_at	Apoc1	0.0004688	7.1486076
93356_at	Mcm7	0.0004239	10.3764185
93358_at	1500010B24Rik	0.0000037	10.1138701
93360_at	Pmm1	0.0016284	6.9859979
93364_at	Catna1	0.0000293	8.8974370
93365_s_at	2410174K12Rik	0.0004548	10.0589190
93366_r_at	2410174K12Rik	0.0001911	8.2338417
93371_at	Bnc	0.0115874	4.1548273
93372_at	Anp32a	0.0000008	8.5168947
93389_at	Prom1	0.0162695	8.6098981
93405_at	E2f3	0.0009654	5.4272398
93410_at	1810073P09Rik	0.0023759	5.8296116
93413_at	Terf2	0.0000495	6.3043954
93414_at	Abcb1b	0.0058983	3.1417911
93419_at	1110061O04Rik	0.0001217	7.1892447
93421_at	Pftk1	0.0090939	5.8476206
93422_at	Pftk1	0.0066426	4.6062405
93424_at	Phr1	0.0000063	7.5771908
93426_at	1110014E10Rik	0.0024513	8.0644400
93427_at	9930104H07Rik	0.0045336	4.7233904
93436_i_at	4632419I22Rik	0.0131917	4.0106513
93440_at	4930564D15Rik	0.0006892	6.2869636
93441_at	2700099C18Rik	0.0000026	7.0631181
93451_at	Lmo7	0.0000048	7.3188393
93452_at	Cer1	0.0000058	6.9692374
93464_at	Akap9	0.0026894	5.2631201
93470_at	Dnmt2	0.0036411	6.0332825
93471_at	Slc4a7	0.0010320	8.2622403
93476_at	Prkdc	0.0000516	6.0342637
93478_at		0.0000016	6.4908031
93488_at	AW550801	0.0000011	9.6510410
93491_f_at	1100001F19Rik	0.0048900	7.6906029
93492_at	Pscd2	0.0025282	7.4958355
93493_at	Ddx5	0.0000012	9.5393208
93498_s_at	Aplp2	0.0000248	8.9079930
93499_at	Cappa1	0.0000008	9.2426371
93500_at	Alas1	0.0058140	7.2760195
93501_f_at	Sucla2	0.0006436	7.9528742
93502_r_at	Sucla2	0.0132266	3.6849373
93503_at	Sfrp2	0.0000010	7.9301388

93509_at	Ube2b	0.0004387	6.7536868
93512_f_at	Adk	0.0000009	9.3665458
93518_at	Rnps1	0.0000015	9.9270028
93519_s_at	Nedd8	0.0005834	9.4027688
93520_at	Srrm1	0.0000269	5.8104847
93521_at	Srrm1	0.0000098	10.0660152
93525_f_at	Ube2j2	0.0030388	6.7827843
93528_s_at	Bteb1	0.0001313	7.4492079
93529_at	2310047C21Rik	0.0001417	7.0552074
93530_at	2310047C21Rik	0.0061552	5.1751359
93531_at	Ndufa8	0.0017358	7.9943084
93533_at	1500011L16Rik	0.0007895	8.4678300
93535_at	Sca2	0.0043493	7.9250432
93536_at	Bax	0.0011775	8.7722954
93538_at	Ttrap	0.0000022	7.6713251
93539_at	1810004D07Rik	0.0041010	8.0187019
93540_at	Adprh	0.0000258	8.6283116
93541_at	Tagln	0.0162695	5.7628144
93546_s_at	Cbfb	0.0000098	9.7911889
93547_at	Cbfb	0.0000008	10.0023589
93548_at	1190006C12Rik	0.0030388	9.8278076
93550_at	Csrp2	0.0000008	9.6344824
93551_at	Polr2l	0.0051821	9.3234622
93557_at	Sps2	0.0001128	7.6345773
93559_at	Apex1	0.0001528	10.2967359
93560_at	Acyp1	0.0001527	7.6695944
93562_at	Ndufb3	0.0001364	8.9801159
93563_s_at	Nid2	0.0000967	7.7634028
93564_at	Yars	0.0006436	8.0006599
93568_i_at	2610042L04Rik	0.0000088	8.8118229
93569_f_at	2610042L04Rik	0.0000008	7.7102157
93571_at	Spnb2	0.0001911	8.9139139
93572_at	Ndufs1	0.0000048	8.8044334
93573_at	Mt1	0.0000015	8.7767639
93580_at	Pum2	0.0032280	9.1943812
93583_s_at	Igh−6	0.0048900	4.9307474
93588_at	Gtl3	0.0000011	8.2302701
93595_at	Cln2	0.0000069	8.9921014
93598_at	Snx5	0.0004877	7.9768228
93599_at	2500003M10Rik	0.0000023	8.8082197
93600_at	Obrgrp	0.0000655	7.4404747
93604_f_at	Igsf4	0.0000708	9.8194334
93609_at	Spg20	0.0016809	5.6565553
93611_at	Tbx6	0.0000654	7.7398574
93612_at	Mmp15	0.0004878	5.9628861
93615_at	Pbx3	0.0000439	9.0904575
93616_g_at	Pbx3	0.0016284	7.9058939
93620_at	Rpo1−4	0.0000126	8.2276845
93621_at	5730599O09Rik	0.0034293	8.1421319
93626_at	Abcg2	0.0025288	8.3080024
93627_at	E430019N21Rik	0.0034293	7.0539572
93630_at	Cugbp1	0.0002565	5.9480855
93632_g_at	Lbcl1	0.0008730	6.8603982
93634_at	Fbxw1b	0.0000210	7.3352650
93642_at	Gdf1	0.0000344	7.2388922
93646_at	Ptk9	0.0028038	4.1129130
93655_at	Usf1	0.0088930	6.7683049
93656_g_at	Usf1	0.0112863	6.8665911
93669_f_at	Sox11	0.0000318	8.4039299
93670_at	Erf	0.0003424	7.2740706
93674_at	Fgd1	0.0012569	6.3913443
93676_at	Rad51ap1	0.0000228	8.0389535
93682_at	Ldb2	0.0021644	6.8079323
93699_at	Polg2	0.0072900	5.8410326
93701_at	Smarca5	0.0000009	7.3649676
93702_at	AI462446	0.0099678	6.9261130
93704_at	Foxf1a	0.0000040	5.7222723
93706_at	Ikbkb	0.0036382	5.7160849
93711_at	Sec23a	0.0017358	5.8964275
93714_f_at	H2−K	0.0004878	5.5232990
93718_at	Rab23	0.0000929	5.1648710
93720_at	Agpat1	0.0000828	7.5191813
93721_at	Cap1	0.0146787	6.7954602
93727_at	Gspt1	0.0000193	10.2245061
93728_at	Tgfb1i4	0.0000015	8.3471955
93730_at	Syn1	0.0000082	12.3051283
93731_at	Fkbp9	0.0001217	8.3902617
93732_f_at	Rgs19ip1	0.0000058	7.7913272
93733_r_at	Rgs19ip1	0.0017358	6.1742678
93735_f_at	Psmc3	0.0000895	9.1639246
93740_at	Nsep1	0.0025288	12.6223862
93741_at	C87860	0.0000016	7.4414557
93742_at	5730449L18Rik	0.0059802	7.3288390
93743_at	Hsbp1	0.0000654	6.5508477
93748_at	Grinl1a	0.0000034	10.1089056
93749_at	Maoa	0.0000293	8.0246273
93750_at	Gsn	0.0020324	6.5622291

93751_at	8430421I07Rik	0.0007895	8.5301109
93752_at	E430001P04Rik	0.0001647	9.3815097
93753_at	Litaf	0.0040969	6.7602267
93757_at	1300011P19Rik	0.0008446	9.3455007
93758_at	Incenp	0.0002966	8.2844342
93762_at	Ppp2r4	0.0011014	9.1669419
93764_at	Grim19−pending	0.0001528	8.2515188
93767_i_at		0.0001304	10.1071353
93768_f_at		0.0000538	9.6482833
93772_i_at	Zfp265	0.0000081	8.6103810
93773_f_at	Zfp265	0.0000026	8.9860897
93780_at	Them2	0.0000069	6.6253960
93782_at	Rnf4	0.0000028	9.9305459
93784_at	Cfdp	0.0000013	9.1761682
93785_at	Folr1	0.0001647	7.7625334
93786_i_at	Mrpl18	0.0000222	8.8344103
93787_f_at	Mrpl18	0.0003656	10.0984054
93789_s_at	Sin3b	0.0000010	8.2958037
93793_at	Lasp1	0.0000895	8.4336650
93794_at	Appbp1	0.0000009	8.7289085
93795_at	Itpa	0.0003949	8.0357132
93797_g_at	Atp1a1	0.0000010	9.2886230
93798_at	Atp1a1	0.0000018	9.8275355
93803_at	Psme3	0.0000013	8.9648529
93806_at	Sh3bgrl	0.0000048	8.3354839
93812_at	Clns1a	0.0000023	9.4896244
93815_at	0610041L09Rik	0.0005607	9.1309751
93818_g_at	Rnp24−pending	0.0004878	8.7855018
93819_at	Rnp24−pending	0.0000345	9.9603046
93821_at	D8Ertd594e	0.0000013	6.2741689
93823_at	Ilf2	0.0000075	8.2574080
93826_at	Ppp2r5a	0.0000269	8.0039157
93829_at	Rod1	0.0000016	6.9533385
93830_at	Nono	0.0000895	10.7768185
93831_at	Nono	0.0000063	8.1073651
93834_at	Hist1h2bc	0.0046120	5.9220309
93836_at	Bnip3	0.0000053	7.6754337
93839_at	Rtn3	0.0068924	8.5814029
93840_at	Apom	0.0001911	8.7341009
93841_at	D3Ertd194e	0.0000015	7.7894928
93842_at	Dap	0.0072900	7.6720093
93843_at	Dhrs1	0.0002759	7.9630451
93845_at	Abcf2	0.0002565	8.8179035
93851_at	Rabggta	0.0139338	6.5806944
93852_at	Mef2a	0.0061552	6.0360536
93857_at	Kpna3	0.0015264	5.5395810
93858_at	Cxcl10	0.0057230	2.1410034
93859_at	Mtif2	0.0000040	6.9127600
93868_at	Nsdhl	0.0032287	5.9441755
93870_at	Trim24	0.0010312	4.6798868
93877_at	2210419D22Rik	0.0005414	8.0017022
93878_at	Mllt10	0.0003548	5.9210474
93880_at	Eomes	0.0000012	6.1905393
93889_f_at	Hist1h2ba	0.0006888	4.7251316
93900_at	Bat2	0.0004548	5.6719434
93902_at	Gab1	0.0086090	5.7771135
93907_f_at	LOC280487	0.0000090	10.6071567
93908_f_at		0.0051821	7.9447356
93909_f_at		0.0004239	9.6228144
93912_at	Bat3	0.0003949	7.6118430
93916_at	Smyd5	0.0036404	6.0165745
93918_at	Taf9	0.0000126	8.2007142
93919_at	Nrk	0.0001225	4.7095093
93921_at	Bat3	0.0051821	8.2930005
93922_g_at	Bat3	0.0000853	8.7696789
93923_at	E030022H21	0.0003187	7.9995532
93924_f_at	Tuba7	0.0000374	8.0205302
93935_at	Nlk	0.0011025	6.2154533
93937_at	Gas8	0.0003187	6.5258780
93941_at	T	0.0000063	9.4335228
93943_f_at	Zfp36l2	0.0139359	3.6472774
93944_r_at	Zfp36l2	0.0061541	7.2087292
93950_at	Foxa2	0.0000439	6.9786339
93951_at	Golga3	0.0072900	6.4062735
93953_at	Prss12	0.0000629	6.6129319
93958_at	Rnf14	0.0047487	5.7564194
93962_at	Rap1a	0.0013416	5.7150407
93964_s_at	Ddx6	0.0038464	7.9089459
93966_at	Ube4b	0.0086090	8.3985860
93967_at	Anapc11	0.0058129	9.0122252
93970_at	Ipo7	0.0000034	7.5342740
93971_f_at	Psmd12	0.0000178	8.9643056
93972_at	Ndufs2	0.0003949	9.4933018
93973_at	Eif3s9	0.0132266	10.5248285
93976_at	Cab39	0.0000044	7.6752995
93980_at	2600016B03Rik	0.0000034	6.8770979
93982_at	1110021N07Rik	0.0119030	7.5729022

93984_at	Atpi	0.0001044	10.3173825
93985_at	AW558171	0.0051811	5.5363890
93986_at	2410003A14Rik	0.0000009	9.1597914
93987_f_at	3110001N18Rik	0.0002216	11.8168024
93990_at	Hnrph1	0.0000040	10.9255144
93991_at	Mor1	0.0000269	10.5511318
93992_at	5430437P03Rik	0.0000708	8.0581998
93993_at	Lman2	0.0042231	8.6634741
93994_at	Chpt1	0.0093423	4.2575448
93997_at	Ifrg15−pending	0.0000069	7.6902237
93999_at	Snrpg	0.0000318	10.8804621
94001_at	Elavl1	0.0000008	10.1279680
94002_at	Cul1	0.0000090	8.9582274
94003_at	Prkwnk1	0.0000009	8.9445442
94005_at	3110004O18Rik	0.0000020	6.8854287
94007_at	Morf4l1	0.0000008	11.1272608
94008_at	2610301I15Rik	0.0004548	7.7121388
94010_g_at	2610301I15Rik	0.0012162	5.4787699
94015_at	Th1l	0.0119030	7.8977222
94017_s_at	Sfrs2	0.0000021	12.2943130
94019_at	Bzw1	0.0000010	8.6148946
94020_at	Ptk9l	0.0003188	7.5264208
94021_at	Trim11	0.0106982	7.4820510
94022_at	Gltscr2	0.0022336	8.5271560
94024_at	Ris2	0.0000012	9.5550831
94025_at	Psmb3	0.0003303	10.0889915
94030_at	D3Ertd176e	0.0002966	7.9723805
94031_at	Rab2	0.0000210	8.2296860
94034_at	Smfn	0.0000063	6.7627075
94036_at	Cdc42ep4	0.0007377	7.6548829
94038_at	2700060E02Rik	0.0006739	9.5063686
94040_at	Erh	0.0000031	11.5019739
94041_at	Hnrpk	0.0000126	9.4807914
94042_f_at	Gng5	0.0036411	11.8965324
94043_at	Atp6ip1	0.0015272	7.6557897
94044_at	D11Ertd530e	0.0000516	7.6273187
94046_at	Csk	0.0119030	7.3244893
94048_at	Cdc34	0.0000178	9.6396879
94052_at	Dpm2	0.0004878	7.9685878
94055_at	Cttn	0.0001127	6.4500384
94056_at	Scd1	0.0006892	7.6898743
94057_g_at	Scd1	0.0090924	6.8140180
94062_at	Ndufv2	0.0003188	9.9403441
94064_at	Zfp91	0.0005607	6.5879430
94066_at	Rnf14	0.0018496	7.8450021
94067_at	Dcps−pending	0.0000605	7.9807399
94068_at	Rps19	0.0001470	12.0817349
94071_at	Gosr2	0.0000655	7.6618057
94072_g_at	Gosr2	0.0009656	7.7018820
94073_at	Polr2g	0.0004239	9.7630951
94076_i_at	Rpn2	0.0000188	8.2272288
94077_f_at	Rpn2	0.0008446	9.8948950
94080_at	Sdha	0.0009030	6.4671811
94081_at	2700027J02Rik	0.0000405	9.9443491
94084_at	2010012C09Rik	0.0002216	8.3625628
94088_at	Ptbp2	0.0000082	7.4693519
94099_at	Nptx2	0.0001527	6.6383460
94105_at	Cdc42	0.0000075	9.1936716
94106_at		0.0002660	7.9444282
94109_at	LOC226442	0.0000106	7.7510889
94110_f_at	LOC234358	0.0000210	7.8381492
94111_r_at	LOC234358	0.0000126	7.2635049
94114_at	Hist1h1a	0.0019694	5.1558845
94124_at	Top3a	0.0002058	6.8868913
94126_at	Wnt2b	0.0154561	2.5303080
94135_at	Tcfcp2	0.0077054	5.8566971
94163_at	Atp4a	0.0050322	6.6410019
94174_at	Catnal1	0.0162601	4.7595808
94189_at	Bcl6b	0.0053896	4.4357087
94192_at	Pbef−pending	0.0002216	7.3018886
94196_at	Ikbkg	0.0065145	5.9096603
94197_at	Ugcg	0.0132246	5.4361290
94200_at	Gbx2	0.0000138	7.8196351
94211_at	6720460F02Rik	0.0041002	7.5449542
94216_at	Sdhc	0.0000079	9.3512063
94217_f_at	Cdca3	0.0000269	6.8411949
94218_at	Tcp1	0.0034293	7.6433457
94219_at	Psmb2	0.0001128	9.6717029
94223_at	Net1	0.0000605	5.8800039
94225_at	Apg5l	0.0036397	7.1526774
94228_at	Xpo1	0.0000034	9.2304775
94229_at	0610009M14Rik	0.0011025	7.7649180
94232_at	Ccnd1	0.0000053	8.6392486
94233_at	1110038F14Rik	0.0072900	7.4275511
94235_at	Gtl6	0.0019703	6.3613972
94236_at	Nisch	0.0000048	10.3349275
94239_at	Pnn	0.0000027	7.8298707

94241_at	Coasy−pending	0.0000201	5.8549956
94242_at	D11Ertd672e	0.0004548	8.4164412
94243_at	4930432B04Rik	0.0000605	8.2208652
94247_at	5730453H04Rik	0.0032287	8.1904942
94248_at	Ap1m1	0.0000023	8.2124921
94250_at	Eif3s10	0.0002385	9.2772547
94252_at	Eif2s3x	0.0001774	10.6283769
94253_at	Eif2s1	0.0000082	9.9745572
94257_at	Rraga	0.0000116	8.5616811
94259_at	Tebp−pending	0.0000008	9.8252645
94260_at	3110040D16Rik	0.0000013	9.6715084
94262_at	9330151F09Rik	0.0000193	7.7346987
94263_f_at	Psmb7	0.0005231	9.9020644
94264_at	Raf1	0.0000405	8.5121364
94273_at	2610311I19Rik	0.0000163	7.8581030
94274_at	6720465F12Rik	0.0000010	9.4215216
94276_at	Hsd17b12	0.0007895	8.4808450
94277_at	Mtx1	0.0006892	7.8543699
94278_at	Lcp1	0.0022336	6.7202933
94281_at	Cnot2	0.0000150	8.5301368
94282_at	Asah1	0.0022331	6.4194082
94286_at	9130011J15Rik	0.0000008	6.9167839
94288_at	Hist1h1c	0.0016284	6.2484278
94289_r_at	Maged2	0.0009656	7.0029409
94290_at	Lobe−pending	0.0009030	6.9443850
94294_at	Ccnb2	0.0000193	10.1261106
94295_at	Gtf2i	0.0000126	10.2454919
94296_s_at	Gtf2i	0.0000063	9.1978415
94297_at	Fkbp5	0.0004878	8.0930245
94299_at	2310042E05Rik	0.0008446	7.7567962
94300_f_at	2310042E05Rik	0.0000655	7.3958300
94301_at	Atp6v0e	0.0015272	8.3871589
94302_at	Psmd4	0.0038649	9.8490644
94303_at	Hnrpd	0.0000034	10.6951815
94305_at	Col1a1	0.0004239	6.1284318
94308_at	Fbln1	0.0038649	8.6296849
94309_g_at	Fbln1	0.0061552	9.6163549
94312_at	AA415817	0.0003678	6.8671841
94313_at	Snrp1c	0.0050332	9.1101228
94319_at	Rab18	0.0000405	8.8087599
94322_at	Sqle	0.0012165	8.5038948
94323_at	Nutf2	0.0000009	11.3775182
94325_at	Hmgcs1	0.0000021	8.0582530
94330_at	Npl	0.0000559	6.6020754
94331_at	Stat6	0.0003678	6.3377150
94335_r_at	Ina	0.0098638	7.0961552
94340_at	1110004L07Rik	0.0000026	7.9482448
94341_at	Jmj	0.0004238	9.1057461
94345_at	Il6st	0.0000034	6.3424189
94346_at	Wtap	0.0000178	7.3586979
94352_at	Mbtd1	0.0003949	8.1416251
94355_at	Trp53bp1	0.0009973	3.5461560
94356_at	Trp53bp1	0.0028592	7.6984436
94359_at	AA960558	0.0038649	8.0192348
94360_at	2700029M09Rik	0.0000026	8.2127453
94361_at	Ddx21	0.0000026	9.4487465
94362_at	Nras	0.0000034	9.8142426
94363_at	BC030906	0.0001647	7.4104016
94364_at	Glg1	0.0000008	8.4393547
94366_at	2310079N02Rik	0.0000269	6.2309665
94367_at	Uck2−pending	0.0000010	9.5357109
94368_at	Supv3l1	0.0003678	7.0553682
94369_at	Gnpnat1	0.0000655	7.5188435
94375_at	Hk2	0.0003425	9.3515904
94376_s_at	Mre11a	0.0000654	7.0973275
94380_at	Ide	0.0023765	6.6959850
94382_at	1110057K04Rik	0.0070875	7.4645808
94384_at	Ier3	0.0001313	5.4150252
94386_at	Son	0.0026894	8.4144375
94387_at	Spata5	0.0000079	6.3507258
94388_at	Ap3s2	0.0000082	6.6775692
94390_at	Akap8	0.0000605	7.2778075
94392_f_at	Ang	0.0004390	4.6958997
94393_r_at	Elovl2	0.0033269	5.5344203
94397_at	1200014O24Rik	0.0000708	7.6934924
94399_at	Inpp5b	0.0146787	6.5501155
94403_at	AU043625	0.0007377	7.2685503
94405_at	Slc6a6	0.0017358	4.8329855
94407_at	B3Gat3	0.0139359	6.6128750
94410_f_at	2700094L05Rik	0.0034293	8.7640748
94412_at	Cdk2	0.0000012	7.4546760
94413_at	E130307D12	0.0000021	8.7454094
94415_at	6230421P05Rik	0.0000063	7.7254750
94420_f_at	Cry1	0.0001417	7.9204800
94422_at	KIAA0678	0.0000031	6.8925358
94424_at	AU022220	0.0000028	7.0779372
94426_at	6330575P11Rik	0.0000028	9.2024770

94427_at	Copg1	0.0000055	7.3005333
94429_at	Eef1a2	0.0026894	5.9092366
94430_at	2410003H12Rik	0.0119030	7.0140871
94432_at	Siat1	0.0010320	7.1542528
94433_at	Slc38a2	0.0000210	8.8009004
94434_at	Zfp95	0.0081460	6.4854000
94435_at	D10Ertd438e	0.0006217	7.2541955
94438_at	Pfkm	0.0000828	7.2997105
94439_at	Osbpl11	0.0000930	6.4888193
94445_at	Pls3	0.0000009	8.8449878
94448_at	Bcl10	0.0000150	7.4061531
94450_at	D13Wsu123e	0.0000150	9.2997398
94451_at	D13Wsu123e	0.0000016	8.7501389
94452_g_at	D13Wsu123e	0.0000008	9.8372764
94454_at	Dazap2	0.0000269	7.2811135
94455_at	1010001J12Rik	0.0000228	10.0193855
94456_at	Set	0.0007377	10.6963087
94457_at	Ubce7ip3−pending	0.0000018	8.5091454
94461_at	Pbef−pending	0.0026894	7.3617438
94462_at	Eif2b1	0.0079236	8.6025100
94463_at	Clcn3	0.0000098	6.5068466
94464_at	Clcn3	0.0000009	7.7966532
94465_g_at	Clcn3	0.0000008	7.0815464
94466_f_at	Cebpa−rs1	0.0000374	9.1752866
94469_at	2410018D16Rik	0.0000031	8.6634867
94470_i_at	Slc30a5	0.0079705	6.3182492
94471_r_at	Slc30a5	0.0054898	6.3536731
94473_at	1810010L20Rik	0.0032287	8.1467380
94476_at	4930553M18Rik	0.0000019	5.5817991
94478_at	Rab5a	0.0000028	8.0476553
94480_at	D1Ertd161e	0.0086090	5.8927790
94481_at	Ugp2	0.0034279	4.9571079
94482_at	Csnk2a2	0.0000895	7.6654181
94483_at	Csnk2a2	0.0000019	8.9107864
94484_at	Hbs1l	0.0001911	8.6995384
94485_at	Peci	0.0008950	6.9235207
94486_at	AA959742	0.0001217	9.9067015
94488_at	1110059P08Rik	0.0006436	7.2652007
94489_at	Ptp4a1	0.0000026	10.2513954
94490_at	Cnot8	0.0054888	6.8015522
94492_at	Dstn	0.0023770	10.7694728
94493_at	Cldn3	0.0074951	5.2689159
94494_at	Farsl	0.0000013	7.8326120
94499_at	Mgea5	0.0000116	9.0683725
94503_at	Mel	0.0000008	9.1142032
94506_at	Cpsf5	0.0000008	9.0975140
94507_at	Facl2	0.0000138	4.8744434
94508_at	1810020E01Rik	0.0006890	8.3425518
94509_at	Ncbp2	0.0000031	7.7048623
94510_at	2610312E17Rik	0.0004878	10.0564317
94514_s_at	Arcn1	0.0000895	7.6741622
94518_at	0610033H09Rik	0.0000476	9.1216460
94524_at	Dap3	0.0000516	7.1742978
94528_at	Nubp1	0.0000012	7.3636298
94530_at	C85417	0.0030388	7.6402526
94531_at	2310005O14Rik	0.0000629	6.2099007
94532_at	Atp6v1e1	0.0011775	8.1581373
94534_at	Idh3a	0.0013552	7.1034684
94536_s_at	2900073G15Rik	0.0106982	10.0341470
94542_at	Mbtd1	0.0002058	6.8250843
94548_at	1200003O06Rik	0.0004043	9.4642393
94549_at	1200003O06Rik	0.0010314	8.2049725
94550_at	Snx1	0.0000034	7.7639099
94552_at	Pcbp1	0.0000048	11.5278980
94556_at	Snx10	0.0106965	6.5116620
94558_g_at	Gtf3a	0.0000374	8.0863551
94559_at	Gtf3a	0.0000269	7.2211561
94562_at	Gnpat	0.0000011	8.2200678
94641_at	Cdh10	0.0101375	2.8966710
94642_at	Gdi2	0.0090924	2.3666376
94662_at	4833441D16Rik	0.0000012	6.5331470
94687_at	Foxb1	0.0001528	5.3154368
94689_at	C79248	0.0000018	8.0742054
94713_at	Myo7a	0.0065145	6.7879602
94738_s_at	Defcr−rs2	0.0000605	5.8582422
94747_at	Csf2rb1	0.0068924	2.6542293
94754_at	Lhx8	0.0139359	3.1764376
94766_at	Eef1a1	0.0000023	13.4434026
94768_at	Rad21	0.0000605	9.9527958
94769_at	Mmp8	0.0040931	2.5804399
94784_at	D030034H08	0.0002758	7.1263500
94788_f_at	Tubb5	0.0000009	12.3697285
94789_r_at	Tubb5	0.0000044	12.4454878
94802_at	MGC79213	0.0000023	6.6575229
94804_at	Pbx1	0.0000708	7.5742185
94805_f_at	Hist1h2ac	0.0001417	13.3941898
94806_at	−−−	0.0046120	8.9400444

94807_at	Slc25a1	0.0022336	8.4223071
94810_at	Ewsh	0.0000034	10.5453520
94811_s_at	Gtf2h1	0.0008164	7.6426043
94812_at	Gtf2h1	0.0000828	7.1814894
94814_at	Gnai3	0.0000516	10.3690127
94818_at	Ogt	0.0000069	7.9581603
94821_at	Xbp1	0.0005607	7.6602050
94823_at	BC029892	0.0000269	12.5815653
94826_at	Itgb4bp	0.0000193	9.5284065
94829_at	1110020A09Rik	0.0048900	7.5578264
94830_at	8030460C05Rik	0.0002044	7.2492569
94831_at	Ctsb	0.0000098	9.7293495
94833_at	Fstl	0.0020256	7.6716095
94834_at	Ctsh	0.0000026	8.8503202
94835_f_at	Tubb2	0.0058129	7.2869896
94837_at	Eif3s8	0.0000269	11.0609234
94840_at	Hexa	0.0007377	7.5516760
94841_at	Psma5	0.0025288	9.9275354
94842_at	Blmh	0.0001911	9.3577909
94845_at	5730454B08Rik	0.0154561	8.8803873
94850_at	0610041P13Rik	0.0000318	6.3633923
94852_at	Glul	0.0003423	6.8533305
94853_at	Gnb1	0.0000178	8.0626205
94854_g_at	Gnb1	0.0038649	8.9636036
94855_at	Phb	0.0002385	7.4188461
94860_at	Timm17a	0.0025288	9.1873032
94861_at	4930453N24Rik	0.0072887	5.8358055
94865_at	BC002236	0.0000132	7.5431953
94868_at	Qars	0.0025288	9.4999633
94869_at	Aebp2	0.0046129	6.5177010
94871_r_at	2310075M17Rik	0.0001527	6.8428052
94875_at	Mrpl20	0.0000011	8.1719025
94876_f_at	Gorasp2	0.0065145	9.4711595
94877_at	Btbd1	0.0023037	4.7646430
94881_at	Cdkn1a	0.0000708	5.3239025
94885_at	Unc84a	0.0046129	7.3229334
94889_at	Vapa	0.0000082	10.4737938
94895_at	9430020E02Rik	0.0001217	8.2827130
94896_at	Hnrpab	0.0000967	9.1940497
94898_at	2010004P11Rik	0.0001044	7.1527685
94899_at	Rhoip3−pending	0.0000150	9.4537147
94903_at	3930401E15Rik	0.0012572	9.5358532
94909_at	Mrps17	0.0001313	8.6186852
94910_at	Nde1	0.0000048	8.7008710
94912_at	Mrps21	0.0007895	9.1190490
94917_at	Fbxo8	0.0000053	5.0835141
94918_at	Aars	0.0002759	8.2116419
94920_at	AI415282	0.0150607	6.1747405
94922_i_at	4930431L18Rik	0.0005521	6.2139509
94923_f_at	4930431L18Rik	0.0000031	9.9076738
94925_at		0.0018496	7.8317863
94929_at	Ptpn1	0.0009978	8.3715058
94931_at	1810045K17Rik	0.0004709	7.1012555
94932_at	Pdgfa	0.0068924	8.2279609
94935_at	Tbl1x	0.0009654	5.8101438
94937_at	Zfp277	0.0068912	6.0785696
94941_at	Eif2ak4	0.0065133	5.4285942
94942_at	Cstf1	0.0000292	8.3537386
94946_at	Map3k3	0.0038649	6.5651973
94947_g_at	Map3k3	0.0000026	6.9704387
94951_at	1810030A06Rik	0.0030388	8.1212493
94953_at	Racgap1	0.0007377	7.7326204
94954_at	D5Ertd249e	0.0000210	8.5843053
94955_at	5530600A18Rik	0.0034286	4.9656938
94963_at	Vcl	0.0015272	8.6719881
94966_at	G6pdx	0.0002043	7.0745388
94967_at	D19Wsu12e	0.0003678	7.5045856
94968_at	Nfyc	0.0000476	8.8196531
94969_at	Nfyc	0.0139359	7.5413205
94970_at	C230060M08Rik	0.0004547	5.9691109
94975_at	Ireb2	0.0000138	7.5268941
94976_at	AL022610	0.0000013	8.4346284
94978_at	2310037I24Rik	0.0000228	9.1013235
94979_at	BC018507	0.0000228	7.8380523
94980_at	Dusp11	0.0022331	6.3254617
94983_at	9030624B09Rik	0.0000037	6.1031510
94985_at	Nsap1−pending	0.0000058	8.3502133
94989_at		0.0000021	8.6513114
94992_at	Copb1	0.0000193	8.0306201
94995_at	A030007L17Rik	0.0125493	6.2345153
94998_at	Rala	0.0022331	6.1941136
95000_g_at	Cubn	0.0000015	10.4721337
95001_at	Akap8	0.0001044	8.1438929
95002_at	D17Wsu92e	0.0003678	7.9004919
95003_at	Mt1a	0.0007377	7.4053552
95004_at	Luc7l	0.0018496	7.8381975
95007_at	Tpr	0.0004390	5.8793240

95014_at	Fbxo6b	0.0072900	7.3524200
95016_at	Nrp	0.0000008	8.1333059
95021_at	C130008N12	0.0000008	10.1723588
95022_at	Akap12	0.0095968	6.5592846
95028_r_at		0.0000210	8.1903198
95029_at	Cgi152−pending	0.0000030	8.2024477
95031_at	1110059H15Rik	0.0000263	4.3380377
95032_at	Prc1	0.0000053	8.4636363
95033_at	Jmjd1	0.0000053	7.7338824
95034_f_at	Ipo4	0.0012658	6.8207907
95035_at	Ipo4	0.0000559	8.9716693
95037_at	Cdk9	0.0011023	8.0652377
95040_at	Pdcd6ip	0.0004548	5.5754224
95044_at	Eg1−pending	0.0002759	7.8591598
95049_at	Snrpd2	0.0023770	11.0570672
95050_at	Chordc1	0.0012572	8.3134036
95053_s_at	Sdhb	0.0072900	9.3200102
95054_at	Tars	0.0000210	8.8526631
95057_at	Herpud1	0.0002216	7.0060179
95058_f_at	2610205H19Rik	0.0000515	5.3609021
95061_at	Bcas2	0.0000655	6.4927920
95063_at	2310021G01Rik	0.0007377	9.8693102
95064_at	Acaa2	0.0001774	8.6787900
95066_at	Taldo1	0.0018492	8.4669069
95067_at	Mrpl2	0.0002759	7.8754450
95068_at	Dnaja2	0.0000015	7.3372926
95069_at	Ssrp1	0.0000010	10.6681599
95070_at	Nars	0.0000170	7.1698466
95072_at	Cyc1	0.0000228	11.4119679
95074_at	Pxf	0.0053329	7.0007214
95076_at	1500032L24Rik	0.0008446	9.2137647
95077_at	Rabggtb	0.0000044	8.2478098
95084_f_at	Grhpr	0.0001264	8.8559060
95086_at	1110002H14Rik	0.0000009	9.6930950
95091_at	Sec13r	0.0011025	9.1360502
95092_at	Ppp3ca	0.0000037	6.4775220
95093_at	2610511G16Rik	0.0003425	6.6351005
95094_g_at	2610511G16Rik	0.0000967	6.6591718
95096_at	Qk	0.0000075	9.5640318
95097_at	Actr10	0.0000012	7.8883072
95100_at	Anapc5	0.0000708	10.5029722
95101_at	Tde1l	0.0158536	5.3886106
95103_at	2310065K24Rik	0.0000013	8.2786052
95109_at	Nol5a	0.0000008	10.4324578
95110_at	Ppil2	0.0001177	7.1951656
95117_at	Igf2r	0.0004545	8.4715138
95118_r_at	Kif22	0.0010320	10.0418675
95119_at	1110038D17Rik	0.0012572	7.0041316
95123_at	4930566A11Rik	0.0000193	5.9780684
95124_i_at	Rbx1	0.0001020	8.8527924
95128_at	Anapc2−pending	0.0000178	8.4369276
95131_f_at	Ndufb2	0.0004877	9.8110034
95134_at	3110038L01Rik	0.0111567	7.0150870
95135_at	3110038L01Rik	0.0000013	8.1380836
95137_at	1810014L12Rik	0.0000476	9.5769934
95138_at	1110018O08Rik	0.0022336	7.5229251
95139_at	1110018O08Rik	0.0068924	7.1291236
95140_at	5230400G24Rik	0.0000040	7.6213962
95142_s_at	Capzb	0.0000605	9.0025179
95147_at	Pgls	0.0000010	8.8022222
95148_at	Ak2	0.0000090	8.5183742
95149_at	Copz1	0.0007895	9.0029486
95153_at	2810404F18Rik	0.0000016	7.6141019
95155_at	Zrfp1−pending	0.0000736	7.2483591
95156_g_at	Zrfp1−pending	0.0000019	4.8106585
95158_at	H2−Ke2	0.0077080	9.4407488
95159_at	Mrps18b	0.0000269	8.3777016
95161_at	D10Ertd73e	0.0054898	8.8725127
95184_f_at		0.0039805	6.1465044
95215_f_at	Ubc	0.0006436	11.3805055
95232_at	Hnrpl	0.0000025	9.5975555
95282_at	Hspca	0.0000011	12.7518413
95285_at	KRIM−1	0.0003425	6.0520600
95287_at	Luc7l2	0.0000036	6.5397658
95288_i_at	C330012F17Rik	0.0006867	5.0645571
95297_at	Hoxa1	0.0000015	8.8458165
95317_at	Bsn	0.0043493	4.2279148
95318_at	Zfp105	0.0001217	8.0702602
95335_at	Cx3cr1	0.0166877	5.7997742
95341_at	Tbrg4	0.0000708	7.7592855
95350_at	Ttr	0.0000075	7.7402252
95351_at	Ncoa6	0.0004089	4.9639358
95356_at	Apoe	0.0000405	11.8687161
95357_at	1200009I24Rik	0.0000708	5.3872251
95359_at	Hspcb	0.0000048	12.9638424
95363_at	Gzmm	0.0162695	3.2889235
95364_at	Gna14	0.0117931	4.4106915

95383_at	Tm7sf2	0.0077067	6.9094080
95393_at	Btbd3	0.0009032	5.4761336
95395_at	9130022A11Rik	0.0001128	6.9631691
95400_i_at	Helb	0.0002153	7.7921681
95401_at	MGC47289	0.0090939	7.9758255
95406_at	1810037I17Rik	0.0036411	9.1331587
95408_at	2310003F16Rik	0.0000516	9.2315771
95409_at	1110019J04Rik	0.0000178	9.1862674
95411_at	Smacp−pending	0.0000318	9.3001203
95412_at	Pdcd6	0.0000178	7.8554781
95413_at	6030432N09Rik	0.0000009	8.9958222
95416_at	Usp15	0.0000163	7.9713845
95418_at	1190017B18Rik	0.0001617	5.2666085
95420_at	Pgd	0.0017358	8.6971731
95423_at	Cai	0.0000106	8.5540701
95424_at	Smt3h1	0.0000037	11.0719146
95425_at	Acadl	0.0020972	4.7217780
95427_at	Rpa1	0.0000090	9.3860926
95430_f_at	D9Wsu18e	0.0066999	5.3326050
95431_at	D16Ium22e	0.0004543	7.4173788
95432_f_at	D16Ium22e	0.0000048	8.9861596
95433_at	Ddx54	0.0000228	7.8321823
95435_at	2610313E07Rik	0.0001911	6.4429605
95437_at	Cop1−pending	0.0001527	7.6394698
95438_at	Arht1	0.0000828	6.5022101
95441_at	Timm23	0.0001128	10.6162226
95444_at	4930579A11Rik	0.0106982	8.5457016
95445_at	4833439L19Rik	0.0000248	8.2218427
95446_at	6330577E15Rik	0.0000082	9.3903393
95447_at	1810034K20Rik	0.0002660	7.3392118
95448_at	Psmc2	0.0000021	9.4564799
95451_at	2810405J04Rik	0.0000013	8.9429320
95453_f_at	S100a1	0.0086076	5.4214246
95456_r_at	Shfdg1	0.0001313	10.3522232
95458_s_at	1110001C20Rik	0.0000075	9.3263523
95460_at	Cops5	0.0018496	8.8919520
95462_at	Bzw2	0.0000163	10.3427503
95466_at	Cotl1	0.0017358	5.8315830
95467_at	Scoc	0.0001128	6.8761125
95468_at	Egln1	0.0000008	7.7116763
95470_at	Gdpd1	0.0000178	8.2295254
95472_f_at	Uqcrb	0.0004239	9.7354123
95477_at	ORF18	0.0099642	7.6289492
95479_at	C1d−pending	0.0001417	5.9585750
95482_at	Usp7	0.0000026	9.1429634
95483_at	Psmd1	0.0009032	9.9994788
95485_at	Hadhsc	0.0000098	8.5754567
95486_at	Ptdsr	0.0001128	9.0832492
95488_at	BC010304	0.0000037	8.0973390
95489_at	Fliih	0.0000009	6.8579769
95490_at	Kdelr1	0.0051821	9.2421170
95491_at	Park7	0.0000766	10.0611374
95496_at	5730409F23Rik	0.0028592	8.3868822
95497_at	Tipin−pending	0.0000008	7.5462271
95498_at	Mrps15	0.0006892	6.9593352
95501_at	2410001C21Rik	0.0000605	6.4218970
95505_at	Tor1b	0.0000091	6.3962908
95507_at	Prps1	0.0000193	9.3023139
95508_at	Nckap1	0.0000044	9.6351386
95512_at	Pcmt1	0.0162671	5.9800513
95513_at	Statip1	0.0000106	7.5997348
95514_at	2610510H01Rik	0.0000828	9.0292708
95516_at	Rab9	0.0000015	8.2918142
95518_at	1810015C04Rik	0.0000098	6.2089451
95522_i_at	Zfp68	0.0000125	6.2055287
95525_at	Ncoa6	0.0016039	5.6840475
95526_at	Xpo7	0.0000210	9.3372981
95530_at	6330549H03Rik	0.0000012	7.8697100
95531_at	Amot	0.0000019	10.2337939
95536_at	Tceb3	0.0000138	6.7287751
95539_at	Gtpat12	0.0001911	6.7821351
95540_r_at	Ddx27	0.0093408	5.2738245
95542_at	Tpm4	0.0000013	10.7098616
95543_at	Tpm4	0.0000033	7.5574551
95544_at	2210403N08Rik	0.0106965	5.1827914
95549_at	Prim2	0.0003187	6.5570150
95551_at	1700020M16Rik	0.0006217	7.6351013
95556_at	Mrpl45	0.0000405	8.3144332
95559_at	6330403K07Rik	0.0017358	5.5346584
95561_at	1700013H19Rik	0.0000009	6.6557470
95567_at	2610510E10Rik	0.0000116	6.4769938
95568_at	1500011J06Rik	0.0009656	5.2248861
95573_at	Baz2a	0.0146787	8.4515539
95577_at	AI314180	0.0038649	7.2859400
95580_at	5830417I10Rik	0.0065145	6.7812643
95584_at	Dppa2	0.0025282	3.5165162
95591_at	Extl3	0.0036404	8.4786586

95592_at	1110019N10Rik	0.0000013	8.0763036
95594_at	Mfn1	0.0000405	7.1528096
95601_at	Ubqln1	0.0000345	8.7054988
95602_at	Trpc4ap	0.0000040	9.0247001
95603_at	Gldc	0.0000063	8.9370646
95604_at	C330006A16Rik	0.0003949	7.9740574
95606_at	Nsap1−pending	0.0000034	7.1144529
95608_at	Ctsb	0.0055535	4.8075393
95609_at	Ppp1r15b	0.0000098	8.2153401
95610_at	Cdc5l	0.0000605	6.9817806
95611_at	Lpl	0.0000228	6.0612633
95612_at	Rfc5	0.0000008	8.7971824
95613_at	2010200I23Rik	0.0012165	6.9742720
95614_at	Cbx5	0.0000516	9.0354910
95616_at	Crsp3	0.0000766	6.7103371
95619_at	1700040I03Rik	0.0014314	5.5065555
95622_at	Klhdc2	0.0000559	8.0044388
95628_at	Diap3	0.0000374	5.4993037
95629_at	D11Ertd172e	0.0068924	8.4869747
95631_at	Ppp4c	0.0000476	9.9745367
95634_at	0610010K14Rik	0.0000058	7.5305101
95635_g_at	0610010K14Rik	0.0004878	8.1798718
95637_at	Flnb	0.0009656	8.3735420
95643_at	Wdr6	0.0001044	9.2004194
95646_at	Cpt2	0.0001841	8.6573232
95647_f_at	4022402H07Rik	0.0000075	7.9956135
95648_at	4022402H07Rik	0.0002759	8.0988518
95649_at	Phf5a	0.0002385	8.8749850
95650_at	Ssfa1	0.0048900	10.6602824
95652_at	Vps52	0.0048900	10.9805040
95653_at	Mrpl37	0.0000967	8.0567172
95654_at	Clic1	0.0000069	8.4929086
95655_at	5830411E10Rik	0.0030842	4.9052394
95657_f_at	D13Wsu177e	0.0000405	9.5546841
95659_at	Baf53a−pending	0.0001911	9.1564594
95662_at	X83328	0.0008443	7.6659521
95666_at	9430009J09Rik	0.0000106	8.9472589
95673_s_at	Basp1	0.0000116	11.0933244
95675_at	Map4k3	0.0000037	7.0610874
95677_at	Prp8bp−pending	0.0000021	9.0487803
95681_f_at	Ppp1r2	0.0018496	6.1999313
95682_at	Ddb1	0.0007895	11.0650187
95683_g_at	Ddb1	0.0004548	10.6709413
95685_at		0.0000040	8.3936920
95686_at	Rab14	0.0030376	5.1427048
95688_at	Degs	0.0000476	9.2412092
95689_at	Mtch1	0.0017732	7.5594498
95690_at	1110030L07Rik	0.0000177	8.7369089
95692_at	2610024I03Rik	0.0000015	9.2880122
95696_at	Txnl2	0.0027725	10.4673291
95698_at	Ndufb7	0.0000476	10.0888748
95699_f_at	2010009J04Rik	0.0000012	10.6385542
95700_r_at	2010009J04Rik	0.0000228	9.4946311
95701_at	4930415K17Rik	0.0000044	7.6766903
95703_at	Uble1a	0.0000559	9.4173388
95708_at	D3Ucla1	0.0006436	9.4644391
95709_at	D7Wsu86e	0.0139359	8.6748082
95712_at	Orc6l	0.0000058	9.3683583
95714_at	0610009D07Rik	0.0025288	9.4017347
95715_at	Nfkbib	0.0014318	10.6153233
95716_at	Ywhag	0.0000150	10.8715059
95717_at	Elp3	0.0000374	8.5263583
95722_at	Glrx1	0.0000558	6.9201409
95723_r_at	2610009E16Rik	0.0032287	10.4440395
95726_at	Mlf2	0.0004355	9.1555331
95727_at	Apoa5	0.0109800	4.8527348
95730_at	Mrps34	0.0001313	8.8897135
95731_at	laminin, alpha 4	0.0000292	5.8200057
95734_at	Mrpl3	0.0046129	7.3533173
95735_at	Nolc1	0.0000026	7.4822942
95736_at	Mrpl4	0.0000034	9.1653757
95738_at	Pycs	0.0000655	5.9216872
95740_at	2300003P22Rik	0.0139359	5.8373032
95742_at	Psmd13	0.0001647	9.2448540
95743_at	Paip2−pending	0.0002058	8.5592313
95744_at	Atp6v1a1	0.0003425	8.2833769
95745_g_at	Atp6v1a1	0.0001646	8.1688819
95746_at	Atp6v1a1	0.0000016	9.5563413
95750_at	1110054L24Rik	0.0000058	7.6472874
95753_at	A730011O11Rik	0.0000023	6.9102651
95754_at	Mbtps1	0.0000034	9.3915634
95755_at	Csda	0.0004390	8.9869862
95756_at	Epcs3	0.0000098	7.8545098
95758_at	Scd2	0.0038649	11.2378041
95759_at	2900092E17Rik	0.0022316	7.3346503
95765_at	Adh5	0.0000708	9.5096418
95785_s_at	Rab7	0.0000008	7.6625164

95787_s_at	Scp2	0.0018496	7.1840184
95791_s_at	Sfrs2	0.0000008	10.4971063
95795_at	Supt4h	0.0001646	7.3014043
95800_s_at	Zfx	0.0024513	4.1718749
95801_s_at	Zfp260	0.0005231	5.7287946
95805_at	Cdc2l2	0.0031318	7.3236529
95861_at	D15Ertd55e	0.0088456	4.7215845
95883_at	D530048A03Rik	0.0000026	6.1302669
95885_at	1200009F10Rik	0.0079230	5.0555558
95887_at	8430426K15Rik	0.0017358	6.5762905
95889_at	AI854770	0.0004877	7.2168836
95894_at	AI427100	0.0011392	5.3291877
95896_at	Cox7c	0.0002472	5.6159091
95897_at	Atp2c1	0.0002966	7.5600455
95907_at	Uba52	0.0122205	6.4922889
95910_f_at	C330008I15Rik	0.0011818	6.2242832
95911_at	3010025C11Rik	0.0003188	5.7873580
95912_at	Rnf2	0.0023337	4.9080556
95914_at	6720461J16Rik	0.0001911	6.4629178
95917_at	AA407107	0.0000063	4.3591978
95938_at	C80292	0.0162695	5.7682182
95944_at	Dhx36	0.0000013	7.8844982
95948_at		0.0086076	4.7416881
95964_at	A430103N23Rik	0.0004390	4.7569297
95989_at	C79777	0.0125455	4.4790599
96002_at	Nedf−pending	0.0111528	6.7937693
96003_at	Mta1l1	0.0005414	8.0858350
96007_at	0610038P07Rik	0.0026894	10.4501093
96009_s_at	Pap	0.0034293	5.3417754
96010_at	Kpna3	0.0000040	9.4942418
96011_at	Matr3	0.0000012	8.0241595
96012_f_at	Matr3	0.0000069	11.1549735
96013_r_at	Matr3	0.0004238	5.1695588
96016_at	2700094K13Rik	0.0001774	10.9857344
96018_r_at	Zdhhc5	0.0000708	6.8932072
96023_at	D8Wsu151e	0.0000012	6.3512952
96024_at	Ahcy	0.0000269	10.8064692
96025_g_at	Ahcy	0.0000012	11.3546773
96029_at	Sf3a3	0.0000126	9.5833197
96037_at	Bri3	0.0002565	9.7940926
96038_at	Rnase4	0.0020977	4.4171247
96041_at	Rbm3	0.0000012	10.2683536
96042_at	Sod2	0.0001217	6.2956865
96045_at	2010321M09Rik	0.0168101	6.7378873
96046_at	Hdac1	0.0154561	9.2174587
96047_at	Rbp4	0.0119030	10.0252519
96048_at	Hrsp12	0.0002966	6.0431260
96050_at	Smarcb1	0.0041010	9.3828742
96052_at	Acp1	0.0004878	8.3556490
96053_i_at	Acp1	0.0000159	7.1411463
96054_f_at	Acp1	0.0000516	7.6921337
96057_at	Aldh2	0.0017358	7.2411749
96058_s_at	Aldh2	0.0000177	7.5147279
96059_at	D4Ertd786e	0.0006215	6.5871759
96060_at	Serpinb6a	0.0000040	7.6278524
96061_at	Usp14	0.0000126	8.0188031
96063_at	Xrcc5	0.0000439	7.3952287
96065_at	Lxn	0.0001749	6.0200027
96066_s_at	Pkm2	0.0000015	12.6612280
96069_at	Akr7a5	0.0119030	8.9436276
96072_at	Ldh1	0.0000345	12.8942670
96073_at	Req	0.0009656	8.8775439
96075_at	Wdr1	0.0015272	8.6212743
96079_at	0610010K06Rik	0.0000559	7.5490426
96081_at	Tk1	0.0066999	8.2965763
96082_at	Mrpl30	0.0001416	7.4632918
96083_s_at	Hnrpdl	0.0000075	11.0428038
96084_at	Hnrpdl	0.0000063	8.7185668
96085_at	Gsta4	0.0000177	6.2955853
96086_at	1110031B06Rik	0.0000248	8.1328849
96087_at	Mgat1	0.0043493	7.6320207
96088_at	Ndr2	0.0162695	5.7020650
96092_at	Hp	0.0025288	6.1589210
96094_at	Apoa1	0.0005607	11.0927417
96098_at	Mrpl36	0.0065145	7.2018057
96106_at	2400006P09Rik	0.0000016	7.6235147
96110_at	Cbr1	0.0011025	7.3513057
96112_at	Etfa	0.0000075	7.6493919
96113_at	D18Wsu98e	0.0016284	9.5261063
96115_at	Dp1	0.0048891	8.7234400
96117_r_at	H13	0.0002565	7.8147734
96121_at	1110055N21Rik	0.0001417	8.7644850
96125_at	Daxx	0.0000106	7.1830184
96127_at	Sgpl1	0.0000228	8.0605683
96130_at	Stk2	0.0000405	7.1868804
96131_at	AI447804	0.0006892	6.6224555
96132_at	AB023957	0.0000895	6.7957362

96134_at	Dp1l1	0.0000048	7.7333727
96135_at	3110003A17Rik	0.0000138	8.9073631
96136_at	Pmscl2	0.0008446	5.8763807
96138_at	Mars	0.0000967	9.0390057
96140_at	Pcm1	0.0000126	6.8062470
96148_at	Susp1−pending	0.0000629	6.2414588
96152_at	Narg1	0.0017358	9.2062633
96154_at	Renbp	0.0003948	7.1954870
96155_at	Cdk5rap3	0.0022336	7.8741798
96157_at	Zfp91	0.0004239	8.2808039
96167_at	Bag3	0.0000629	7.0962913
96169_at	Sall4	0.0000516	9.4247918
96171_at	Deaf1	0.0086061	6.9479887
96174_at	Pom121	0.0003678	8.5607200
96176_at	Arih2	0.0125493	7.6583227
96178_at	Myst2	0.0000138	8.6216358
96183_at	Foxp1	0.0132266	6.4402202
96184_at	1110014H17Rik	0.0026894	6.5795770
96185_at	Ap3d	0.0013419	8.5026752
96186_at	Lrp10	0.0018496	7.6296373
96187_at	Pkp4	0.0000150	8.5880305
96188_at	Adar	0.0128801	5.8139663
96189_at	2410141K03Rik	0.0009594	5.5174866
96191_at	D130059B05Rik	0.0002565	8.1552613
96192_at	Sp3	0.0000013	8.2374762
96197_f_at	5730589K01Rik	0.0000028	6.8158846
96200_at	2410018C03Rik	0.0119030	8.6516389
96206_at	D330037A14Rik	0.0024507	6.6979203
96208_at	6430596G11Rik	0.0002216	7.7139135
96212_at	2310061I04Rik	0.0112863	7.7575918
96215_f_at		0.0000293	10.7949207
96217_at	Polb	0.0000063	6.1218240
96219_at	1810031K02Rik	0.0007771	6.4433889
96220_at	Lig3	0.0000090	6.0781964
96222_at	BC003993	0.0112863	7.4489003
96224_at	Btrc	0.0106965	6.6743102
96232_at	Cul2	0.0000228	8.2458089
96234_at	Cpsf3	0.0017358	7.8023318
96236_at	Cdc16	0.0000010	8.6566420
96237_at	SMAF1	0.0030363	3.0771142
96238_at	Rab11a	0.0002216	5.4460940
96239_at	Vrk3	0.0068912	7.0733982
96242_at	BC005632	0.0143009	4.6287097
96243_f_at	Aldh9a1	0.0000013	8.1048076
96249_at	Sep15−pending	0.0027725	9.2807032
96252_at	Pdcd6ip	0.0001417	8.2814758
96254_at	Dnajb1	0.0000015	8.1448081
96255_at	Bnip3l	0.0000008	9.1027100
96256_at	Prdx3	0.0025288	8.5079625
96257_at	Hp1bp3	0.0000828	7.0451975
96258_at	Mgst3	0.0051821	6.8830321
96259_at	Psa	0.0000044	8.4301340
96260_at	Snd1−pending	0.0081474	8.6629984
96261_at	2310028O11Rik	0.0001471	8.7136269
96262_at	Rab5c	0.0001774	7.4786757
96263_at	4930524H12Rik	0.0066999	6.5892215
96264_at	0610008N23Rik	0.0003678	8.1076833
96266_at	Hnrpm	0.0000069	10.3019960
96267_at	Ndufv1	0.0000163	10.1549284
96268_at	Suclg1	0.0001417	8.8974705
96269_at	Idi1	0.0000178	7.7823105
96270_at	D11Bwg0434e	0.0096031	9.0974986
96271_at	2310075C12Rik	0.0001911	7.5424087
96272_at	Ptprf	0.0086090	8.9385623
96281_at	Atp6v1g1	0.0001128	9.3694999
96284_at	AI463719	0.0013419	9.6651387
96288_at	3110038B19Rik	0.0070862	7.2046693
96289_at	Stoml2	0.0065145	9.7428804
96290_f_at	Rpl21	0.0012572	12.2296576
96292_r_at	BC059730	0.0041002	9.5695833
96293_at	2410015N17Rik	0.0002216	10.3878021
96294_s_at	2410015N17Rik	0.0009656	9.2273153
96295_at	Psat1	0.0001417	9.9716822
96296_at	Mrpl15	0.0000098	8.1220566
96297_at	Ebna1bp2	0.0000017	8.0099050
96301_at	Rps27	0.0023765	7.9250953
96302_at	Sfrs7	0.0000013	8.8712816
96307_s_at	1100001I22Rik	0.0001528	12.8526510
96322_at	Edf1	0.0000178	8.6672180
96324_at	Kars	0.0094167	5.1610314
96325_at	2510039O18Rik	0.0000861	7.2458287
96329_at	2410104I19Rik	0.0004548	9.3077924
96331_at	Snx2	0.0000044	7.4413799
96333_g_at	Snx2	0.0000248	8.3477362
96335_at	Mrpl38	0.0034286	8.0724292
96338_at	Egln2	0.0000063	8.5814283
96339_at	Rpl31	0.0081474	2.3499389

96341_at	Gcipip−pending	0.0000895	7.1163347
96342_at	1700006C06Rik	0.0000655	8.3336186
96351_at	5730434I03Rik	0.0001313	7.7488862
96352_at	2400001E08Rik	0.0007377	8.3095975
96353_at	1110021D01Rik	0.0000015	8.3078910
96354_at	Mbnl	0.0025288	5.5018818
96355_at	2900055D03Rik	0.0061552	5.0637000
96358_at	LOC381438	0.0002385	13.0897054
96359_at	Hdlbp	0.0006892	9.9321537
96360_at	Arhgdia	0.0000210	10.8078706
96375_at	cathepsin H	0.0000053	6.6100805
96416_f_at	Hist1h3a	0.0004239	8.0278087
96418_r_at	Hoxb8	0.0083725	6.4502681
96491_at	B130024H06Rik	0.0096015	5.5862420
96493_at	2810002I04Rik	0.0020982	6.1748570
96522_at	Calm1	0.0009656	10.3418217
96530_at		0.0000708	7.8542026
96531_at	Tbl3	0.0000026	8.1368061
96539_at	9330147J08Rik	0.0001128	7.5524750
96545_s_at	A730042J05Rik	0.0000011	6.9178041
96546_r_at	A730042J05Rik	0.0003949	9.9137804
96552_at	2410118I19Rik	0.0036411	7.2312966
96554_r_at	Taf15	0.0001647	4.4166223
96558_at	Mutyh	0.0086090	6.8525514
96560_at	LOC270179	0.0070875	5.6978843
96561_at	Nfatc2ip	0.0018492	5.4921826
96563_at	2810030C21Rik	0.0003810	7.2789893
96564_at	Hspa8	0.0000018	11.6085300
96565_at		0.0128821	6.5374966
96567_at	Rho	0.0077054	2.0267129
96570_at	BC027756	0.0081404	4.8440400
96572_at	Azi2	0.0086076	2.6148453
96573_at	Actg	0.0000026	12.6200789
96575_at	Rpl8	0.0000021	12.7759770
96578_r_at	4932431F02Rik	0.0006892	7.8641823
96579_at		0.0166877	5.2316527
96580_at	Pbx3	0.0009032	7.3963358
96587_at	Arf3	0.0122205	4.2566219
96594_at	Hspa4	0.0001528	10.1391120
96598_at	D430039C20Rik	0.0150607	6.6783843
96604_at	1110032N12Rik	0.0000083	7.7533476
96607_at	Chp−pending	0.0009654	6.8194343
96608_at	Phyh	0.0000228	6.8765347
96609_at	2610019N13Rik	0.0000015	9.7663819
96610_at	Atp6v1h	0.0015268	7.8728821
96613_at	5730536A07Rik	0.0000069	8.3663001
96614_at	4933426M11Rik	0.0012572	6.8883268
96615_at	0610043B10Rik	0.0005231	7.9704964
96616_at	4921525H23Rik	0.0011025	7.9992888
96619_at	Abcf3	0.0020324	5.8529247
96621_at	1110061L23Rik	0.0024513	7.8491614
96623_at	Ugcg	0.0003678	7.7739799
96625_at	D630024B06Rik	0.0000028	7.9362602
96626_at	2300002G02Rik	0.0000031	9.0062997
96628_at	Eprs	0.0025282	8.3821312
96629_at	D7Rp2e	0.0002966	7.4506744
96630_at	Spink3	0.0068900	5.0546691
96632_at	Morf4l2	0.0000967	11.2704832
96633_s_at	Morf4l2	0.0000009	10.0664869
96634_at	5730469M10Rik	0.0000248	7.1061165
96635_at	Gle1l	0.0000516	7.2072070
96637_at	Tbc1d1	0.0004547	7.1277074
96641_at	Noc4	0.0004239	9.0582785
96643_at	1600023A02Rik	0.0101375	7.8004351
96646_at	Sad1−pending	0.0002965	7.2598942
96647_at	4833420K19Rik	0.0000010	8.1946615
96649_at	2310042M24Rik	0.0000011	7.7213322
96651_at	Smarce1	0.0006008	7.3767961
96652_at	Mrpl28	0.0028592	9.1677663
96653_at	0610007O07Rik	0.0000317	8.7904456
96656_at	8430408H12Rik	0.0119030	7.3389955
96658_at	2900010J23Rik	0.0012572	8.7722866
96661_at	Nrd1	0.0023770	8.0524361
96663_at	Surf6	0.0001313	7.1077049
96664_at	Ureb1−pending	0.0000011	9.3789844
96665_at	Vps26	0.0001774	7.8567103
96666_at	Ntan1	0.0051821	5.7388370
96667_at	Vps41	0.0000317	7.5685900
96668_at	Timm17b	0.0166877	9.3227794
96669_at	2400003C14Rik	0.0000053	7.6907906
96674_at	Tnpo3	0.0000053	9.5726559
96676_at	1810049H20Rik	0.0081474	8.0147612
96677_at	2410195B05Rik	0.0000018	8.5932416
96678_at	Dhrs4	0.0000034	6.2000352
96686_i_at	2010100O12Rik	0.0084697	9.0963818
96687_f_at	2010100O12Rik	0.0000294	8.1699132
96693_at	Rars	0.0000044	9.5296449

96695_at	Ube2a	0.0001911	5.4849906
96698_at	Psmd5	0.0000516	8.2347152
96700_r_at	Rap1b	0.0014784	4.0328359
96701_at	Uble1b	0.0000090	10.3373767
96703_at	Maged1	0.0001911	9.6308312
96707_at	Zipro1	0.0000010	8.5142689
96708_at	P24b−pending	0.0051821	6.6028701
96709_at	1110008P14Rik	0.0162695	6.4866500
96711_at	1110014N07Rik	0.0008443	8.5177587
96724_r_at	Ssx2ip	0.0079223	5.6384701
96725_at	Cic	0.0000116	7.1811791
96726_at	Cdk8	0.0000034	8.9042215
96730_at	Tpp2	0.0001774	5.4369274
96732_at	1500001L20Rik	0.0132246	8.2002851
96733_at	Rap1gds1	0.0000028	8.5311945
96737_at	BC023106	0.0013416	6.4820173
96738_at	Adam9	0.0000248	5.9895018
96739_at	1810045K17Rik	0.0061530	5.5986535
96741_at	Phf12	0.0000116	6.7650991
96743_at	1810035L17Rik	0.0000111	9.0807055
96744_at	Acp6	0.0000013	7.3262936
96747_at	Arhu	0.0003678	7.5438142
96750_at	0710007A14Rik	0.0000193	8.5830491
96754_s_at		0.0000012	10.1616232
96755_at		0.0000010	10.3504606
96756_at	1110007M04Rik	0.0124699	6.7880528
96758_s_at	Sec14l2	0.0035323	6.5950531
96760_at	Timm10	0.0004877	5.9067604
96762_at	LOC238799	0.0077080	10.1249473
96765_at	Peg3	0.0000063	11.0632106
96771_at	Erbb3	0.0065133	6.9149025
96772_at	Prim1	0.0000040	7.0831995
96774_at	Plekhc1	0.0096031	7.2998965
96775_at	Cbx1	0.0002759	7.0806571
96777_at	Sf3b1	0.0000178	8.2884892
96778_at	Rrs1	0.0000026	9.0567634
96779_f_at	2410022L05Rik	0.0000011	7.9930594
96780_at	2410022L05Rik	0.0000021	6.6763223
96781_at	AL023001	0.0000053	7.8082480
96784_at	2900037I21Rik	0.0000009	8.5477391
96790_f_at	A530057M15Rik	0.0011775	5.8876847
96792_at	Apob	0.0000008	8.2924516
96793_at	Dmap1	0.0048900	7.0174289
96797_s_at	Fbxw5	0.0001217	8.2859469
96799_at	Fbxw5	0.0002385	6.6842268
96802_at	Pdcd7	0.0119011	6.9975531
96803_at	Gbe1	0.0041010	4.1939030
96804_at	Nol1	0.0000069	9.1085776
96811_at	1700093E07Rik	0.0009646	6.2851186
96813_f_at	DXImx46e	0.0011023	8.6063031
96814_r_at	DXImx46e	0.0128821	5.9043282
96818_at	Dtx2	0.0011775	7.4060820
96822_at	Eif2b5	0.0000040	9.0071114
96827_at	2410008J01Rik	0.0000026	9.2121121
96833_at	Nucks−pending	0.0017358	7.5888105
96834_at	Sfrs9	0.0000069	10.5500643
96836_r_at	Zfp161	0.0013419	3.9488094
96837_at	ORF61	0.0030382	8.6949356
96838_at	Rce1	0.0061552	8.1953284
96840_at	Gabarapl2	0.0000019	8.3727032
96841_at	Pim3	0.0004709	7.8506070
96845_at	2310024J23Rik	0.0001774	6.0220813
96847_at	Vps28	0.0001128	8.4537035
96849_at	Timm8a	0.0007377	8.2570479
96852_at	Prkar1a	0.0000082	9.1229665
96854_at	Copa	0.0000040	9.2836499
96859_at	Rnf10	0.0000163	11.1918096
96861_at	Mrpl50	0.0000106	6.9466223
96862_at	1110002B05Rik	0.0000023	9.0009804
96864_at	AI648866	0.0000010	7.1205128
96865_at	Marcks	0.0000439	8.4937599
96868_at	Fgb	0.0047451	4.5036654
96869_at	Gabarap	0.0007895	8.8980588
96870_at	Aco2	0.0032287	10.3894922
96871_at	2310042G06Rik	0.0000018	7.7956867
96872_at	Sgt	0.0000655	9.5549148
96874_g_at	D6Ertd772e	0.0000516	9.6238393
96876_at	Laptm4a	0.0011025	10.4365901
96878_at	Cyb5m−pending	0.0000015	7.0890351
96879_at	Ogdh	0.0000345	8.4716506
96881_at	1110059J08Rik	0.0038649	7.5036072
96882_at	Ga17	0.0065145	9.9066997
96883_at	Eif3s4	0.0018496	10.9511220
96885_at	2510015F01Rik	0.0005231	10.8768222
96887_at	Np15	0.0009032	9.0583099
96890_at	1300002A08Rik	0.0000015	7.0617377
96891_at	Anp32b	0.0000034	11.4979156

96892_at	Psma1	0.0000345	8.0376671
96896_at	Actr2	0.0000374	9.6103759
96899_at	Ndufs3	0.0009032	9.1834234
96902_at	2900091E11Rik	0.0001911	6.9023786
96907_at	Cherp	0.0000010	8.8631890
96909_at	Ndufab1	0.0000708	9.7577621
96911_at	Gnb2	0.0001911	9.5538625
96913_at	Hadhb	0.0000031	7.8597119
96917_at	2410166I05Rik	0.0109870	6.8046543
96919_at	Atp6v0c	0.0006436	10.9943443
96924_at	9430077D24Rik	0.0002216	8.3077293
96925_at	2810024B22Rik	0.0000010	7.8320035
96930_at	Ehd1	0.0030388	7.3656866
96936_at	Copg1	0.0003425	8.1108970
96940_at	Tead2	0.0016809	9.6371630
96942_at	Eif3s6ip	0.0000009	11.0338203
96943_at	Gps1	0.0000031	9.9826675
96946_at	2810025O06Rik	0.0000018	7.0750020
96948_at	Qdpr	0.0000008	8.9860669
96949_at	2210410K23Rik	0.0000895	9.3459163
96951_at	Atp6v1d	0.0002966	7.5901814
96952_at	Psma6	0.0013419	10.6676874
96954_at	C76483	0.0000374	7.3228287
96956_at	Prdx5	0.0011775	8.1063149
96957_at	Cib1	0.0072874	6.7065911
96959_at	Ube2n	0.0000116	9.9582246
96962_at	Rpl6	0.0001217	12.2815789
96987_at	Mllt7	0.0077080	7.7118673
96995_at	Sms	0.0168074	2.9490178
97004_at	Olfr71	0.0077080	7.9500307
97055_s_at	Prdx1	0.0006436	11.9368018
97061_g_at	Ywhaq	0.0000044	11.4365120
97090_at	Tcf20	0.0013419	5.0557099
97104_g_at	0610038L10Rik	0.0002565	7.2447869
97107_at	1700007D05Rik	0.0034293	4.8524410
97114_at	Psap	0.0104128	8.6290122
97124_at	Fin15	0.0001044	5.8763655
97132_at	Krt2−8	0.0000075	8.2378236
97154_f_at	−−−	0.0000011	6.9648517
97161_at	Sfpq	0.0036397	5.1633260
97163_g_at	D030022P06Rik	0.0002216	7.3036865
97164_at	2610207P08Rik	0.0000058	7.9730307
97165_r_at	D2Ertd93e	0.0013858	5.2641872
97166_at	Elac2	0.0000374	7.1149292
97171_f_at		0.0025288	7.3046957
97181_f_at	Iap	0.0000075	10.8214490
97182_at	Ccne2	0.0001044	4.5777255
97184_at	BC023106	0.0096031	5.7961241
97186_s_at		0.0158536	4.7698650
97197_r_at		0.0001983	5.5998932
97199_at	Cpne1	0.0030388	7.6959773
97201_s_at	Ndufa5	0.0011025	9.2773100
97205_at	2700079K05Rik	0.0000021	9.1348848
97207_f_at	Lypla1	0.0001982	7.9286614
97208_at	Ash2l	0.0086076	7.0882891
97210_at	1700037H04Rik	0.0000655	7.8256086
97211_at	3230401N03Rik	0.0001128	8.8153506
97217_at	Ahcyl1	0.0000040	9.7115696
97220_at	Dscr2	0.0000012	8.8860804
97227_at	Gna12	0.0028592	5.8270461
97229_at	5730427N09Rik	0.0006892	8.7378917
97237_at	1810003N24Rik	0.0000090	9.3653076
97238_at	Tacc3	0.0000269	7.5575377
97240_g_at	D19Ertd721e	0.0000040	9.5104371
97241_at	D19Ertd721e	0.0000037	8.4712806
97249_at	D7Wsu180e	0.0006892	8.7254959
97250_at	Nola3	0.0000605	10.7822992
97251_at	Mrps10	0.0001774	6.4873568
97253_at	2810411G23Rik	0.0043493	5.9397849
97254_at	Rbm8	0.0000069	9.6652953
97255_at	Cugbp2	0.0090894	4.7587755
97257_at	Lactb2	0.0006006	5.1545289
97258_at	Lactb2	0.0005803	4.8272338
97259_at	Arpp19−pending	0.0030388	6.7596551
97261_at	Dnaja1	0.0000439	9.6597461
97262_at	Csnk1d	0.0146787	9.1878806
97263_s_at	Csnk1d	0.0000034	9.6403918
97264_r_at	Csnk1d	0.0046111	7.5633286
97269_f_at	Kcp2−pending	0.0019703	9.7548081
97271_at	1500034E06Rik	0.0001528	7.5490204
97272_at	Hnrpa1	0.0014016	8.3569694
97274_at	Psmd14	0.0000258	9.0081299
97277_at	1810015M01Rik	0.0090924	6.6363630
97279_at	6430402H10Rik	0.0000344	7.6143502
97284_at	Bcl2l13	0.0000516	8.3842555
97285_f_at	Ubxdc2	0.0105455	5.7189849
97288_at	Pdzk1	0.0125493	6.0373988

97293_at	Rbm10	0.0001774	7.9775996
97295_at	D4Ertd421e	0.0000019	8.8690627
97296_at	Mrpl44	0.0000374	7.2505449
97301_at	Rab14	0.0000138	9.0499928
97302_at	Ivns1abp	0.0000048	8.5306005
97304_at	Ubp1	0.0000058	8.5898718
97305_at	1110017C15Rik	0.0000008	8.8471248
97307_f_at	Ndufb5	0.0043501	10.7788738
97308_at	5730466P16Rik	0.0011025	7.7433199
97309_at	3110002K08Rik	0.0000075	9.2683266
97310_at	3110002K08Rik	0.0000075	8.0064916
97312_at	Cd164	0.0000034	9.4647867
97313_at	Gdi1	0.0125493	7.7806041
97315_at	2610028L19Rik	0.0001044	8.9865702
97317_at	Enpp2	0.0000053	5.7999864
97318_at	Hars2	0.0036411	6.5562176
97320_at	1600025H15Rik	0.0001774	7.4854075
97327_at	Fen1	0.0000021	8.5298843
97329_at	C77668	0.0001217	8.5153622
97330_at	Abcf1	0.0000069	9.5455482
97333_at	Dnchc1	0.0000210	9.1164342
97339_at	−−−	0.0013419	8.1103573
97340_at	Sart3	0.0000210	8.2853658
97343_at	D5Wsu46e	0.0096015	8.2851141
97345_at	Anp32e	0.0000967	7.9274630
97346_at	2610001J05Rik	0.0054898	4.7366734
97347_at	Ltbp4	0.0065145	6.2189189
97349_at	4930488L10Rik	0.0000057	6.7468758
97355_at	AW050020	0.0086076	5.8665078
97358_at	Lphn1	0.0000010	8.2769581
97363_at	Ptk2	0.0132266	7.5409758
97364_at	Asf1b	0.0002759	6.6683540
97367_at	Akap1	0.0000605	5.7128376
97368_at	Akap1	0.0077067	7.5108284
97369_g_at	Akap1	0.0081474	6.7475222
97370_at	4633402N23Rik	0.0002058	7.3439278
97375_at	Pkd1	0.0001313	5.5477341
97380_at	1700016A15Rik	0.0000063	8.6932876
97382_at	Tcp11	0.0162671	3.7774752
97383_at	Slc6a6	0.0007895	8.8334469
97390_at	Cdc25a	0.0001646	8.5115697
97392_at	2410015J15Rik	0.0000248	6.5854408
97393_at	Vrk1	0.0000828	7.0070750
97394_at	Smarca5	0.0000012	8.4608393
97395_at	D19Wsu55e	0.0000116	9.5533558
97397_at	D5Ertd33e	0.0000786	6.0882989
97401_at	1300006C06Rik	0.0000053	7.1785214
97403_at	2310050B20Rik	0.0002860	7.4216032
97404_at	1500034J01Rik	0.0000895	7.4598433
97405_at	Rps6ka1	0.0000031	7.7590396
97406_at	Cbfa2t2h	0.0001044	6.6257782
97407_at	Gdbr1−pending	0.0016043	7.2396801
97411_at	Ect2	0.0012572	8.2091571
97413_at	1600029D21Rik	0.0004877	5.3641678
97414_at	AI326906	0.0009656	8.5207094
97419_at	2310010I22Rik	0.0146787	7.3813134
97421_at	Smc2l1	0.0000075	8.7251355
97422_at	LOC381314	0.0002759	7.7857628
97423_at	1500035H01Rik	0.0106982	7.5533124
97428_at	Dom3z	0.0086076	7.9672412
97433_at	Mcm3ap	0.0000228	7.3645518
97434_at	2810405F18Rik	0.0077040	4.5972060
97436_at	Man2c1	0.0068924	8.3768951
97441_at	Acinus	0.0008446	8.6314845
97443_at	Mrpl52	0.0009032	9.6747768
97444_at	Ifi30	0.0000021	6.6252486
97445_at	Ppid	0.0000008	9.4972443
97446_at	Dhx30	0.0000069	8.5318117
97447_at	1010001H21Rik	0.0006890	7.1789583
97448_at	9030221M09Rik	0.0000008	8.4988198
97449_at	Aldh7a1	0.0081474	8.8429087
97450_s_at	Aldh7a1	0.0000034	9.3697443
97456_at	Facl5	0.0016595	6.3065845
97458_at	Gnb1	0.0011025	9.7559804
97459_at	Psma4	0.0000008	10.4705675
97462_at	3110006P09Rik	0.0000027	8.9845420
97463_g_at	3110006P09Rik	0.0000026	9.7831106
97464_at	3110006P09Rik	0.0000193	9.2198679
97468_at	Cks1	0.0000037	10.2163173
97471_at	Arcp−pending	0.0000126	7.9051174
97472_at	Slc25a17	0.0011025	8.0679816
97473_at	Tm4sf7	0.0001044	8.2485969
97477_at	Timm8b	0.0018496	8.2143868
97478_at	C030004C14Rik	0.0026894	7.6526284
97479_at	Ube2l3	0.0051821	9.6901970
97480_f_at	Dnajb3	0.0150585	4.1893088
97483_at	Rpl19	0.0012572	12.8830710

97484_at	2210402G22Rik	0.0000116	8.7735792
97486_at	U2af1	0.0000015	10.0898561
97487_at	Serpine2	0.0001364	6.8816497
97488_at	1200011O22Rik	0.0000075	8.9474605
97490_at	Bcl7b	0.0166853	4.9841993
97491_at	Hnrpr	0.0000028	8.4029646
97497_at	Notch1	0.0001527	7.5025277
97498_at	Fhl1	0.0068924	8.5092044
97500_g_at	Fhl1	0.0018496	7.7553066
97502_at	Dld	0.0000016	8.7604436
97504_at	Ccnd2	0.0000009	7.0469622
97505_at	Arl1	0.0000248	8.0417666
97506_at	Rnf2	0.0000708	7.3586916
97509_f_at	Fgfr1	0.0000708	9.3796613
97512_at	2010107E04Rik	0.0002758	9.9559942
97514_at	1810063B05Rik	0.0000021	6.8189648
97515_at	Hsd17b4	0.0000516	7.7797326
97516_at	G2an	0.0000021	8.7938920
97517_at	Rrp41−pending	0.0061541	7.6058354
97518_at	Fdft1	0.0036411	7.8117641
97519_at	Spp1	0.0162695	2.5600539
97521_at	Ass1	0.0132266	6.5593261
97527_at	Cks2	0.0000163	10.1432231
97528_at	Cnih	0.0028592	9.5750651
97530_at	Ube2i	0.0025288	8.8622274
97532_at	Ppp5c	0.0008437	7.0587165
97535_at	Ywhah	0.0003949	9.5219593
97538_at	Gus	0.0008164	6.7683181
97540_f_at	H2−D1	0.0000106	7.3166748
97541_f_at	H2−D1	0.0003110	2.7520869
97543_at	Sept2	0.0000016	8.1671119
97544_at	Ywhaz	0.0086090	10.6850836
97548_at	LOC328110	0.0044780	4.4213000
97549_at	Cfl2	0.0101375	5.8035847
97554_at	BC005624	0.0000082	6.6786150
97556_at	Anp32e	0.0139359	9.3867940
97559_at	Eef2	0.0000028	11.8448915
97560_at	Psap	0.0065133	4.3282425
97565_r_at	Tfdp1	0.0002859	5.4428451
97576_f_at	MGC68300	0.0122205	2.3976338
97593_f_at	Fliih	0.0010320	7.3221420
97647_at	Rps16	0.0000138	12.7717876
97666_r_at	C76213	0.0000293	5.3739701
97684_at	Prkcabp	0.0038649	5.7702500
97695_s_at	Rpl7	0.0000008	12.5476093
97696_r_at	Rpl7	0.0000828	13.0727123
97703_at	2810470K21Rik	0.0000967	6.6214445
97704_at	Ell2−pending	0.0061552	2.0105899
97705_at	5031401C21Rik	0.0000967	7.0534343
97721_at	Fgf15	0.0012986	6.2434916
97722_at	Ssr1	0.0000090	8.0316708
97723_at	Cbx2	0.0018496	6.8301934
97740_at	Dusp16	0.0000559	5.9388946
97743_at	BC049953	0.0163693	8.7503553
97750_at	Lamr1	0.0002966	11.1293372
97751_f_at	Gapd	0.0006008	9.2757280
97758_at	Prdx1	0.0025288	11.8213887
97778_at	Siat6	0.0032287	3.0685352
97797_at	2210409M21Rik	0.0005231	8.9146798
97798_at	4930504E06Rik	0.0025288	5.5570702
97800_at	Fastk	0.0001128	7.2966930
97803_at	Mpp1	0.0000150	7.3039904
97807_at	1110021H02Rik	0.0041010	7.1665801
97808_at	Sf3b1	0.0020982	9.4721201
97809_at	Bat8	0.0000018	9.4936054
97812_at	Ranbp9	0.0004709	6.1658897
97813_at	Rela	0.0000405	8.0806956
97816_at	2600011C06Rik	0.0000605	6.5700437
97817_at	Spec1−pending	0.0009656	7.8539944
97818_at	Snx4	0.0000405	8.7756823
97819_at	Gsto1	0.0000126	8.3305010
97820_at	Galk1	0.0048900	9.3182455
97822_at	Pak2	0.0012572	7.7221032
97823_g_at	Pak2	0.0001416	7.8457746
97824_at	D11Ertd175e	0.0011025	10.6156598
97829_at	Cdipt	0.0090939	7.5114156
97833_at	Pfkp	0.0000011	5.9427234
97834_g_at	Pfkp	0.0000032	5.7078013
97838_at	Rnu22	0.0000016	9.4996501
97839_at	Snx6	0.0000034	9.6799978
97841_at	1500016L11Rik	0.0001647	7.3842393
97843_at	Ncoa4	0.0000016	8.1154539
97846_at	Cdc5l	0.0000018	8.4101527
97847_at	Rbmx	0.0028592	7.1543938
97853_at	Psip2	0.0000605	7.8768329
97857_at	Zdhhc3	0.0019703	7.6170209
97859_at	LOC212111	0.0146787	6.9577072

97863_at	D8Ertd354e	0.0001417	5.9581900
97864_at	2510049I19Rik	0.0000063	6.0359752
97865_g_at	2510049I19Rik	0.0000010	8.5151908
97866_at	2510049I19Rik	0.0000012	8.7592855
97868_at	Dnaja3	0.0002565	8.2600028
97869_at	Etfdh	0.0009337	5.1003722
97870_s_at	Ero1l	0.0001128	8.3464116
97871_at		0.0000895	8.4961781
97874_at	1500032D16Rik	0.0001774	7.6967116
97875_at	Adrm1	0.0000075	9.2715586
97876_at	Vps29	0.0000605	8.1601813
97880_at	Dlst	0.0000086	8.0758076
97886_at	Spr	0.0058140	7.0021669
97887_at	Apoc2	0.0002385	10.5896819
97890_at	Sgk	0.0000018	8.0828768
97892_at	Brd8	0.0000457	7.2497688
97894_at	Vars2	0.0000008	9.3632260
97895_f_at	Hat1	0.0000194	9.7074067
97896_r_at	Hat1	0.0000082	8.1807035
97897_at	C78339	0.0031318	7.1544956
97901_at	Ubtf	0.0004548	8.7181905
97903_at	0610027F08Rik	0.0003425	8.7936075
97904_at	Actr3	0.0000040	10.1979904
97906_at	Siah2	0.0065145	5.6385590
97907_at	0910001B06Rik	0.0009032	10.1102103
97908_at	1110007A06Rik	0.0000058	8.8279103
97911_at	0910001L09Rik	0.0009032	7.6427047
97914_at	Hspa9a	0.0000012	10.4633217
97916_at	5730494N06Rik	0.0000016	8.1489742
97918_at	AA536743	0.0119030	6.7588179
97919_at	1110021E09Rik	0.0004238	7.8153818
97922_at	Ncb5or	0.0006436	8.8266132
97923_at	B230218H07	0.0000060	8.5665365
97925_at	Csnk1e	0.0043501	7.7046042
97934_at	Galnt1	0.0068924	7.7276025
97935_at	4121402D02Rik	0.0000051	7.0966330
97939_at	Txndc1	0.0000031	8.2716818
97941_at	Capn6	0.0139160	2.3571640
97951_s_at	Tsc2	0.0001527	8.1223895
97953_g_at	Tsc2	0.0000126	7.3019931
97956_g_at	Tyrp1	0.0024513	3.0062284
97957_at	Slc27a4	0.0034293	5.4862320
97958_at	3632413B07Rik	0.0000021	6.7967658
97960_at	Usp22	0.0000037	7.4324498
97966_at	MGC28864	0.0034293	7.0774191
97967_at	Plxnd1	0.0139359	4.0045994
97972_at	Rnf103	0.0000516	6.0184989
97974_at	Zfpm1	0.0000026	8.5507193
97975_at	Nfatc3	0.0001313	7.2401511
97977_at	Ntn1	0.0041010	7.1076543
97979_at	Ppp1r7	0.0000028	7.8182457
97984_i_at	Kns2	0.0005491	6.9607147
97985_f_at	Kns2	0.0008446	7.8283362
97988_at	Mrg1	0.0018951	5.4991365
97989_at	Ppp3cb	0.0002058	5.8567535
97991_at	Kras2	0.0000150	7.0622399
97992_at	BC024683	0.0112863	6.2413374
97994_at	AI465550	0.0004709	8.4346180
97995_at	Tcf7	0.0015272	6.5632218
97998_at	Drpla	0.0139359	5.8304208
98000_at	Ly64	0.0046129	5.5282225
98001_at	Arhgef1	0.0000016	6.3189841
98004_at	Pkia	0.0096015	3.5363610
98005_at	Pkia	0.0086076	3.2985379
98006_at	Pola2	0.0000075	8.1510524
98007_at	Rps6ka2	0.0028592	7.4798263
98013_at	BC033609	0.0000075	6.6125898
98020_at	Drg1	0.0000516	9.3671474
98021_at	Paf53−pending	0.0006890	6.8502600
98024_at	Nfyb	0.0000537	8.5196439
98028_at	Twist1	0.0032287	7.4128003
98031_at	Bok	0.0007893	6.9028696
98032_at	Zfp35	0.0013858	6.8967467
98033_at	1100001H23Rik	0.0039805	6.5907383
98034_at	H2−DMb1	0.0072900	0.5379903
98037_at	1110003H18Rik	0.0009599	7.9788430
98038_at	Hmgb3	0.0072900	11.0209509
98044_at	Dab2	0.0026074	7.2139751
98045_s_at	Dab2	0.0000009	9.6172107
98047_at	5730410I19Rik	0.0000058	8.4355519
98048_at	Nssr	0.0000248	7.5618274
98049_at	1300018I05Rik	0.0000476	6.7897981
98051_at	AI114950	0.0068924	6.7169300
98053_at	Ywhab	0.0016284	7.9182675
98057_at	1110031E24Rik	0.0000069	5.3154856
98059_s_at	Lmna	0.0005605	6.1789357
98060_at	Lmna	0.0013548	5.6761825

98061_at	1110001K21Rik	0.0000405	8.1845439
98064_at	Aamp	0.0000033	9.6821218
98066_r_at	Brd2	0.0029465	6.7836498
98069_s_at	2400003N08Rik	0.0000345	7.7578719
98070_at	2400003N08Rik	0.0000405	8.0532529
98071_f_at	Dck	0.0002058	8.7648162
98075_at	G431001I09Rik	0.0015268	8.7231413
98076_at	Erp29	0.0020982	8.8898160
98077_at	Snrpd3	0.0000178	8.6826173
98079_at	Car14	0.0026894	9.4608614
98081_at	Rpo1−3	0.0000026	7.7997321
98083_at	Copeb	0.0000058	6.9848861
98084_at	Arl2bp	0.0000008	9.9701611
98085_f_at	Rpo1−1 Rps28	0.0000019	12.8456957
98087_at	Tbk1	0.0003184	6.8610874
98090_at	Hrb2	0.0002966	6.4758778
98094_f_at	Amfr	0.0003949	7.5148551
98097_r_at	2610019N19Rik	0.0001217	6.0966374
98099_at	Nudt9	0.0007377	7.2654228
98101_at	Gtf2a2	0.0096031	8.2052740
98104_at	Atp6v0b	0.0000318	9.0348199
98106_at	Timm44	0.0000008	8.1656976
98107_at	Coro1c	0.0006892	9.3335667
98110_at	Mdm2	0.0000040	7.0045817
98111_at	Hsp105	0.0000012	10.3547722
98112_r_at	Lap3	0.0015268	4.2242401
98113_at	Psmb1	0.0077080	9.5656192
98114_at	Npc1	0.0000011	8.5304844
98119_at	Rpl30	0.0004548	12.8477004
98121_at	Fnta	0.0000019	8.8087928
98127_at	Capza2	0.0000044	8.3367981
98132_at	Cycs	0.0000766	7.1012075
98134_at	2610101N10Rik	0.0004547	7.1728300
98140_at	Cdh1	0.0008446	7.0930767
98143_at	Fut8	0.0005604	6.3220168
98146_at	Nup88	0.0000138	8.6503135
98147_at	Usp5	0.0026894	8.6881784
98149_s_at	1110033J19Rik	0.0000012	9.3398709
98150_at	Rab11b	0.0002778	8.7602309
98151_s_at	Catns	0.0000345	7.6392815
98152_at	Catns	0.0032280	9.0617161
98153_at	Cct3	0.0000010	10.2061680
98154_at	1300004C11Rik	0.0000031	7.9680929
98155_r_at	Pold2	0.0012572	9.1527177
98168_at	Rpl7a	0.0003188	12.4566108
98169_s_at	Fzd3	0.0053319	7.2007528
98245_at	Csnk2a2	0.0002565	7.2921146
98254_f_at		0.0048900	11.5066728
98278_at	Spry4	0.0115892	6.3019460
98285_at	Zfp146	0.0001276	3.5361524
98298_at	Dpysl2	0.0036404	6.5583118
98305_at	Foxm1	0.0000439	7.1266318
98306_g_at	Foxm1	0.0000228	8.4227795
98312_at	Rspondin−pending	0.0028592	4.0939445
98322_at	Slc22a5	0.0021644	4.3909646
98326_f_at	4921513E08Rik	0.0081432	1.3793152
98333_at	Rps18	0.0001128	13.0292557
98335_at	Recc1	0.0000048	8.3077740
98337_at	Nkx1−2	0.0025288	6.6886365
98342_at	Rpl10	0.0000248	12.8189066
98344_f_at	2410018L13Rik	0.0154561	7.7837364
98346_at	9530051K01Rik	0.0004548	6.8812959
98356_at	D16Wsu65e	0.0101358	4.9943078
98379_r_at	Cop1−pending	0.0050332	7.3267861
98390_at	Impdh1	0.0101342	7.3581310
98402_at	Macf1	0.0000559	8.9620802
98404_at	U2af2	0.0000655	11.5955260
98415_at	Crlf3	0.0051821	8.2389116
98418_at	Dvl1	0.0004877	7.7298779
98419_at	Meox1	0.0001171	5.1951009
98424_at	Ptpn13	0.0000009	8.1525464
98428_at	Spg4	0.0012572	6.0969898
98431_at	Dusp12	0.0001495	6.8573827
98433_at	Bid	0.0112828	6.3886593
98434_at	Arhgef7	0.0000044	7.5155526
98436_s_at	Casp3	0.0025288	8.3926637
98437_at	Casp3	0.0000138	7.8465493
98441_at	Fmr1	0.0000011	7.3866560
98446_s_at	Ephb4	0.0000008	10.6083232
98451_at	Dnajb10	0.0026291	4.4306441
98452_at	Flt1	0.0000895	5.2787948
98454_at	Palm	0.0079215	8.0002587
98455_at	Atf2	0.0000116	7.9350716
98459_at	Shmt1	0.0000048	8.3504043
98460_at	Fto	0.0000008	8.4257584
98461_at	1200014P03Rik	0.0008446	7.3290912
98463_at	Smarca4	0.0000063	10.3145414

98464_at	1110011C06Rik	0.0000439	5.8709647
98468_r_at	Brd1	0.0000604	5.5183692
98477_s_at	Ank3	0.0002215	5.7905409
98482_at	Pthr1	0.0002759	6.8065482
98485_at	Rhpn2	0.0000018	6.4101535
98489_at	Hurp−pending	0.0007893	7.1894746
98490_at	2610313E07Rik	0.0001217	6.2148066
98491_at	2610313E07Rik	0.0004239	6.9765677
98492_at	Cklfsf7	0.0001044	7.9180889
98493_at	3200002M19Rik	0.0000150	7.4327161
98496_at	Gys3	0.0016284	7.8823903
98497_at	Eps15−rs	0.0112845	4.4050674
98502_at	Dusp19	0.0058971	6.3463006
98503_at	Evi5	0.0000028	7.0263633
98504_at	Rock2	0.0004239	6.4626335
98505_i_at	Cpo	0.0001797	6.6661506
98506_r_at	Cpo	0.0014318	5.8462585
98509_at	BC002199	0.0000009	9.0315407
98511_at	Raly	0.0000269	7.9687890
98512_at	Banp	0.0058140	7.9086128
98514_at	Tfpi	0.0005426	6.3055143
98515_at	Gtf2e2	0.0000106	8.7084674
98516_at	BC056474	0.0000008	8.7076076
98521_at	Vamp3	0.0006436	7.8443095
98522_at	Psmd8	0.0000082	8.5412132
98525_f_at	edr	0.0000082	12.4413873
98532_at	Cdkap1	0.0000069	10.7134086
98535_at	Comt	0.0000516	6.9111293
98538_at	2610507B11Rik	0.0000126	7.6069304
98539_at	Cops2	0.0000016	6.2003956
98540_g_at	Cops2	0.0002385	7.6112452
98544_at	Guk1	0.0005231	8.4743539
98545_at	Bcap37	0.0000019	10.1566957
98550_at	Set	0.0011025	10.7598552
98552_at	Pmf1	0.0036404	7.7860302
98553_at	Slmap	0.0000048	7.3952276
98554_at	Ndr3	0.0061508	4.6996941
98557_f_at	Psmb4	0.0008446	10.8256485
98560_at	Cltb	0.0154538	5.3559333
98564_f_at	Rps26	0.0000374	12.5736656
98571_s_at	Naca	0.0000044	11.4564671
98572_at	Dnajb11	0.0014504	8.0812156
98573_r_at	Ranbp1	0.0000967	10.4738266
98574_at	Prpf8	0.0000008	10.2352666
98575_at	Fasn	0.0000031	9.1534173
98577_f_at	Erv4	0.0019082	4.3823711
98579_at	Egr1	0.0011772	5.4978488
98580_at	Ppm1a	0.0002473	7.5117350
98586_at	Nap1l1	0.0005607	8.7842588
98587_at	Nap1l1	0.0000439	10.3026811
98588_at	Fah	0.0011025	6.8630468
98593_at	Cmas	0.0000044	8.2103230
98594_at	1190002N15Rik	0.0000895	8.0074102
98599_at	Hdgfrp2	0.0004877	7.9135285
98602_at	Rangap1	0.0001128	9.4095152
98603_s_at	Rangap1	0.0000063	7.9213785
98604_at	Nnp1	0.0000098	8.8111739
98605_at	Wars	0.0000023	8.3995557
98606_s_at	Wars	0.0020982	9.1493165
98608_at	MGC18745	0.0001841	9.4532628
98610_at	Mrps28	0.0036411	7.6185168
98613_at	2700085E05Rik	0.0112863	7.5826877
98615_at	0610041E09Rik	0.0001217	8.5004986
98616_f_at	Myh7	0.0023770	4.1567050
98617_at	Wbp11	0.0000015	9.5393693
98619_at	Dtymk	0.0007895	7.9909943
98623_g_at	Igf2	0.0000011	11.4656597
98624_at	Rnpc1	0.0001044	8.0435519
98625_s_at	Adh5	0.0000026	10.4961452
98626_at	1810017G16Rik	0.0001417	7.8944365
98627_at	Igfbp2	0.0000011	10.1147392
98628_f_at	Hif1a	0.0000405	10.1943082
98629_f_at	Hif1a	0.0000344	10.2423812
98635_at	D11Moh35	0.0000293	8.7395099
98756_at	2810043O03Rik	0.0001647	4.6883293
98758_at	Alox15	0.0000008	6.2154233
98759_f_at	Tuba2	0.0000201	12.8983390
98761_i_at	Zfp97	0.0000040	8.0737234
98762_f_at	Zfp97	0.0000106	5.8667860
98767_at	Yy1	0.0016284	8.0136792
98803_at	Zfp354a	0.0125493	3.1202246
98804_at	Hesx1	0.0000026	5.2358695
98815_at	Evx1	0.0008732	6.5896669
98831_at	Foxj1	0.0125493	6.1408288
98839_at	Six2	0.0038649	6.3351779
98860_at	Sts	0.0014318	3.9793591
98865_at	Has2	0.0004238	6.2792651

98868_at	Bcl2	0.0099642	3.7636663
98874_at	Traf6	0.0119030	3.9347146
98875_at	D2Ertd391e	0.0036404	6.5156346
98880_at	D6Wsu116e	0.0000708	7.8989326
98882_s_at	Ndel1	0.0006154	8.1827788
98884_r_at	Ndel1	0.0000359	5.3872364
98890_at	1700012G19Rik	0.0125474	5.9741754
98893_at	Pb1−pending	0.0000828	6.7831704
98894_at	Pb1−pending	0.0001647	7.8674247
98896_at	5830457O10Rik	0.0000012	8.9671871
98905_at	Sept7	0.0009032	9.4730661
98906_at	Fbxo9	0.0061552	5.9617333
98908_at	4833416I09Rik	0.0036411	8.5139276
98909_at	Lias	0.0000031	7.7489508
98910_at	D030012E24Rik	0.0001128	8.4760420
98911_at	Jak1	0.0112845	7.0453446
98912_at	AI256361	0.0000559	7.6239033
98914_at	Asf1a	0.0106982	9.9186043
98915_at	1600023E10Rik	0.0001417	8.4689944
98916_at	Ppp2r2a	0.0128821	5.9305018
98920_g_at	Blp1−pending	0.0000111	7.0543729
98921_at	Blp1−pending	0.0001044	7.4075487
98922_at	Itm1	0.0001554	5.7093455
98923_at	Rnac−pending	0.0003188	7.8554107
98927_at	Rab6	0.0002058	7.8560445
98929_at	1110018B13Rik	0.0024513	8.4172570
98930_at	Cope	0.0000009	8.6752479
98931_at	2610016K11Rik	0.0012572	6.9480497
98933_at	Mark3	0.0000048	8.1428712
98937_at	Tbrg1	0.0000090	8.9892494
98943_at	Rpa2	0.0023770	6.5854153
98944_at	Sec23b	0.0000008	8.2687369
98945_at	Sh3glb1	0.0000124	4.8814037
98946_at	Wsb1−pending	0.0000708	7.9673251
98948_at	Ns−pending	0.0000018	9.0278667
98950_at	Rragc	0.0000269	8.1408848
98953_at	Gc20−pending	0.0000439	9.3119689
98958_at	Ey2−pending	0.0059813	5.2263156
98959_at	0610016J10Rik	0.0006892	7.7768529
98967_at	Fabp7	0.0004547	5.6934182
98968_at	Myo5a	0.0002216	4.5240137
98972_at	Usp8	0.0146765	7.4217076
98973_at	2610318G08Rik	0.0000708	8.8965724
98975_at	2410008G02Rik	0.0000009	7.7468397
98977_at	Tinf2	0.0083955	7.7343878
98981_s_at	Tcf12	0.0000405	8.7485878
98982_at	AW547477	0.0000439	10.1518257
98983_at	P4ha2	0.0023770	7.1484033
98984_f_at	Gpd2	0.0000031	7.8599138
98988_at	Mail−pending	0.0001044	4.7922066
98990_at	Bcar1	0.0028592	7.9334033
98991_at	Smarcad1	0.0000098	7.1512746
98993_at	Ppp2r5c	0.0000052	8.5824076
98995_at	E2f5	0.0011020	7.7622416
98996_at	Stk18	0.0000013	8.0326749
98999_at	Adsl	0.0010320	8.5889869
99001_at	Zfp292	0.0000292	7.4393887
99011_at	Galnt3	0.0025288	4.2531104
99014_at	Apbb1	0.0001044	7.0747515
99015_at	Pml	0.0000476	6.7823932
99018_at	2700025J07Rik	0.0000654	4.7655058
99019_at	Por	0.0046129	7.6470161
99023_at	Pafah1b2	0.0024513	7.0990591
99025_at	Ddx19	0.0000009	8.3789022
99027_at	Bcl2l	0.0025288	8.0149132
99028_at	Rnf8	0.0003425	7.7249515
99031_at	Ankrd17	0.0000605	6.6825002
99034_at	Irx3	0.0000082	9.3119616
99038_at	Adss2	0.0000011	9.1253754
99039_g_at	Adss2	0.0000015	9.5650294
99041_at	Taf1b	0.0020982	7.1216945
99044_at	Zfp358	0.0000263	7.1315574
99049_at	Casp2	0.0005231	7.4434804
99054_at	Llglh	0.0000048	9.8888164
99059_at	Elf3	0.0056487	6.3877720
99064_at	Usp4	0.0012165	8.2215956
99067_at	Gas6	0.0043532	6.4169402
99068_at	Mcpr	0.0000150	8.4340025
99070_at	Chuk	0.0000082	7.5899993
99073_at	Ccnf	0.0000013	9.1507372
99074_at	Vil	0.0000318	6.4054972
99076_at	Nr1d2	0.0042231	4.9814840
99077_at	Thra	0.0162695	4.6288618
99078_at	1110033C18Rik	0.0000034	8.0690771
99080_at	2810012H18Rik	0.0000037	7.8024562
99082_at	Fkbp10	0.0007377	7.2693562
99087_at	D10Ertd749e	0.0000011	9.0796991

99093_at	2210402A09Rik	0.0000193	12.6169069
99095_at	Max	0.0000011	7.6450959
99096_at	Ddx24	0.0000018	7.7117040
99098_at	Fdps	0.0021644	9.3486051
99099_at	Stat3	0.0154561	7.2625010
99100_at	Stat3	0.0030382	5.8608571
99101_at	Eif3s7	0.0000034	10.5589194
99102_at	Usp9x	0.0000405	5.6697609
99103_at	Irf3	0.0007891	6.3037950
99106_at	Cops6	0.0002759	9.7168272
99109_at	Ier2	0.0000008	8.6223757
99111_at	Skd3	0.0015268	6.6557498
99112_at	Slc25a10	0.0162695	7.3233057
99113_at	Cops3	0.0000126	8.8644697
99115_at	2610041P16Rik	0.0009032	10.0681585
99119_at	Cfl1	0.0000405	11.5571691
99120_f_at	Chd4	0.0038649	11.9471004
99121_at	Fxr1h	0.0000053	5.7356373
99123_s_at	Fxc1	0.0011025	7.7978755
99126_at	Xist	0.0000009	6.6476441
99127_at	Sca10	0.0000019	10.0253902
99129_at	Clast3−pending	0.0054898	7.7300417
99133_at	Slc3a2	0.0000019	9.8191361
99135_at	Cdc37	0.0011025	9.7712879
99138_at	Chc1	0.0000008	8.6433936
99139_at	1810012I05Rik	0.0132246	7.0832813
99146_at	Stx6	0.0048900	6.1156169
99147_at	−−−	0.0001647	8.7799781
99148_at	Fh1	0.0086090	9.2915661
99149_at	2310035M22Rik	0.0000178	9.2971765
99150_at	Ict1	0.0036411	7.3343936
99151_at	2610002K22Rik	0.0001841	8.8891139
99154_s_at	1810020D17Rik	0.0049208	5.8440026
99158_at	Sh3d3	0.0038649	5.7268934
99159_at	Ppif	0.0000439	7.3567681
99160_s_at	1110025J15Rik	0.0020972	7.0952988
99161_at	1110025J15Rik	0.0050332	7.4172350
99162_at	2410004J23Rik	0.0000010	8.0713138
99164_at	Mapbpip−pending	0.0083740	7.5584673
99167_at	1110039H05Rik	0.0001647	8.5568865
99168_at	Pigs	0.0101375	8.1764286
99169_at	Carm1−pending	0.0000015	9.7064445
99179_at	3010002G01Rik	0.0003187	7.4623174
99180_at	Gtpbp4	0.0000048	9.8204035
99182_at	2610511E03Rik	0.0112863	7.2448653
99183_at	Ppp3r1	0.0000269	8.1664052
99184_at	Csad	0.0043541	6.5916845
99186_at	Ccna2	0.0000374	9.3738820
99187_f_at	2010315L10Rik	0.0000012	7.1681499
99188_at	2010315L10Rik	0.0000150	8.5294412
99191_at	Cri1	0.0007895	8.6362771
99194_at	1200003F12Rik	0.0000116	7.0839019
99196_at		0.0011025	9.7966307
99198_at	Rpl32−ps	0.0117971	5.5154842
99269_g_at	Tdo2	0.0043501	4.5234904
99325_at	Wnt3	0.0016284	6.0872335
99335_at	Hk1	0.0006892	12.0372339
99336_at	Rps5	0.0001128	12.3037555
99338_at	Tp120a−pending	0.0000019	7.8628046
99340_at	Gnb2−rs1	0.0000048	12.6188563
99361_at	Wnt8a	0.0000185	6.7794710
99365_at	Coq3	0.0048900	6.9528474
99367_at	Mapre1	0.0031318	3.6354914
99374_at	Epo	0.0038633	3.7440943
99397_at	Ercc2	0.0032287	6.8146614
99405_at	−−−	0.0001470	4.5086202
99431_at	Soat2	0.0000098	7.0795223
99448_at	Tln	0.0012572	7.9292344
99457_at	Mki67	0.0000090	8.9814921
99458_i_at	Mark2	0.0000088	9.2158139
99459_f_at	Mark2	0.0000106	5.8800902
99462_at	Top2b	0.0000040	6.4913634
99464_at	Rxrip110	0.0003949	7.0807897
99467_at	Rasa1	0.0000163	7.3776981
99469_at	Pex6	0.0038649	7.5408661
99471_at	Stard7	0.0003188	7.8022222
99475_at	Socs2	0.0000034	7.6539311
99485_at	Dffa	0.0000053	6.6111555
99490_at	2410044K02Rik	0.0002216	7.0474848
99493_at	AW496496	0.0008446	6.2449807
99497_at	Arfip2	0.0015767	7.0639928
99499_at	Mettl1	0.0011392	6.6561956
99502_at	Zfp148	0.0000150	6.0389971
99512_at	6030411K04Rik	0.0000009	10.0072022
99521_at	Ak4	0.0001647	6.8795085
99528_at	Spin	0.0066452	6.1923296
99529_f_at	Rnf138	0.0000098	6.2305660

99532_at	Tob1	0.0011775	6.1668874
99535_at	Ccrn4l	0.0000044	7.6156056
99537_at	Ruvbl1	0.0000248	9.3211836
99541_at	Kif11	0.0000708	8.0426523
99561_f_at	Cldn7	0.0006436	7.6789491
99562_at	Man2b1	0.0001128	7.9744199
99563_at	Spin	0.0000018	8.7446108
99564_at	Np95	0.0023770	9.9222578
99566_at	Tpi	0.0000069	11.7684895
99574_at	Znrf2	0.0000021	7.9482742
99575_at	Mgst3	0.0000026	9.1474514
99577_at	Kitl	0.0002385	6.5664121
99578_at	Top2a	0.0001647	9.1730069
99581_at	Hint	0.0000008	10.9808594
99587_at	Rab7	0.0000058	7.4972085
99590_at	Rps17	0.0016595	11.9651195
99592_f_at	Rdh11	0.0000088	8.2729037
99594_at	Mrpl51	0.0000031	9.4133437
99597_at	Gnai2	0.0026894	8.4836080
99598_g_at	Gnai2	0.0003678	10.6531121
99602_at	Tieg1	0.0038649	7.2127851
99603_g_at	Tieg1	0.0005050	6.1844193
99604_at	1810015H18Rik	0.0000708	8.8454079
99607_at	Skp1a	0.0000178	9.4767824
99608_at	Prdx2	0.0000177	6.6067440
99610_at	Ss18	0.0000345	8.6885389
99617_at	LOC218811	0.0000027	8.2754616
99619_at	D7Ertd743e	0.0061552	8.0375384
99620_at	Sfpq	0.0000559	7.2535209
99621_s_at		0.0000018	9.5526734
99622_at	Klf4	0.0000011	5.6116597
99624_at	9530068E07Rik	0.0006434	7.7690985
99628_at	Sfrs5	0.0000126	9.5925234
99629_at	Ei24	0.0012572	8.3719923
99630_at	Mrpl54	0.0000269	6.8032735
99631_f_at	Cox6a1	0.0020982	10.6131283
99632_at	Mad2l1	0.0000178	9.3512980
99633_at	Ncdn−pending	0.0007895	7.3556916
99635_at	Ing4	0.0043493	7.0870214
99636_at	Pdip38−pending	0.0000150	7.7451593
99639_at	Uchrp	0.0000012	9.7026632
99640_at	Minpp1	0.0007238	8.3995614
99642_i_at	Cpe	0.0000040	7.2619924
99643_f_at	Cpe	0.0000009	7.5250725
99644_at	Zfp289	0.0061552	8.7814293
99645_at	4921506J03Rik	0.0000605	8.5289395
99650_at	Csnk1a1	0.0154561	10.0113414
99651_at	Ry1−pending	0.0002298	7.9453370
99652_at	Bat5	0.0051821	7.9551630
99656_at	D8Ertd812e	0.0000013	7.6316900
99660_f_at	Cox7c	0.0001313	12.5523904
99662_at	Pcnt2	0.0003949	8.8758289
99663_g_at	Pcnt2	0.0032287	9.4632000
99664_at	Pcnt2	0.0032287	4.0214012
99665_at	Satb1	0.0001774	6.9919120
99666_at	Cs	0.0000516	8.9812107
99669_at	Lgals1	0.0016284	8.1939942
99674_at	Mrpl43	0.0090939	7.9234579
99677_at	2610042O14Rik	0.0000967	6.4067611
99678_f_at	Atp5l	0.0083725	4.6875585
99701_f_at	Sprr2b	0.0017358	6.8438821
99778_at	Rpl12	0.0020982	8.7910773
99801_at	Nodal	0.0004545	4.9905810
99812_at	Capn3	0.0072561	2.9827108
99842_at	Col19a1	0.0162648	3.5771494
99851_at	Zfp292	0.0001911	8.3374663
99855_at	Map3k5	0.0001127	5.2620547
99863_at	AI643885	0.0004878	4.9665717
99866_at		0.0008730	6.1965340
99872_s_at	Ftl1	0.0002058	11.8117500
99878_at	Ddx10	0.0013419	5.2592685
99914_at	Hand2	0.0000263	4.5252827
99917_at	Ezh2	0.0081474	9.1575635
99920_at	Terf1	0.0000010	5.9765608
99924_at	Tubg1	0.0036411	7.9609189
99926_at	Pigr	0.0106982	6.2594760
99935_at	Tjp1	0.0026894	8.8968611
99938_at	Xrcc1	0.0125493	7.2854085
99940_at	2410004N11Rik	0.0004548	7.6100044
99947_at	Tial1	0.0000008	8.4277145
99949_at	AI854876	0.0000031	8.0404256
99950_at	Tbp	0.0001911	6.8143892
99953_at	Rgl2	0.0000063	7.4034532
99954_at	U3−55k−pending	0.0000766	8.7164569
99955_at	Numb	0.0006008	6.5324399
99959_at	Ak4	0.0000318	7.9000736
99960_at	Map2k4	0.0028592	6.8885338

99961_s_at	Cdc2l2	0.0000016	7.3617699
99962_at	Kif2a	0.0000009	7.1521339
99970_at	Ptpn21	0.0104128	5.3811247
99975_at	Prkrir	0.0009337	7.5510565
99978_s_at	Mapk14	0.0000082	8.9625150
99985_at	Txnrd1	0.0000163	9.6412471
99986_at	Gosr2	0.0030376	6.5098206
99988_at	4933427L07Rik	0.0054898	6.3678582
99990_at	Rbbp6	0.0000044	8.5304918
99993_at	Anpep	0.0106982	6.2430440
99999_at	Np220	0.0119030	7.5688775
AFFX-18SRNAMur/X00686_M_at	Rn18s	0.0161274	6.3938458
AFFX-BioB-3_at		0.0000300	7.7277521
AFFX-BioB-5_at		0.0002233	7.6256831
AFFX-BioB-M_at		0.0000244	8.7469066
AFFX-BioC-3_at		0.0007977	8.9364731
AFFX-BioC-5_at		0.0000008	9.2177822
AFFX-BioDn-3_at		0.0005942	11.5646118
AFFX-BioDn-5_at		0.0000014	9.4077688
AFFX-CreX-3_at		0.0000001	13.0820567
AFFX-CreX-5_at		0.0000001	12.5931857
AFFX-CreX-5_st		0.0126520	5.4989520
AFFX-GapdhMur/M32599_3_at	Gapd	0.0000017	13.1226835
AFFX-GapdhMur/M32599_5_at	MGC68323	0.0002495	13.2489485
AFFX-GapdhMur/M32599_M_at	Gapd	0.0000010	13.2943658
AFFX-MURINE_B2_at		0.0002612	13.6977768
AFFX-TransRecMur/X57349_3_at	Trfr	0.0000003	6.8936566
AFFX-b-ActinMur/M12481_3_at	Actb	0.0031740	13.0931770

Table 4: Probesets of the MG-U74 experiment significant at the 5%-level with Benjamini-Hochberg FDR control

3.3 MOE4302

Affy ID	GeneSymbol	BHcorrected Pval	Mean Expression
1415671_at	Atp6v0d1	0.0000430	8.7706029
1415672_at	C130038N16Rik	0.0004544	9.6137336
1415694_at	Wars	0.0000430	8.2516552
1415698_at	Golph2	0.0000430	9.6311869
1415705_at	9130011J15Rik	0.0001044	6.9504404
1415723_at	Eif5	0.0000855	10.0978599
1415724_a_at	Cdc42	0.0002762	9.4889053
1415728_at	Pabpn1	0.0003553	9.4970904
1415734_at	Rab7	0.0006135	9.8749388
1415748_a_at	Dctn5	0.0009273	8.2858679
1415752_at	BC031181	0.0013815	8.8813857
1415756_a_at	Snap25bp	0.0031282	7.3122303
1415757_at	AW049900	0.0028151	7.6506584
1415763_a_at	2510006D16Rik	0.0011672	9.1015224
1415764_at	5730454B08Rik	0.0004472	9.7943712
1415783_at	Vps35	0.0000430	10.3800085
1415784_at	Vps35	0.0001456	7.3230222
1415785_a_at	Cct8	0.0022734	10.9806865
1415789_a_at	BC002236	0.0001879	8.3409551
1415802_at	Slc16a1	0.0000495	11.4818274
1415807_s_at	Sfrs2	0.0018289	10.5572388
1415814_at	Atp6v1b2	0.0002147	9.6308554
1415816_at	Cct7	0.0018263	11.0443826
1415824_at	Scd2	0.0000978	8.1863368
1415829_at	Lbr	0.0005446	10.5113832
1415831_at	Psmd2	0.0003553	10.6481330
1415836_at	Pycs	0.0017270	7.8393730
1415840_at	Elovl5	0.0031330	9.7947190
1415849_s_at	Stmn1	0.0005131	11.5212620
1415853_at	Def8	0.0010416	7.2763971
1415855_at	Kitl	0.0000430	7.5630697
1415863_at	Eif4g2	0.0001118	11.0914664
1415869_a_at	Trim28	0.0000855	11.5598374
1415878_at	Rrm1	0.0000430	9.9404812
1415880_a_at	Lamp1	0.0000495	11.1959565
1415882_at	Ghitm	0.0013076	8.6058458
1415890_at	Papss1	0.0018289	8.2167350
1415897_a_at	Mgst1	0.0000913	6.7838579
1415903_at	Slc38a1	0.0001766	9.1226668
1415904_at	Lpl	0.0002591	7.4022072
1415906_at	Tmsb4x	0.0000430	10.3584035
1415910_s_at	2810413N20Rik	0.0000430	9.1382666
1415911_at	Impact	0.0000430	7.8941746
1415912_a_at	Rps13	0.0003137	12.0731064
1415917_at	Mthfd1	0.0004020	9.7588452
1415927_at	Actc1	0.0001275	6.3404873
1415928_a_at	Map1lc3	0.0001657	8.8171737
1415929_at	Map1lc3	0.0001654	8.4284147
1415930_a_at	Map1lc3	0.0000495	9.3462204
1415933_a_at	Cox5a	0.0010416	11.4197064
1415935_at	Smoc2	0.0022643	4.9556710
1415938_at	Spink3	0.0000978	11.4817096
1415942_at	Rpl10	0.0022750	12.5349205
1415945_at	Mcm5	0.0000430	10.8575897
1415947_at	Creg	0.0024877	6.5265246
1415948_at	Creg	0.0001884	5.7958624
1415949_at	Cpe	0.0000747	8.0923638
1415961_at	Itm2c	0.0000977	9.2063692
1415966_a_at	Ndufv1	0.0022020	8.9218393
1415974_at	Map2k2	0.0032998	8.6220100
1415977_at	1300017C10Rik	0.0000494	9.0387681
1415983_at	Lcp1	0.0001550	6.1887330
1415985_at	1810061H24Rik	0.0000430	10.1438953
1415990_at	Vdac2	0.0006517	10.8825948
1415998_at	Vdac1	0.0000495	10.2431865
1416005_at	Psmc1	0.0003777	9.3408800
1416006_at	Mdk	0.0001768	11.4005971
1416009_at	Tm4sf8−pending	0.0000430	10.1247392
1416014_at	Abce1	0.0000567	9.4677273
1416015_s_at	Abce1	0.0000430	10.3874619
1416021_a_at	Fabp5	0.0000430	11.4360608
1416022_at	Fabp5	0.0001456	11.2324846
1416023_at	Fabp3	0.0028115	6.3511409
1416026_a_at	Rpl12	0.0006523	12.5576851
1416030_a_at	Mcm7	0.0001456	11.0238456
1416031_s_at	Mcm7	0.0010416	10.4281240
1416037_a_at	Cct2	0.0004825	11.7293310
1416041_at	Sgk	0.0000495	8.4943614
1416049_at	Gldc	0.0002436	9.0826037
1416058_s_at	Atp5c1	0.0014645	10.7974948
1416059_at	Sec23b	0.0000650	9.3343402
1416065_a_at	Ankrd10	0.0000652	8.2453686

1416067_at	Ifrd1	0.0011672	8.2844238
1416068_at	Kars	0.0000430	10.1088234
1416069_at	Pfkp	0.0000652	7.4165642
1416071_at	Ddx18	0.0008454	9.5229675
1416081_at	Madh1	0.0002145	7.0690774
1416088_a_at	Rps15	0.0020408	12.0366362
1416094_at	Adam9	0.0006140	7.4663448
1416103_at	Ywhaz	0.0000529	7.7223577
1416113_at	Fkbp8	0.0000747	8.8912984
1416114_at	Sparcl1	0.0019281	4.5423169
1416122_at	Ccnd2	0.0010408	9.4699419
1416123_at	Ccnd2	0.0008742	5.1215764
1416131_s_at	C920006C10Rik	0.0002765	7.1634511
1416140_a_at	Dhx30	0.0001659	9.2734834
1416142_at	Rps6	0.0000430	8.1113490
1416143_at	Atp5j	0.0005783	10.7429897
1416148_at	Laptm4b	0.0022734	9.6786143
1416153_at	Srp54	0.0000978	9.9002742
1416167_at	Prdx4	0.0002767	10.7606420
1416175_a_at	Vdac3	0.0000430	10.3226416
1416177_at	Rbmxrt	0.0001277	10.4218933
1416180_a_at	Rdx	0.0005122	8.3818794
1416181_at	Mesdc2	0.0000855	10.2225782
1416183_a_at	Ldh2	0.0034789	9.5745361
1416187_s_at	Pnrc2	0.0011681	8.2085858
1416199_at	Kifc3	0.0034895	5.8236661
1416202_at	Bcap37	0.0003134	10.5108017
1416211_a_at	Ptn	0.0000650	6.4221924
1416226_at	Arpc1b	0.0012273	7.4493909
1416239_at	Ass1	0.0005778	6.9285847
1416265_at	Capn10	0.0034746	6.7993960
1416267_at	Scoc	0.0000429	7.4247149
1416280_at	Uble1b	0.0003336	10.5845363
1416288_at	Dnaja1	0.0018289	10.8414271
1416299_at	Shcbp1	0.0007343	9.1162537
1416300_a_at	Slc25a3	0.0011681	11.7893939
1416319_at	Adk	0.0011672	8.1784064
1416330_at	Cd81	0.0009273	9.2824437
1416340_a_at	Man2b1	0.0001118	7.9810719
1416341_at	Polr2c	0.0000430	9.3965827
1416349_at	Mrpl34	0.0000914	9.3807398
1416354_at	Rbmx	0.0000430	8.7602463
1416355_at	Rbmx	0.0001652	4.6762919
1416362_a_at	Fkbp4	0.0005131	10.5968483
1416365_at	Hspcb	0.0010408	12.0917202
1416368_at	Gsta4	0.0000568	8.8276346
1416378_at	Pnkp	0.0010186	6.3357292
1416382_at	Ctsc	0.0001657	9.9960262
1416384_a_at	Cope	0.0000854	9.0834482
1416394_at	Bag1	0.0001659	9.0528477
1416404_s_at	Rps16	0.0007343	12.5394144
1416417_a_at	Ndufb7	0.0000991	9.9562680
1416419_s_at	Gabarapl1	0.0032977	7.7190196
1416420_a_at	Rpl9	0.0028188	12.8216119
1416427_at	Ccni	0.0008253	10.9323325
1416428_at	AB041579	0.0000495	9.1925654
1416429_a_at	Cat	0.0022734	8.2727189
1416430_at	Cat	0.0007320	8.1065629
1416431_at	2310057H16Rik	0.0000430	8.1855262
1416442_at	Ier2	0.0007343	8.8856465
1416451_s_at	Tbn	0.0008246	6.9684385
1416479_a_at	1110021D01Rik	0.0000652	8.5017896
1416480_a_at	Hig1−pending	0.0000567	9.7070020
1416494_at	Ndufs5	0.0002767	8.6297208
1416497_at	Cai	0.0003137	8.5156895
1416510_at	Mrpl4	0.0004544	9.0494183
1416530_a_at	Pnp	0.0016362	9.3099865
1416532_at	AI481500	0.0001877	8.8821101
1416536_at	9430059D04Rik	0.0013835	8.4660469
1416545_at	Zdhhc7	0.0010408	7.6695706
1416551_at	Atp2a2	0.0001118	8.3962271
1416552_at	Dppa5	0.0000746	6.5522602
1416556_at	2700085A14Rik	0.0004020	9.6875841
1416567_s_at	Atp5e	0.0000652	9.1176418
1416572_at	Mmp14	0.0008446	6.0640783
1416589_at	Sparc	0.0002291	9.4841769
1416610_a_at	Clcn3	0.0000430	8.4920808
1416624_a_at	Uba52	0.0000430	12.9855474
1416628_at	0610006I08Rik	0.0000652	8.3435667
1416629_at	Slc1a7	0.0000430	7.2740055
1416645_a_at	Afp	0.0000430	10.7981011
1416646_at	Afp	0.0000430	9.8362844
1416654_at	Slc31a2	0.0009265	6.0507748
1416655_at	1500002I11Rik	0.0002434	7.1996873
1416658_at	Frzb	0.0002765	8.4539504
1416663_at	Ndufa9	0.0007337	9.6748455
1416671_a_at	Mcoln1	0.0034746	7.3034321

1416680_at	Ube3a	0.0002439	9.1021196
1416683_at	Plxnb2	0.0006523	8.1016236
1416693_at	Foxc2	0.0028188	7.9874129
1416696_at	Il25	0.0026692	8.0256916
1416708_a_at	D7Bwg0611e	0.0006507	6.4264557
1416712_at	Pep4	0.0013825	8.1176229
1416726_s_at	6720465F12Rik	0.0000430	10.6664024
1416727_a_at	Cyb5	0.0004544	9.3984761
1416740_at	Col5a1	0.0012346	6.8185350
1416742_at	Cfdp	0.0000430	10.1671164
1416749_at	Prss11	0.0008226	6.6702392
1416750_at	Oprs1	0.0000568	8.5307989
1416755_at	Dnajb1	0.0000430	9.1173945
1416756_at	Dnajb1	0.0000430	8.6756032
1416762_at	S100a10	0.0001456	8.5962001
1416767_a_at	1110003E01Rik	0.0005764	6.8585655
1416768_at	1110003E01Rik	0.0002145	6.4686691
1416792_at	Ppm1g	0.0009280	10.7966294
1416800_at	Trpm7	0.0022750	7.2944888
1416810_at	Mea1	0.0004267	8.3200865
1416812_at	Tia1	0.0026709	7.7095172
1416813_at	Tia1	0.0018276	7.8120487
1416829_at	Atp5b	0.0013835	11.9411097
1416832_at	4933419D20Rik	0.0022719	9.0192449
1416836_at	Lrp10	0.0009265	8.1776635
1416841_at	1110059E24Rik	0.0000854	7.9194987
1416857_at	Sdf2	0.0000648	8.4756590
1416858_a_at	Fkbp3	0.0002147	10.9465698
1416859_at	Fkbp3	0.0000429	10.5317865
1416860_s_at	Ing1	0.0001456	8.5806403
1416868_at	Cdkn2c	0.0005069	4.2233466
1416877_a_at	Mrpl51	0.0000568	10.0020003
1416899_at	Utf1	0.0032998	8.3672583
1416911_a_at	6330407G11Rik	0.0032998	6.8596004
1416961_at	Bub1b	0.0003777	9.0992384
1416962_at	Chc1	0.0005127	8.3622723
1416965_at	Pcsk1n	0.0000493	7.0653642
1416967_at	Sox2	0.0021535	8.7327228
1416970_a_at	Cox7a2	0.0000568	12.0638516
1416971_at	Cox7a2	0.0000495	10.8822807
1416978_at	Fcgrt	0.0012310	3.7605702
1416979_at	2510048O06Rik	0.0009273	10.2540055
1417001_a_at	D4Wsu53e	0.0006517	8.3318858
1417018_at	Efemp2	0.0000430	7.7051940
1417022_at	Slc7a3	0.0013085	9.0099294
1417024_at	Hars	0.0034703	8.5800969
1417038_at	Sept9	0.0001512	8.9740759
1417040_a_at	Bok	0.0006586	6.8570129
1417057_a_at	Ppid	0.0025335	10.3550171
1417059_at	Kcp2−pending	0.0002145	9.4602445
1417064_at	5830427H10Rik	0.0034768	8.4803035
1417065_at	Egr1	0.0000495	5.8777219
1417073_a_at	Qk	0.0026709	6.1239185
1417081_a_at	Syngr2	0.0001553	8.1583594
1417085_at	Akr1c6	0.0002141	1.5687213
1417087_at	Glg1	0.0000430	9.2647679
1417095_a_at	Hsp70−4	0.0006125	7.6554419
1417112_at	Arl2bp	0.0000568	9.1360334
1417117_at	Cstf1	0.0004472	6.4053192
1417124_at	Dstn	0.0003432	9.8177894
1417125_at	Ahcy	0.0003553	11.1598765
1417135_at	Srpk2	0.0001657	6.2101202
1417143_at	Edg2	0.0000742	7.2173054
1417146_at	2410018C20Rik	0.0011537	7.2560421
1417149_at	P4ha2	0.0013835	7.0596267
1417157_at	Actr10	0.0000430	8.2323232
1417182_at	Dnaja2	0.0000977	7.5496471
1417183_at	Dnaja2	0.0000430	9.0827773
1417195_at	D8Ertd594e	0.0009795	6.6261740
1417196_s_at	D8Ertd594e	0.0032977	6.6342081
1417197_at	D8Ertd594e	0.0003553	5.8399029
1417204_at	Kdelr2	0.0022750	10.2322438
1417210_at	Eif2s3y	0.0002765	6.9061802
1417216_at	Pim2	0.0000495	10.0302564
1417219_s_at	Tmsb10	0.0028170	11.4930112
1417225_at	Arl6ip5	0.0034746	7.2949857
1417233_at	2810014D17Rik	0.0008259	9.1524147
1417235_at	Ehd3	0.0032977	6.5684115
1417243_at	2310065K24Rik	0.0000854	8.7464046
1417245_at	E130016I23Rik	0.0000799	7.2010485
1417253_at	Frg1	0.0000608	8.6878533
1417259_a_at	Capzb	0.0020394	8.5272472
1417287_at	H13	0.0000747	8.1036904
1417288_at	Plekha2	0.0001317	7.8798032
1417289_at	Plekha2	0.0026385	6.5763279
1417293_at	Hs6st1	0.0001192	7.8938560
1417302_at	Rcor	0.0019295	8.8735501

1417304_at	Chrd	0.0003435	5.7653081
1417311_at	Crip2	0.0002767	8.5703094
1417316_at	Them2	0.0024953	7.7321736
1417339_a_at	Dnclc1	0.0000978	12.3204455
1417343_at	Fxyd6	0.0002145	7.1636199
1417357_at	Emd	0.0034789	8.4253739
1417359_at	Mfap2	0.0004017	7.6873354
1417368_s_at	Ndufa2	0.0009280	9.5866095
1417371_at	Peli1	0.0031291	8.1200355
1417373_a_at	Tuba4	0.0000650	9.1733677
1417374_at	Tuba4	0.0000978	7.9488401
1417376_a_at	Igsf4	0.0022734	8.4722882
1417377_at	Igsf4	0.0014624	8.4055708
1417378_at	Igsf4	0.0002439	9.1881078
1417385_at	Psa	0.0014624	8.5576582
1417386_at	Psa	0.0022734	8.3255367
1417387_at	3110004H13Rik	0.0014645	7.6337241
1417388_at	Bex2	0.0001116	9.7106843
1417394_at	Klf4	0.0000568	6.1265556
1417395_at	Klf4	0.0005127	5.8594139
1417401_at	Rai14	0.0014602	6.3179965
1417404_at	Elovl6	0.0006910	8.2523441
1417405_at	Stard3	0.0014645	7.6924408
1417409_at	Jun	0.0000742	6.7904422
1417417_a_at	Cox6a1	0.0011681	11.4257153
1417418_s_at	Cox6a1	0.0020394	10.3166298
1417456_at	Gnpat	0.0001888	9.0277543
1417460_at	Ifitm3l	0.0002439	10.2202908
1417465_at	Fnta	0.0000430	9.6880720
1417473_a_at	6330579B17Rik	0.0019308	5.3200017
1417475_at	Cgi152−pending	0.0000430	9.1444641
1417490_at	Ctsb	0.0002767	10.4612451
1417510_at	Vps4a	0.0000430	7.7549812
1417513_at	Evi5	0.0022750	8.5314151
1417517_at	Plagl2	0.0003137	7.1256140
1417519_at	Plagl2	0.0019755	6.4006259
1417524_at	Cnil	0.0011663	5.9493709
1417525_at	Hand1	0.0000430	8.9877751
1417538_at	Slc35a1	0.0003333	7.9506485
1417541_at	Hells	0.0020408	9.7475328
1417559_at	Sfxn1	0.0001659	7.9379913
1417560_at	Sfxn1	0.0016374	9.5718720
1417562_at	Eif4ebp1	0.0000568	9.6781869
1417569_at	D15Ertd412e	0.0016374	5.2339683
1417574_at	Cxcl12	0.0006523	8.0823244
1417581_at	Dhodh	0.0001117	7.1187591
1417582_s_at	Dhodh	0.0000429	7.5048882
1417583_a_at	Crlz1	0.0010416	9.0261497
1417588_at	Galnt3	0.0018238	6.0014873
1417595_at	Meox1	0.0028170	5.8134649
1417600_at	Slc15a2	0.0001274	6.4367807
1417604_at	Camk1	0.0000493	5.1148238
1417606_a_at	Calr	0.0000652	11.6538337
1417619_at	Plinp1−pending	0.0004020	7.8810257
1417622_at	Slc12a2	0.0001277	8.6543487
1417635_at	Spa17	0.0008246	5.0130623
1417648_s_at	Snx5	0.0000494	9.2784019
1417649_at	Cdkn1c	0.0001277	8.7153197
1417652_a_at	Tbca	0.0002439	10.7158355
1417666_at	6430706C13Rik	0.0000974	8.0491752
1417670_at	Timm44	0.0000606	7.7693644
1417681_at	Cpsf5	0.0000430	10.9466401
1417693_a_at	Gab1	0.0025335	7.5903527
1417694_at	Gab1	0.0008253	7.5426656
1417703_at	Pvrl2	0.0001275	8.8772228
1417705_at	AI850305	0.0013825	7.7959674
1417713_at	Eif2s2	0.0010408	10.8341728
1417715_a_at	Got2	0.0000430	11.0543774
1417716_at	Got2	0.0000430	9.2913585
1417719_at	Sap30	0.0000430	9.5641996
1417722_at	Pgls	0.0004020	9.0404297
1417734_at	Nakap95−pending	0.0002010	7.1058690
1417737_at	Mrps31	0.0001763	8.2525850
1417741_at	Pygl	0.0000912	7.0457454
1417745_at	Cpn1	0.0002584	5.9137624
1417748_x_at	Foxm1	0.0020366	6.8899040
1417753_at	Pkd2	0.0001454	8.0834279
1417761_at	Apoa4	0.0001454	7.3893880
1417766_at	Cyb5m−pending	0.0000430	8.3661415
1417767_at	Cyb5m−pending	0.0000650	7.3487598
1417821_at	G7e−pending	0.0000430	7.6771812
1417825_at	Es10	0.0000430	9.7064326
1417829_a_at	2310012G06Rik	0.0009265	7.8745690
1417836_at	3110050F08Rik	0.0013076	6.8518694
1417837_at	Tssc3	0.0001659	10.9571113
1417840_at	1500031L02Rik	0.0000647	7.6199677
1417841_at	Pxmp2	0.0010408	6.2793268

1417864_at	Pgk1	0.0001888	12.4909345
1417868_a_at	Ctsz	0.0006523	10.5890640
1417888_at	Trim13	0.0026385	6.1227012
1417890_at	AB041662	0.0003123	7.9728523
1417913_at	2810037C03Rik	0.0000855	8.7592746
1417920_at	Amn	0.0000430	9.6170115
1417921_at	2610029G23Rik	0.0007337	9.6040265
1417927_at	Ddx19	0.0000429	9.1207763
1417928_at	Ril−pending	0.0001362	7.4033142
1417937_at	Dact1	0.0004017	7.6713872
1417938_at	Rad51ap1	0.0000854	8.1773797
1417939_at	Rad51ap1	0.0010168	5.8788032
1417951_at	Eno3	0.0016339	7.2144556
1417959_at	1110003B01Rik	0.0000975	6.6429515
1417969_at	2310046N15Rik	0.0007469	6.9580806
1417988_at	Resp18	0.0022689	0.5709404
1418000_a_at	Itm2b	0.0001277	9.9418296
1418002_at	2010110M21Rik	0.0005127	9.5886794
1418006_at	5830416A07Rik	0.0000430	8.2738663
1418027_at	Exo1	0.0001655	7.4621163
1418030_at	Slco3a1	0.0034661	5.1339238
1418032_at	AI646725	0.0021506	7.6585905
1418042_a_at	Abcc5	0.0001657	8.4958269
1418084_at	Nrp	0.0001888	7.7134415
1418086_at	Ppp1r14a	0.0011527	5.3748136
1418091_at	Crtr1−pending	0.0029664	6.2370300
1418101_a_at	Rtn3	0.0001657	9.9667008
1418129_at	Dhcr24	0.0003132	5.9178937
1418135_at	Mllt2h	0.0001274	6.9215251
1418153_at	Lama1	0.0002427	7.1174416
1418154_at	N4bp1−pending	0.0002767	8.0085604
1418167_at	Tfap4	0.0005089	7.1954086
1418187_at	Ramp2	0.0002432	7.8620609
1418188_a_at	Ramp2	0.0002596	7.1919159
1418206_at	Sdf2l1	0.0002147	7.1784694
1418209_a_at	Pfn2	0.0000696	8.2625411
1418210_at	Pfn2	0.0001359	8.0274236
1418217_at	Nme7	0.0012346	8.1287345
1418223_at	Spc18−pending	0.0000747	10.3091227
1418235_at	Apg5l	0.0017282	6.0739413
1418237_s_at	Col18a1	0.0022750	9.3480726
1418258_s_at	6720463E02Rik	0.0000430	8.2531200
1418264_at	Solt	0.0004263	8.1194213
1418274_at	Nutf2	0.0000430	11.2270163
1418277_at	Rp9h	0.0028170	8.2327180
1418292_at	Asna1	0.0000494	9.7628195
1418295_s_at	Dgat1	0.0008246	7.6235479
1418319_at	1810047C23Rik	0.0006523	8.0132610
1418326_at	Slc7a5	0.0009818	8.5729441
1418329_at	2810003H13Rik	0.0013076	5.6668186
1418341_at	Rab4a	0.0001888	7.4206120
1418365_at	Ctsh	0.0003137	9.6231393
1418371_at	6720463E02Rik	0.0008749	8.0331743
1418377_a_at	Siva−pending	0.0005788	9.2069399
1418379_s_at	Gpr124	0.0005079	6.3773191
1418402_at	Adam19	0.0000977	7.5933514
1418430_at	Kif5b	0.0028151	8.9795138
1418436_at	Stx7	0.0001454	8.7311583
1418451_at	Gng2	0.0004540	6.7916190
1418462_at	Pmscl1	0.0001659	8.1844129
1418490_at	Sds−rs1−pending	0.0019308	4.2195651
1418496_at	Foxa1	0.0004267	6.0590502
1418506_a_at	Prdx2	0.0000430	11.3938995
1418510_s_at	Fbxo8	0.0007343	7.0820724
1418517_at	Irx3	0.0004472	9.5881130
1418524_at	Pcm1	0.0000651	8.7158213
1418526_at	Nssr	0.0000978	7.9945686
1418528_a_at	Dad1	0.0001888	10.1311070
1418532_at	Fzd2	0.0014645	9.8138856
1418533_s_at	Fzd2	0.0000977	9.5406932
1418534_at	Fzd2	0.0000568	8.4385800
1418553_at	D030053O22Rik	0.0025335	7.8977131
1418560_at	Pdha1	0.0004825	10.0522226
1418564_s_at	1200009K13Rik	0.0034789	10.9929441
1418569_at	2410043F08Rik	0.0000529	6.5848079
1418585_at	Ccnh	0.0003333	5.8167565
1418587_at	Traf3	0.0010400	6.0284431
1418589_a_at	Mlf1	0.0025286	5.7085190
1418615_at	Astn1	0.0008313	5.4674513
1418622_at	Rab2	0.0007783	9.5370832
1418634_at	Notch1	0.0026709	7.7049771
1418649_at	Egln3	0.0001456	7.3110052
1418662_at	2210012G02Rik	0.0019755	4.8969664
1418664_at	Mpdz	0.0007343	8.2983420
1418677_at	Actn3	0.0025269	5.7287226
1418684_at	2310012P17Rik	0.0033091	5.8472769
1418692_at	Mel	0.0001118	9.3140751

1418701_at	Comt	0.0000742	6.6054705
1418702_a_at	2810428I15Rik	0.0000565	8.5551373
1418707_at	Bag4	0.0003544	6.1217114
1418711_at	Pdgfa	0.0001275	7.5091829
1418719_at	2410004L22Rik	0.0003550	7.4356973
1418723_at	Edg7	0.0001971	3.5283696
1418726_a_at	Tnnt2	0.0001274	3.8399236
1418733_at	Twist1	0.0000853	6.9492580
1418756_at	Trh	0.0000567	8.3590868
1418760_at	Rdh11	0.0000976	6.8387296
1418761_at	Igf2bp1	0.0009818	9.1686608
1418763_at	D16Ertd502e	0.0006561	6.7297542
1418774_a_at	Atp7a	0.0008446	5.7464181
1418789_at	Sntg2	0.0034969	2.8351706
1418794_at	Cds2	0.0013085	7.4141015
1418795_at	Cds2	0.0005127	7.3202866
1418824_at	Arf6	0.0001192	5.3448502
1418827_at	3110010F15Rik	0.0029702	6.9730999
1418828_at	3110010F15Rik	0.0000744	8.0336995
1418837_at	2410027J01Rik	0.0001886	5.6582219
1418846_at	Ap4m1	0.0034746	5.7642850
1418856_a_at	Fanca	0.0001655	7.8907678
1418861_at	Piasg−pending	0.0007337	8.5071674
1418863_at	Gata4	0.0000975	7.0675650
1418873_at	Sfxn4	0.0022734	4.3944602
1418897_at	F2	0.0022673	6.2111208
1418910_at	Bmp7	0.0011672	6.1053924
1418925_at	Celsr1	0.0001768	6.7739346
1418932_at	Nfil3	0.0003773	6.0960532
1418938_at	Dio2	0.0031237	5.3404521
1418947_at	Nek3	0.0009273	7.2314085
1418961_at	1110005F07Rik	0.0033043	6.3906411
1418969_at	Skp2	0.0002008	8.9747805
1419022_a_at	Eno1	0.0028170	12.9262846
1419027_s_at	Gltp−pending	0.0000976	8.0104175
1419029_at	Ero1l	0.0000991	7.2009898
1419034_at	Csnk2a1	0.0002284	6.5423881
1419062_at	Epb4.1l3	0.0001118	8.9252744
1419076_a_at	Brca2	0.0004020	8.3669953
1419091_a_at	Anxa2	0.0000977	7.8160734
1419130_at	4933426M09Rik	0.0000494	7.4053074
1419140_at	Acvr2b	0.0016362	9.6837798
1419155_a_at	Sox4	0.0010416	8.1721557
1419156_at	Sox4	0.0007343	7.1468145
1419158_a_at	Harsl	0.0007337	6.4542165
1419163_s_at	Dnajc3	0.0021477	6.6989263
1419180_at	BC003321	0.0009810	7.2418066
1419204_at	Dll1	0.0002147	8.9361225
1419205_x_at	2610029K21Rik	0.0001884	6.8495152
1419252_at	Eps15	0.0003333	8.2319544
1419260_a_at	Snrpb	0.0002439	10.9841593
1419273_at	Rmp−pending	0.0004006	8.9215906
1419278_at	Usp31	0.0002434	8.2258114
1419279_at	Pip5k2a	0.0020394	7.2958595
1419287_at	1700006C06Rik	0.0020408	8.5629940
1419299_at	2010012O05Rik	0.0034697	5.5394777
1419300_at	Flt1	0.0008226	5.8146722
1419304_at	T	0.0000430	10.0931054
1419309_at	Gp38	0.0000641	8.4855574
1419350_at	Hook2−pending	0.0016339	6.6473419
1419367_at	Decr1	0.0005788	7.7500694
1419399_at	Mttp	0.0000430	8.3197492
1419406_a_at	Bcl11a	0.0031034	5.8070516
1419430_at	Cyp26a1	0.0001515	8.8542455
1419450_at	Ormdl3	0.0026692	6.5647393
1419452_at	Uchl5	0.0013825	9.0327579
1419462_s_at	Gtl3	0.0003137	8.7474106
1419470_at	Gnb4	0.0006507	7.1663045
1419485_at	Foxc1	0.0009810	5.3663184
1419486_at	Foxc1	0.0001275	7.1869045
1419516_at	D0HXS9928E	0.0003553	7.8099366
1419545_a_at	Atp6v1c1	0.0002012	8.6767832
1419549_at	Arg1	0.0000913	6.1402979
1419553_a_at	Rabggtb	0.0000747	9.2258833
1419565_a_at	Zfx	0.0034725	5.6536272
1419614_at	Pla2g13	0.0007770	7.6961729
1419635_at	4833420G17Rik	0.0013835	6.2253494
1419636_at	4833420G17Rik	0.0001203	5.9508650
1419637_s_at	4833420G17Rik	0.0013853	8.2991122
1419654_at	Tle3	0.0009251	7.3581132
1419657_a_at	C330005L02Rik	0.0002147	8.6643558
1419682_a_at	5630401H01Rik	0.0004010	6.8238843
1419688_at	Gpc6	0.0014645	6.8582956
1419693_at	Colec12	0.0005790	6.2421551
1419700_a_at	Prom1	0.0000430	9.2649601
1419717_at	Sema3e	0.0029570	5.1680147
1419722_at	Prss19	0.0000652	7.2579052

1419754_at	Myo5a	0.0004760	6.3550384
1419765_at	Cul2	0.0016327	5.6779235
1419803_s_at	2700094L05Rik	0.0014634	8.8492180
1419809_s_at	Cog4	0.0001118	7.8348943
1419814_s_at	S100a1	0.0005778	6.7183154
1419817_s_at	D1Ertd161e	0.0007337	6.5825679
1419889_at		0.0016281	3.4894084
1419933_at	1110004D19Rik	0.0004267	3.0994043
1419935_s_at	Csnk2a2	0.0002141	7.4908371
1419947_at	D4Ertd117e	0.0001117	7.8723847
1419976_s_at	Nfatc3	0.0000493	7.8457764
1420028_s_at	Mcm3	0.0006517	10.7286774
1420106_at	D9Mgi7	0.0019308	6.2800071
1420113_s_at	2410022L05Rik	0.0000565	8.3416416
1420136_a_at		0.0026623	4.5310141
1420142_s_at	Pa2g4	0.0020408	10.1568730
1420171_s_at	Myh9	0.0003211	10.1752540
1420296_at	T25545	0.0004536	6.8314063
1420337_at	Gbx2	0.0000430	7.6577301
1420338_at	Alox15	0.0000651	6.0428000
1420360_at	Dkk1	0.0029683	7.4290069
1420381_a_at	Rpl31	0.0001277	8.1677818
1420388_at	Prss12	0.0004020	7.8045394
1420397_a_at	Mint−pending	0.0000795	6.5929585
1420411_a_at	Pi4k2b−pending	0.0001454	5.9574770
1420414_at	Hoxa11	0.0023520	5.2085962
1420458_at	Tac4	0.0009584	5.1396730
1420484_a_at	Vtn	0.0005901	4.6874552
1420489_at	Mrps14	0.0002596	9.2450318
1420491_at	Eif2s1	0.0000746	5.6290043
1420498_a_at	Dab2	0.0001194	10.4738771
1420565_at	Hoxa1	0.0000495	8.8898929
1420570_x_at	Tcl1b3	0.0001117	9.9765316
1420598_x_at	Defcr−rs2	0.0000430	7.4517363
1420604_at	Hesx1	0.0005788	6.5234722
1420609_at	Axot	0.0009825	8.7794921
1420611_at	Prkacb	0.0011681	7.8878075
1420616_at	Ash2l	0.0000494	9.6535770
1420621_a_at	App	0.0001117	7.7419008
1420628_at	Pura	0.0003553	7.3830919
1420634_a_at	Madh2	0.0000651	7.0836151
1420637_at	Prps2	0.0004017	7.1799020
1420707_a_at	Traip	0.0006512	6.4920069
1420712_a_at	Hpn	0.0003773	6.1538920
1420719_at	Tex15	0.0005783	7.0155895
1420720_at	Nptx2	0.0000430	6.2777673
1420731_a_at	Csrp2	0.0001456	9.5968738
1420754_at	Ttf1	0.0002762	5.4147086
1420808_at	Ncoa4	0.0031330	8.9440741
1420814_at	Gdi3	0.0001659	10.3951233
1420815_at	Gdi3	0.0007343	11.0500801
1420820_at	2900073G15Rik	0.0003553	10.3801704
1420827_a_at	Ccng1	0.0000913	7.6756522
1420836_at	4933433D23Rik	0.0000641	7.1079146
1420845_at	Mrps2	0.0002357	7.1256908
1420847_a_at	Fgfr2	0.0022750	7.2846957
1420849_at	Crnkl1	0.0015476	6.2287591
1420877_at	Sept6	0.0015443	6.0090759
1420878_a_at	Ywhab	0.0001118	8.3852385
1420882_a_at	Nsd1	0.0000607	7.5834630
1420895_at	Tgfbr1	0.0032998	8.9819204
1420905_at	Il17r	0.0022198	5.9497656
1420914_at	Slc21a2	0.0007435	3.1480977
1420919_at	Sgk3	0.0007343	6.8638356
1420920_a_at	Arf1	0.0019308	10.1524176
1420933_a_at	Eya3	0.0022689	7.8592429
1420939_at	Hs6st2	0.0020878	2.7122470
1420965_a_at	Enc1	0.0033801	8.0442640
1420966_at	Slc25a15	0.0002418	5.6501801
1420967_at	Slc25a15	0.0004540	7.8452845
1420968_at	Nac1−pending	0.0000977	7.6349647
1420979_at	Pak1	0.0003126	7.8450142
1421007_at	Col4a6	0.0005441	3.8657552
1421011_at	retsdr2−pending	0.0028151	5.2577570
1421015_s_at	Pole3	0.0032977	7.3909671
1421024_at	Agpat1	0.0000430	7.2234816
1421025_at	Agpat1	0.0000494	7.6488772
1421033_a_at	Tcerg1	0.0010408	8.8014937
1421045_at	Mrc2	0.0013076	6.9179897
1421046_a_at	Pabpc4	0.0013037	10.3684269
1421052_a_at	Sms	0.0004271	9.8027436
1421063_s_at	Snurf	0.0018276	9.3487211
1421066_at	Jak2	0.0004528	6.3239365
1421113_at	Pepf−pending	0.0001886	6.8421009
1421115_a_at	Zdhhc16	0.0004528	7.8779711
1421116_a_at	Rtn4	0.0001886	8.7584963
1421139_a_at	Zfp386	0.0002760	5.8685585

1421143_at	Diap1	0.0031271	7.1912449
1421144_at	Rpgrip1	0.0001118	7.7113330
1421147_at	Terf2	0.0009584	7.0848667
1421148_a_at	Tial1	0.0000853	8.3044977
1421205_at	Atm	0.0000430	7.3711567
1421260_a_at	Srm	0.0001277	10.8132828
1421261_at	Lipg	0.0029627	2.5481858
1421267_a_at	Cited2	0.0002143	9.0037452
1421284_at	Pign	0.0021506	5.7780814
1421339_at	Extl3	0.0002141	5.2294287
1421344_a_at	Jub	0.0031330	9.5543012
1421385_a_at	Myo7a	0.0013085	6.0856202
1421390_at	Slc12a1	0.0003207	3.3401730
1421496_at	2410116I05Rik	0.0001886	5.9217740
1421498_a_at	2010204K13Rik	0.0010400	7.9186807
1421524_at	Cfc1	0.0001456	7.9749889
1421529_a_at	Txnrd1	0.0005783	10.0604935
1421546_a_at	Racgap1	0.0005446	7.7955321
1421633_a_at	Crtl1	0.0003333	5.0560724
1421657_a_at	Sox17	0.0002434	6.6687697
1421662_a_at	N33−pending	0.0022750	8.7381124
1421749_at	Lin28	0.0000429	9.4079277
1421798_at	Msgn1	0.0002765	6.5821019
1421813_a_at	Psap	0.0025302	7.8162637
1421823_a_at	Ppp2cb	0.0000747	9.9219650
1421830_at	Ak4	0.0025319	8.7249813
1421852_at	Kcnk5	0.0019228	5.9604018
1421861_at	Clstn1	0.0000494	8.0710595
1421872_at	Rab24	0.0000697	7.8563917
1421873_s_at	Rab24	0.0001454	7.3734607
1421892_at	Siat5	0.0017589	4.9424220
1421903_at	2810405O22Rik	0.0005783	7.4394179
1421904_at	Ncoa6ip	0.0013835	8.4967116
1421955_a_at	Nedd4	0.0000978	10.4889995
1421972_s_at	Hcfc1	0.0000429	7.7647009
1421995_at	Tcfap2a	0.0031291	5.9267680
1422033_a_at	Cntf	0.0023502	5.9298824
1422049_at	Nkx1−2	0.0002767	6.7303366
1422050_at	Nkx1−2	0.0000430	9.0577462
1422128_at	3100001N19Rik	0.0011681	8.3252190
1422134_at	Fosb	0.0000430	5.5348702
1422135_at	Zfp146	0.0004817	4.2332595
1422178_a_at	Rab17	0.0025269	6.0414999
1422185_a_at	Dia1	0.0031311	8.9098813
1422186_s_at	Dia1	0.0000568	8.9938519
1422228_at	Wnt8a	0.0001761	7.1326071
1422286_a_at	Tgif	0.0000651	8.4585504
1422318_at	Foxd4	0.0000430	5.4384442
1422418_s_at	Supt4h	0.0032998	9.3195925
1422426_at	Cmar	0.0013037	6.3487471
1422430_at	Fignl1	0.0004544	8.6826349
1422457_s_at	Smt3h1	0.0031291	10.2494003
1422475_a_at	Rps3a	0.0018289	12.7656411
1422476_at	Ifi30	0.0017234	8.2521647
1422480_at	Snx3	0.0014624	10.1813843
1422485_at	Madh4	0.0000568	9.3132730
1422486_a_at	Madh4	0.0001277	8.9465583
1422489_at	Gcs1	0.0000430	9.5032905
1422492_at	Cpo	0.0009601	6.9930181
1422502_at	Adprt1	0.0004267	10.5483088
1422512_a_at	Ogfr	0.0007783	7.7883787
1422520_at	Nef3	0.0014650	5.0314127
1422538_at	Extl2	0.0001886	6.9422729
1422557_s_at	Mt1	0.0001659	9.0604905
1422579_at	Hspe1	0.0001888	10.4534373
1422580_at	Myl4	0.0004013	4.9847846
1422587_at	C630002M10Rik	0.0021506	4.2683374
1422591_at	Tceb3	0.0001222	6.2863024
1422603_at	Rnase4	0.0000429	6.3637511
1422605_at	Ppp1r1a	0.0000747	7.0119185
1422610_s_at	Igf2bp3	0.0002284	9.7684285
1422614_s_at	Gcn5l1	0.0009273	7.5783249
1422627_a_at	Mkks	0.0002767	7.3689657
1422650_a_at	Riok3	0.0009601	7.8356987
1422664_at	Rab10	0.0023485	7.6989445
1422665_a_at	Pcmt1	0.0007343	6.9487530
1422675_at	Smarce1	0.0011672	8.9062572
1422692_at	Rpo2tc1	0.0001657	7.2591352
1422723_at	Stra6	0.0000650	6.1121390
1422731_at	Limd1	0.0006586	7.6064070
1422732_at	Pdip38−pending	0.0031291	7.9793827
1422733_at	Fjx1	0.0000430	6.7836224
1422740_at	Tnfrsf21	0.0035019	6.5219663
1422751_at	Tle1	0.0000429	7.1594704
1422753_a_at	1500004O14Rik	0.0013037	7.1351465
1422763_at	Rgs19ip1	0.0029683	7.7122010
1422767_at	Bysl	0.0034789	8.4097612

1422768_at	Nsap1−pending	0.0000652	8.5064704
1422771_at	Madh6	0.0008253	7.6180176
1422787_at	Fkbpl	0.0028151	6.1104836
1422789_at	Aldh1a2	0.0000430	8.5559662
1422792_at	Pafah1b2	0.0000607	8.7707686
1422793_at	Pafah1b2	0.0000493	7.5124132
1422798_at	Cntnap2	0.0034725	6.6241401
1422799_at	Bat2	0.0002439	9.6549292
1422807_at	Arf5	0.0001456	7.2206433
1422831_at	Fbn2	0.0006905	6.8822845
1422833_at	Foxa2	0.0013085	7.1690323
1422855_at	Cpsf3	0.0011015	8.6082980
1422870_at	Hoxc4	0.0005778	3.3346928
1422914_at	Sp5	0.0011023	7.7310920
1422927_at	2310016N21Rik	0.0033091	6.0112795
1422932_a_at	Vav1	0.0013774	2.9283279
1422950_at	Hes7	0.0020408	6.1376580
1422964_at	Rad23a	0.0015593	5.3279779
1422965_at	Agtrap	0.0013085	5.8478816
1422971_at	Gcn5l2	0.0018276	7.2501314
1422972_s_at	Gcn5l2	0.0011681	8.7941961
1423013_at	Foxf1a	0.0017589	7.2852916
1423037_at	Agtrl1	0.0000430	8.3786861
1423044_at	Prosc	0.0000429	8.1401904
1423049_a_at	Tpm1	0.0000746	8.7591496
1423060_at	Pa2g4	0.0002436	11.0562581
1423071_x_at	Itga6	0.0004271	7.3558357
1423078_a_at	Sc4mol	0.0014624	7.3112824
1423079_a_at	Tomm20−pending	0.0000495	12.0447312
1423080_at	Tomm20−pending	0.0000430	11.3047740
1423081_a_at	Tomm20−pending	0.0013085	8.5982206
1423086_at	Npc1	0.0003550	7.3269426
1423090_x_at	Sec61g	0.0016374	10.0882271
1423096_at	Capn7	0.0000568	7.0488562
1423097_s_at	Capn7	0.0003553	7.2843634
1423098_at	Capn7	0.0000493	7.2829189
1423100_at	Fos	0.0000568	7.1660859
1423111_at	Atp5a1	0.0005131	11.6131818
1423121_at	Ide	0.0008735	6.4728252
1423142_a_at	Gtpbp4	0.0001453	8.1035530
1423151_at	Dnajb11	0.0005783	8.9652940
1423161_s_at	Spred1	0.0001657	8.3889133
1423169_at	Taf7	0.0006512	6.2370287
1423170_at	Taf7	0.0000651	6.1080928
1423174_a_at	Pard6b	0.0001312	7.1087955
1423177_a_at	Ssh3bp1	0.0015476	8.1722300
1423187_at	Gabarapl2	0.0000494	8.3544711
1423188_a_at	6720456B07Rik	0.0003550	9.1459501
1423210_a_at	Nola3	0.0034789	11.3711277
1423211_at	Nola3	0.0000495	10.9084473
1423215_at	5730406I15Rik	0.0019770	10.1456380
1423216_a_at	2510049I19Rik	0.0000495	8.6909256
1423217_a_at	2510049I19Rik	0.0003550	8.2368148
1423219_a_at	Mrpl49	0.0000852	8.0370284
1423241_a_at	Tfdp1	0.0000430	10.1754574
1423245_at	Cops7a	0.0010408	8.5255776
1423247_at	Txndc4	0.0003777	8.4283631
1423250_a_at	Tgfb2	0.0011023	6.7037802
1423272_at	Polg	0.0000430	9.9300702
1423273_at	Polg	0.0004271	7.7642904
1423278_at	Ptprk	0.0006130	7.3176101
1423285_at	Coch	0.0007337	6.6719026
1423289_a_at	1810029B16Rik	0.0003137	7.7696542
1423290_at	Cab140	0.0025335	8.2034022
1423294_at	Mest	0.0000430	9.5349532
1423297_at	Add3	0.0013076	7.3078260
1423300_at	Zdhhc6	0.0025286	7.5933708
1423311_s_at	Tpbg	0.0025335	6.9223079
1423332_at	Sdcbp	0.0008239	9.4624732
1423355_at	Snap29	0.0018238	4.9867927
1423362_at	Sort1	0.0005089	7.9338449
1423377_at	Igfbpl1	0.0001888	4.1887882
1423388_at	Ap1g1	0.0001362	7.8331565
1423389_at	Madh7	0.0000430	6.7418383
1423390_at	Siah1a	0.0000568	7.7517342
1423391_at	Git2	0.0009818	7.1072116
1423393_at	Clic4	0.0000430	10.1319637
1423395_at	Tsnax	0.0011681	8.0167341
1423399_a_at	Yaf2	0.0006517	8.2299109
1423408_a_at	2500003M10Rik	0.0003547	9.8562726
1423429_at	Pem	0.0000430	9.4531946
1423430_at	Mybbp1a	0.0000494	9.9465725
1423433_at	Ssa2	0.0008749	6.3843434
1423468_at	Tsap6−pending	0.0010842	3.0230326
1423480_at	1500002M01Rik	0.0021506	7.2923470
1423484_at	Bicc1	0.0010408	7.1286070
1423488_at	Mmd	0.0013825	7.1369840

1423492_at	Mrpl45	0.0027975	8.5987687
1423501_at	Max	0.0016362	5.8513236
1423502_at	Brd2	0.0022734	8.2666621
1423506_a_at	Nnat	0.0000495	9.0929319
1423517_at	Cct6a	0.0001456	12.0998856
1423520_at	Lmnb1	0.0003137	8.2904730
1423521_at	Lmnb1	0.0010416	9.3237933
1423552_at	Leprotl1	0.0007332	7.4855340
1423601_s_at	Tcof1	0.0005127	7.9297004
1423608_at	Itm2a	0.0005131	8.2610792
1423611_at	Akp2	0.0010369	7.5218066
1423617_at	2610019N19Rik	0.0000747	8.2264017
1423624_at	Fancl	0.0001453	8.1159796
1423641_s_at	Cnot7	0.0000430	9.3920041
1423643_at	Ddx39	0.0000495	11.2183383
1423644_at	Aco1	0.0017589	7.4523929
1423648_at	P5−pending	0.0003137	9.9817084
1423649_at	2010300G19Rik	0.0014645	6.3175806
1423653_at	Atp1a1	0.0002147	9.6408809
1423659_a_at	BC017607	0.0021535	6.9606580
1423664_at	Qdpr	0.0000429	8.4473204
1423669_at	Col1a1	0.0001117	6.0235502
1423674_at	Usp1	0.0014645	8.4128928
1423675_at	Usp1	0.0002765	8.3251885
1423684_at	Hnrpk	0.0005796	10.8926485
1423685_at	Aars	0.0001655	9.2686202
1423689_a_at	Ags3−pending	0.0012328	6.6810777
1423691_x_at	Krt2−8	0.0005783	9.9821570
1423700_at	Rfc3	0.0005127	8.2676745
1423702_at	H1f0	0.0001456	9.2092021
1423703_at	Ppan	0.0013085	9.0620089
1423707_at	B230114J08Rik	0.0014634	6.3907685
1423716_s_at	0610008F14Rik	0.0014645	9.7726474
1423721_at	Tpm1	0.0000494	8.9791948
1423725_at	Pls3	0.0004017	9.4734270
1423728_at	Eif3s6ip	0.0029683	10.9732882
1423747_a_at	D530020C15Rik	0.0000430	7.8745229
1423748_at	D530020C15Rik	0.0002436	7.8888695
1423754_at	Fgls−pending	0.0031311	7.7538734
1423756_s_at	Igfbp4	0.0000855	10.2238597
1423757_x_at	Igfbp4	0.0000493	9.3367528
1423759_a_at	1190006A08Rik	0.0003134	9.1104359
1423763_x_at	Rps28	0.0004020	12.6092689
1423774_a_at	Prc1	0.0031311	7.7769249
1423781_at	Appbp1	0.0000430	9.8083542
1423785_at	Egln1	0.0000495	8.0854938
1423787_at	4832420O05Rik	0.0001456	9.5226866
1423801_a_at	Aprt	0.0000430	10.3432229
1423804_a_at	Idi1	0.0009265	7.0540155
1423805_at	Dab2	0.0001118	8.3732534
1423810_at	Pme1−pending	0.0013076	8.3781646
1423816_at	1110012O05Rik	0.0000430	9.2795082
1423817_s_at	2010315L10Rik	0.0001450	8.4606478
1423818_a_at	Arl6ip1	0.0000430	10.3790706
1423819_s_at	Arl6ip1	0.0000430	10.3073861
1423824_at	5031439A09Rik	0.0003336	8.7072972
1423825_at	5031439A09Rik	0.0003550	7.9932222
1423835_at	B830002A16Rik	0.0002143	6.5192865
1423838_s_at	2400003C14Rik	0.0000652	9.1679114
1423847_at	2810406C15Rik	0.0000430	9.4213907
1423848_at	Mphosph6	0.0000430	8.5386740
1423851_a_at	9430059P22Rik	0.0002944	6.5150461
1423852_at	9430059P22Rik	0.0000430	7.1760556
1423873_at	2810025O06Rik	0.0000430	8.0788669
1423882_at	BC027246	0.0000978	9.3475638
1423883_at	Facl2	0.0005127	6.9583617
1423884_at	Tex292	0.0000430	9.7797081
1423890_x_at	Atp1b1	0.0018276	8.7239888
1423901_at	Gtl6	0.0021535	7.5452073
1423903_at	D7Ertd458e	0.0017282	5.7010924
1423907_a_at	Ndufs8	0.0016362	9.3455744
1423916_s_at	Mlf2	0.0023979	9.9082333
1423924_s_at	D14Ertd226e	0.0012355	7.8796903
1423925_at	Dhx16	0.0004540	7.9872923
1423933_a_at	1600029D21Rik	0.0018250	4.6983314
1423939_a_at	Yif1	0.0013835	8.6299640
1423945_a_at	Pkig	0.0000430	7.5009435
1423946_at	Pdlim2	0.0015443	5.7985375
1423951_at	Blp2−pending	0.0004017	7.6287697
1423957_at	2700083B06Rik	0.0027975	8.6560548
1423960_at	Grcc3f	0.0022719	8.3229890
1423965_at	Mic2l1	0.0029521	5.8733965
1423971_at	2410044K02Rik	0.0018225	6.8025593
1423978_at	Sbk−pending	0.0000495	7.6018162
1423992_at	BC031407	0.0000430	10.1940223
1424003_at	4930444A02Rik	0.0005778	5.9236855
1424009_at	Ingaprp−pending	0.0003550	4.0154541

1424018_at	Hint	0.0007343	10.9806380
1424028_at	5830457O10Rik	0.0000430	9.1801959
1424029_at	2610102M01Rik	0.0022673	6.2623138
1424039_at	1810045K07Rik	0.0000567	9.1145017
1424045_at	5730437N04Rik	0.0008735	7.5034198
1424047_at	2500002K03Rik	0.0007337	8.0937639
1424048_a_at	1500005G05Rik	0.0000568	8.1048373
1424050_s_at	Fgfr1	0.0003137	9.7890743
1424053_a_at	D8Ertd325e	0.0000799	9.9196176
1424059_at	BC024816	0.0009273	8.1628346
1424065_at	Edem−pending	0.0034789	7.5081812
1424085_at	Ndufa4	0.0000747	10.5750955
1424091_at	AW108241	0.0002944	7.2898873
1424099_at	2310016C16Rik	0.0001118	7.2714974
1424101_at	Hnrpl	0.0013085	9.8483438
1424109_a_at	Glo1	0.0001454	9.9735373
1424110_a_at	Nme1	0.0010408	10.5289068
1424112_at	Igf2r	0.0000977	8.5119783
1424115_at	Ppp5c	0.0014645	8.5160231
1424117_at	BC056474	0.0003134	8.0411075
1424133_at	6530411B15Rik	0.0004540	8.1386638
1424134_at	4930470D19Rik	0.0000652	8.2961271
1424135_at	4930470D19Rik	0.0002767	7.5780006
1424143_a_at	Ris2	0.0000430	10.5215870
1424144_at	Ris2	0.0000430	9.6011362
1424151_at	Jtv1−pending	0.0003137	10.2499039
1424153_s_at	Sall4	0.0000978	8.5614027
1424159_at	1300010M03Rik	0.0000977	6.9006556
1424167_a_at	Pmm1	0.0000430	7.5125443
1424197_s_at	2810451D06Rik	0.0020352	6.5262401
1424198_at	Dlg5	0.0013066	8.0359181
1424200_s_at	Sec13l−pending	0.0013076	9.1729304
1424205_at	Smarca5	0.0000495	9.2103264
1424206_at	Smarca5	0.0000978	10.7452539
1424207_at	Smarca5	0.0000652	8.8444998
1424210_at	Keo4−pending	0.0001274	7.4530470
1424211_at	5730438N18Rik	0.0019755	7.4189972
1424214_at	9130213B05Rik	0.0023995	3.5484980
1424220_a_at	Porcn	0.0018263	6.5851025
1424235_at	Ormdl2	0.0003550	8.8819322
1424252_at	Hnrpdl	0.0009280	9.5379832
1424254_at	Mil2−pending	0.0000430	10.3838700
1424265_at	Npl	0.0004017	8.1593339
1424269_a_at	Myln	0.0023995	10.0279866
1424285_s_at	Arl6ip4	0.0011023	8.7238605
1424290_at	BC010311	0.0016350	6.7658758
1424291_at	2410008G02Rik	0.0000430	9.4489895
1424296_at	Gclc	0.0000495	8.1620549
1424299_at	2010001O09Rik	0.0004013	7.1008643
1424300_at	Gemin6	0.0006512	7.9838797
1424312_at	2810031L11Rik	0.0019175	5.8275078
1424313_a_at	Ndufs7	0.0004540	8.8815507
1424327_at	3200002M19Rik	0.0009265	7.4957162
1424344_s_at	Eif1a	0.0029683	8.5553237
1424348_at	BC025641	0.0031330	9.2532113
1424351_at	1600023A02Rik	0.0000430	8.1843744
1424355_a_at	Sin3b	0.0002762	8.5027520
1424365_at	1810037I17Rik	0.0000430	9.2752939
1424381_at	Sf4	0.0009825	8.5565843
1424382_at	D7Ertd671e	0.0022198	7.6281273
1424417_at	2210415M20Rik	0.0002425	6.2045209
1424424_at	Slc39a1	0.0004540	8.2620924
1424435_a_at	Gart	0.0016374	9.5005490
1424441_at	Slc27a4	0.0014645	7.1780069
1424450_at	Gprc5c	0.0022750	7.0218893
1424456_at	Pvrl2	0.0001115	7.9426525
1424483_at	4022402H07Rik	0.0013829	8.2072280
1424526_a_at	2610025M23Rik	0.0000745	6.1061162
1424530_at	Sec14l2	0.0004267	7.4431268
1424559_at	AW060207	0.0006916	6.7755788
1424561_at	Ece2	0.0019308	2.2346204
1424562_a_at	Slc25a4	0.0000430	10.3941214
1424573_at	3110020O18Rik	0.0013085	6.5583658
1424598_at	Ddx6	0.0005788	8.1537021
1424607_a_at	Xdh	0.0000430	10.0870567
1424609_a_at	Xdh	0.0000430	10.1565012
1424610_at	G430055L02Rik	0.0009280	7.6002705
1424674_at	Ermelin−pending	0.0008749	7.8213496
1424684_at	Rab5c	0.0010416	7.2253293
1424696_at	4933412D19Rik	0.0001768	7.7577041
1424712_at	Elys−pending	0.0010416	8.4599822
1424720_at	Mgat4b	0.0025335	7.8902025
1424726_at	BC014685	0.0014613	7.8378310
1424732_s_at	3110005G23Rik	0.0016374	7.3931096
1424736_at	Eef2	0.0000978	12.4466289
1424738_at	4932432K03Rik	0.0004764	6.7768193
1424743_at	2610003J06Rik	0.0013018	6.6957792

1424752_x_at	LOC235907	0.0003414	6.9910902
1424753_at	Nudt14	0.0013076	7.0949585
1424768_at	Cald1	0.0001115	7.8274023
1424769_s_at	Cald1	0.0002145	8.2557772
1424770_at	Cald1	0.0000651	7.4027172
1424778_at	D10Ucla1	0.0000651	7.3059327
1424779_at	D10Ucla1	0.0018263	6.3411679
1424803_at	BC020002	0.0008253	6.9704042
1424807_at	Lama4	0.0011628	3.8145599
1424808_at	Lama4	0.0012337	1.4473149
1424820_a_at	Ndfip1	0.0001274	8.4593881
1424821_at	Ndfip1	0.0000652	8.5269761
1424827_a_at	Csnk1a1	0.0006523	10.5409232
1424843_a_at	Gas5	0.0007343	8.8906644
1424852_at	5430401D19Rik	0.0031330	5.8338707
1424863_a_at	Hipk2	0.0003333	7.4275838
1424877_a_at	Alad	0.0013829	7.9110471
1424886_at	Ptprd	0.0031251	5.5715895
1424887_at	BC012312	0.0005094	7.4340750
1424893_at	Ndel1	0.0004267	5.4581176
1424903_at	Smcy	0.0010369	5.5167554
1424907_a_at	0610012A19Rik	0.0000565	9.0116760
1424946_a_at	1300002C08Rik	0.0003773	7.1583343
1424951_at	1300006M19Rik	0.0016350	6.9424976
1424955_at	BC024400	0.0007314	8.7346460
1425010_at	Zfp119	0.0004755	3.6435653
1425016_at	Ephb2	0.0025286	6.5923345
1425022_at	Usp3	0.0005788	7.7489734
1425023_at	Usp3	0.0000746	6.7372112
1425028_a_at	Tpm2	0.0002354	6.5784952
1425048_a_at	Hmgb1	0.0000430	11.6235857
1425074_at	Wrn	0.0009795	6.0286674
1425075_at	C430014D17Rik	0.0002767	7.6462155
1425097_a_at	Zfp106	0.0018276	6.3431011
1425098_at	Zfp106	0.0006135	4.3397589
1425129_a_at	Taldo1	0.0004020	8.9798811
1425134_a_at	2010319C14Rik	0.0001277	8.8009186
1425140_at	Lactb2	0.0004821	7.6192169
1425157_x_at	1300010A20Rik	0.0004252	5.4453485
1425179_at	Shmt1	0.0031251	8.5665503
1425189_a_at	Mrpl15	0.0001277	9.3549539
1425204_s_at	2810457M08Rik	0.0000430	8.4946071
1425228_a_at	Dguok	0.0025302	7.4322320
1425229_a_at	Tcf7l2	0.0007776	4.4978687
1425246_at	0610008F07Rik	0.0028043	5.6263828
1425266_a_at	Rap1gds1	0.0004017	8.0999037
1425270_at	Kif1b	0.0029664	6.5058203
1425332_at	Zfp106	0.0002145	9.5537636
1425336_x_at	H2−K	0.0000978	7.2583990
1425349_a_at	Myef2	0.0000650	6.6389448
1425362_at	A630095P14Rik	0.0031251	5.2132543
1425364_a_at	Slc3a2	0.0000651	9.9980642
1425440_x_at	1010001P06Rik	0.0029570	3.1711802
1425458_a_at	Grb10	0.0000430	11.0325448
1425466_at	4930538C18Rik	0.0007343	8.4706543
1425482_s_at	AI035571	0.0007332	6.2997163
1425497_a_at	Prpf4b	0.0008439	8.1616051
1425521_at	Paip1−pending	0.0011023	5.4375644
1425536_at	Stx3	0.0000798	9.1729220
1425545_x_at	H2−K	0.0019295	6.7844324
1425554_a_at	Cdc16	0.0000430	9.5582314
1425567_a_at	Anxa5	0.0003547	6.1881540
1425568_a_at	1600019D15Rik	0.0001888	8.5272711
1425581_s_at	Galnt7	0.0001359	7.3550815
1425610_s_at	Galnt2	0.0004013	7.2370824
1425640_at	Mllt2h	0.0001117	3.8005279
1425652_s_at	Rbpms	0.0001659	8.1771750
1425665_a_at	Srp54	0.0001362	8.7958458
1425716_s_at	Bak1	0.0008246	5.0712160
1425718_a_at	Ivns1abp	0.0000652	10.3650295
1425764_a_at	Bcat2	0.0001657	6.8588519
1425767_a_at	Six4	0.0003333	7.1604393
1425784_a_at	Olfm1	0.0022750	6.9374494
1425858_at	Ubc−rs2	0.0017270	7.1048087
1425908_at		0.0006517	5.3737731
1425911_a_at	Fgfr1	0.0000568	9.6094989
1425978_at	Srfcp−pending	0.0028151	4.6495197
1426001_at	Eomes	0.0000430	6.9490110
1426010_a_at	Epb4.1l3	0.0002942	8.0836135
1426024_a_at	Dbn1	0.0005446	9.5264319
1426030_a_at	Apeh	0.0013076	8.2653425
1426083_a_at	Btg1	0.0000987	8.3274023
1426088_at	protein kinase C, zeta	0.0014645	12.1394133
1426089_a_at	BC003331	0.0000650	5.7329293
1426100_a_at	Tk2	0.0017295	5.6963218
1426114_at	Hnrpab	0.0000855	9.8555703
1426118_a_at	Tomm40	0.0001659	9.5968688

1426162_a_at	Rpl7	0.0009825	12.3463973
1426165_a_at	Casp3	0.0008462	7.6711941
1426170_a_at	Cd8b	0.0005064	1.9071022
1426177_a_at	Nkx1−2	0.0031330	5.3134118
1426187_a_at	Hs1bp1	0.0025319	9.5913401
1426195_a_at	Cst3	0.0033091	9.1921016
1426207_at	Ikbkb	0.0000745	7.7844435
1426214_at	D1Wsu40e	0.0000651	4.6185062
1426255_at	Nefl	0.0004263	6.0837369
1426258_at	Sorl1	0.0005788	7.5456614
1426264_at	Dlat	0.0013774	7.7478527
1426266_s_at	2010001H09Rik	0.0001621	7.5711253
1426279_at	5830415L20Rik	0.0031330	8.3684286
1426294_at	Crtl1	0.0003132	4.7993207
1426304_x_at	B4galt7	0.0022198	5.2202237
1426340_at	Slc1a3	0.0004817	4.9055598
1426342_at	Simp−pending	0.0002147	10.9817683
1426348_at	Col4a1	0.0000978	8.5783128
1426352_s_at	Tial1	0.0003333	3.6562222
1426354_at	Bap1	0.0029586	8.7716402
1426359_at	5730454B08Rik	0.0003134	7.7378101
1426371_at	3732409C05Rik	0.0026675	8.2005628
1426378_at	2310046H11Rik	0.0009280	10.6927881
1426381_at	LOC226169	0.0008259	9.1856579
1426387_x_at	9030221M09Rik	0.0000746	7.4872777
1426392_a_at	Actr3	0.0000495	9.8281764
1426395_s_at	2700079K05Rik	0.0034703	7.3422143
1426406_at	2410195B05Rik	0.0011681	8.6372863
1426416_a_at	2310034L04Rik	0.0009810	7.6317642
1426422_at	BC016188	0.0001453	6.5210434
1426426_at	2600016B03Rik	0.0000430	9.6056753
1426471_at	Zfp52	0.0005105	4.4395258
1426473_at	5330419I01Rik	0.0004825	9.6863240
1426480_at	Sbds	0.0015476	8.1036256
1426512_at	Olfm3	0.0019228	0.7741974
1426515_a_at	Dyt1	0.0014624	6.7321702
1426521_at	BC006874	0.0000920	6.4832928
1426523_a_at	Sb52−pending	0.0026365	5.7035428
1426529_a_at	Tagln2	0.0012337	8.4895663
1426533_at	Nol5a	0.0007343	10.3363376
1426539_at	Usp11	0.0031330	6.8869329
1426554_a_at	Pgam1	0.0034768	12.0004572
1426557_at	Mesp1	0.0000978	8.0834010
1426558_x_at	3100002L24Rik	0.0001454	7.8875548
1426559_at	Sbno1	0.0018276	8.8058055
1426572_at	Me2	0.0003550	8.3482543
1426581_at	2810004N20Rik	0.0006474	7.6017418
1426599_a_at	Slc2a1	0.0000495	11.4759008
1426600_at	Slc2a1	0.0000430	11.6498363
1426607_at	3110070M22Rik	0.0002282	5.1860210
1426612_at	Tipin−pending	0.0000430	9.6365976
1426613_a_at	Snrpb2	0.0005446	9.3371784
1426646_at	9130011J15Rik	0.0007337	7.9705188
1426649_at	Tmeff1	0.0003540	8.0723679
1426652_at	Mcm3	0.0025335	9.1347303
1426658_x_at	Phgdh	0.0002147	10.0824953
1426661_at	Rpl27a	0.0031330	12.5711878
1426670_at	Agrn	0.0008253	8.9497476
1426674_at	Eif3s9	0.0011681	10.6041558
1426680_at	1110019I12Rik	0.0022719	7.8022057
1426707_at	Tubgcp3	0.0000565	7.3776106
1426710_at	Calm3	0.0000430	9.3404609
1426725_s_at	Ets1	0.0001117	5.6781007
1426726_at	D17Ertd808e	0.0011663	7.7159328
1426727_s_at	D17Ertd808e	0.0016350	7.8169720
1426736_at	Gspt1	0.0001277	9.7540578
1426738_at	Dgkz	0.0008246	6.8140481
1426752_at	D530048A03Rik	0.0007955	8.4910439
1426756_at	Galnt2	0.0004013	8.1728583
1426758_s_at	Gtl2	0.0000697	9.9349571
1426762_s_at	1810043O07Rik	0.0018276	9.8658353
1426764_at	Oaz2	0.0020408	8.2550560
1426778_at	Dag1	0.0002286	8.8337327
1426783_at	Gcn5l2	0.0016374	8.8750413
1426786_s_at	Dhx38	0.0003547	6.8822926
1426788_a_at	Ssrp1	0.0001657	11.0986383
1426789_s_at	Ssrp1	0.0034768	10.5642950
1426790_at	Ssrp1	0.0003553	10.4755098
1426799_at	D330025I23Rik	0.0002010	8.3550563
1426813_at	2610020N02Rik	0.0016362	8.7834695
1426817_at	Mki67	0.0003547	10.0594904
1426821_at	Cog8	0.0020380	7.0154754
1426824_at	Psme4	0.0031271	8.8436093
1426828_at	2410004N05Rik	0.0002145	6.7925792
1426846_at	G630055P03Rik	0.0000567	7.9010881
1426850_a_at	Map2k6	0.0006517	7.4970904
1426854_a_at	Set	0.0009280	12.2934029

1426864_a_at	Ncam1	0.0033091	6.1037421
1426873_s_at	Jup	0.0021535	7.3290690
1426874_at	BC022641	0.0000430	8.9247668
1426880_at	9430077C05Rik	0.0020352	7.3134702
1426886_at	Cln5	0.0001409	6.5988164
1426890_a_at	1190005L06Rik	0.0013835	8.0117483
1426904_s_at	1200006L06Rik	0.0000858	8.7747243
1426905_a_at	1200006L06Rik	0.0022734	9.4677538
1426908_at	Galnt7	0.0001456	7.9288304
1426910_at	Pawr	0.0020927	7.5396908
1426915_at	Dapk1	0.0032998	8.5254011
1426918_at	Itgb1	0.0000430	9.0171878
1426926_at	Plcg2	0.0000746	7.9229292
1426931_s_at	D19Bwg1357e	0.0007776	9.1931614
1426936_at	LOC215866	0.0000430	8.6745747
1426946_at	Kpnb3	0.0014645	11.0645333
1426948_at	Tpr	0.0010416	9.4335948
1426949_s_at	Tpr	0.0007343	9.7635123
1426953_at	Hmgb2l1	0.0000430	8.0239283
1426958_at	3010033P07Rik	0.0031328	7.6590643
1426977_at	4930502N04Rik	0.0002286	8.5950185
1426978_at	Klhl2	0.0000954	7.1627414
1426981_at	Pace4	0.0002942	6.1063393
1426990_at	Cubn	0.0000495	10.3300726
1427020_at	C130058N24Rik	0.0001192	5.1618120
1427021_s_at	Eif3s5	0.0000430	11.7426205
1427036_a_at	Eif4g1	0.0013835	10.9420055
1427044_a_at	Amph	0.0018276	5.7255652
1427047_at	LOC227699	0.0013085	8.5404829
1427048_at	E130215L21Rik	0.0001886	9.0734184
1427049_s_at	E130215L21Rik	0.0000495	9.4952467
1427058_at	Eif4a1	0.0000430	12.1914279
1427072_at	Stard8	0.0001454	8.7144008
1427095_at	E030027H19Rik	0.0022628	6.8963860
1427105_at	2610510J17Rik	0.0001886	9.0086865
1427115_at	Myh3	0.0016362	4.6291016
1427116_at	BC010250	0.0026692	7.9921789
1427117_at	Mtmr3	0.0026692	6.8227090
1427126_at	Hspa1b	0.0012986	4.8629861
1427127_x_at	Hspa1b	0.0015476	5.5892344
1427153_at	Bckdhb	0.0011663	8.3797802
1427195_at		0.0015465	7.1779107
1427207_s_at	2310036I02Rik	0.0035019	7.8536965
1427229_at	Hmgcr	0.0015454	7.1970404
1427230_at	B930041F14Rik	0.0017560	4.4710470
1427233_at	Sdccag33	0.0000429	5.7578737
1427248_at	Whsc2h	0.0000494	8.8387068
1427262_at	Xist	0.0000430	7.7682110
1427263_at	Xist	0.0000854	5.3068684
1427269_at	2610019N13Rik	0.0022704	7.8197255
1427271_at	6030404E16Rik	0.0005441	5.8199260
1427275_at	Smc4l1	0.0000430	10.4143582
1427277_at	Six1	0.0013829	4.1603498
1427292_at	Igl−V1	0.0014674	0.5865412
1427311_at	Falz	0.0025252	5.3759296
1427343_at	4930526B11Rik	0.0003550	5.0708161
1427347_s_at	Tubb2	0.0001657	5.8391882
1427364_a_at	Odc	0.0004544	8.7890464
1427375_at	3110023L08Rik	0.0019281	6.9652077
1427379_at		0.0006140	5.6807504
1427382_a_at	Suv39h1	0.0031311	8.1391471
1427408_a_at	9330151F09Rik	0.0002942	7.5200556
1427413_a_at	Cugbp1	0.0013076	8.8934520
1427442_a_at	App	0.0000430	8.9399856
1427469_at	9630002H22Rik	0.0035019	7.4826247
1427476_a_at	Trim32	0.0029683	8.3039008
1427518_at	LOC234358	0.0013825	6.4364897
1427523_at	Six3	0.0000977	6.0355541
1427580_a_at	Rian	0.0000978	7.0601266
1427605_at	Hoxb3	0.0022734	4.7968126
1427646_a_at	Lbcl1	0.0013066	6.4215627
1427672_a_at	Utx	0.0019308	7.7157355
1427797_s_at		0.0005127	5.3976559
1427820_at		0.0001456	10.2567004
1427898_at	1200013I08Rik	0.0032998	7.2273751
1427901_at	1110037D14Rik	0.0014634	8.6567952
1427902_at	5033413A03Rik	0.0000568	9.0031174
1427914_a_at	2610301I15Rik	0.0016362	10.0727720
1427916_at	St7l	0.0001191	6.5500785
1427917_s_at	Ssbp3	0.0031330	9.0285861
1427921_s_at	1110046L09Rik	0.0009280	8.0578605
1427939_s_at	Mycbp	0.0007469	7.6978939
1427962_at	BC027663	0.0001657	7.1785739
1427964_at	Cklfsf8	0.0000798	7.3963621
1427978_at	4732418C07Rik	0.0021506	6.5191539
1427994_at	Pigr3	0.0008454	5.4128865
1428001_at	Tshr	0.0018225	0.7398886

1428004_at	3300001G02Rik	0.0003435	8.8594316
1428011_a_at	Erbb2ip	0.0001192	7.0916216
1428029_a_at	H2av−pending	0.0004544	10.3366006
1428050_a_at	0610007H07Rik	0.0020352	8.1760803
1428055_at		0.0007776	7.1391417
1428068_at	1110030L07Rik	0.0013815	9.4304934
1428075_at	Ndufb4	0.0005788	9.4620313
1428096_at	Ipo11	0.0014645	8.2875951
1428099_a_at	Sfrs1	0.0000495	11.6835234
1428111_at	Slc38a4	0.0000430	8.6367786
1428112_at	Armet	0.0022734	10.2580376
1428116_a_at	Tctex1	0.0000495	11.3305008
1428117_x_at	Tctex1	0.0020408	5.3600898
1428121_at	2610528K11Rik	0.0011681	8.4468072
1428126_a_at	4921506J03Rik	0.0005778	6.8590019
1428128_at	4921506J03Rik	0.0013085	10.0715759
1428136_at	2210415K03Rik	0.0000978	8.5719750
1428137_at	2610313E07Rik	0.0028151	6.9989544
1428152_a_at	2510019J09Rik	0.0000495	12.5883260
1428154_s_at	2310022A04Rik	0.0007764	6.4577153
1428168_at	1110007A10Rik	0.0008735	8.5873587
1428172_at	LOC328110	0.0029543	7.1482282
1428174_x_at	Khsrp	0.0005778	8.3320366
1428180_at	2810422J05Rik	0.0004540	7.7983257
1428201_at	1110002H14Rik	0.0000430	9.5966681
1428224_at	Hnrpdl	0.0001277	7.5322972
1428231_at	4733401N12Rik	0.0012355	9.1174234
1428244_at	3110040D16Rik	0.0034768	9.8142836
1428251_at	4931400A14Rik	0.0003336	8.6692938
1428252_at	1190006E07Rik	0.0014592	7.5374237
1428257_s_at	Dncl2a	0.0022750	8.7987100
1428264_at	Prp8bp−pending	0.0002145	9.2348679
1428273_at	1110065L07Rik	0.0003547	6.8359223
1428275_at	1110065L07Rik	0.0004544	7.2923803
1428281_at	2610009I02Rik	0.0028115	6.9697043
1428286_at	2900097C17Rik	0.0008749	10.5495370
1428301_at	2610042L04Rik	0.0000430	9.3537025
1428310_at	D3Wsu161e	0.0001888	8.5651354
1428316_a_at	4833415N24Rik	0.0011672	8.7235739
1428319_at	1110003B01Rik	0.0032957	7.1009597
1428333_at	6530401D17Rik	0.0034768	8.5070153
1428339_at	5730530J16Rik	0.0002012	7.5810275
1428350_at	2310061F22Rik	0.0031311	7.5684762
1428354_at	1110054H05Rik	0.0025319	7.7509983
1428377_at	6330404E16Rik	0.0006496	3.2548712
1428381_a_at	2700038C09Rik	0.0012346	8.8324628
1428386_at	Facl3	0.0004825	6.2086606
1428401_at	2810406K24Rik	0.0004020	9.2353037
1428405_at	Hcfc1r1	0.0011023	7.3898460
1428417_at	3110050N22Rik	0.0000741	6.5508088
1428418_s_at	3110050N22Rik	0.0000430	6.8052819
1428422_at	2210404D11Rik	0.0008253	8.1585522
1428433_at	1110014O20Rik	0.0023979	7.8220423
1428441_at	1500009M05Rik	0.0005127	7.7145792
1428451_at	2900010D03Rik	0.0031330	9.0181750
1428452_at	2810025M15Rik	0.0001553	8.4978915
1428464_at	Ndufa3	0.0001657	9.4867588
1428467_at	1190002A23Rik	0.0009825	8.1299416
1428478_at	2700029E10Rik	0.0020380	7.0303706
1428494_a_at	Polr2i	0.0002624	9.4458790
1428498_at	2610206B13Rik	0.0005783	7.8919731
1428506_at	Atic	0.0001657	10.5585521
1428510_at	Lphn1	0.0003553	8.2358225
1428513_at	1810009B06Rik	0.0008239	6.8533082
1428515_at	2410012H22Rik	0.0006167	6.6617605
1428518_at	1700029A22Rik	0.0014613	7.6986287
1428524_at	2310051N18Rik	0.0025286	6.8475977
1428529_at	2810026P18Rik	0.0003137	9.5165240
1428542_at	2010003J03Rik	0.0002944	8.3579114
1428583_at	9530056D24Rik	0.0001886	7.8830326
1428589_at	2810443J12Rik	0.0019308	8.5840953
1428605_at	1810023B24Rik	0.0000429	8.3558086
1428608_at	1500001M02Rik	0.0006517	9.9296590
1428615_at	P2y5	0.0000652	8.2598983
1428619_at	2310005N03Rik	0.0009273	8.1506920
1428621_a_at	D11Wsu68e	0.0027954	8.5517267
1428624_at	2810482I07Rik	0.0028170	8.8944953
1428642_at	Frcl1	0.0014662	4.9623657
1428651_at	1110046J11Rik	0.0006130	6.9727930
1428666_at	Nars	0.0013085	9.8688152
1428670_at	2610305J24Rik	0.0006507	6.4576232
1428672_at	Snrpf	0.0004020	9.7811328
1428683_at	A930016P21Rik	0.0025319	5.3035499
1428688_at	Pdcd11	0.0015476	9.0104936
1428694_at	5033413D16Rik	0.0000740	8.6443241
1428697_at	Dpp8	0.0007326	6.4832866
1428738_a_at	D14Ertd449e	0.0031173	7.0172589

1428760_at	5031401C21Rik	0.0000858	8.1505237
1428782_a_at	Uqcrc1	0.0031330	10.2941061
1428802_at	Mgat3	0.0027953	3.2741474
1428820_at	Mapre1	0.0000855	6.6812734
1428825_at	Nr6a1	0.0020408	9.6529510
1428826_at	Nr6a1	0.0002439	9.5411509
1428850_x_at	2410026K10Rik	0.0006523	8.2965577
1428865_at	Bcl2l12	0.0000429	6.8856151
1428870_at	Nolc1	0.0000567	8.2948578
1428872_at	4121402D02Rik	0.0005432	6.3069048
1428874_at	1110019N10Rik	0.0005131	8.6506099
1428910_at	2310022B05Rik	0.0002765	8.6946846
1428926_at	1110003O08Rik	0.0009006	6.4936050
1428936_at	2810442I22Rik	0.0027954	8.5630759
1428946_at	5730469D23Rik	0.0000494	7.9275628
1428951_at	4921532D18Rik	0.0020895	7.3057236
1428987_at	Dncl2b	0.0009265	5.4648261
1429021_at	Epha4	0.0001277	7.2633774
1429038_at	1500034J01Rik	0.0004020	8.0481296
1429039_s_at	1500034J01Rik	0.0007337	8.0149074
1429040_at	2610005L07Rik	0.0000430	7.2647886
1429048_at	2410089B13Rik	0.0006507	8.1006150
1429060_at	9430072K23Rik	0.0007005	6.4421889
1429061_at	1810063B05Rik	0.0018276	7.1001643
1429079_a_at	2310045N01Rik	0.0025269	7.2007018
1429089_s_at	A530094D01	0.0009507	6.6748076
1429096_at	2810455D13Rik	0.0012346	6.0782821
1429103_at	Tomm22	0.0010400	9.3193163
1429107_at	1110059H15Rik	0.0027872	6.8560001
1429111_at	Tln2	0.0034768	7.9084999
1429117_at	Tradd	0.0021477	4.5475155
1429119_at	4833421E05Rik	0.0009273	7.2205344
1429126_at	2600001M11Rik	0.0005127	7.5706042
1429155_at	4933411K20Rik	0.0003773	7.0667215
1429169_at	Rbm3	0.0001659	7.3000545
1429177_x_at	Sox17	0.0000430	7.4009746
1429181_at	1700009P17Rik	0.0017282	5.4740662
1429198_at	1810030O07Rik	0.0002762	7.0567426
1429204_at	2900075A18Rik	0.0026306	6.2997126
1429232_at	2610528B01Rik	0.0034725	3.4066124
1429239_a_at	Stard4	0.0010186	6.2715600
1429240_at	Stard4	0.0002436	7.8052811
1429244_at	2610524H06Rik	0.0005783	7.3502268
1429261_at	2210411K11Rik	0.0000430	7.1319316
1429270_a_at	1700013H19Rik	0.0000430	9.1779451
1429276_at	C030048J01Rik	0.0019281	5.2401048
1429278_at	2410170E07Rik	0.0000430	7.9677398
1429283_at	1500009M05Rik	0.0016374	4.1980811
1429302_at	Csnk2a2	0.0000852	7.6502542
1429310_at	5530600M07Rik	0.0000430	7.1514895
1429331_at	4632427E13Rik	0.0000746	6.5032512
1429359_s_at	Rbpms	0.0005446	8.7019408
1429364_at	4930579G24Rik	0.0000429	7.0695058
1429370_a_at	Psmd11	0.0003553	8.9708820
1429375_at	1500026N15Rik	0.0014645	7.2913645
1429376_s_at	1500026N15Rik	0.0000990	8.3543413
1429388_at	Enk−pending	0.0000495	7.2168501
1429411_a_at	Ey2−pending	0.0020324	8.4104609
1429413_at	5730456K23Rik	0.0001362	7.7361687
1429454_at	4432404J10Rik	0.0034746	8.1405741
1429458_at	2410127L17Rik	0.0005783	9.3102315
1429461_at	2810417D08Rik	0.0000430	8.3143753
1429463_at	Prkaa2	0.0006512	6.0593215
1429466_s_at	0610008A10Rik	0.0004821	5.1491353
1429473_at	1200003I07Rik	0.0000746	6.5862664
1429476_s_at	Dnaja2	0.0000641	10.8418620
1429478_at	6720463M24Rik	0.0005105	6.6307848
1429502_at	Stch	0.0022643	5.6288509
1429514_at	Ppap2b	0.0026709	7.5047703
1429526_at	4432404P07Rik	0.0001044	6.6077707
1429587_at	D8Ertd233e	0.0001274	7.9957607
1429643_a_at	Pde1c	0.0034725	3.7383465
1429665_at	6230416J20Rik	0.0016374	5.3809445
1429681_a_at	Gpsn2	0.0000430	10.8486929
1429691_at	5430405N12Rik	0.0013076	4.9126511
1429707_at	Plaa	0.0001886	9.0681207
1429708_at	Ndufa11	0.0031330	9.5787641
1429734_at	4632434I11Rik	0.0022719	7.0480170
1429759_at	Rps6ka6	0.0001118	10.2522004
1429760_at	Rps6ka6	0.0001654	6.8120419
1429775_a_at	Tm7sf1	0.0008749	6.1872243
1429784_at		0.0000430	7.4795051
1429792_at	9530048O09Rik	0.0001766	6.2487242
1429815_at	4921513G22Rik	0.0008728	3.8457041
1429817_at	4933406N12Rik	0.0016509	5.9894192
1429821_at	2810046L04Rik	0.0034994	5.6690916
1429822_at	4633401B06Rik	0.0003773	5.1308433

1429839_a_at	Yaf2	0.0031305	7.5710964
1429846_at	9030411K21Rik	0.0001456	4.8252062
1429859_a_at	Arl2bp	0.0000430	7.9742430
1429871_at	Hmmr	0.0018263	5.8559423
1429882_at	2610005L07Rik	0.0000430	6.5429009
1429885_at	4930431P19Rik	0.0018225	1.8458355
1429891_at	1700028N11Rik	0.0028133	3.4615958
1429900_at	5330406M23Rik	0.0013057	2.9795203
1429907_at	1700094D03Rik	0.0017589	6.4526975
1429911_at	D030046N04Rik	0.0000429	7.1395108
1429936_at	2700059L22Rik	0.0006894	2.2501719
1429957_at	2310002B14Rik	0.0023963	5.9787200
1429979_a_at	1810073N04Rik	0.0022734	7.0054910
1429999_at	4933403M19Rik	0.0018200	5.0989902
1430000_at	B230117O15Rik	0.0003132	4.3313361
1430012_at	1110050K14Rik	0.0005109	6.0417656
1430021_a_at	Uble1a	0.0005131	9.3676621
1430023_at	5133400G04Rik	0.0006507	5.8596766
1430034_at	2610204B21Rik	0.0005100	6.5967819
1430048_at	7630402I04Rik	0.0006899	4.1773697
1430075_at	1810061H24Rik	0.0000430	8.1162056
1430092_at	Serac1	0.0031212	4.1101527
1430109_at	4833412E19Rik	0.0004528	6.3502255
1430123_a_at	Akr1a4	0.0005131	11.2835684
1430127_a_at	Ccnd2	0.0002291	8.9425763
1430128_a_at	Dp1l1	0.0001657	7.9032295
1430147_a_at	4930553M18Rik	0.0014687	7.1925628
1430161_at	Dlst	0.0003324	4.9633638
1430164_a_at	Grb10	0.0001886	6.5441987
1430195_at	2810043O03Rik	0.0000429	5.9487632
1430228_at	5730564G15Rik	0.0005788	7.6281075
1430272_at	2900056P18Rik	0.0034746	4.9648385
1430288_x_at	2410030A14Rik	0.0016362	12.4105418
1430309_at	4933421G18Rik	0.0001765	8.9124383
1430326_s_at	Qpc−pending	0.0003134	9.7179979
1430343_at	3830404O05Rik	0.0018212	6.4368526
1430368_s_at	1700019D03Rik	0.0000494	6.6585609
1430405_at	1700061I17Rik	0.0018200	3.4735699
1430493_at	2310061G22Rik	0.0034768	4.3240632
1430504_at	4930562N12Rik	0.0008966	1.7749516
1430519_a_at	Cnot7	0.0003550	8.0226855
1430526_a_at	2610209L14Rik	0.0008728	6.1376994
1430554_at	9430095K15Rik	0.0000430	7.3369781
1430555_s_at	9430095K15Rik	0.0000430	7.3596541
1430604_a_at	Dab2	0.0013066	6.9841010
1430619_a_at	Mvk	0.0005127	6.4151662
1430623_s_at	5830411E10Rik	0.0013008	6.3848742
1430648_at	2810451E09Rik	0.0002762	5.6720997
1430693_at	4833426H19Rik	0.0023963	5.3789823
1430702_at	5830427D03Rik	0.0032998	6.6717602
1430713_s_at	Grim19−pending	0.0000568	10.3634156
1430769_s_at	2900009I07Rik	0.0006998	7.1641857
1430780_a_at	Pmm1	0.0000567	7.0552457
1430889_a_at	Tpmt	0.0031311	5.9548593
1430980_a_at	Eif4a1	0.0000430	11.8156474
1430982_at	Sfrs1	0.0000495	9.7653925
1430985_at	1810027O10Rik	0.0001277	9.5541507
1430992_s_at	1500009M05Rik	0.0031133	5.9610381
1430999_a_at	Scoc	0.0000854	6.8256914
1431004_at	Loxl2	0.0002765	7.7513110
1431020_a_at	1500031J01Rik	0.0002591	7.6076698
1431032_at	Agl	0.0001451	5.7794301
1431033_x_at	Agl	0.0034673	5.6428532
1431037_a_at	Elavl1	0.0000495	9.6688114
1431053_at	B930097C17Rik	0.0000529	6.0214458
1431054_at	2410088K19Rik	0.0001888	9.1505196
1431086_s_at	Pcmt1	0.0006523	7.5060494
1431094_at	1110006E14Rik	0.0000430	7.7838581
1431117_x_at	1810029B16Rik	0.0032998	5.4161396
1431135_at	Itga6	0.0014078	1.4754801
1431182_at	Hspa8	0.0005788	7.9687653
1431188_a_at	Tom1	0.0015476	6.7345433
1431212_a_at	CGI−09−pending	0.0029664	6.4094717
1431225_at	Sox11	0.0000650	7.5909224
1431233_at	Cnnm4	0.0025319	6.3050499
1431241_at	0610041L09Rik	0.0003324	6.6439645
1431293_a_at	1110019C08Rik	0.0000430	8.2340920
1431314_a_at	5830417I10Rik	0.0000978	7.4628609
1431316_at	Itch	0.0010393	5.9413914
1431328_at	Ppp1cb	0.0006916	7.5910324
1431337_a_at	1810055E12Rik	0.0001454	7.4507043
1431353_at	C330050A14Rik	0.0009825	5.6263181
1431362_a_at	Smoc2	0.0001191	1.9850213
1431385_a_at	Mbtps1	0.0001044	6.9037385
1431393_at	4930447C04Rik	0.0002928	3.8509752
1431415_a_at	Tbpl1	0.0004528	8.0462405
1431420_s_at	2610524G07Rik	0.0000430	10.3863792

1431633_x_at	4930526L06Rik	0.0008246	2.5488036
1431645_a_at	Gdi3	0.0001453	9.3838096
1431708_a_at	Tia1	0.0000494	3.8735445
1431765_a_at	Rps2	0.0018289	13.0389952
1431771_a_at	Irak1bp1	0.0000650	6.7913028
1431777_a_at	Hmgn3	0.0000857	6.2634803
1431830_at	2810439M05Rik	0.0008728	6.2219491
1431834_a_at	Emilin1	0.0015605	3.6811222
1431877_a_at	Grhl2−pending	0.0028188	6.2460463
1431931_a_at	1110031N17Rik	0.0005436	7.4299435
1431960_at	Wwox	0.0026709	6.1298375
1431988_at	1700112E06Rik	0.0009532	4.1499413
1431997_at	Gapd	0.0017295	9.8966060
1432003_a_at	Rnf41	0.0000495	7.5490361
1432016_a_at	Idh3a	0.0001886	9.4058313
1432052_at	2610104C07Rik	0.0009280	5.5447382
1432164_a_at	5730591C18Rik	0.0000430	10.8312083
1432189_a_at	Sox5	0.0009265	3.1368186
1432195_s_at	Ccnl2	0.0015465	9.3814470
1432270_a_at	2210412K09Rik	0.0008742	9.2824438
1432331_a_at	Prrx2	0.0002765	5.8807567
1432393_a_at	5730409G07Rik	0.0000493	7.3821210
1432394_a_at	Aatf	0.0005127	7.5332384
1432464_a_at	2310057J16Rik	0.0006899	8.0242077
1432538_a_at	Rfc3	0.0001997	8.4181285
1432556_a_at	3100002J23Rik	0.0006161	2.5011437
1432603_at	Nrip1	0.0012221	5.3706481
1432646_a_at	2900097C17Rik	0.0000429	5.4597436
1432663_at	1500005C15Rik	0.0018212	2.9912803
1432747_at	4933403J19Rik	0.0026623	4.8182861
1432873_at	4932702M13Rik	0.0011646	2.5816247
1432921_at	4933416E14Rik	0.0026692	3.2928537
1433140_a_at	Nac1−pending	0.0003537	6.3670131
1433177_at	4933427C19Rik	0.0002755	2.2249438
1433218_at	4930566N20Rik	0.0024840	2.1877312
1433405_at	1700084K02Rik	0.0027933	4.1291172
1433442_at	C530050O22Rik	0.0017282	8.9942671
1433443_a_at	Hmgcs1	0.0000641	9.8792645
1433447_x_at	Cct4	0.0022750	11.3692350
1433457_s_at	Grsf1	0.0000990	9.5047442
1433466_at	AI467606	0.0002596	1.3760652
1433468_at	6430527G18Rik	0.0010408	6.4590421
1433475_a_at	C78339	0.0001886	8.5521266
1433486_at	Clcn3	0.0000430	7.9902698
1433487_at	Clcn3	0.0013085	8.0736963
1433489_s_at	Fgfr2	0.0004017	8.2814296
1433495_at	2810024B22Rik	0.0000651	8.7383268
1433496_at	2810024B22Rik	0.0023538	7.0987790
1433497_at	Aqr	0.0010416	8.8910261
1433502_s_at	AW550801	0.0005094	8.2432889
1433504_at	Pygb	0.0006517	7.1183204
1433508_at	Copeb	0.0000567	7.1785442
1433543_at	2900037I21Rik	0.0006523	8.8083646
1433560_at	9330175B01Rik	0.0021520	6.7385586
1433562_s_at	Atp5f1	0.0016362	10.9897508
1433594_at	D3Ertd176e	0.0022689	7.9034715
1433611_s_at	C77604	0.0001886	9.7513318
1433617_s_at	B4galt5	0.0015465	7.6482731
1433627_at	LOC207352	0.0009265	8.5510009
1433634_at	E130305N23Rik	0.0006517	8.3472885
1433640_at	D3Ertd330e	0.0025319	7.3701999
1433641_at	Madh5	0.0011681	7.3861359
1433645_at	2210409B22Rik	0.0001277	8.4069381
1433648_at	Spag9	0.0006910	7.6058075
1433656_a_at	Ns−pending	0.0002767	10.0173567
1433668_at	Prol2	0.0002767	9.3134544
1433674_a_at	Rnu22	0.0000567	10.4410698
1433675_at	Rnu22	0.0003134	9.3499164
1433685_a_at	6430706D22Rik	0.0001277	8.9860612
1433688_x_at	3100001N19Rik	0.0020408	11.5766091
1433696_at	D17Ertd441e	0.0006517	9.5065004
1433704_s_at	3100002M17Rik	0.0026709	8.9911497
1433706_a_at	Hspc121−pending	0.0000854	8.4544429
1433721_x_at	2410030A14Rik	0.0024953	12.4604637
1433730_at	9830169G11Rik	0.0032998	7.3776814
1433736_at	Hcfc1	0.0026692	9.6848193
1433738_at	5730445M16Rik	0.0000747	8.2560071
1433745_at	Trio	0.0001116	7.6072607
1433747_at	2010309L07Rik	0.0028115	6.6021567
1433748_at		0.0008253	6.9854907
1433751_at	2900042E17Rik	0.0005788	9.7133020
1433752_s_at	D030016E14Rik	0.0004536	7.8165782
1433771_at	Cgi67	0.0004532	7.7648266
1433777_at	4732493N06Rik	0.0005778	8.3216368
1433778_at	D130072O21Rik	0.0001116	8.9299735
1433795_at	Tgfbr3	0.0001116	7.8815366
1433806_x_at	Calr	0.0000495	11.3537935

1433810_x_at	Ddx5	0.0009825	6.6395993
1433815_at	5830437M04Rik	0.0018628	2.2533112
1433824_x_at	Grsf1	0.0000652	9.2814121
1433834_at	F830029L24Rik	0.0000568	9.2058380
1433849_at	Cdc27	0.0010998	6.7249384
1433856_at	AW555814	0.0008454	7.7558355
1433857_at	Fath	0.0001742	10.5881189
1433868_at	Btbd3	0.0001116	7.1467995
1433875_at	4732418C07Rik	0.0018289	8.1134382
1433881_at	E030019A03Rik	0.0008694	7.1071132
1433883_at	Tpm4	0.0000430	10.5057817
1433897_at	AI597468	0.0001884	7.8650126
1433907_at	Pknox2	0.0007783	7.3193207
1433908_a_at	Cttn	0.0028115	10.2147026
1433913_at	Rmp−pending	0.0008259	6.9025279
1433942_at	Myo6	0.0003547	6.6726733
1433955_at	Wdr9	0.0017282	6.6861940
1434001_at	2210019E14Rik	0.0026709	8.2577895
1434003_a_at	Dhps	0.0003432	8.1575074
1434004_at	Dhps	0.0005446	7.5053508
1434016_at	Znrf2	0.0014624	7.8673633
1434023_at	AU016693	0.0006115	7.6280485
1434035_at	Dnajb6	0.0001888	7.7833324
1434069_at	G630042G04	0.0005131	7.0198062
1434070_at	Jag1	0.0000978	7.9047299
1434081_at	Ap1g1	0.0001275	7.8602777
1434092_at	Nos3	0.0014687	4.4382260
1434097_at		0.0007337	7.2128915
1434105_at	A930003G21Rik	0.0025335	6.9894194
1434106_at	A930003G21Rik	0.0000430	7.0491917
1434116_at	Cbx2	0.0010416	8.9523764
1434122_at	E130008B10Rik	0.0034725	6.2796176
1434129_s_at	AI447312	0.0006140	7.3114334
1434135_at	D230016N13Rik	0.0016362	6.9298945
1434155_a_at	2310061I04Rik	0.0012337	7.8215769
1434165_at	5730466J16Rik	0.0004020	8.3584480
1434174_at	1110030H10Rik	0.0031271	6.7306308
1434176_x_at	Pdip46−pending	0.0008253	8.9663105
1434177_at	Ece1	0.0010416	7.0236676
1434178_at	Mll3	0.0013037	7.3674865
1434183_at	AA589507	0.0013085	7.8600249
1434187_at	B230397C21	0.0010416	7.5673472
1434194_at	Mtap2	0.0015454	5.2943504
1434202_a_at	MGC58343	0.0000429	4.9326212
1434206_s_at	Ppp2r5c	0.0001738	5.6867817
1434223_at	1810007P19Rik	0.0012337	5.4075383
1434229_a_at	Polb	0.0007005	7.3501943
1434230_at	Polb	0.0002145	7.9470580
1434235_at	Slc20a2	0.0013076	8.6223284
1434251_at	6030411K04Rik	0.0000430	10.2492244
1434265_s_at	Ank2	0.0010204	6.8580705
1434278_at		0.0001118	12.2033437
1434279_at		0.0000430	10.8090987
1434280_at		0.0003777	10.3092078
1434281_at	1500034J01Rik	0.0000429	8.2168816
1434292_at	E130013N09Rik	0.0022704	5.5078290
1434296_at	−−−	0.0001315	7.1003590
1434312_at	AI788669	0.0008253	7.6142360
1434316_at	Chsy1	0.0000567	8.3198030
1434328_at	Rpl15	0.0000430	10.1580715
1434337_at	Cklfsf3	0.0000652	9.0664102
1434340_at	1110020P15Rik	0.0001512	2.9860298
1434342_at	AI850290	0.0024896	2.3930583
1434353_at	Sfmbt2	0.0000746	6.7298396
1434357_a_at	Kpnb1	0.0001116	8.9178059
1434360_s_at	Ptprg	0.0000494	7.8140833
1434362_at	AW550831	0.0034768	9.2968286
1434367_s_at	Nutf2	0.0000652	12.1187122
1434375_at	B930006L02Rik	0.0017270	8.0172427
1434392_at	Murr2	0.0008469	9.1591568
1434393_at	Murr2	0.0014602	7.3787310
1434403_at	Spred2	0.0003553	8.9124626
1434436_at	5630401M14Rik	0.0001454	7.8540976
1434452_x_at	D3Ertd194e	0.0000650	7.4605442
1434455_at	Fbxo6a	0.0016374	2.6580530
1434462_at		0.0000852	7.1440662
1434468_at	4930431L18Rik	0.0000430	10.0692745
1434469_at	4930431L18Rik	0.0000430	8.7741229
1434480_at	4930402E16Rik	0.0001884	6.9388473
1434511_at	NfiX1−protein	0.0003773	6.7011314
1434518_at	Phka2	0.0013066	7.2415273
1434530_at	R75022	0.0002762	7.6551494
1434546_at	BC024683	0.0009601	8.0290626
1434557_at	Hip1	0.0011672	7.2781170
1434559_at	Stx3	0.0011023	6.6856706
1434579_x_at	Ndufs8	0.0000913	9.5269574
1434587_x_at	Ptdss2	0.0006905	7.9391628

1434588_x_at	Tbca	0.0017295	7.7902447
1434625_at	4930432O21Rik	0.0001736	5.6802285
1434627_at	Nrf1	0.0034725	7.8849800
1434628_a_at	Rhpn2	0.0002432	7.2227099
1434637_x_at	Sin3b	0.0000430	8.6837529
1434645_at	C530008M17Rik	0.0001657	7.2723359
1434700_at	6030408C04Rik	0.0006140	7.2195186
1434707_at	Sbf1	0.0025319	7.4890156
1434709_at	C130076O07Rik	0.0004463	5.4824580
1434718_at	Cul3	0.0016339	6.9619226
1434734_at	Fsbp−pending	0.0020352	4.3248045
1434739_at	NYSAR35	0.0000651	6.8215987
1434748_at	Ckap2	0.0001886	8.0759273
1434766_at	Prkaa2	0.0005089	5.4743118
1434773_a_at	Slc2a1	0.0001118	12.0207091
1434775_at	Pard3	0.0023979	8.1547605
1434788_at	D930050A07Rik	0.0000746	7.6765546
1434789_at	AW260467	0.0003336	7.2964951
1434801_x_at	Slc25a5	0.0001515	11.4491317
1434813_x_at	Wars	0.0029683	8.9991981
1434820_s_at	Pkig	0.0000855	7.7425342
1434853_x_at	Mkrn1	0.0001117	8.6988845
1434854_a_at	2210402A09Rik	0.0028188	12.3528142
1434870_at	2810004N23Rik	0.0001888	7.8622433
1434871_at		0.0006905	1.5274450
1434875_a_at	Hmgn3	0.0002436	7.3232424
1434879_at	Cdc34	0.0003134	10.3108023
1434897_a_at	Slc25a4	0.0000430	10.4417435
1434909_at	C030003H22Rik	0.0000977	8.8214688
1434935_at	5530400K14Rik	0.0006140	8.6553804
1434938_at	Rbm9	0.0029683	7.1663749
1434939_at	Foxf1a	0.0000430	8.5654756
1434949_at	1200015K23Rik	0.0021477	5.5109475
1434956_at	AI481227	0.0002425	6.5860732
1434962_x_at	Ccl27	0.0002139	5.6248251
1434967_at	2900036G02Rik	0.0001655	7.9280683
1434970_a_at	Mrpl15	0.0001765	9.0520649
1434971_x_at	Mrpl15	0.0001657	9.7232702
1434972_x_at	Sfrs1	0.0004544	11.0550423
1434975_x_at	9030221M09Rik	0.0000430	8.3639036
1434976_x_at	Eif4ebp1	0.0000978	10.3892727
1434981_at	E130303B06Rik	0.0004764	7.3971771
1434985_a_at	Eif4a1	0.0004544	11.6415131
1435011_x_at	Akr1a4	0.0004020	10.1898714
1435014_at	6330580M05Rik	0.0012337	5.4816962
1435016_at	4733401O11Rik	0.0002757	6.0422086
1435018_at	5930434B04Rik	0.0002284	7.5355843
1435019_at	E030022H21	0.0006523	9.1340697
1435022_at	Cart1	0.0020338	4.9598681
1435055_a_at	Tom1	0.0002767	8.2614252
1435056_x_at	BC003494	0.0016350	8.0550245
1435101_at	Flana−pending	0.0019255	6.8686323
1435103_x_at	Farsl	0.0007776	9.4013958
1435172_at	Eomes	0.0000430	7.5687589
1435176_a_at	Idb2	0.0000430	8.8135114
1435187_at	Tomm20−pending	0.0003333	7.4721525
1435223_at	BC036333	0.0005131	8.0186303
1435225_s_at	mKIAA1286	0.0013085	5.4379109
1435231_at	D2Ertd97e	0.0004472	7.7336671
1435232_x_at	Mrpl15	0.0018289	9.5698529
1435241_at	D930036F22Rik	0.0028188	7.1645515
1435267_at	A430108E01Rik	0.0000430	8.8761613
1435315_s_at	2900034E22Rik	0.0010400	7.2640659
1435321_at	3732412D22Rik	0.0008735	6.3404481
1435324_x_at	Hmgb1	0.0000568	11.8573047
1435327_at	AW112037	0.0007332	7.6164726
1435335_a_at	Mafb	0.0011646	6.8947737
1435340_at	LOC230674	0.0001454	6.1727130
1435353_a_at	4933439C20Rik	0.0001449	7.1437018
1435357_at	D4Wsu53e	0.0011672	5.6507963
1435372_a_at	Pa2g4	0.0015476	10.4820239
1435376_at	2010305K11Rik	0.0001886	7.1212699
1435379_at	mKIAA0133	0.0000430	8.0370591
1435381_at	3110080J08Rik	0.0010186	3.8658104
1435382_at	Ndn	0.0023979	8.5032898
1435397_at	BC038156	0.0002765	6.5821308
1435414_s_at	Dctn1	0.0028151	9.0723144
1435458_at	Pim1	0.0000652	7.2099406
1435462_at		0.0004764	4.9520008
1435475_at	Lman2l	0.0007776	8.3820145
1435484_at		0.0000854	7.8222739
1435514_at	Lztfl1	0.0005109	3.6746168
1435518_at	Rap1b	0.0009280	9.0806924
1435521_at	Msi2h	0.0006120	5.3993908
1435522_a_at	2310016E02Rik	0.0003547	10.1824307
1435524_at		0.0023995	8.8253427
1435534_a_at	Tomm20−pending	0.0000430	10.0277938

1435544_at	Mtr3−pending	0.0007343	8.9496864
1435550_at	C430014K11Rik	0.0000978	8.1808884
1435561_at	Erf	0.0002762	7.1493887
1435587_at	A730042J05Rik	0.0002436	7.2418589
1435596_at	A530088I07Rik	0.0023995	2.7093654
1435602_at	Sps2	0.0000430	9.5432983
1435628_x_at		0.0000494	7.5742589
1435630_s_at	Acat2	0.0031311	9.8880793
1435637_at	2310047C21Rik	0.0002432	5.4772908
1435653_at	Abhd2	0.0028188	8.3776947
1435659_a_at	Tpi	0.0000855	11.2073083
1435676_at	D030051D21	0.0001117	6.9030226
1435681_s_at	−−−	0.0025236	5.0293317
1435682_at	cartilage homeo protein 1	0.0000430	6.8124501
1435717_at	4833428C12Rik	0.0034618	7.0922875
1435777_at	C130081G24	0.0014091	4.8083661
1435778_at	3010027A04Rik	0.0000529	9.4034428
1435789_x_at		0.0001888	10.2402730
1435798_a_at	Sfrs14	0.0032998	7.6306201
1435803_a_at	Eif4el3	0.0000975	8.8133150
1435821_s_at	6330548N22Rik	0.0014634	7.6304259
1435836_at	D530020C15Rik	0.0004821	6.4936319
1435859_x_at	Psmc2	0.0026709	10.7338501
1435864_a_at	1810063B05Rik	0.0004006	8.0276324
1435869_s_at	Ap2a2	0.0004524	8.4846107
1435872_at	Pim1	0.0000430	6.6909196
1435892_at	D430002O22Rik	0.0023915	4.5029419
1435929_at	9630033F20Rik	0.0015476	5.1807096
1435973_at		0.0029664	5.5475882
1436000_a_at	Skp2	0.0018289	8.3359163
1436020_at	D8Ertd457e	0.0001456	7.3682578
1436026_at	1110032O19Rik	0.0009280	8.0535876
1436038_a_at	Dscr5	0.0005084	8.4235166
1436041_at	Hand2	0.0031291	6.7348252
1436048_at	AI414418	0.0000914	6.9320986
1436057_at	Gtl2	0.0032957	6.7465325
1436070_at		0.0023979	5.5692941
1436132_at	D430036N24Rik	0.0015476	5.8146495
1436158_at	Eif4ebp2	0.0000978	9.6875069
1436164_at	C130040I11Rik	0.0002282	6.9728849
1436170_a_at	Csng	0.0020338	2.9672001
1436178_at	AW553532	0.0008253	7.7087215
1436183_at	9830115L13Rik	0.0000607	6.3900649
1436192_at	AW493672	0.0001362	6.2428177
1436194_at	C330008K14Rik	0.0001408	7.1248264
1436202_at	9430072K23Rik	0.0014613	7.0885298
1436221_at	D1Ertd471e	0.0015476	5.2214031
1436240_at		0.0000430	5.9734493
1436266_x_at	Cbx1	0.0034789	9.6750499
1436270_at	−−−	0.0005790	6.4175140
1436275_at	Kcnip2	0.0028170	3.6191224
1436287_at		0.0000494	7.9020268
1436303_at	5033403D15	0.0035019	8.5354051
1436315_at	Myst3	0.0000429	8.3037840
1436317_at	9030223K07Rik	0.0031311	7.3024862
1436318_at	Tardbp	0.0020408	7.7084933
1436321_at	B3gnt7	0.0001659	7.9263092
1436322_a_at	2810001A02Rik	0.0009273	7.5104529
1436337_at		0.0014624	5.9885052
1436339_at	1810058I24Rik	0.0000746	8.9397980
1436457_at	F830028O17Rik	0.0029702	5.5989193
1436458_at	AI851735	0.0005127	5.8324900
1436460_at	BC030440	0.0003333	6.5230198
1436472_at		0.0007343	4.3070208
1436478_at	3010002C02Rik	0.0031311	4.3043107
1436494_x_at	D8Ertd812e	0.0000747	7.8112798
1436509_at	2410014A08Rik	0.0025319	8.8808929
1436517_at	H1fx	0.0001118	8.6160155
1436523_s_at	1810022K09Rik	0.0001453	10.0414194
1436539_at	9330188N17Rik	0.0031291	3.1703620
1436540_at		0.0006161	6.4469155
1436541_at	2310008H09Rik	0.0000854	7.9267930
1436543_at	BC034507	0.0001113	6.8202295
1436549_a_at	Hnrpa1	0.0019295	9.4230485
1436589_x_at	AI325941	0.0001361	4.3057245
1436595_at	D8Ertd233e	0.0011023	7.5494059
1436596_at	H2av−pending	0.0007469	6.2278630
1436654_at	5830483C08Rik	0.0013804	6.3670383
1436677_at	1810032O08Rik	0.0000430	6.1207966
1436710_at	E130119J17Rik	0.0018276	6.4908334
1436713_s_at		0.0000429	8.7995270
1436714_at	Lpp	0.0003914	7.1490414
1436715_s_at	Cdipt	0.0012355	8.1211514
1436736_x_at	D0H4S114	0.0003207	8.1451699
1436739_at	Agtr1	0.0019228	5.3928087
1436740_at	2610005L07Rik	0.0000428	7.9274070
1436750_a_at	Oxct	0.0000429	7.2916284

1436757_a_at	Cox6b	0.0001277	11.3578369
1436764_at	Pard3	0.0000567	7.7603687
1436765_at	Pard3	0.0015465	5.5358977
1436775_a_at	Ankrd17	0.0034768	9.1292369
1436784_x_at	Sf3b4	0.0034789	8.3947100
1436790_a_at	Sox11	0.0000568	8.9273089
1436791_at	Wnt5a	0.0006135	6.6816128
1436796_at	1110061A14Rik	0.0005783	8.1758565
1436797_a_at	Surf4	0.0002593	9.0750755
1436803_a_at	Ndufb9	0.0000854	9.8365188
1436808_x_at	Mcm5	0.0000430	11.2434958
1436810_x_at	2900010M23Rik	0.0012355	7.7021217
1436812_at	Fkrp−pending	0.0026692	7.5209264
1436816_at	4832420O05Rik	0.0007476	8.7387309
1436817_at	AI448003	0.0002434	7.7265770
1436869_at	Shh	0.0007343	6.6851977
1436871_at	Sfrs7	0.0000430	7.8391979
1436874_x_at	Slc25a5	0.0020408	11.5255521
1436885_a_at	Cherp	0.0000745	9.0232955
1436890_at	5730445F03Rik	0.0021520	6.3670762
1436893_a_at	Axot	0.0000568	8.2931927
1436897_at	Mfhas1	0.0005441	6.8978812
1436902_x_at	Tmsb10	0.0000430	11.9617923
1436908_at	Pcm1	0.0008246	6.9440583
1436919_at	LOC277414	0.0000855	7.9888721
1436921_at	Atp7a	0.0028151	7.2565490
1436922_at	2410005L11Rik	0.0020338	5.2018538
1436925_at	Ches1	0.0000976	7.2960520
1436930_x_at	Hmbs	0.0003550	7.9219365
1436934_s_at	Aco2	0.0005127	8.3554032
1436936_s_at	Xist	0.0000495	9.5956838
1436938_at	6720477E09Rik	0.0001763	5.0630429
1436944_x_at	9030221M09Rik	0.0000652	8.3927597
1436958_x_at	Tpm3	0.0004017	10.4243516
1436962_at	−−−	0.0000430	7.4834524
1436989_s_at	Slc12a6	0.0014091	3.8900370
1436990_s_at	1620401E04Rik	0.0029683	7.6691398
1436993_x_at	Pfn2	0.0010851	7.5530164
1436995_a_at	Rpl26	0.0006523	12.7559361
1437005_a_at	Rpl18	0.0011681	12.8416718
1437018_at	Pnma2	0.0000746	4.6389110
1437026_at	3010020C06	0.0017575	7.0880467
1437027_x_at	Rnps1	0.0001277	10.8562374
1437056_x_at	1810049K24Rik	0.0034746	3.1201826
1437070_at	A530086E13Rik	0.0002767	7.5482198
1437073_x_at	AV025504	0.0034789	7.7051564
1437074_at		0.0034789	7.8119925
1437103_at	C330012H03Rik	0.0001766	9.2261221
1437108_at	2410088K19Rik	0.0002942	7.3168473
1437109_s_at	2410088K19Rik	0.0001277	9.2200117
1437121_at	4432409M07Rik	0.0005790	1.8171849
1437127_at	C730040L01Rik	0.0002141	6.1143459
1437142_a_at	Pigo	0.0009818	8.0816457
1437148_at	Arpc2	0.0010385	10.7016634
1437161_x_at	Rbpms	0.0000495	8.9484820
1437162_at		0.0000494	7.6953253
1437163_x_at	Gtf2h4	0.0011007	8.9917133
1437165_a_at	Pcolce	0.0000973	6.1660996
1437172_x_at	Hadhb	0.0006507	8.0933213
1437181_at	Peli2	0.0020380	6.2997561
1437193_s_at	Snrpb	0.0001118	11.1216456
1437194_x_at	1200011O22Rik	0.0013076	9.2253438
1437206_at	2900045N06Rik	0.0032957	7.4094559
1437211_x_at	Elovl5	0.0003137	9.8397665
1437223_s_at	Xbp1	0.0024934	9.7251195
1437237_x_at		0.0003134	7.0759458
1437278_a_at	Uble1b	0.0014634	10.3718564
1437283_at	Kpnb2b−pending	0.0009273	7.7983099
1437289_at	1110001C20Rik	0.0019770	8.1247220
1437303_at	Il6st	0.0000430	7.0712813
1437308_s_at	F2r	0.0003553	9.4697773
1437313_x_at	Hmgb2	0.0028188	10.8673671
1437314_a_at	D8Ertd812e	0.0004544	7.7110381
1437335_x_at	Pdip46−pending	0.0004017	9.0285192
1437350_at	Ovca2−pending	0.0015443	6.8202316
1437358_at	Wdfy1	0.0002439	7.7061229
1437359_at	Rnps1	0.0033067	7.1670329
1437360_at	LOC279653	0.0000430	8.8131849
1437372_at	4733401N12Rik	0.0002944	7.1218171
1437378_x_at	Scarb1	0.0002147	6.8115171
1437395_at	LOC230595	0.0022734	8.7584372
1437405_a_at	Igfbp4	0.0000697	10.6670563
1437417_s_at	Gpc6	0.0018263	5.8166218
1437426_at	−−−	0.0008220	7.0800034
1437436_s_at	Gprk6	0.0028170	8.5568405
1437445_at	Trpm1	0.0031291	1.9364139
1437458_x_at	Clu	0.0000740	6.0605517

1437465_a_at	P4hb	0.0004020	11.6536926
1437478_s_at	D4Wsu27e	0.0000975	7.0828203
1437536_at	Fkrp−pending	0.0002286	5.8338616
1437543_at	D3Ertd330e	0.0021520	8.0149705
1437558_at	B130021B11Rik	0.0034768	6.3158689
1437604_x_at	AB023957	0.0023979	6.7780812
1437626_at		0.0003553	9.6521312
1437634_at	6330441O12Rik	0.0007332	7.0639128
1437649_x_at	Ppib	0.0000495	11.1194140
1437658_a_at	Rnu22	0.0000991	8.3266118
1437670_x_at	Cd151	0.0023979	8.7004774
1437687_x_at	Fkbp9	0.0022689	9.3867890
1437689_x_at	Clu	0.0020366	5.0825522
1437690_x_at	Csnk1d	0.0004536	9.6302334
1437696_at	BC049807	0.0012189	5.6978620
1437703_at	Fbxo12	0.0008233	8.0965120
1437707_at		0.0016304	5.9024239
1437708_x_at	Vamp3	0.0008749	8.8423804
1437709_x_at	BC017545	0.0002286	8.4075304
1437711_x_at	Odc	0.0001659	11.2448915
1437713_x_at	1500010J02Rik	0.0013815	5.2462531
1437717_x_at	2610005L07Rik	0.0000430	7.8042712
1437723_s_at	1110021N07Rik	0.0003553	9.5781562
1437745_at	A730019I05Rik	0.0003336	7.6033402
1437759_at	Pfkp	0.0017295	6.0406269
1437769_at		0.0022704	6.1775497
1437783_x_at	Tmem4	0.0000430	10.0338878
1437821_at		0.0003544	6.7747989
1437838_x_at	Grsf1	0.0003137	10.4320990
1437845_x_at	BC003494	0.0016362	9.0924702
1437850_a_at	Cnbp	0.0026709	9.5196508
1437853_x_at	Ndn	0.0004544	7.8400818
1437859_x_at	Eif5a	0.0000855	11.8148344
1437883_s_at	2700050F09Rik	0.0018212	5.9818107
1437901_a_at	Vps41	0.0002286	7.1964665
1437923_at	AI314760	0.0010408	7.4797046
1437936_at		0.0016550	5.6958299
1437943_s_at	Mea1	0.0016350	6.8686360
1437947_x_at	Vdac1	0.0001118	8.7348799
1437965_at	MGC30806	0.0017546	6.3768068
1437993_x_at	Qdpr	0.0000697	9.7840958
1438009_at	Hist1h2ae	0.0000747	12.6422413
1438020_at	Crtl1	0.0000429	5.4128368
1438021_at	BC013481	0.0006910	6.3679127
1438025_at	9130004K12Rik	0.0013028	6.1346461
1438030_at	Rasgrp3	0.0000430	6.9586768
1438032_at	mKIAA1016	0.0032895	5.0231533
1438046_at	AU019823	0.0022734	6.4296720
1438049_at	A430108E01Rik	0.0000747	8.6098272
1438058_s_at	Ptov1	0.0007343	10.3252554
1438094_x_at	2810409H07Rik	0.0006512	8.9685764
1438104_at		0.0024746	5.1233654
1438118_x_at	Vim	0.0000651	9.7630143
1438138_a_at	Pex6	0.0003336	7.1057883
1438152_at	Gpaa1	0.0012346	7.3751207
1438155_x_at	Pigo	0.0013085	7.9997723
1438167_x_at	B430214A04Rik	0.0006910	7.4272740
1438168_x_at	Ddx39	0.0000568	10.8348989
1438199_at		0.0031232	6.5384788
1438200_at	Sulf1	0.0002000	8.4560138
1438219_at	Pura	0.0016304	2.0815190
1438237_at		0.0011681	6.1376568
1438238_at	2010315B03Rik	0.0002427	2.9704226
1438239_at	Mid1	0.0002439	7.7775229
1438251_x_at	Prss11	0.0017282	5.5648609
1438278_a_at	BC003993	0.0001277	7.9548998
1438287_x_at	Ddx39	0.0016550	5.9915409
1438292_x_at	Adk	0.0008469	10.0289767
1438307_at		0.0005441	6.2905821
1438315_x_at	Akr7a5	0.0028188	8.7413411
1438316_a_at	BC027663	0.0003123	5.7457062
1438333_at	A230098A12Rik	0.0002765	8.8215998
1438339_at	Fancd2	0.0003126	6.3763370
1438345_at		0.0014645	6.6394876
1438360_x_at	Slc25a5	0.0018238	9.2378237
1438365_x_at	Laptm4b	0.0018289	11.5495210
1438366_x_at	Clcn3	0.0000430	9.0362394
1438390_s_at	Pttg1	0.0001659	10.9527567
1438391_x_at	Hadh2	0.0018276	6.5227976
1438398_at	Rnpc2	0.0016362	7.2554146
1438402_at	9630050M13Rik	0.0024934	6.6306173
1438403_s_at	Ramp2	0.0004010	8.5088012
1438408_at	5730467H21Rik	0.0016550	6.4471682
1438410_at	A230098A12Rik	0.0009280	10.6217711
1438454_at	B430203M17Rik	0.0000746	8.0750404
1438463_x_at	Zdhhc6	0.0009825	7.9100579
1438501_at	Rps17	0.0002434	7.3880015

1438507_x_at	3100001N19Rik	0.0002944	11.8231647
1438516_at		0.0000652	7.7764942
1438528_at		0.0000988	6.4120600
1438537_at		0.0010408	6.2540690
1438545_at	Slc25a5	0.0002286	8.6501800
1438546_x_at	Slc25a5	0.0020394	9.5433988
1438560_x_at	Cct4	0.0018276	10.3294955
1438576_x_at		0.0023852	3.6563899
1438606_a_at	Clic4	0.0026709	9.7972444
1438631_x_at	BC017545	0.0000650	8.2164684
1438637_x_at	Sf3b2	0.0005094	10.0232790
1438638_x_at	1700027J05Rik	0.0009825	7.5434514
1438640_x_at	Pgk1	0.0005788	12.9304045
1438647_x_at	Cetn2	0.0005127	8.3183900
1438649_x_at	Pbp	0.0001275	12.0676040
1438651_a_at	Agtrl1	0.0000641	10.6237145
1438656_x_at	Timm17b	0.0031237	9.0809806
1438658_a_at	Edg3	0.0000977	5.0795349
1438686_at	Eif4g1	0.0014634	7.0512917
1438688_at	5033413A03Rik	0.0000430	11.4533803
1438714_at	Zfp207	0.0001888	9.4243372
1438723_a_at	2210402A09Rik	0.0005788	12.8174061
1438757_at	C130069I09	0.0010842	6.4898635
1438761_a_at	Odc	0.0000697	11.6975177
1438787_at	−−−	0.0023995	9.2775050
1438788_at	D5Wsu152e	0.0003773	7.1394109
1438794_x_at	Rps13	0.0017560	11.6285402
1438810_at	D10Ertd755e	0.0002010	6.4707517
1438846_x_at	Ihpk1	0.0026675	0.8995812
1438847_at	2610524G07Rik	0.0034789	6.5938904
1438883_at	Fgf5	0.0013835	6.2803452
1438908_at	Map3k12	0.0031330	8.5602531
1438910_a_at	Epb7.2	0.0001654	6.3658632
1438916_x_at	5830467J12Rik	0.0034661	6.2595375
1438921_at		0.0009237	4.5074010
1438922_x_at	Slc25a5	0.0002765	8.9963792
1438938_x_at	Bcap37	0.0003137	10.2691876
1438941_x_at	Ampd2	0.0001411	9.1122394
1438957_x_at	Cds2	0.0000429	9.5491706
1438961_s_at	Blmh	0.0001655	9.6989175
1438968_x_at	Spint2	0.0011663	8.6331728
1438972_x_at	2810410L24Rik	0.0028170	6.7544507
1438992_x_at	Atf4	0.0027975	8.7264661
1438999_a_at	Nfat5	0.0000430	6.7167571
1439018_at	6330505N24	0.0019281	7.2426810
1439024_at	Bag4	0.0011015	7.7475435
1439050_at	AI649393	0.0011672	5.5044193
1439057_x_at	Zdhhc6	0.0022750	9.2474056
1439058_at	5730453G22Rik	0.0003132	6.7951158
1439059_at	BC031748	0.0000430	6.6238002
1439065_x_at	−−−	0.0000430	7.4310286
1439069_a_at	9030221M09Rik	0.0000430	8.4871581
1439094_at	Cltc	0.0014645	6.6902643
1439111_at		0.0000495	6.4418759
1439148_a_at	Pfkl	0.0013085	11.1883091
1439161_at	D19Ertd703e	0.0011672	6.8032750
1439163_at		0.0008998	6.9960983
1439174_at		0.0000744	4.7418456
1439216_at		0.0005735	4.9801575
1439224_at		0.0002804	4.1920795
1439235_x_at	Blp1−pending	0.0005436	7.7759712
1439241_x_at	Srd5a2l	0.0009584	6.7857464
1439252_at	Incenp	0.0004260	6.1232149
1439253_x_at	2610524G07Rik	0.0000430	10.6710031
1439266_a_at	1500004O14Rik	0.0001888	8.6463225
1439267_x_at	Cox5a	0.0022146	10.6571922
1439276_at		0.0029608	5.9876222
1439295_x_at		0.0003003	7.0566901
1439300_at	Chic1	0.0000798	4.8486501
1439301_at		0.0006899	5.8382157
1439311_at	B830012L14Rik	0.0001509	5.9392701
1439316_at	Rnpc2	0.0001768	7.6158050
1439321_at		0.0028151	6.4547616
1439341_at		0.0003553	9.6838937
1439364_a_at	Mmp2	0.0007476	8.5327872
1439366_at	Grsf1	0.0034703	3.1315768
1439371_x_at	Timm44	0.0009280	8.1206810
1439376_x_at		0.0008239	4.1213678
1439380_x_at	Gtl2	0.0000430	10.5842309
1439387_x_at	2310061F22Rik	0.0002000	8.3993661
1439399_a_at	Rnu22	0.0007783	10.1264658
1439403_x_at	Rnf12	0.0006916	7.1896876
1439413_x_at	Morf4l2	0.0008477	11.7611565
1439427_at	Cldn9	0.0002807	4.4526078
1439432_x_at	Morf4l2	0.0031330	11.3570560
1439433_a_at	Slc35a2	0.0001654	6.9036793
1439435_x_at	Pgk1	0.0020394	9.8939194

1439441_x_at	Lats2	0.0034768	5.2880627
1439443_x_at	Tkt	0.0022750	11.3671922
1439463_x_at	Hmgb1	0.0000430	11.3353164
1439466_s_at	1110069M14Rik	0.0000430	9.9028224
1439470_at		0.0004825	6.6789642
1439477_at	5430406J06Rik	0.0001117	7.2111812
1439483_at	AI506816	0.0000430	6.8513779
1439498_at	2810401C09Rik	0.0000746	8.1360889
1439553_s_at	Nutf2	0.0000430	11.0775596
1439560_x_at		0.0009265	6.6781369
1439562_at	F730047E07Rik	0.0001117	7.1682405
1439578_at	2210404M20Rik	0.0010408	5.9552321
1439607_at		0.0024840	1.7659023
1439630_x_at	Sbsn−pending	0.0009584	5.6164197
1439638_at	Erbb2ip	0.0026605	5.3908252
1439650_at	Rtn4	0.0000430	7.3518489
1439658_at	5430424A14Rik	0.0018250	2.6147847
1439663_at	Ptch	0.0006910	6.6282035
1439678_at		0.0010998	5.7854613
1439757_s_at	Epha4	0.0010337	5.0673673
1439764_s_at	C330012H03Rik	0.0009601	9.2218132
1439778_at	Cables1	0.0034789	2.2986990
1439780_at	1500016H10Rik	0.0000650	6.5546879
1439805_at	Nfat5	0.0009006	5.9947816
1439811_at	Mtr	0.0007337	7.0318360
1439840_at	A430088C08Rik	0.0023963	5.9956336
1439856_at	Acvr2b	0.0003550	8.0136036
1439882_at		0.0003767	6.6024545
1439943_at	Vps54	0.0007940	3.6845231
1439948_at	BC046401	0.0016362	6.0698658
1439955_at	A630082N15Rik	0.0010393	1.6395052
1439988_at	C81203	0.0017197	5.3031459
1440011_at		0.0004540	7.4477859
1440037_at		0.0000652	8.3777660
1440040_at		0.0029589	5.0099564
1440046_at	BC031748	0.0034768	4.7620843
1440107_at		0.0016550	6.1039197
1440162_x_at	A630043P06	0.0024820	4.8798468
1440168_x_at	9430010P06Rik	0.0002012	7.9320295
1440184_at	A430108E01Rik	0.0010851	5.3783906
1440223_at	Rbm6	0.0001044	6.9577083
1440227_at		0.0002134	6.2386557
1440228_at	C630001B19	0.0027872	5.8208487
1440248_at	D130060C09Rik	0.0004792	5.2869677
1440254_at		0.0003330	7.5452753
1440267_at	E330005K07Rik	0.0003531	5.0142161
1440278_at	Dnclc1	0.0001116	5.4224135
1440316_at	6430526N21Rik	0.0024821	5.3727737
1440341_at		0.0026286	4.9359029
1440421_at		0.0004536	6.2890665
1440452_at	Drp2	0.0014613	6.6090478
1440461_at		0.0006507	5.8619671
1440490_at		0.0005079	4.6373110
1440511_at		0.0021981	5.8427611
1440542_at		0.0006580	7.5597813
1440553_at	Nrbf1	0.0032936	3.3523533
1440565_at		0.0015420	2.3099426
1440594_at		0.0005131	6.0434412
1440624_at		0.0021477	2.0709048
1440715_s_at	D11Ertd497e	0.0011672	8.1381066
1440717_at	AA407881	0.0010851	5.6736067
1440755_at		0.0016234	5.2782624
1440771_at	9130423L19Rik	0.0011681	4.7339154
1440815_x_at	1700049E17Rik	0.0009244	2.4435520
1440816_x_at	Ddx1	0.0023979	9.9926340
1440822_x_at	Reps1	0.0017282	8.3056787
1440825_s_at	1700009P13Rik	0.0013825	5.5635612
1440857_at	2410008J01Rik	0.0001550	6.1676203
1440861_a_at	Kcnc3	0.0024915	6.3995289
1440862_at		0.0008735	5.9133658
1440870_at	5730557K01Rik	0.0017234	3.6199174
1440880_at	Mppe1−pending	0.0018225	6.1468380
1440915_at	−−−	0.0000567	6.2651173
1440935_at		0.0034746	7.3663528
1440962_at	Slc8a3	0.0008749	2.5119562
1440966_at	Axot	0.0028170	7.5877367
1440972_at	Nsd1	0.0003553	5.9778543
1440990_at	4832420M10	0.0005783	6.6471914
1441001_at	AI225934	0.0004013	5.6958080
1441050_at		0.0000977	5.0953054
1441052_at		0.0021535	6.1139283
1441072_at		0.0031271	6.3060987
1441097_at		0.0004168	5.2273412
1441177_at		0.0026692	6.9946751
1441248_at	Clcn3	0.0002613	6.4208643
1441274_at		0.0008239	6.8500090
1441275_at	Takrp−pending	0.0032977	5.7808109

1441315_s_at	Slc19a2	0.0007462	5.0584496
1441328_at		0.0013794	6.2931971
1441354_at	AU018740	0.0002996	5.7802343
1441359_at	9230115F04Rik	0.0009265	5.5473977
1441362_at		0.0005489	4.9663579
1441379_at	AU045094	0.0034661	1.2472721
1441400_at	1810029B16Rik	0.0012241	6.2323538
1441409_at	AI449705	0.0029570	2.8089506
1441452_at		0.0018250	5.9311066
1441558_at	D230044B12Rik	0.0029645	6.5550274
1441682_s_at	1110004L07Rik	0.0001657	9.0643689
1441684_at	2610202A04Rik	0.0009258	5.6493765
1441689_at	Nup153	0.0004267	6.0657884
1441736_at		0.0019295	3.4852712
1441746_at		0.0003518	3.3854977
1441789_at		0.0003432	4.5682537
1441814_s_at	2400006N03Rik	0.0034746	6.5780503
1441816_at	2900056M20Rik	0.0010385	6.2919790
1441823_at		0.0018276	5.8401633
1441870_s_at	Pkd2	0.0002147	8.7080817
1441894_s_at		0.0010842	7.1057390
1441911_x_at	Gart	0.0003547	7.1754471
1441942_x_at	Rnut1	0.0007770	5.8896825
1441943_x_at	2810413N20Rik	0.0004180	7.6676872
1441953_at	4933409N07Rik	0.0000639	6.0281003
1441955_s_at	Paip1−pending	0.0000429	5.4898948
1441959_s_at	1200003C05Rik	0.0000855	7.5483662
1442025_a_at	AI467657	0.0009244	4.2610828
1442028_at	AI593864	0.0020394	6.8555530
1442044_at	Rps6	0.0006905	5.6148549
1442062_at	7120426M23Rik	0.0000494	6.2867211
1442083_at	1500011J06Rik	0.0000430	6.5691144
1442094_at	Ing5	0.0001113	7.3333797
1442099_at	6330567E21Rik	0.0005105	4.6411381
1442124_at	AI450326	0.0008735	6.4324767
1442129_at	1810058I24Rik	0.0025286	6.0777115
1442148_at	5031401C21Rik	0.0001657	7.7019547
1442174_at	6720430O15	0.0004256	6.7122592
1442213_at		0.0013076	3.9875165
1442235_at		0.0009251	5.2623332
1442258_at	1300002A08Rik	0.0006905	5.3949499
1442311_at		0.0034746	6.4325990
1442340_x_at	Cyr61	0.0002627	5.5241871
1442358_at	AA409587	0.0013076	7.3055047
1442385_at		0.0019281	1.8618679
1442402_at	Posh−pending	0.0001742	5.5108836
1442410_at	AV016528	0.0020366	4.0474249
1442442_at		0.0000794	5.4276053
1442511_at	Ipo7	0.0034682	6.5448263
1442594_at	Ttk	0.0004271	6.0746529
1442604_at		0.0032998	5.0895804
1442644_at		0.0005790	5.1868856
1442655_at		0.0005446	8.0064916
1442723_at		0.0009265	3.8986102
1442744_at	C79248	0.0005778	5.6103837
1442750_at	D230016N13Rik	0.0017295	4.0849424
1442793_s_at	Tbrg4	0.0001888	9.0603656
1442800_x_at	A830059I20Rik	0.0011015	4.0911505
1442804_at	Fgr	0.0006115	4.8072971
1442806_at	9430030N17Rik	0.0031173	4.0287428
1442865_at	C130007D14	0.0001117	6.9709221
1442899_at		0.0024988	3.1330462
1442933_at	6230415M23Rik	0.0005429	6.5498480
1442945_at	2010109K09Rik	0.0029551	4.3787213
1443003_at		0.0006448	3.9908782
1443026_at		0.0022750	6.7334301
1443037_at		0.0000853	6.5922870
1443052_at		0.0008200	3.9830241
1443072_at	Pkd1l1	0.0005778	5.9817859
1443077_at	9430041J06Rik	0.0014539	5.1510340
1443099_at		0.0022163	4.3420610
1443104_at		0.0002346	4.5399089
1443146_at	2610509G12Rik	0.0005418	5.9153647
1443166_at		0.0015044	5.6540813
1443240_at		0.0006501	7.6396229
1443296_at	Pctk1	0.0026692	5.3156544
1443314_at	2410042D21Rik	0.0001277	6.1678179
1443337_at		0.0000697	6.9020309
1443366_at	Napg	0.0034994	5.5344027
1443489_at		0.0000975	6.5792116
1443522_s_at	Phip	0.0022163	7.1398975
1443526_at		0.0014624	6.9336950
1443649_at		0.0001551	6.7358243
1443772_at		0.0002765	3.9963070
1443792_at	Tsga14	0.0034746	4.1818933
1443814_x_at	Ctsh	0.0000854	9.0080161
1443862_at		0.0016339	6.9410281

1443893_at		0.0002439	5.8252764
1443905_at		0.0001043	5.8336722
1443935_at	KIAA0240	0.0006161	5.9377466
1443948_at	2210019E14Rik	0.0026692	6.8257675
1443954_at	Rad18	0.0001765	6.6831881
1444009_at	Rassf4	0.0020268	1.5377348
1444139_at	1700037B15Rik	0.0026365	5.5242687
1444188_at		0.0019281	4.9492995
1444194_at		0.0001657	6.5104795
1444341_at	8030451F13Rik	0.0031251	4.4860581
1444343_at		0.0033020	5.6714284
1444379_at	D930023J19Rik	0.0022198	5.2955910
1444396_at	1110029F20Rik	0.0010823	3.0493746
1444406_at		0.0022146	5.9738586
1444416_at		0.0006512	4.1614232
1444458_at		0.0003760	5.4934840
1444552_at		0.0034789	4.6255249
1444583_at		0.0000567	7.0236206
1444620_at		0.0013037	4.5954201
1444676_at		0.0009584	5.7000527
1444722_at		0.0014674	5.9579281
1444753_at	Nek7	0.0021535	5.4536266
1444761_at		0.0001044	6.6221052
1444765_at	Rbpms	0.0018263	6.0893207
1444774_at		0.0022734	2.2541911
1444845_at	3110021P21Rik	0.0018238	4.7904286
1445097_at		0.0016550	4.6278801
1445183_s_at	D7Ertd523e	0.0020366	5.0760765
1445186_at	Stc2	0.0028115	6.4916137
1445239_at	BC031407	0.0001553	7.2784844
1445264_at	Catsper2	0.0010408	4.7298403
1445372_at		0.0013066	4.2040988
1445387_at	Susp1−pending	0.0001190	5.1247100
1445495_at		0.0000568	6.2151689
1445535_at	AA407107	0.0008728	4.8475934
1445602_at		0.0025319	4.6809182
1445630_at		0.0032895	5.1421667
1445689_at		0.0001657	6.4380705
1445773_at		0.0002616	5.2737699
1445815_at	Fzd8	0.0020380	3.1895343
1445867_at	AL023008	0.0009810	6.4255844
1445941_at		0.0019295	4.4718893
1446048_at		0.0001886	6.2103071
1446086_s_at	Gli2	0.0004524	6.8640494
1446094_at		0.0001879	6.7255000
1446118_at	D17Ertd165e	0.0013853	5.5684005
1446127_at		0.0002593	5.2470335
1446147_at	C79248	0.0000430	8.7639956
1446148_x_at	C79248	0.0000652	8.3259600
1446193_at		0.0025252	4.1474954
1446196_at	Hmga2	0.0014662	5.7276121
1446330_at		0.0013057	4.0610139
1446344_at	Nyx	0.0013804	4.7407584
1446346_at	Adamts9	0.0003123	5.8500737
1446464_at	Psme4	0.0018225	5.1064558
1446550_at	Gspt1	0.0014592	6.9788159
1446693_at		0.0002141	5.3032155
1446700_at		0.0007770	4.6182165
1446713_at		0.0000494	5.5029753
1446732_at		0.0025302	6.1318643
1446737_a_at	E330005F07Rik	0.0010385	5.9307186
1446807_at	Usp8	0.0006517	5.9507272
1446856_at	D3Wsu167e	0.0008239	3.9604549
1446897_at		0.0014528	5.2254251
1446899_at		0.0001112	5.9804360
1446926_at	Asc−pending	0.0012310	5.1513177
1446933_at	Cml66−pending	0.0014560	4.8272684
1446951_at	D930031A02Rik	0.0009780	3.3197807
1446953_at		0.0001116	6.5927666
1446957_s_at	N4bp1−pending	0.0001888	7.3087619
1446972_at	D15Wsu126e	0.0034725	6.7072945
1446982_at		0.0006561	5.4214140
1447024_at		0.0002755	5.4638193
1447108_at		0.0009795	5.4366905
1447181_s_at	Slc7a7	0.0012252	6.8435112
1447320_x_at	Rpo1−3	0.0000430	10.0258568
1447360_at	Tgfb1i4	0.0000430	6.9579245
1447374_at		0.0020324	5.8850332
1447448_s_at		0.0015618	5.5502622
1447465_at		0.0005131	5.6072516
1447470_at		0.0020352	5.0400756
1447567_at		0.0012319	5.7437060
1447576_at	2010001K21Rik	0.0029478	2.1298716
1447581_at		0.0003652	2.7391069
1447643_x_at	Snai2	0.0023520	6.8518745
1447666_x_at	3200002M19Rik	0.0013076	6.0121699
1447720_x_at	Prkaca	0.0002143	7.9214731

1447734_x_at	Aldo1	0.0000494	10.1070721
1447754_x_at	2010320B01Rik	0.0028170	7.2500646
1447767_at		0.0033020	5.5034725
1447780_x_at	2300002G02Rik	0.0009494	7.6517306
1447822_x_at	2700038N03Rik	0.0000854	6.0888542
1447825_x_at	Pcdh8	0.0015593	7.5024493
1447833_x_at	Mfap2	0.0005788	5.4249250
1447883_x_at	1010001H21Rik	0.0008253	8.7417790
1447894_x_at	Vps52	0.0001454	5.3829822
1447896_s_at	2010109N14Rik	0.0000991	11.0901394
1447904_s_at	Fnta	0.0000430	10.3367856
1447931_at	Whsc1l1	0.0028097	6.7174702
1447936_at	2410006H16Rik	0.0023995	8.7362675
1448029_at	Tbx3	0.0002427	7.3369409
1448103_s_at	Nono	0.0014645	11.4498855
1448117_at	Kitl	0.0003134	6.6482794
1448121_at	Wbp2	0.0005122	8.0725131
1448122_at	Tcp1	0.0025319	11.6814383
1448127_at	Rrm1	0.0000430	9.5190328
1448133_at	C87860	0.0013030	8.2506699
1448135_at	Atf4	0.0009280	9.4438393
1448140_at	2810413N20Rik	0.0001118	8.5121615
1448145_at	Wwp2−pending	0.0001274	6.8338573
1448147_at	Tnfrsf19	0.0000429	8.7218006
1448152_at	Igf2	0.0000652	10.7822651
1448153_at	Cox5a	0.0018289	11.2157163
1448154_at	Ndr2	0.0029589	6.5811125
1448156_at	Tff1	0.0021520	8.8017065
1448166_a_at	Psmb1	0.0008259	10.4192645
1448169_at	Krt1−18	0.0005131	10.5883075
1448179_at	Usmg5	0.0000853	9.7039135
1448182_a_at	Cd24a	0.0004184	11.5980371
1448185_at	Herpud1	0.0001766	6.9758054
1448188_at	Ucp2	0.0018289	7.8835305
1448194_a_at	H19	0.0000495	11.7932731
1448195_at	Taf5l	0.0010416	7.5550832
1448199_at	Ankrd10	0.0000430	8.9829014
1448202_x_at	2610524G07Rik	0.0000430	10.5165557
1448208_at	Madh1	0.0000858	8.3599128
1448210_at	Rab1	0.0000430	8.3543028
1448212_at	Clast3−pending	0.0005783	9.0966379
1448215_a_at	Dpp3	0.0004540	8.8308841
1448218_s_at	Ywhaz	0.0018289	9.9801079
1448223_at	Fto	0.0000495	8.2957544
1448226_at	Rrm2	0.0016362	10.8546652
1448229_s_at	Ccnd2	0.0000430	7.4321394
1448230_at	Uchrp	0.0000430	9.8814137
1448233_at	Prnp	0.0001117	7.5620814
1448234_at	Dnajb6	0.0001659	9.6344219
1448235_s_at	Hmgb1	0.0000430	9.0180263
1448237_x_at	Ldh2	0.0010408	9.5537019
1448240_at	Mbtps1	0.0001659	9.8467819
1448244_at	Lypla1	0.0020394	8.1064268
1448254_at	Ptn	0.0000977	7.1674066
1448259_at	Fstl	0.0000430	8.9046580
1448261_at	Cdh1	0.0005788	10.5591201
1448263_a_at	0610010E05Rik	0.0034768	9.4888809
1448277_at	Pold2	0.0000855	8.6541283
1448286_at	Hadh2	0.0001659	8.6269568
1448287_at	Rpo1−3	0.0031330	9.6063874
1448289_at	Crmp1	0.0000429	8.0105476
1448292_at	Uqcr−pending	0.0001275	9.3489545
1448315_a_at	Leftb	0.0000853	8.0091411
1448319_at	Akr1b3	0.0014645	9.9327402
1448321_at	Smoc1	0.0000567	7.3605647
1448322_a_at	Cox4a	0.0000429	11.2307358
1448325_at	Myd116	0.0028170	7.4146710
1448326_a_at	Crabp1	0.0000651	7.9730030
1448331_at	Ndufb7	0.0000697	9.1620405
1448335_s_at	Ccni	0.0035019	8.9157319
1448364_at	Ccng2	0.0013825	6.3335125
1448368_at	Dctn6	0.0001513	7.5233846
1448369_at	Pola2	0.0000430	7.8636610
1448372_a_at	Tmem4	0.0000855	9.7046930
1448388_a_at	1110002B05Rik	0.0010408	9.1295297
1448392_at	Sparc	0.0001659	9.0761581
1448395_at	Sfrp1	0.0010408	8.8055403
1448406_at	Cri1	0.0002000	8.4099598
1448424_at	Frzb	0.0000430	7.3067711
1448425_at	Eif3s10	0.0031330	11.4687681
1448445_at	Acp6	0.0001275	6.9666045
1448449_at	Ripk3	0.0003153	5.3891332
1448455_at	Cln8	0.0009273	7.3669879
1448472_at	Vars2	0.0000495	9.1134848
1448473_at	Bub3	0.0006916	7.8677767
1448474_at	Nek7	0.0007314	7.2914975
1448480_at	1110017C15Rik	0.0000430	9.3812767

1448493_at	Paip2−pending	0.0013076	10.2570289
1448501_at	Tm4sf6	0.0001454	9.4636970
1448509_at	3110001A13Rik	0.0034725	6.4014086
1448516_at	Tsn	0.0008233	8.5783254
1448521_at	Brd7	0.0000430	8.3827474
1448523_at	Nphp1	0.0029645	4.6898451
1448524_s_at	Ssr4	0.0000495	8.9894905
1448533_at	Ckap1	0.0028170	9.6059371
1448543_at	2310042G06Rik	0.0009273	7.8786691
1448545_at	Sdc2	0.0001766	6.6532923
1448569_at	Cd8b	0.0001768	9.0873340
1448577_x_at	Syngr2	0.0008742	8.2400314
1448579_at	Glg1	0.0000429	9.3344419
1448580_at	Glg1	0.0001118	8.0223131
1448586_at	Hsp70−4	0.0000647	9.1299124
1448597_at	Cstf1	0.0015618	8.3271738
1448601_s_at	Msx1	0.0017282	8.2990151
1448604_at	Uck2−pending	0.0018289	8.6652402
1448606_at	Edg2	0.0001768	8.4912675
1448611_at	D8Ertd594e	0.0000494	7.3320888
1448622_at	Lsm4	0.0000430	10.2771658
1448624_at	Cd2bp2	0.0020366	8.0769929
1448641_at	Mbtd1	0.0014645	9.3295243
1448653_at	Eed	0.0034768	9.3314145
1448654_at	Mtch2	0.0000429	6.9573794
1448664_a_at	Apeg1	0.0003553	7.0124812
1448677_at	Noc4	0.0000430	9.4204936
1448679_at	Hyal2	0.0034789	8.1301892
1448682_at	Dnclc1	0.0009280	11.6561728
1448685_at	2900010M23Rik	0.0002147	9.3475984
1448688_at	Podxl	0.0004540	10.9545406
1448689_at	Rras2	0.0032998	9.0406216
1448690_at	Kcnk1	0.0000495	8.2411359
1448691_at	Ubin−pending	0.0020408	8.9883659
1448692_at	Ubin−pending	0.0019308	7.6138418
1448702_at	1110057H19Rik	0.0001655	8.4731606
1448704_s_at	H47	0.0005131	9.4751893
1448732_at	Ctsb	0.0000495	10.0244577
1448736_a_at	Hprt	0.0007343	9.9418743
1448737_at	Tm4sf2	0.0000746	8.8911759
1448753_at	Srp9	0.0001888	7.8775757
1448754_at	Rbp1	0.0000855	9.2665474
1448769_at	Ugalt2	0.0002765	9.2206309
1448771_a_at	Fth	0.0010416	11.3972983
1448784_at	Taf10	0.0006512	10.6082546
1448791_at	Snx5	0.0000652	10.8280141
1448794_s_at	Zrf2	0.0031311	8.7113779
1448795_a_at	Tbrg4	0.0013076	8.7003034
1448797_at	Elk3	0.0000743	5.0977367
1448801_a_at	Timm44	0.0001515	9.6325075
1448808_a_at	Nme2	0.0020408	12.1031891
1448816_at	Ptgis	0.0000652	8.3782744
1448818_at	Wnt5a	0.0005769	6.9771346
1448820_a_at	Eif2s2	0.0011672	10.0432912
1448823_at	Cxcl12	0.0032977	7.6804810
1448830_at	Dusp1	0.0000495	7.1650724
1448833_at	Foxm1	0.0028170	7.5340554
1448835_at	E2f6	0.0005441	8.1635218
1448844_at	Cyb5m−pending	0.0000430	7.8862239
1448849_at	Mrpl40	0.0011672	8.8896401
1448864_at	Snrk	0.0003137	6.6407238
1448882_at	0610009E20Rik	0.0015605	8.8878673
1448883_at	Lgmn	0.0000495	9.9007791
1448884_at	Gtf2e2	0.0000430	9.8268185
1448885_at	Rap2b	0.0007337	6.8386138
1448889_at	Slc38a4	0.0000430	8.9336115
1448899_s_at	Rad51ap1	0.0008728	7.9433592
1448904_at	D6Wsu176e	0.0032998	7.1766779
1448907_at	Thop1	0.0010393	9.0092216
1448928_at	Hdac6	0.0000495	9.0077805
1448943_at	Nrp	0.0001659	8.2746798
1448944_at	Nrp	0.0012346	5.8558915
1448947_at	2810004N23Rik	0.0000430	9.6964735
1448960_at	4930415K17Rik	0.0001888	8.5335087
1448970_at	1200007B05Rik	0.0034703	7.6977171
1448971_at	2410022L05Rik	0.0002765	7.5574219
1449003_a_at	Vti1b	0.0011672	8.8720470
1449005_at	Slc16a3	0.0026640	10.3349269
1449007_at	Btg3	0.0000640	9.2356777
1449017_at	Nutf2	0.0000747	7.8587767
1449018_at	Pfn1	0.0005131	11.4327973
1449039_a_at	Hnrpdl	0.0001456	10.6685485
1449042_at	Ctcf	0.0006916	8.7418050
1449046_a_at	1110007C05Rik	0.0018276	6.1382196
1449059_a_at	Oxct	0.0000855	8.7422284
1449064_at	Tdh	0.0004544	8.3566350
1449066_a_at	Arhgef7	0.0000745	7.7889392

1449071_at	Mylc2a	0.0000798	7.3095490
1449082_at	Mfap5−pending	0.0005740	4.8381953
1449085_at	1810055P05Rik	0.0014687	9.1746506
1449090_a_at	Yes	0.0018136	6.6953244
1449098_a_at	Poli	0.0000647	5.4811543
1449117_at	Jund1	0.0001277	9.2608788
1449118_at	Dbt	0.0002942	7.1140668
1449120_a_at	Pcm1	0.0001275	6.4156876
1449126_at	Zfp90	0.0004821	6.3344960
1449137_at	Pdha1	0.0000430	8.7623274
1449140_at	D11Ertd603e	0.0015465	8.4912158
1449141_at	2410043F08Rik	0.0008253	7.4851577
1449155_at	2310047G20Rik	0.0001882	7.5628604
1449168_a_at	Akap2	0.0017589	6.8759907
1449186_at	Bag4	0.0006135	7.6781568
1449187_at	Pdgfa	0.0010861	7.2589045
1449210_at	Igf2bp1	0.0000652	8.6218788
1449219_at	Fads3	0.0020352	0.9966175
1449236_at	Dll3	0.0001456	8.2136794
1449303_at	Sesn3	0.0011672	7.7982877
1449315_at	Odz3	0.0011023	8.2473842
1449319_at	Rspondin−pending	0.0010823	5.8566624
1449342_at	6330408J20Rik	0.0000855	9.3565516
1449347_a_at	Xlr4	0.0021506	7.1146915
1449365_at	Edg8	0.0029627	2.7064668
1449391_at	Zfp37	0.0025236	6.0833899
1449397_at	Hoxb2	0.0003137	7.9455691
1449412_at	1810046J19Rik	0.0011015	8.2212634
1449434_at	Car3	0.0000567	6.9098418
1449448_at	BC019367	0.0034994	7.4883905
1449459_s_at	Asb13	0.0020927	6.1237137
1449473_s_at	Tnfrsf5	0.0006507	3.5573563
1449506_a_at	Eef1d	0.0010408	10.9629767
1449534_at	Sycp3	0.0006135	5.6615283
1449552_at	Zfr	0.0011672	7.4247894
1449553_at	2610200G18Rik	0.0002147	8.1445614
1449561_at	4921504I05Rik	0.0010177	3.6726875
1449578_at	Supt16h	0.0005131	8.5724692
1449582_at	Cdx1	0.0000430	7.4662330
1449592_at	Tcf15	0.0011681	6.4835143
1449615_s_at	Hdlbp	0.0003336	6.9504367
1449628_s_at	Stard7	0.0001888	9.7156930
1449633_s_at	C330027I04Rik	0.0016362	6.2854843
1449645_s_at	Cct3	0.0006517	9.9439937
1449674_s_at	Pdcd6ip	0.0008253	7.1781316
1449681_at	Hdgf	0.0004821	6.5179470
1449699_s_at	C330027C09Rik	0.0027954	7.3356713
1449724_s_at	D8Ertd738e	0.0000430	9.2590993
1449730_s_at	Fzd3	0.0032977	6.1180050
1449806_at		0.0013076	4.7221870
1449839_at	Casp3	0.0022750	9.8682076
1449848_at	Gna14	0.0013066	6.1165595
1449859_at	CGI−141−pending	0.0000430	4.6150135
1449862_a_at	Pi4k2b−pending	0.0009265	6.6845645
1449868_at	Tbx6	0.0001116	7.9355500
1449889_a_at	6030432N09Rik	0.0012337	9.7219856
1449893_a_at	Lrig1	0.0002012	6.0883067
1449899_at	Grin3b	0.0023947	4.6112833
1449903_at	Crtam	0.0004524	3.4109720
1449933_a_at	5730449L18Rik	0.0001453	7.9365562
1449939_s_at	Dlk1	0.0011015	9.4201797
1449944_a_at	Sec61a2−pending	0.0019770	8.4375142
1449972_s_at	Zfp97	0.0000991	7.2780020
1450011_at	Hsd17b12	0.0004544	9.1842574
1450017_at	Ccng1	0.0000494	6.7253351
1450036_at	Sgk3	0.0002439	7.4152666
1450045_at	Srrm1	0.0005446	6.6384567
1450048_a_at	Idh2	0.0012355	9.0671171
1450061_at	Enc1	0.0001659	7.8306637
1450062_a_at	Maged1	0.0022750	9.0483495
1450070_s_at	Pak1	0.0001361	8.2190180
1450079_at	Nrk	0.0000430	7.9872636
1450102_a_at	Amfr	0.0003550	8.6486006
1450117_at	Tcf3	0.0001453	8.1910633
1450118_a_at	Tnnt3	0.0010990	4.4884650
1450131_a_at	Bspry	0.0007343	6.3392224
1450150_a_at	Rpl13	0.0000914	12.7336666
1450186_s_at	Gnas	0.0003553	11.1881896
1450187_a_at	Galt	0.0019308	7.7653634
1450256_at	Cer1	0.0001118	7.4388957
1450257_at	Cer1	0.0005131	6.7066025
1450269_a_at	Pfkl	0.0000430	10.7316822
1450380_at	Epdm2−pending	0.0004540	6.5467067
1450390_x_at	Rps18	0.0031271	5.8050943
1450407_a_at	Anp32a	0.0000430	9.1435336
1450409_a_at	4930570C03Rik	0.0018250	4.2754531
1450416_at	Cbx5	0.0009825	8.2436076

1450418_a_at	2310034L04Rik	0.0033091	5.5771293
1450506_a_at	2700083B06Rik	0.0034789	7.7421983
1450510_a_at	Cacna1a	0.0006496	3.8027183
1450519_a_at	Prkaca	0.0002767	8.6341798
1450522_a_at	H1f0	0.0034789	8.9916971
1450567_a_at	Col2a1	0.0013853	6.9438363
1450634_at	Atp6v1a1	0.0000567	9.0165524
1450641_at	Vim	0.0002439	10.1918695
1450643_s_at	Facl2	0.0015465	5.7159742
1450649_at	Gng10	0.0004544	8.3521389
1450650_at	Myo10	0.0007337	8.2608267
1450657_at	Ppie	0.0006586	7.7656463
1450660_at	Pts	0.0000493	6.2776580
1450677_at	Chek1	0.0018225	7.3649098
1450694_at	Fkbp2	0.0002033	8.3162659
1450700_at	Cdc42ep3	0.0026536	4.3013638
1450722_at	Nup50	0.0013085	8.5626274
1450723_at	Isl1	0.0000914	6.8514149
1450729_at	Hs2st1	0.0003137	8.2115665
1450744_at	Ell2−pending	0.0006517	6.3468108
1450757_at	Cdh11	0.0000568	8.6499473
1450779_at	Fabp7	0.0001659	6.4116362
1450813_a_at	Tnni1	0.0000796	5.4028979
1450843_a_at	Serpinh1	0.0000430	10.2479447
1450852_s_at	F2r	0.0003922	9.0625621
1450858_a_at	1100001F19Rik	0.0008253	9.8369847
1450866_a_at	Mrpl17	0.0001888	9.8053909
1450873_at	Gtpbp4	0.0028188	9.5056405
1450895_a_at	1810020G14Rik	0.0026692	7.4575209
1450903_at	Rad23b	0.0011681	9.8160990
1450907_at	5730406I15Rik	0.0002593	9.2839720
1450911_at	Ppib	0.0001659	11.1221069
1450913_at	B4galt6	0.0015465	7.0905965
1450919_at	Mpp1	0.0013835	7.8605742
1450924_at	Hdgfrp3	0.0001116	6.9596301
1450934_at	Eif4a2	0.0000430	10.8641791
1450943_at	2010012C16Rik	0.0000650	7.8315172
1450947_at	2610528J11Rik	0.0014634	7.3767835
1450970_at	Got1	0.0022734	8.9335292
1450981_at	Cnn2	0.0000568	8.7742322
1450989_at	Tdgf1	0.0000430	6.1958963
1450990_at	Gpc3	0.0011681	10.3809027
1450992_a_at	Meis1	0.0001057	6.5052056
1450995_at	Folr1	0.0034768	7.8446372
1451000_at	1810020E01Rik	0.0012346	9.3884479
1451011_at	Zfp358	0.0001116	7.2376142
1451019_at	Ctsf	0.0003003	5.1199107
1451035_a_at	Akr1a4	0.0007343	10.3664798
1451042_a_at	Mina	0.0002143	8.1722488
1451046_at	Zfpm1	0.0000429	8.0024265
1451047_at	Itm2a	0.0003550	7.9357547
1451052_at	2610019N19Rik	0.0011663	8.4746848
1451056_at	Psmd7	0.0000430	9.1959940
1451058_at	2400002F11Rik	0.0000493	8.2564858
1451064_a_at	Psat1	0.0013841	9.5534159
1451065_a_at	Ddx39	0.0000430	10.1308257
1451071_a_at	Atp1a1	0.0000568	9.7927792
1451081_a_at	D8Ertd325e	0.0000651	8.1904252
1451091_at	Txndc5	0.0001659	9.4533610
1451097_at	Vasp	0.0002944	8.1654403
1451098_at	Pcoln3	0.0001274	8.5376918
1451101_a_at	Rps28	0.0003553	12.7314856
1451105_at	B130052G07Rik	0.0005783	7.8790959
1451109_a_at	Nedd4	0.0002439	11.1257227
1451110_at	Egln1	0.0000529	7.4450668
1451111_at	4832420O05Rik	0.0000529	8.1255782
1451119_a_at	Fbln1	0.0014645	9.1150395
1451120_at	Rpo1−3	0.0000568	8.7162682
1451124_at	Sod1	0.0005131	10.6435241
1451130_at	2010315L10Rik	0.0001659	8.5781374
1451131_at	Arl6ip1	0.0000529	10.0020256
1451134_a_at	Blp1−pending	0.0001143	9.4104785
1451138_x_at	2700087H15Rik	0.0028188	7.5148585
1451144_at	1110064N10Rik	0.0001886	9.7514211
1451146_at	Zfp386	0.0000430	6.9020127
1451152_a_at	Atp1b1	0.0024953	8.5252661
1451162_at	Hsbp1	0.0002145	8.8555454
1451167_at	1700023O11Rik	0.0010186	6.9330845
1451169_at	Pm5−pending	0.0002429	7.1250441
1451171_at	2310008H04Rik	0.0025252	7.2005494
1451172_at	1200015A19Rik	0.0001317	9.1256870
1451175_at	1810011E08Rik	0.0000430	9.2820162
1451188_at	Wdr26	0.0001454	8.0515684
1451190_a_at	Sbk−pending	0.0000651	8.2381673
1451191_at	Crabp2	0.0001116	8.7405488
1451195_a_at	Txndc1	0.0005769	9.0337362
1451197_s_at	BC031407	0.0000430	9.5171997

1451201_s_at	Rnh1	0.0004817	7.8781397
1451216_at	Noa36−pending	0.0000430	8.6672281
1451217_a_at	1500034J20Rik	0.0031330	8.4524903
1451224_at	Scamp5	0.0015476	7.1711767
1451227_a_at	Slc10a3	0.0011015	5.9842272
1451229_at	MGC27683	0.0005118	4.9338436
1451237_s_at	1500011D06Rik	0.0034746	9.2068320
1451248_at	BC006705	0.0000747	8.5705154
1451249_at	D8Ertd812e	0.0000430	8.1242586
1451255_at	Lisch7−pending	0.0032936	9.2424449
1451262_a_at	Jtv1−pending	0.0003137	9.7033445
1451283_at	9030624B09Rik	0.0000854	7.3025701
1451294_s_at	Snrpe	0.0022750	11.5226043
1451310_a_at	Ctsl	0.0000652	11.4489569
1451324_s_at	3830421F13Rik	0.0020394	6.8909490
1451347_at	AI854876	0.0025319	6.6815537
1451351_at	BC017545	0.0001886	8.4884396
1451358_a_at	Racgap1	0.0023947	8.3095554
1451359_at	BC005662	0.0013853	7.9520412
1451370_at		0.0013028	5.2560050
1451383_a_at	Chuk	0.0014602	6.7412645
1451387_s_at	0610039D01Rik	0.0005122	9.0014212
1451393_at	4632428M11Rik	0.0029702	6.7943875
1451397_at	A830080H02Rik	0.0008259	7.2864629
1451398_at	BC009118	0.0000428	7.3843213
1451411_at	Gprc5b	0.0010973	5.5522462
1451437_at	5033406L14Rik	0.0009006	6.8806043
1451458_at	Tmem2	0.0001277	8.8879803
1451459_at	Elys−pending	0.0000858	8.8298204
1451470_s_at	Eif5a	0.0008259	12.5704537
1451480_at	E2f4	0.0001997	8.0823281
1451545_at	Tdrd3	0.0002284	7.5901841
1451559_a_at	Dhrs4	0.0026675	6.7652766
1451580_a_at	Ttr	0.0000495	10.7366972
1451586_at	Tegt	0.0022750	10.0220534
1451588_at	1810022C23Rik	0.0004248	4.2293577
1451618_at	Rho	0.0019281	6.3245258
1451621_at	5830417C01Rik	0.0011646	8.0163220
1451652_a_at	5033428A16Rik	0.0000495	8.6306787
1451665_a_at	Ap4s1	0.0011023	7.6556767
1451676_at	Drap1	0.0000744	8.2030408
1451692_at	2410015B03Rik	0.0021535	6.5143464
1451703_s_at	Aprt	0.0001277	10.6901714
1451734_a_at	Dbn1	0.0002145	7.7255715
1451736_a_at	Map2k7	0.0001553	9.0144187
1451768_a_at	Slc20a2	0.0025236	7.6904104
1451784_x_at	H2−K	0.0034919	6.1743125
1451786_at	MGC31081	0.0002012	5.1579061
1451805_at	Phip	0.0017589	5.4387031
1451884_a_at	Lsm2	0.0034768	9.3348687
1451896_a_at	Cherp	0.0000430	9.5612199
1451935_a_at	Spint2	0.0011681	9.1753430
1451971_at	Cul4a	0.0016563	7.7708713
1451978_at	Loxl	0.0021520	5.6461998
1451979_at	4930469P12Rik	0.0001659	8.6388029
1451980_at	Cas1−pending	0.0010400	8.1049732
1451991_at	Epha7	0.0026675	3.7231788
1451994_s_at	Npac−pending	0.0003134	9.0845740
1452004_at	Calca	0.0021491	5.5225860
1452011_a_at	Uxs1	0.0013841	6.3571769
1452020_a_at	Siva−pending	0.0011672	10.5954616
1452035_at	Col4a1	0.0001888	10.3397528
1452036_a_at	Tmpo	0.0029683	10.3402122
1452044_at	2010015J01Rik	0.0009825	9.2731297
1452049_at	1500016H10Rik	0.0007343	9.4639370
1452051_at	Actr3	0.0035043	10.0401401
1452058_a_at	Rnf11	0.0029513	8.2290530
1452062_at	Prpsap2	0.0004544	8.5195503
1452077_at	Ddx3y	0.0016362	6.2903307
1452080_a_at	Tes3−ps	0.0032998	7.0523755
1452096_s_at	BC006874	0.0002593	7.9408025
1452100_at	Dullard	0.0022719	8.8144054
1452114_s_at	Igfbp5	0.0000494	7.0999674
1452119_at	2600005C20Rik	0.0007770	8.2827613
1452125_at	9330151F09Rik	0.0010416	8.6098645
1452128_a_at	C6.1A−pending	0.0020394	8.3538607
1452167_at	2810407C02Rik	0.0020408	10.0007641
1452168_x_at	Gspt1	0.0002145	9.9383829
1452169_a_at	Dgkz	0.0000429	9.0284332
1452183_a_at	Gtl2	0.0000854	10.2910870
1452191_at	2510048K03Rik	0.0000978	5.9733081
1452192_at	LOC234344	0.0000430	9.6375705
1452207_at	Cited2	0.0000651	10.1705691
1452208_at	2810470D21Rik	0.0031311	7.3578012
1452214_at	9130011J04Rik	0.0028188	8.2305941
1452219_at	BC026370	0.0031328	7.5421030
1452223_s_at	2900054P12Rik	0.0029478	4.7655738

1452232_at	Galnt7	0.0000430	8.1514657
1452249_at	Prickle1	0.0010408	7.3773619
1452267_at	E030034P13Rik	0.0018263	7.5449076
1452270_s_at	Cubn	0.0001515	10.7653753
1452294_at	2010005A06Rik	0.0004013	6.3883978
1452304_a_at	Arhgef5	0.0005122	7.1342196
1452305_s_at	2610510J17Rik	0.0000652	7.9906718
1452318_a_at	Hspa1b	0.0018212	5.6567506
1452321_at	D530019K20Rik	0.0015465	6.8483977
1452323_at	BC008150	0.0007332	6.4714306
1452336_at	BC027382	0.0011681	7.0047603
1452364_at	D11Ertd530e	0.0013076	8.1781557
1452401_at	Wtap	0.0000854	9.5334364
1452402_at	Uchl3	0.0004540	5.9420788
1452427_s_at	Hspc121−pending	0.0017234	7.2827456
1452428_a_at	B2m	0.0003137	8.1365779
1452430_s_at	Sfrs1	0.0004528	10.3465636
1452448_at	Aqr	0.0009258	6.0015474
1452458_s_at	2410005L11Rik	0.0022198	7.1183527
1452497_a_at	Nfatc3	0.0017270	7.1847496
1452513_a_at	Fanca	0.0009258	5.7303501
1452534_a_at	Hmgb2	0.0000567	11.0698071
1452540_a_at	Hist1h2bc	0.0015476	8.0465201
1452545_a_at	Itgb1	0.0003137	9.2625529
1452582_at	A530057M15Rik	0.0015476	7.3796496
1452593_a_at	2610301I15Rik	0.0006507	4.7233265
1452603_at	5330431N19Rik	0.0007776	7.4410467
1452623_at	LOC268670	0.0029570	4.5371486
1452635_x_at	4930553M18Rik	0.0001277	7.4974507
1452654_at	Zdhhc2	0.0013066	7.1805152
1452662_a_at	Eif2s1	0.0006517	10.5732888
1452664_a_at	Tm7sf3	0.0011672	6.2837943
1452670_at	Myl9	0.0005127	5.8929752
1452679_at	2410129E14Rik	0.0008742	7.3185605
1452690_at	Khsrp	0.0005788	7.6882792
1452701_x_at	Uba52	0.0000495	12.3686548
1452712_at	2610510D13Rik	0.0000494	8.9151994
1452713_a_at	Prp8bp−pending	0.0022689	8.6053024
1452719_at	5730496N17Rik	0.0032925	5.4393088
1452725_a_at	2400006P09Rik	0.0025319	8.5199888
1452730_at	1110033J19Rik	0.0000640	9.6136108
1452731_x_at	2610042L04Rik	0.0000430	8.7305459
1452734_at	0610007O07Rik	0.0000430	9.4351272
1452735_at		0.0000430	9.5286767
1452740_at	Myh10	0.0013085	10.3716389
1452754_at	5730592L21Rik	0.0004821	8.5357306
1452755_at	0910001B06Rik	0.0018263	5.3581989
1452767_at	Rrbp1	0.0002147	9.5945792
1452769_at	3732413I11Rik	0.0004544	10.0485899
1452773_at	5730494N06Rik	0.0000430	8.5693254
1452774_at	2610510D13Rik	0.0000568	8.9664273
1452789_at	Snn	0.0006135	8.2617213
1452790_x_at	Ndufa3	0.0003333	9.9705827
1452795_at	1110008B24Rik	0.0020408	9.4848662
1452810_at	4921521J11Rik	0.0003132	8.6932423
1452812_at	Lphn1	0.0001118	7.8259442
1452813_a_at	5033428A16Rik	0.0003126	7.9139375
1452816_at	1700029A22Rik	0.0001621	5.7306947
1452820_at	Wdr11	0.0005131	7.9548537
1452821_at	Tial1	0.0000652	8.0763779
1452840_at	1500009L16Rik	0.0031282	5.7373142
1452847_at	2410008K03Rik	0.0007337	7.1821783
1452849_at	Sin3b	0.0011015	7.2139299
1452856_at	1110034C16Rik	0.0007337	7.9842659
1452877_at	2700029M09Rik	0.0000494	9.6022293
1452881_at	4833427B12Rik	0.0000855	8.3424289
1452896_at	Gtl3	0.0000430	8.4753766
1452898_at	2810408E15Rik	0.0003134	8.2861486
1452899_at	Rian	0.0006140	7.6038145
1452901_at	3526402H21Rik	0.0002767	9.2063349
1452905_at	Gtl2	0.0011681	9.7628325
1452907_at	Galc	0.0005118	5.9032123
1452916_at	2610014H22Rik	0.0022719	8.5636132
1452917_at	Rfc5	0.0007343	9.2488391
1452919_a_at	1700012G19Rik	0.0006586	8.9512953
1452923_at	1810058I14Rik	0.0000991	7.3898202
1452927_x_at	Tpi	0.0005788	11.6331988
1452931_at	5630401H01Rik	0.0005127	5.8221351
1452945_at	2610020C11Rik	0.0002286	6.7415257
1452956_a_at	D12Ertd647e	0.0010990	4.9945202
1452968_at	Cthrc1	0.0000428	6.7738302
1452971_at	Upf3a	0.0003333	7.5329108
1452982_at	A330103N21Rik	0.0014602	8.4441576
1452989_at	2900009J20Rik	0.0002139	6.1188041
1452997_at	2610005L07Rik	0.0000430	9.9145325
1453001_at	2610207P08Rik	0.0006517	8.3504655
1453004_at	3110004L20Rik	0.0006916	7.5675948

1453006_at	2610306H15Rik	0.0022628	5.3459270
1453009_at	1110060I01Rik	0.0000567	5.8821350
1453015_at	5830471E12Rik	0.0020408	8.6598703
1453019_at	1200009I24Rik	0.0009810	6.1357431
1453030_at	Msl2−pending	0.0004006	6.3862575
1453056_at	1700007D07Rik	0.0000977	7.6025977
1453077_a_at	5031401C21Rik	0.0000430	6.9188395
1453084_s_at	2310067L16Rik	0.0011015	3.0923070
1453099_at	Csnk2a2	0.0000430	9.3397656
1453102_at	5530600M07Rik	0.0010400	7.2030942
1453140_at	9030612M13Rik	0.0000493	8.0219300
1453145_at	4933439C20Rik	0.0000651	6.4183143
1453155_at	Smp1−pending	0.0018276	8.7292681
1453166_at	2010012O16Rik	0.0017295	7.0588787
1453167_at	1700013G24Rik	0.0031251	1.5722309
1453172_at	Stch	0.0000430	6.8263932
1453174_at	2310076G13Rik	0.0024934	7.1777242
1453201_at	4632411J06Rik	0.0009273	5.1443667
1453206_at	2600017P15Rik	0.0005118	6.7899679
1453208_at	2700089E24Rik	0.0019295	7.1607878
1453257_at	D8Ertd319e	0.0002767	8.3328700
1453263_at	C030004C14Rik	0.0020380	7.1641588
1453271_at	4932409F11Rik	0.0003132	8.4650992
1453282_at	Cxadr	0.0004013	7.8224393
1453283_at	Pgm1	0.0015476	6.4063503
1453285_at	2600017H02Rik	0.0000430	9.4806530
1453290_at	Hmgb2l1	0.0011663	6.9224106
1453299_a_at	Pnp	0.0001274	8.2096777
1453303_at	4833417J20Rik	0.0027892	1.7588050
1453304_s_at	Ly6e	0.0000921	8.7657552
1453311_at	2310008B10Rik	0.0012346	5.2675540
1453313_at	Sesn3	0.0000854	8.3757297
1453320_at	1700027A23Rik	0.0000854	5.2156186
1453360_a_at	Tex9	0.0004463	6.7467910
1453421_at	AW550801	0.0021520	3.6970175
1453458_at	9430019C24Rik	0.0010842	4.2057780
1453473_a_at	Tctex1	0.0000430	10.5426576
1453488_at	4930458D05Rik	0.0018250	3.8696029
1453501_at	Hoxb1	0.0000854	8.0975344
1453509_at	0610005K03Rik	0.0009273	2.4527920
1453556_x_at	2410026K10Rik	0.0015476	7.7788202
1453589_a_at	2610005L07Rik	0.0000430	5.9625959
1453623_a_at	Rad23a	0.0021520	6.4477514
1453740_a_at	Ccnl2	0.0000495	8.4796256
1453753_at	2810047L02Rik	0.0002436	7.5590555
1453762_at	1810012I05Rik	0.0024953	7.1640859
1453840_at	Pabpc1	0.0003134	7.0588413
1453848_s_at	2610005H11Rik	0.0031311	7.0524418
1454136_a_at	4921524J17Rik	0.0004263	7.3846995
1454186_a_at	2310051N18Rik	0.0000430	6.5567069
1454213_at	4930438O05Rik	0.0019295	3.5397179
1454599_at	4930425F17Rik	0.0015567	5.0657766
1454608_x_at	Ttr	0.0001317	11.7268286
1454609_x_at	6430527G18Rik	0.0013794	5.9388960
1454626_at	Cltc	0.0016374	10.9207437
1454664_a_at	Eif5	0.0014624	10.6220708
1454677_at	Timp2	0.0032957	6.1893928
1454679_at	D8Ertd457e	0.0000430	8.3814891
1454680_at	9030221A05Rik	0.0012337	7.5395401
1454686_at	6430706D22Rik	0.0002593	7.6997084
1454698_at	Hspc121−pending	0.0001819	8.2294011
1454700_at	MGC36545	0.0031291	6.4081266
1454701_at	4930503L19Rik	0.0010385	6.6310183
1454732_at	6430517J16Rik	0.0000430	8.1264871
1454749_at	Pcnt2	0.0026326	8.0956295
1454750_a_at	6720484B16	0.0000567	7.8986975
1454752_at	AI606861	0.0008749	7.1487448
1454755_at	9130023N17Rik	0.0031291	5.4518732
1454757_s_at	D12Ertd647e	0.0013066	2.3392889
1454764_s_at	Slc38a1	0.0006523	9.0292470
1454778_x_at	Rps28	0.0022750	12.5400539
1454807_a_at	Snx12	0.0031311	7.7467600
1454813_at	1110017O22Rik	0.0007343	11.7815417
1454815_at	AB041541	0.0014581	7.1898327
1454818_at	Gmeb2	0.0012355	6.4485097
1454838_s_at	AW548124	0.0003918	8.4963200
1454841_at	4921511H13Rik	0.0025269	7.5841962
1454843_at	Prps2	0.0025269	8.2736828
1454860_x_at	Dad1	0.0002436	9.0334881
1454866_s_at	5730466J16Rik	0.0005127	8.6988340
1454872_at	B230308N11Rik	0.0003003	8.6239984
1454875_a_at	Rbbp4	0.0010861	9.7292663
1454888_at	Pfdn4	0.0009280	9.4069814
1454890_at	Amot	0.0000568	10.4480060
1454891_at	Cds2	0.0013066	7.2378315
1454899_at	Lpp	0.0016374	6.8308851
1454904_at		0.0001277	11.9194275

1454905_at	5430411K16Rik	0.0031291	7.6883567
1454906_at	Rarb	0.0019242	5.2053465
1454909_at	B230378H13Rik	0.0007469	7.3378224
1454933_at	2610027C15Rik	0.0011672	6.9435864
1454957_at	Nob1p−pending	0.0001118	8.6730484
1454964_at	Lt1	0.0004020	8.7977812
1454974_at	Ntn1	0.0000430	7.3097570
1454980_at	4930402E16Rik	0.0001649	8.2147813
1454982_at	AW493672	0.0022163	7.1071604
1454996_at	2700067E09Rik	0.0014645	7.5317753
1455015_at	4933431N12Rik	0.0032916	3.1264985
1455035_s_at	Nol5a	0.0005783	10.2068407
1455036_s_at	1810004I06Rik	0.0025335	10.6237887
1455039_a_at	Sin3b	0.0000430	8.7528374
1455047_at	Fbxo3	0.0031282	7.1468737
1455054_a_at	Tes3−ps	0.0001192	7.4786754
1455057_at	Gmps	0.0034746	7.5043424
1455060_at	C87777	0.0013085	7.5100130
1455069_x_at	Slc25a4	0.0000430	10.6425665
1455070_at	AL118268	0.0002767	8.7880287
1455092_at	Zfp207	0.0009818	7.1710931
1455096_at	Flrt2	0.0003003	5.2601117
1455099_at	Mgat1l−pending	0.0022750	9.7228135
1455101_at		0.0014581	3.6246897
1455107_at	AU017960	0.0005094	9.1238594
1455127_at	5430438H03Rik	0.0013085	7.3914446
1455145_at		0.0000494	8.0727838
1455149_at	Posh−pending	0.0015476	6.4176922
1455157_a_at	2310061F22Rik	0.0034768	8.0522021
1455173_at	Gspt1	0.0000799	7.8330114
1455179_at	1110068J02Rik	0.0025286	5.4863015
1455195_at	Rps24	0.0005131	6.9664291
1455201_x_at	Apoa1	0.0010416	10.8209715
1455211_a_at	Timm9	0.0000493	9.8417744
1455220_at	MGC37615	0.0028043	5.8453933
1455245_x_at	Rpl13	0.0001118	12.7812891
1455246_at	Smarcc1	0.0016350	9.3669328
1455283_x_at	Ndufs8	0.0001453	9.6352472
1455287_at	5830411I20	0.0028151	7.1174804
1455320_at	AI480535	0.0002147	7.9585892
1455341_at	2010003J03Rik	0.0008469	8.0075758
1455357_x_at	Tomm20−pending	0.0000430	11.2684931
1455359_at	C130080N23Rik	0.0011672	7.9484848
1455390_at	C130099A02	0.0000430	7.3920349
1455391_at	Rad23a	0.0001277	8.4727537
1455401_at	Camkk2	0.0023963	5.8425227
1455402_at	Nap4−pending	0.0010393	7.1904078
1455420_at	Rad23b	0.0006517	6.4862243
1455425_at		0.0014645	8.0617342
1455427_at	Agpt4	0.0034789	2.6554762
1455476_a_at	mKIAA0182	0.0011663	6.6508309
1455494_at	Col1a1	0.0007337	5.6773746
1455545_at	1110065P20Rik	0.0011654	7.2148738
1455547_at	Scrg3	0.0001313	7.4225799
1455556_at	Notch2	0.0000855	8.8134356
1455558_at	−−−	0.0002762	6.1026834
1455563_at	−−−	0.0018276	8.0166615
1455572_x_at	Rps18	0.0002147	12.7161248
1455588_at	BC034664	0.0017295	6.1999085
1455593_at	Apob	0.0000429	9.0254266
1455604_at	AI427138	0.0000494	7.6911839
1455606_at		0.0008259	7.4483904
1455607_at	Thsd2	0.0000430	7.2670805
1455651_at	Terf2	0.0011663	7.3703597
1455668_at	6720429E03	0.0010400	6.4313693
1455713_x_at	Bcap37	0.0009280	10.4542057
1455726_at	−−−	0.0003773	7.2299523
1455735_at	Ap1s3	0.0000651	7.4576661
1455760_at	−−−	0.0014645	6.3171562
1455773_at		0.0021535	5.0827647
1455792_x_at	Ndn	0.0034746	8.2348956
1455794_at	D130058I21Rik	0.0034768	7.6857342
1455812_x_at	Slitl2	0.0006125	7.1437354
1455814_x_at	Ddx39	0.0000430	10.7664931
1455826_a_at	Bace	0.0000429	6.3943975
1455852_at		0.0034682	6.0245241
1455860_at	D630024D12Rik	0.0007343	6.2518799
1455871_s_at	1300011C24Rik	0.0010416	8.7861046
1455878_at	2700023E23Rik	0.0000494	7.6111796
1455892_x_at		0.0000799	6.8013951
1455893_at	2610028F08Rik	0.0029551	2.0032233
1455896_a_at	Kcnk1	0.0001118	6.7237467
1455904_at	Gas5	0.0016374	9.5922124
1455908_a_at	Risc−pending	0.0025319	9.3006992
1455913_x_at	Ttr	0.0000747	10.7775016
1455936_a_at	Rbpms	0.0000651	8.2433365
1455942_at	Fbxl11	0.0023433	8.3165322

1455943_at	AI596398	0.0032977	6.4565832
1455987_at	Sec61a	0.0009558	7.3788797
1455988_a_at	Cct6a	0.0004020	11.5296871
1455993_at	Odz4	0.0031311	6.0736312
1455997_a_at	Uqcrb	0.0002439	12.1125761
1456012_x_at	0610007O07Rik	0.0000742	8.1773305
1456022_at	B230339E18Rik	0.0008462	6.5043811
1456032_x_at	H2afz	0.0018289	10.4800336
1456040_at	Sf3b2	0.0002765	9.3754964
1456055_x_at	Pold1	0.0007343	8.4672946
1456070_at	5430405N12Rik	0.0017575	5.6817840
1456085_x_at	Cd151	0.0006135	7.9216200
1456097_a_at	4930471O16Rik	0.0001116	6.7564661
1456124_x_at	Svs5	0.0022719	3.5594629
1456155_x_at	Fuca	0.0004821	5.5724655
1456159_at		0.0000492	7.2244884
1456170_x_at	Calr	0.0003553	11.4786476
1456180_at	−−−	0.0013794	5.5043616
1456195_x_at	Itgb5	0.0025286	7.1721099
1456201_at	4632427E13Rik	0.0018613	6.9221017
1456256_at	Eif5	0.0000429	8.4610261
1456276_at	2300009A05Rik	0.0025302	3.6655620
1456279_a_at	Bcap31	0.0005131	8.6736379
1456292_a_at	Vim	0.0000430	9.0805125
1456296_at	5832426L23Rik	0.0024896	6.0782553
1456319_at	X83313	0.0000430	8.0064542
1456329_at	A230098A12Rik	0.0000495	10.7451403
1456338_at	9130023D20Rik	0.0006464	6.9115528
1456369_at	Pot1	0.0027971	2.0909635
1456390_at	R75353	0.0001862	8.4274556
1456393_at	Pdcd4	0.0011681	8.8924685
1456399_at	Bace	0.0001274	6.4329541
1456413_at	9430063L05Rik	0.0023915	5.3432715
1456422_at		0.0032916	5.3725823
1456424_s_at	Pltp	0.0002434	7.0451942
1456447_at	Rpl18	0.0031330	5.8160193
1456449_at		0.0000528	6.6158410
1456466_x_at	Sca10	0.0002012	8.5367199
1456482_at	Pik3r3	0.0020380	7.0163218
1456492_at	A930019L04Rik	0.0004002	6.9715838
1456497_x_at	2210402A09Rik	0.0001657	12.6576346
1456501_at	2610528E23Rik	0.0000850	4.9093478
1456511_x_at	Eras	0.0000853	7.6129829
1456515_s_at	Figlb	0.0020394	8.1474627
1456541_x_at	2400004H09Rik	0.0008259	7.9123157
1456550_at	Bcl2l12	0.0012231	5.1177467
1456553_at		0.0009549	5.7491558
1456565_s_at	Map3k12	0.0001652	5.1788550
1456580_s_at	0610008F14Rik	0.0000652	10.4722724
1456597_at	C030036P15Rik	0.0000651	8.2317913
1456604_a_at	Pcmt1	0.0000430	7.7614682
1456610_at	BC038313	0.0014634	8.0307621
1456616_a_at	Bsg	0.0000652	11.0435061
1456633_at	6330504P12Rik	0.0008423	1.7169272
1456642_x_at	S100a10	0.0010408	8.1708952
1456655_at		0.0000430	7.8215930
1456659_at		0.0002757	5.9822424
1456661_at		0.0029904	7.2562573
1456663_x_at	Blp1−pending	0.0022750	5.9207718
1456728_x_at	Aco1	0.0005127	9.5974385
1456730_x_at	Baf53a−pending	0.0019308	9.8404521
1456733_x_at	Serpinh1	0.0002293	9.8141717
1456743_x_at	Morf4l2	0.0002439	11.6606335
1456752_at		0.0006517	8.2660570
1456753_at	Mllt7	0.0000977	8.4776094
1456783_at	9330107J05Rik	0.0000430	7.8825046
1456795_at	D330027G24Rik	0.0028188	8.8814641
1456808_at		0.0034725	5.8500881
1456865_x_at	Rrs1	0.0016374	9.6249954
1456898_at	Pura	0.0001879	6.1991636
1456901_at		0.0018263	5.3430658
1456960_at		0.0008313	5.8332745
1456970_at		0.0022704	3.9662549
1457047_at		0.0000977	6.1828897
1457058_at	Adamts2	0.0025269	4.9312852
1457073_at		0.0011672	6.9548105
1457078_at	D030014N22Rik	0.0003137	6.4025181
1457079_at		0.0020667	5.6652961
1457141_at		0.0025302	5.9511611
1457302_at		0.0003553	6.8181188
1457314_at	−−−	0.0002010	7.9280848
1457384_at		0.0026588	4.6919510
1457417_at		0.0031192	3.5911625
1457424_at	Eya1	0.0014687	5.3984736
1457458_at	mKIAA1064	0.0000430	5.0301834
1457489_at		0.0004013	7.3186543
1457503_at		0.0003550	5.4581819

1457508_at	C430003N24Rik	0.0000495	6.5323503
1457528_at	Slc4a7	0.0028170	7.1908952
1457573_at		0.0018162	5.8758576
1457579_at	D11Ertd717e	0.0006523	6.0153365
1457582_at	Uty	0.0003992	4.5528965
1457588_at	C76213	0.0003137	5.7778340
1457632_s_at	Mrg1	0.0016374	8.4348066
1457672_at	1810014J18Rik	0.0004002	5.7068021
1457679_at	Suv39h2	0.0002936	1.8326449
1457697_at	A330021E22Rik	0.0023947	0.2412224
1457712_at	AW549269	0.0008735	6.6146023
1457723_at		0.0028170	7.2632393
1457739_at	Ilf3	0.0001551	6.1055862
1457749_at	A830039B04Rik	0.0035043	5.1937447
1457760_at	A930004J17Rik	0.0014624	6.2906769
1457780_at		0.0031147	4.4923717
1457809_at	Pcaf	0.0016456	4.5591563
1457823_at	Cyr61	0.0002762	5.4928235
1457844_a_at	4931410H15	0.0029478	3.1484790
1457848_at		0.0009244	4.3244601
1457913_at		0.0004013	8.0008177
1457936_at	Mapk8	0.0006517	5.7999242
1457945_at		0.0005778	5.9637119
1457962_at		0.0015567	4.0853518
1458052_at		0.0022598	3.1952938
1458053_at	C130078H13	0.0010408	4.9051907
1458056_at	AI450757	0.0013085	6.6311538
1458065_at		0.0025252	6.2209869
1458069_at		0.0013057	3.8446047
1458073_at	9330156P08Rik	0.0020338	3.8440957
1458094_at		0.0026692	5.2527679
1458179_at		0.0013066	5.7807528
1458218_s_at	Pde7a	0.0032977	8.1967820
1458232_at	Dkk1	0.0023963	4.7713003
1458347_s_at	Tmprss2	0.0005778	9.1988669
1458351_s_at	Klhl2	0.0006512	6.3221326
1458375_at	−−−	0.0026640	3.7372552
1458461_at	E330021D16Rik	0.0029543	2.2482253
1458480_at	2810429C13Rik	0.0016362	5.1852752
1458502_at		0.0029645	5.9373373
1458508_at	Matr3	0.0000430	7.8523118
1458541_at	Dctn4	0.0006501	4.0449106
1458549_at		0.0001886	6.3045963
1458618_at		0.0032998	5.3301128
1458626_at	Nos1	0.0013037	3.2365059
1458632_at		0.0013037	4.0608189
1458669_at		0.0028185	5.4668064
1458676_at	5330401F18Rik	0.0018174	3.5336983
1458687_at	−−−	0.0002434	5.7223648
1458701_at	2310032D16Rik	0.0014613	1.4229390
1458800_at		0.0025286	3.9082985
1458863_at	6330415G19Rik	0.0005757	3.8724593
1458876_at		0.0026467	6.1130132
1458910_at		0.0005778	5.7145593
1458980_at		0.0022719	5.5895128
1459093_at		0.0001361	1.6018692
1459253_at		0.0000567	6.9527179
1459445_at		0.0017518	3.5174856
1459487_at		0.0003132	3.4923513
1459488_at		0.0031311	5.7158911
1459593_x_at	−−−	0.0017575	7.4289165
1459609_at		0.0009567	3.9057736
1459657_s_at	Rpo1−3	0.0000568	9.2199500
1459670_at		0.0002423	5.0368450
1459718_x_at		0.0014565	4.1278210
1459720_x_at	Tipin−pending	0.0020366	4.8616436
1459737_s_at	Ttr	0.0000495	11.0101876
1459806_x_at	Mrps23	0.0009265	6.5265439
1459827_x_at	Hps1	0.0019308	6.6203400
1459832_s_at	Ap1m1	0.0016362	8.2021881
1459843_s_at	Madh1	0.0000495	7.7611168
1459987_s_at	Cct3	0.0031330	10.9374837
1460017_at		0.0008322	6.1959971
1460032_at		0.0003330	6.4302980
1460035_at	Bcap37	0.0000795	6.2405368
1460037_at	2610510H03Rik	0.0002439	9.3793219
1460063_at	D5Ertd798e	0.0001192	5.9249971
1460094_at	AI790205	0.0007776	2.3526921
1460095_at		0.0034703	1.9047102
1460102_at	Clasp1	0.0007776	7.1157926
1460116_s_at	Spred1	0.0017282	5.8422917
1460159_at	C130067A03Rik	0.0001118	2.7416438
1460168_at	Slbp	0.0003137	10.2357639
1460178_at	1300002A08Rik	0.0004528	6.9127900
1460187_at	Sfrp1	0.0006517	7.5100032
1460188_at	Hcph	0.0021491	5.8536231
1460192_at	Osbpl1a	0.0006491	6.9956808

1460195_at	Mrps11	0.0006523	8.4185568
1460198_a_at	Psmb3	0.0000430	9.9369167
1460224_at	Snx2	0.0002143	8.5682059
1460247_a_at	Skp2	0.0000651	8.8827377
1460339_at	Psma4	0.0000652	10.9155224
1460346_at	Arsa	0.0003547	8.1616523
1460351_at	S100a11	0.0002944	7.6612390
1460369_at	LOC233987	0.0008194	5.4086149
1460379_at	Hoxb4	0.0005446	5.8324980
1460397_at	2410153K17Rik	0.0001044	7.2178334
1460401_at	2310050N11Rik	0.0001742	8.2726941
1460446_at	5530402J05Rik	0.0000747	8.1265579
1460453_at	2610315E15Rik	0.0012262	6.4892650
1460456_at	2010316F05Rik	0.0000977	7.4244128
1460468_s_at	2810451A06Rik	0.0008673	6.1569336
1460506_s_at	1810004I06Rik	0.0010408	9.7240497
1460541_at	AI643885	0.0001275	8.5086243
1460547_a_at	Hnrpk	0.0002439	11.7032097
1460548_a_at	Eral1	0.0029702	7.6096779
1460551_at	Ran	0.0010416	8.9865411
1460554_s_at	Glg1	0.0009280	9.2064268
1460567_at	9930116O05Rik	0.0000651	7.4010183
1460575_at	D3Ertd194e	0.0011681	7.9767566
1460581_a_at	Rpl13	0.0000430	12.9738773
1460602_at	Dlc1	0.0020408	6.8285379
1460629_at	Trim16	0.0013028	3.1832806
1460637_s_at	Pfdn5	0.0006523	8.9352493
1460646_at	Csnk2a2	0.0024953	8.3978885
1460664_at	Znfn1a4	0.0023963	1.9020614
1460665_a_at	Cnot7	0.0000495	7.3239397
1460668_at	Gal	0.0000567	6.7182450
1460670_at	Riok3	0.0004825	7.0287785
1460701_a_at	Mrpl52	0.0005131	9.1461306
1460712_s_at	Ap1g1	0.0004821	8.1663131
1460716_a_at	Cbfb	0.0001454	9.6501175
1460722_at	Soat2	0.0016362	7.6462423
1460739_at	D11Bwg0280e	0.0025302	7.2807763
AFFX-BioB-M_at		0.0019016	8.1672903
AFFX-MURINE_b1_at	Bc1	0.0000001	11.6525720
AFFX-b-ActinMur/M12481_5_at	Actb	0.0000733	12.4983903
AFFX-b-ActinMur/M12481_M_at	Actb	0.0003408	12.5204773
AFFX-r2-P1-cre-5_at		0.0010416	12.9942370

Table 5: Probesets significant at the 5%-level with Bejnamini-Hochberg FDR control

The gene symbols for the intersection list and the genes only detected with the MOE4302 are generated and may be requested (as tab-delimited files) separately.

3.4 Testing on the combined dataset (probesets)

We use the dataset produced as described in the "Mapping" section and normalized with generalized additive models (GAM, (Chambers, 1992)) as normalization algorithm.

We apply the following FDR corrections:

- None (raw p-values)
- Bonferroni (Bonf)
- Benjamini-Hochberg (BH)
- Benjamini-Yekutieli (BY)

Neither the Welch's two-sample t-test nor the Bayesian regularization by (Baldi and Long, 2001) detects significant differences for any of the probesets.

4 Mapping MG-U74 data on MOE4302 data

All probesets of the MOE430 chip group were completely designed from scratch. Hence, probesets generated from the same UniGene cluster (i.e. putatively measuring the mRNA of the same gene) are not necessarily comparable. Simply mapping probesets according to the best match of the used template leads to a dataset which cannot be normalized properly (fig. 13).

To select valid probeset pairs, we omit pairs which do not reproduce. In detail, we have four extracts hybridized once on the MG-U74 and once on the MOE4302 arrays. If the expression intensity is consistently higher (or lower) in at least three out of the four chip pairs the probeset is excluded from further analysis. The threshold on the logarithmic fold-change is varied and takes values shown in tab. 6 preserving different numbers of probesets. Thresholding to a maximum logarithmic fold-change of 3 between replicates seems to be a fair trade-off between chances to fit reasonable normalization parameters and information loss.

Threshold	No. remaining probesets (MAS5.0)	(Li-Wong)
2	8737	7838
3	9478	8645
4	9827	9119
None	10038	10038

Table 6: Threshold and numbers of preserved probesets

5 Conclusions

The quality of the new hybridizations is much more promising. However, we were unable to gain additional benefits from combining the two chip platforms on the raw data level, i.e. before the deduction of the signicantly differential expression.

> In conclusion, the application of statistics to the life sciences is likely to become more fruitful if greater emphasis is placed on the articulation of global conjectures by the specialist most intimatetly involved; if statistics is used to deduce probability statements from such conjectures; if emphasis is placed not on ”validating” the probability statement involved but on FALSIFYING it; and if the inductivist attitude of the profession is softened. We badly need a brand of applied statistics which in temperament is inspirationist, global, deductivist, and falsificationist rather than empiricist, local, inductivist, and verificationist in spirit. Hypotheses are like nets, only he who casts can catch.

((Dolby, 1982))

. . . **and, of course, keep (Tilstone, 2003) or (Gibson, 2003) in mind!**

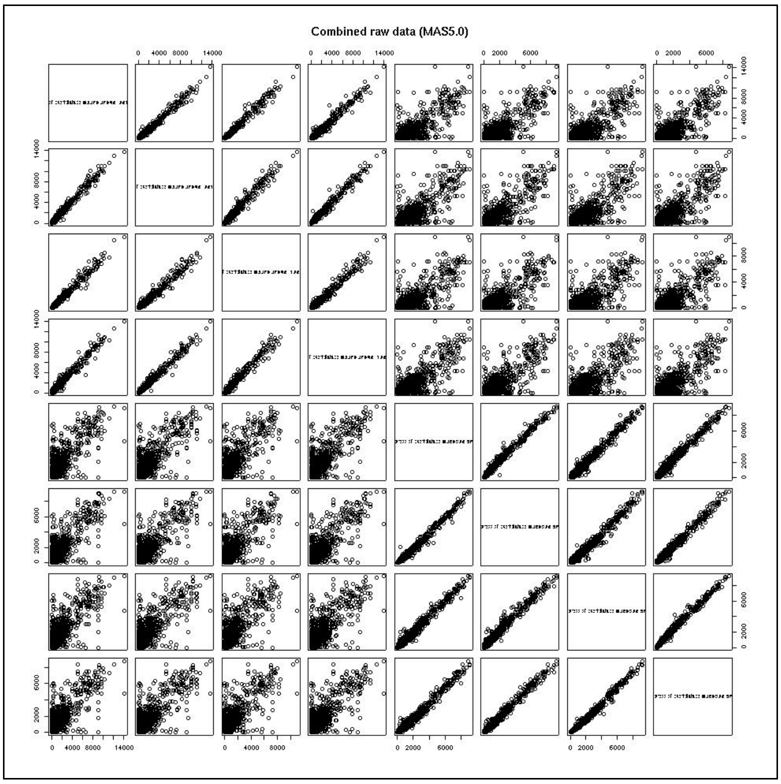

Figure 13: Scatterplot matrix of the raw combined data without filtering

References

Baldi, P. and Long, A. D. (2001). A bayesian framework for the analysis of microarray expression data: regularized t-test and statistical inference of gene changes. *Bioinformatics*, 17(6):509–519.

Benjamini, Y. and Hochberg, Y. (1997). Multiple hypotheses testing with weights. *Scand. J. Stat.*, 24:407–418.

Bolstad, B. M., Irizarry, R. A., Astrand, M., and Speed, T. P. (2003). A comparison of normalization methods for high density oligonucleotide array data based on variance and bias. *Bioinformatics*, 19(2):185–193.

Chambers, J. M. (1992). *Statistical Models in S*, chapter 4. Linear models. Wadsworth & Brooks/Cole.

Cleveland, W. S. (1979). Robust locally weighted regression and smoothing scatterplots. *J. Amer. Statist. Assoc.*, 74:829–36.

Cleveland, W. S. (1981). Lowess: A program for smoothing scatterplots by robust locally weighted regression. *The American Statistician*, 35:54.

Cleveland, W. S., Grosse, E., and Shyu, W. M. (1992). *Statistical Models in S*, chapter Local regression models. Chapter 8. Wadsworth & Brooks/Cole.

Dolby, G. R. (1982). The role of statistics in the methodology of the life sciences. *Biometrics*, 38:1069–1083.

Gibson, G. (2003). Microarray analysis. *PLoS*, 1(1):028–029.

Ihaka, R. and Gentleman, R. (1996). R: A language for data analysis and graphics. *Journal of Computational and Graphical Statistics*, 5(3):299–314.

Li, C. and Wong, W. H. (2001). Model-based analysis of oligonucleotide arrays: Expression index computation and outlier detection. *PNAS*, 98(1):31–36.

Tilstone, C. (2003). Vital statistics. *Nature*, 424:610–612.
