# Supplementary material for: Microarray analysis of Foxa2 mutant mouse embryos reveals novel gene expression and inductive roles for the gastrula organizer and its derivatives
Source: BMC Genomics. 2008 Oct 30;9:511. doi: 10.1186/1471-2164-9-511 (PMC2605479; doi:10.1186/1471-2164-9-511)
Supplement: Additional file 7 — Supplementary Table 4. Gene Ontology (GO) terms significantly enriched (p ≤ 0.01) among genes expressed in the primary tissues affected in Foxa2 mutants. [file 1471-2164-9-511-S7.pdf]

Gene Ontology (GO) terms significantly enriched (p≤0.01) among genes expressed in the primary tissues affected in Foxa2 mutants.

**GO Term**	**Specific Term**	**GO ID**	**P value (Average)**	**E value**	**Gene Hits**
Molecular function	thyrotropin-releasing hormone activity	GO:0008437	0.001269	788.16	1
Molecular function	N-acetyllactosaminide 3-alpha-galactosyltransferase activity	GO:0047276	0.001269	788.16	1
Molecular function	sequence-specific DNA binding	GO:0043565	0.00199	7.18	4
Molecular function	transcription factor activity	GO:0003700	0.002335	5.08	5
Molecular function	ferric-chelate reductase activity	GO:0000293	0.003802	262.72	1
Molecular function	oxidoreductase activity, oxidizing metal ions, NAD or NADP as acceptor	GO:0016723	0.005066	197.04	1
Molecular function	hormone activity	GO:0005179	0.007563	15.16	2
Molecular function	ceramidase activity	GO:0017040	0.00759	131.36	1
Molecular function	oxidoreductase activity, oxidizing metal ions	GO:0016722	0.00885	112.59	1
Biological process	anterior/posterior pattern formation	GO:0009952	0.000362	21.11	3
Biological process	morphogenesis of an epithelium	GO:0002009	0.000432	19.87	3
Biological process	tube morphogenesis	GO:0035239	0.000652	17.26	3
Biological process	vasculogenesis	GO:0001570	0.000694	50.85	2
Biological process	regionalization	GO:0003002	0.000986	14.97	3
Biological process	epithelial cell differentiation	GO:0030855	0.001335	36.66	2
Biological process	tube development	GO:0035295	0.001754	12.25	3
Biological process	myeloid progenitor cell differentiation	GO:0002318	0.002536	394.08	1
Biological process	ectoderm formation	GO:0001705	0.002536	394.08	1
Biological process	branching morphogenesis of a tube	GO:0048754	0.002584	26.27	2
Biological process	pattern specification process	GO:0007389	0.002925	10.24	3
Biological process	gastrulation	GO:0007369	0.003025	24.25	2
Biological process	positive regulation of transcription from RNA polymerase II promoter	GO:0045944	0.003182	9.93	3
Biological process	morphogenesis of a branching structure	GO:0001763	0.00321	23.53	2
Biological process	lung development	GO:0030324	0.003305	23.18	2
Biological process	respiratory tube development	GO:0030323	0.003401	22.85	2
Biological process	positive regulation of transcription, DNA-dependent	GO:0045893	0.004774	8.6	3
Biological process	positive regulation of RNA metabolic process	GO:0051254	0.004774	8.6	3
Biological process	anatomical structure development	GO:0048856	0.004983	3.05	7
Biological process	ceramide catabolic process	GO:0046514	0.005066	197.04	1
Biological process	sphingoid catabolic process	GO:0046521	0.005066	197.04	1
Biological process	notochord development	GO:0030903	0.006329	157.63	1

Gene Ontology (GO) terms significantly enriched (p≤0.01) among genes expressed in the primary tissues affected in Foxa2 mutants.

**GO Term**	**Specific Term**	**GO ID**	**P value (Average)**	**E value**	**Gene Hits**
Biological process	prostate gland development	GO:0030850	0.006329	157.63	1
Biological process	positive regulation of transcription	GO:0045941	0.00708	7.46	3
Biological process	epithelial cell maturation	GO:0002070	0.00759	131.36	1
Biological process	anatomical structure morphogenesis	GO:0009653	0.007648	3.85	5
Biological process	positive regulation of nucleobase, nucleoside, nucleotide and nucleic acid metabolic process	GO:0045935	0.007908	7.17	3
Biological process	positive regulation of neuron differentiation	GO:0045666	0.00885	112.59	1
Cellular component	anchored to membrane	GO:0031225	0.009027	13.83	2
Cellular component	transcription factor complex	GO:0005667	0.013698	5.85	3
Cellular component	nucleoplasm part	GO:0044451	0.025484	4.63	3
Cellular component	nucleoplasm	GO:0005654	0.028975	4.4	3
Cellular component	extracellular region part	GO:0044421	0.034232	2.34	6
Cellular component	Golgi apparatus	GO:0005794	0.035134	4.08	3
Cellular component	nuclear lumen	GO:0031981	0.046815	3.64	3
Cellular component	membrane raft	GO:0045121	0.050788	19.22	1
Cellular component	cellular_component	GO:0005575	0.061503	1.16	19
Cellular component	membrane-enclosed lumen	GO:0031974	0.062899	3.23	3
Cellular component	organelle lumen	GO:0043233	0.062899	3.23	3
Cellular component	basement membrane	GO:0005604	0.071121	13.59	1
Cellular component	extracellular region	GO:0005576	0.072875	1.95	6
Cellular component	extracellular space	GO:0005615	0.085216	2.06	5
Cellular component	tight junction	GO:0005923	0.086391	11.1	1
Cellular component	Golgi membrane	GO:0000139	0.088719	10.8	1
Cellular component	endoplasmic reticulum membrane	GO:0005789	0.088719	10.8	1
Cellular component	nuclear envelope-endoplasmic reticulum network	GO:0042175	0.093358	10.24	1
