# Supplementary material for: Microarray analysis of Foxa2 mutant mouse embryos reveals novel gene expression and inductive roles for the gastrula organizer and its derivatives
Source: BMC Genomics. 2008 Oct 30;9:511. doi: 10.1186/1471-2164-9-511 (PMC2605479; doi:10.1186/1471-2164-9-511)
Supplement: Additional file 8 — Supplementary Table 5. Gene Ontology (GO) terms significantly enriched (p ≤ 0.01) among genes expressed in the secondary tissues affected in Foxa2 mutants. [file 1471-2164-9-511-S8.pdf]

Gene Ontology (GO) terms significantly enriched (p≤0.01) among genes expressed in the secondary tissues affected in Foxa2 mutants.

**GO Term**	**Specific Term**	**GO ID**	**P value (Average)**	**E value**	**Gene Hits**
Molecular function	sequence-specific DNA binding	GO:0043565	0.000003	7.14	9
Molecular function	retinal dehydrogenase activity	GO:0001758	0.000048	174.13	2
Molecular function	transcription factor activity	GO:0003700	0.000053	4.49	10
Molecular function	3-chloroallyl aldehyde dehydrogenase activity	GO:0004028	0.00012	116.09	2
Molecular function	transcription regulator activity	GO:0030528	0.0009	3.17	10
Molecular function	RNA polymerase II transcription factor activity, enhancer binding	GO:0003705	0.001961	30.28	2
Molecular function	oxidoreductase activity, acting on the aldehyde or oxo group of donors, NAD or NADP as acceptor	GO:0016620	0.002505	26.79	2
Molecular function	oxidoreductase activity, acting on the aldehyde or oxo group of donors	GO:0016903	0.004513	19.9	2
Molecular function	molecular_function	GO:0003674	0.004525	1.17	42
Molecular function	NF-kappaB-inducing kinase activity	GO:0004704	0.005735	174.13	1
Molecular function	motor activity	GO:0003774	0.005741	8.23	3
Molecular function	arginase activity	GO:0004053	0.00859	116.09	1
Molecular function	growth factor binding	GO:0019838	0.009041	13.93	2
Molecular function	protein-tyrosine kinase activity	GO:0004713	0.009572	6.83	3
Biological process	pattern specification process	GO:0007389	0	12.06	8
Biological process	developmental process	GO:0032502	0	3.1	24
Biological process	multicellular organismal process	GO:0032501	0	3.16	24
Biological process	regionalization	GO:0003002	0	15.43	7
Biological process	multicellular organismal development	GO:0007275	0	3.84	23
Biological process	anterior/posterior pattern formation	GO:0009952	0	21.77	7
Biological process	system development	GO:0048731	0.000001	3.66	17
Biological process	nervous system development	GO:0007399	0.000001	6.04	11
Biological process	regulation of biological process	GO:0050789	0.000001	2.47	25
Biological process	sensory organ development	GO:0007423	0.000001	13.7	7
Biological process	organ development	GO:0048513	0.000002	3.93	15
Biological process	brain development	GO:0007420	0.000002	12.13	7
Biological process	anatomical structure development	GO:0048856	0.000004	3.27	17
Biological process	central nervous system development	GO:0007417	0.000007	9.64	7
Biological process	cellular process	GO:0009987	0.000008	1.47	40

Gene Ontology (GO) terms significantly enriched (p≤0.01) among genes expressed in the secondary tissues affected in Foxa2 mutants.

GO Term	Specific Term	GO ID	P value (Average)	E value	Gene Hits
Biological process	biological regulation	GO:0065007	0.000008	2.24	25
Biological process	inner ear morphogenesis	GO:0042472	0.00001	29.64	4
Biological process	organ morphogenesis	GO:0009887	0.000011	6.16	9
Biological process	ear morphogenesis	GO:0042471	0.000017	25.8	4
Biological process	embryonic morphogenesis	GO:0048598	0.000019	10.66	6
Biological process	anatomical structure morphogenesis	GO:0009653	0.00002	4.09	12
Biological process	actomyosin structure organization and biogenesis	GO:0031032	0.000021	54.99	3
Biological process	somite specification	GO:0001757	0.000024	232.17	2
Biological process	inner ear development	GO:0048839	0.000029	22.47	4
Biological process	embryonic development	GO:0009790	0.00004	6.1	8
Biological process	ear development	GO:0043583	0.000047	19.9	4
Biological process	neurogenesis	GO:0022008	0.000073	6.68	7
Biological process	regulation of cellular process	GO:0050794	0.000083	2.24	21
Biological process	somitogenesis	GO:0001756	0.000103	32.65	3
Biological process	determination of symmetry	GO:0009799	0.000113	31.66	3
Biological process	determination of bilateral symmetry	GO:0009855	0.000113	31.66	3
Biological process	cardiac muscle morphogensis	GO:0055008	0.00012	116.09	2
Biological process	neuron differentiation	GO:0030182	0.000201	6.97	6
Biological process	segmentation	GO:0035282	0.000202	26.12	3
Biological process	cell differentiation	GO:0030154	0.000212	2.82	14
Biological process	cellular developmental process	GO:0048869	0.000212	2.82	14
Biological process	segment specification	GO:0007379	0.000223	87.06	2
Biological process	muscle morphogenesis	GO:0048644	0.000223	87.06	2
Biological process	embryonic pattern specification	GO:0009880	0.000234	24.88	3
Biological process	hindbrain development	GO:0030902	0.000251	24.3	3
Biological process	cytoskeleton organization and biogenesis	GO:0007010	0.000263	5.43	7
Biological process	cell development	GO:0048468	0.000337	3.29	11
Biological process	retinoic acid metabolic process	GO:0042573	0.000357	69.65	2
Biological process	forebrain development	GO:0030900	0.000392	11.51	4
Biological process	generation of neurons	GO:0048699	0.000412	6.09	6
Biological process	Wnt receptor signaling pathway	GO:0016055	0.000431	11.23	4
Biological process	biological_process	GO:0008150	0.00044	1.27	41

Gene Ontology (GO) terms significantly enriched (p≤0.01) among genes expressed in the secondary tissues affected in Foxa2 mutants.

**GO Term**	**Specific Term**	**GO ID**	**P value (Average)**	**E value**	**Gene Hits**
Biological process	neuron development	GO:0048666	0.000499	7.54	5
Biological process	striated muscle cell development	GO:0055002	0.000717	49.75	2
Biological process	myofibril assembly	GO:0030239	0.000717	49.75	2
Biological process	muscle cell development	GO:0055001	0.000826	46.43	2
Biological process	skeletal muscle fiber development	GO:0048741	0.00089	15.83	3
Biological process	muscle fiber development	GO:0048747	0.00089	15.83	3
Biological process	vitamin A metabolic process	GO:0006776	0.001066	40.97	2
Biological process	negative regulation of Wnt receptor signaling pathway	GO:0030178	0.001066	40.97	2
Biological process	camera-type eye development	GO:0043010	0.001146	14.51	3
Biological process	actin cytoskeleton organization and biogenesis	GO:0030036	0.0012	8.55	4
Biological process	cell maturation	GO:0048469	0.001289	13.93	3
Biological process	Wnt receptor signaling pathway, calcium modulating pathway	GO:0007223	0.001481	34.83	2
Biological process	hormone metabolic process	GO:0042445	0.001498	13.22	3
Biological process	muscle development	GO:0007517	0.001526	8.01	4
Biological process	actin filament-based process	GO:0030029	0.001526	8.01	4
Biological process	myoblast maturation	GO:0048628	0.001634	33.17	2
Biological process	myoblast development	GO:0048627	0.001794	31.66	2
Biological process	fat-soluble vitamin metabolic process	GO:0006775	0.001961	30.28	2
Biological process	regulation of Wnt receptor signaling pathway	GO:0030111	0.002317	27.86	2
Biological process	developmental maturation	GO:0021700	0.002318	11.36	3
Biological process	neurite development	GO:0031175	0.002724	6.83	4
Biological process	rhombomere 2 development	GO:0021568	0.002871	348.26	1
Biological process	muscle thin filament assembly	GO:0030240	0.002871	348.26	1
Biological process	atrial cardiac muscle morphogenesis	GO:0055009	0.002871	348.26	1
Biological process	rhombomere development	GO:0021546	0.002871	348.26	1
Biological process	Wnt receptor signaling pathway in forebrain neuroblast division	GO:0021874	0.002871	348.26	1
Biological process	midbrain-hindbrain boundary morphogenesis	GO:0021555	0.002871	348.26	1
Biological process	eye development	GO:0001654	0.003022	10.34	3
Biological process	anatomical structure formation	GO:0048646	0.003128	6.57	4
Biological process	skeletal muscle development	GO:0007519	0.003194	10.14	3

Gene Ontology (GO) terms significantly enriched (p≤0.01) among genes expressed in the secondary tissues affected in Foxa2 mutants.

**GO Term**	**Specific Term**	**GO ID**	**P value (Average)**	**E value**	**Gene Hits**
Biological process	striated muscle cell differentiation	GO:0051146	0.003329	23.22	2
Biological process	neural crest cell development	GO:0014032	0.003552	22.47	2
Biological process	neural crest cell differentiation	GO:0014033	0.003552	22.47	2
Biological process	mesoderm formation	GO:0001707	0.003782	21.77	2
Biological process	determination of left/right symmetry	GO:0007368	0.003782	21.77	2
Biological process	mesoderm morphogenesis	GO:0048332	0.004019	21.11	2
Biological process	regulation of heart contraction	GO:0008016	0.004513	19.9	2
Biological process	heart morphogenesis	GO:0003007	0.004513	19.9	2
Biological process	myoblast differentiation	GO:0045445	0.004513	19.9	2
Biological process	cell fate commitment	GO:0045165	0.004569	8.93	3
Biological process	formation of primary germ layer	GO:0001704	0.004769	19.35	2
Biological process	chordate embryonic development	GO:0043009	0.004794	5.83	4
Biological process	embryonic development ending in birth or egg hatching	GO:0009792	0.005083	5.73	4
Biological process	cellular metabolic process	GO:0044237	0.005158	1.51	26
Biological process	negative regulation of biological process	GO:0048519	0.005198	2.92	8
Biological process	mesenchymal cell development	GO:0014031	0.005579	17.86	2
Biological process	optic cup morphogenesis involved in camera-type eye development	GO:0002072	0.005735	174.13	1
Biological process	paraxial mesodermal cell differentiation	GO:0048342	0.005735	174.13	1
Biological process	negative regulation of mechanoreceptor differentiation	GO:0045632	0.005735	174.13	1
Biological process	neuroblast division	GO:0055057	0.005735	174.13	1
Biological process	paraxial mesodermal cell fate commitment	GO:0048343	0.005735	174.13	1
Biological process	forebrain neuroblast division	GO:0021873	0.005735	174.13	1
Biological process	skeletal myofibril assembly	GO:0014866	0.005735	174.13	1
Biological process	negative regulation of auditory receptor cell differentiation	GO:0045608	0.005735	174.13	1
Biological process	mesenchymal cell differentiation	GO:0048762	0.005862	17.41	2
Biological process	regulation of cellular metabolic process	GO:0031323	0.006101	2.09	13
Biological process	striated muscle development	GO:0014706	0.006255	7.98	3
Biological process	phosphorus metabolic process	GO:0006793	0.00729	3.04	7
Biological process	phosphate metabolic process	GO:0006796	0.00729	3.04	7

Gene Ontology (GO) terms significantly enriched (p≤0.01) among genes expressed in the secondary tissues affected in Foxa2 mutants.

**GO Term**	**Specific Term**	**GO ID**	**P value (Average)**	**E value**	**Gene Hits**
Biological process	telencephalon development	GO:0021537	0.007373	15.48	2
Biological process	heart process	GO:0003015	0.007373	15.48	2
Biological process	heart contraction	GO:0060047	0.007373	15.48	2
Biological process	regulation of metabolic process	GO:0019222	0.007811	2.03	13
Biological process	biopolymer metabolic process	GO:0043283	0.008134	1.68	19
Biological process	protein amino acid phosphorylation	GO:0006468	0.008379	3.35	6
Biological process	forebrain anterior/posterior pattern formation	GO:0021797	0.00859	116.09	1
Biological process	sequestering of actin monomers	GO:0042989	0.00859	116.09	1
Biological process	regulation of auditory receptor cell differentiation	GO:0045607	0.00859	116.09	1
Biological process	midbrain-hindbrain boundary development	GO:0030917	0.00859	116.09	1
Biological process	negative regulation of actin filament polymerization	GO:0030837	0.00859	116.09	1
Biological process	regulation of mechanoreceptor differentiation	GO:0045631	0.00859	116.09	1
Biological process	cell part morphogenesis	GO:0032990	0.008851	4.89	4
Biological process	cell projection morphogenesis	GO:0048858	0.008851	4.89	4
Biological process	cell projection organization and biogenesis	GO:0030030	0.008851	4.89	4
Biological process	mesoderm development	GO:0007498	0.009393	13.66	2
Biological process	cell migration	GO:0016477	0.009506	4.79	4
Cellular component	transcription factor complex	GO:0005667	0.000017	6.9	8
Cellular component	nucleoplasm part	GO:0044451	0.000088	5.45	8
Cellular component	nucleoplasm	GO:0005654	0.000124	5.19	8
Cellular component	nuclear lumen	GO:0031981	0.00045	4.29	8
Cellular component	membrane-enclosed lumen	GO:0031974	0.000993	3.81	8
Cellular component	organelle lumen	GO:0043233	0.000993	3.81	8
Cellular component	actin cytoskeleton	GO:0015629	0.002105	7.33	4
Cellular component	intracellular organelle part	GO:0044446	0.002931	2.18	14
Cellular component	organelle part	GO:0044422	0.003032	2.17	14
Cellular component	protein complex	GO:0043234	0.003337	2.49	11
Cellular component	nuclear part	GO:0044428	0.005636	2.88	8
Cellular component	neuron projection	GO:0043005	0.005995	8.1	3
Cellular component	intracellular part	GO:0044424	0.007702	1.35	32
