# Supplementary material for: Microarray analysis of Foxa2 mutant mouse embryos reveals novel gene expression and inductive roles for the gastrula organizer and its derivatives
Source: BMC Genomics. 2008 Oct 30;9:511. doi: 10.1186/1471-2164-9-511 (PMC2605479; doi:10.1186/1471-2164-9-511)
Supplement: Additional file 9 — Supplementary Table 6. oPOSSUM output: TF motifs identified in promoters of genes reduced in Foxa2 mutants and expressed in regions of Foxa2 activity. [file 1471-2164-9-511-S9.pdf]

oPOSSUM output: TF motifs identified in promoters of genes reduced in Foxa2 mutants and expressed in regions of Foxa2 activity

TF	TF Class	TF Supergroup	IC	Back-ground gene hits	Back-ground gene non-hits	Target gene hits	Target gene non-hits	Background TFBS hits	Background TFBS rate	Target TFBS hits	Target TFBS rate	Z-score	Fisher score
CREB1	bZIP	vertebrate	12.605	6268	8882	10	9	11653	0.0045	23	0.0095	12.74	2.22E-01
Pax5	PAIRED	vertebrate	12.432	2057	13093	5	14	2568	0.0017	6	0.0042	10.36	1.05E-01
TAL1-TCF3	bHLH	vertebrate	14.07	6833	8317	11	8	14163	0.0055	23	0.0095	9.323	1.87E-01
MYC-MAX	bHLH-ZIP	vertebrate	14.237	3723	11427	4	15	5317	0.0019	11	0.0042	8.941	7.23E-01
Cebpa	bZIP	vertebrate	9.187	9684	5466	11	8	32098	0.0124	44	0.0183	8.94	7.86E-01
ZEB1	ZN-FINGER, C2H2	vertebrate	8.305	14121	1029	18	1	275256	0.0533	312	0.0648	8.685	6.27E-01
Roaz	ZN-FINGER, C2H2	vertebrate	17.925	5258	9892	10	9	10026	0.0049	16	0.0083	8.404	8.33E-02
Myb	TRP-CLUSTER	vertebrate	9.883	11245	3905	14	5	50473	0.013	65	0.018	7.419	6.38E-01
ELF5	ETS	vertebrate	8.693	13088	2062	16	3	99640	0.0289	115	0.0358	6.962	7.46E-01
RORA_1	NUCLEAR RECEPTOR	vertebrate	13.19	8489	6661	9	10	20608	0.0066	28	0.0097	6.323	8.39E-01
Evi1	ZN-FINGER, C2H2	vertebrate	17.909	1572	13578	3	16	1918	0.0009	4	0.0019	6.091	3.15E-01
Gfi	ZN-FINGER, C2H2	vertebrate	9.47	11160	3990	14	5	52619	0.017	62	0.0214	5.869	6.17E-01
NFYA	CAAT-BOX	vertebrate	12.925	5722	9428	10	9	10334	0.0053	14	0.0077	5.599	1.37E-01
RORA_2	NUCLEAR RECEPTOR	vertebrate	17.425	3194	11956	6	13	4303	0.0019	7	0.0034	5.52	1.96E-01
Nkx2-5	HOMEO	vertebrate	8.27	13172	1978	17	2	162945	0.0368	176	0.0426	5.249	5.40E-01
MAX	bHLH-ZIP	vertebrate	12.685	7442	7708	7	12	16197	0.0052	21	0.0073	4.771	9.04E-01
Arnt	bHLH	vertebrate	10.992	9976	5174	9	10	34946	0.0068	43	0.0089	4.449	9.71E-01
HLF	bZIP	vertebrate	11.147	6319	8831	9	10	12469	0.0048	16	0.0066	4.412	3.90E-01
HNF1A	HOMEO	vertebrate	15.548	3161	11989	5	14	4806	0.0022	7	0.0034	4.394	3.63E-01
RELA	REL	vertebrate	14.757	5855	9295	7	12	10424	0.0034	14	0.0048	4.299	6.48E-01
Mycn	bHLH-ZIP	vertebrate	10.443	9857	5293	9	10	33506	0.0065	41	0.0085	4.254	9.66E-01
Fos	bZIP	vertebrate	10.67	10961	4189	9	10	43473	0.0112	50	0.0138	4.201	9.95E-01
NR3C1	NUCLEAR RECEPTOR	vertebrate	14.749	3174	11976	6	13	4323	0.0025	6	0.0037	4.108	1.92E-01
FOXI1	FORKHEAD	vertebrate	13.183	9787	5363	11	8	39278	0.0152	43	0.0178	3.655	8.04E-01
MZF1_1-4	ZN-FINGER, C2H2	vertebrate	8.586	13733	1417	17	2	219114	0.0424	225	0.0467	3.611	7.40E-01
Staf	ZN-FINGER, C2H2	vertebrate	17.541	1677	13473	2	17	2024	0.0013	3	0.0021	3.544	6.38E-01
Spz1	bHLH-ZIP	vertebrate	11.907	6562	8588	9	10	13159	0.0047	16	0.0061	3.498	4.47E-01
ELK1	ETS	vertebrate	8.812	11747	3403	14	5	55833	0.018	60	0.0208	3.49	7.59E-01
Foxd3	FORKHEAD	vertebrate	12.945	9870	5280	11	8	43921	0.017	47	0.0195	3.28	8.18E-01
USF1	bHLH-ZIP	vertebrate	11.29	9605	5545	8	11	30783	0.0069	35	0.0085	3.086	9.83E-01

oPOSSUM output: TF motifs identified in promoters of genes reduced in Foxa2 mutants and expressed in regions of Foxa2 activity

TF	TF Class	TF Supergroup	IC	Back-ground gene hits	Back-ground gene non-hits	Target gene hits	Target gene non-hits	Background TFBS hits	Background TFBS rate	Target TFBS hits	Target TFBS rate	Z-score	Fisher score
Lhx3	HOMEO	vertebrate	12.941	9103	6047	10	9	40461	0.0117	44	0.0137	3.056	8.16E-01
MEF2A	MADS	vertebrate	15.709	6423	8727	11	8	14535	0.0047	17	0.0059	2.926	1.29E-01
NFKB1	REL	vertebrate	15.627	3459	11691	5	14	5384	0.0019	7	0.0027	2.866	4.45E-01
**T**	**T-BOX**	**vertebrate**	**17.86**	**1656**	**13494**	**3**	**16**	**1952**	**0.0007**	**3**	**0.001**	**2.79**	**3.45E-01**
Hand1-Tcfe2a	bHLH	vertebrate	10.144	11841	3309	14	5	62563	0.0202	64	0.0221	2.349	7.80E-01
ZNF354C	ZN-FINGER, C2H2	vertebrate	8.958	13763	1387	18	1	207194	0.0401	205	0.0425	2.11	4.70E-01
Myf	bHLH	vertebrate	15.914	8752	6398	9	10	26914	0.0104	28	0.0116	1.991	8.74E-01
HNF4A	NUCLEAR RECEPTOR	vertebrate	9.617	6718	8432	9	10	12991	0.0054	14	0.0063	1.921	4.83E-01
NR2F1	NUCLEAR RECEPTOR	vertebrate	15.924	4261	10889	6	13	6220	0.0028	7	0.0034	1.812	4.54E-01
E2F1	E2F_TDP	vertebrate	13.838	4313	10837	5	14	6897	0.0018	8	0.0022	1.682	6.67E-01
NFIL3	bZIP	vertebrate	14.139	4927	10223	8	11	9156	0.0032	10	0.0038	1.612	2.54E-01
**Foxa2**	**FORKHEAD**	**vertebrate**	**12.43**	**9813**	**5337**	**9**	**10**	**39021**	**0.0151**	**39**	**0.016**	**1.49**	**9.64E-01**
Nobox	HOMEO	vertebrate	9.573	11657	3493	14	5	77135	0.0199	76	0.021	1.353	7.39E-01
Pdx1	HOMEO	vertebrate	9.04	12957	2193	18	1	147626	0.0286	144	0.0299	1.329	2.16E-01
MZF1_5-13	ZN-FINGER, C2H2	vertebrate	9.4	11019	4131	14	5	56288	0.0182	55	0.019	1.087	5.80E-01
SRY	HMG	vertebrate	9.193	12056	3094	16	3	91962	0.0267	89	0.0277	1.051	4.37E-01
Prrx2	HOMEO	vertebrate	9.063	12658	2492	18	1	140682	0.0227	136	0.0235	0.934	1.56E-01
Lhx3	HOMEO	vertebrate	16.354	5402	9748	6	13	12354	0.0052	12	0.0054	0.472	7.24E-01
SOX9	HMG	vertebrate	9.079	10175	4975	10	9	38671	0.0112	37	0.0115	0.446	9.41E-01
REL	REL	vertebrate	10.515	9080	6070	9	10	24842	0.008	23	0.008	-0.14	9.11E-01
YY1	ZN-FINGER, C2H2	vertebrate	8.101	12665	2485	18	1	91677	0.0177	85	0.0176	-0.15	1.57E-01
NHLH1	bHLH	vertebrate	14.132	5659	9491	5	14	10843	0.0042	10	0.0042	-0.166	8.94E-01
Bapx1	HOMEO	vertebrate	8.542	12243	2907	15	4	70226	0.0204	65	0.0202	-0.2	7.06E-01
NF-kappaB	REL	vertebrate	13.345	7066	8084	7	12	15229	0.0049	14	0.0048	-0.21	8.62E-01
ELK4	ETS	vertebrate	14.123	5419	9731	6	13	8856	0.0026	8	0.0025	-0.327	7.27E-01
SP1	ZN-FINGER, C2H2	vertebrate	9.719	10178	4972	14	5	47203	0.0152	43	0.0149	-0.51	3.70E-01
STAT1	Stat	vertebrate	18.431	2699	12451	3	16	3491	0.0016	3	0.0015	-0.603	6.84E-01
Foxq1	FORKHEAD	vertebrate	14.07	6787	8363	8	11	16848	0.006	15	0.0057	-0.633	6.77E-01
SPIB	ETS	vertebrate	9.06	13830	1320	17	2	180763	0.0408	165	0.04	-0.752	7.73E-01
Sox17	HMG	vertebrate	10.502	12180	2970	16	3	72292	0.021	65	0.0202	-0.908	4.73E-01
Arnt-Ahr	bHLH	vertebrate	9.532	12002	3148	10	9	79481	0.0154	70	0.0145	-1.202	9.98E-01
IRF1	TRP-CLUSTER	vertebrate	16.008	5665	9485	6	13	10266	0.004	8	0.0033	-1.81	7.73E-01
FOXD1	FORKHEAD	vertebrate	11.926	9212	5938	10	9	28801	0.0074	23	0.0064	-2.146	8.33E-01

oPOSSUM output: TF motifs identified in promoters of genes reduced in Foxa2 mutants and expressed in regions of Foxa2 activity

TF	TF Class	TF Supergroup	IC	Back-ground gene hits	Back-ground gene non-hits	Target gene hits	Target gene non-hits	Background TFBS hits	Background TFBS rate	Target TFBS hits	Target TFBS rate	Z-score	Fisher score
PBX1	HOMEO	vertebrate	14.641	3260	11890	3	16	4843	0.0019	3	0.0012	-2.541	8.09E-01
Sox5	HMG	vertebrate	10.831	12340	2810	15	4	98228	0.0222	82	0.0199	-2.7	7.31E-01
GABPA	ETS	vertebrate	13.89	6483	8667	5	14	12454	0.004	7	0.0024	-4.335	9.57E-01
NKX3-1	HOMEO	vertebrate	11.127	9267	5883	8	11	34707	0.0078	22	0.0053	-4.871	9.72E-01
TEAD1	TEA	vertebrate	15.678	4761	10389	3	16	7436	0.0029	3	0.0012	-5.237	9.65E-01
Ddit3-Cebpa	bZIP	vertebrate	11.652	5480	9670	3	16	9815	0.0038	4	0.0017	-5.958	9.87E-01
FOXF2	FORKHEAD	vertebrate	14.824	4066	11084	2	17	6528	0.0029	2	0.001	-6.261	9.79E-01
