# Supplementary material for: Microarray analysis of Foxa2 mutant mouse embryos reveals novel gene expression and inductive roles for the gastrula organizer and its derivatives
Source: BMC Genomics. 2008 Oct 30;9:511. doi: 10.1186/1471-2164-9-511 (PMC2605479; doi:10.1186/1471-2164-9-511)
Supplement: Additional file 10 — Supplementary Table 7. oPOSSUM output: putative target genes with conserved Foxa2 binding motifs. [file 1471-2164-9-511-S10.pdf]

oPOSSUM output: putative target genes with conserved Foxa2 binding motifs

Gene ID	Ensembl ID	Chr	Strand	TSS	Promoter Start	Promoter End	TFBS Sequence	TFBS Start	TFBS Rel. Start	TFBS End	TFBS Rel. End	TFBS Orien-tation	TFBS Score
Nptx2	ENSMUSG00000059991	5	1	144799523	144789523	144804522	ACTTATTGGTTT	144788909	-10614	144788921	-10602	1	9.12E-01
				144799523	144789523	144804522	GCAAATTTATTT	144788978	-10545	144788990	-10533	1	8.27E-01
				144799523	144789523	144804522	CTTCATTTGTTT	144795312	-4211	144795324	-4199	1	8.48E-01
				144799523	144789523	144804522	AAATAATTGCTC	144797422	-2101	144797434	-2089	-1	8.59E-01
				144799523	144789523	144804522	AACTATTTACAT	144798159	-1364	144798171	-1352	1	8.91E-01
				144799523	144789523	144804522	TTTCATTTATTT	144798260	-1263	144798272	-1251	1	8.01E-01
				144799523	144789523	144804522	TACAATTTGTTT	144798275	-1248	144798287	-1236	1	8.08E-01
				144799523	144789523	144804522	ATCCATTTGTTT	144798323	-1200	144798335	-1188	1	8.28E-01
Foxa2	ENSMUSG00000037025	2	-1	147738410	147734364	147748410	AAATATTGACTT	147737055	1356	147737067	1344	-1	9.75E-01
				147737339	147734364	147747339	AAATATTGACTT	147737055	285	147737067	273	-1	9.75E-01
				147738410	147734364	147748410	TAATATTAACTT	147737074	1337	147737086	1325	-1	8.58E-01
				147737339	147734364	147747339	TAATATTAACTT	147737074	266	147737086	254	-1	8.58E-01
				147738410	147734364	147748410	ATTTATTTATCT	147738189	222	147738201	210	1	8.51E-01
				147737339	147734364	147747339	ATTTATTTATCT	147738189	-850	147738201	-862	1	8.51E-01
				147738410	147734364	147748410	CCCTGTTTGTTT	147738575	-165	147738587	-177	-1	9.02E-01
				147737339	147734364	147747339	CCCTGTTTGTTT	147738575	-1236	147738587	-1248	-1	9.02E-01
				147738410	147734364	147748410	CATTGTTAGCTC	147744935	-6525	147744947	-6537	1	8.42E-01
				147737339	147734364	147747339	CATTGTTAGCTC	147744935	-7596	147744947	-7608	1	8.42E-01
				147738410	147734364	147748410	AAATATTAATTA	147747220	-8810	147747232	-8822	-1	8.07E-01
				147737339	147734364	147747339	AAATATTAATTA	147747220	-9881	147747232	-9893	-1	8.07E-01
				147738410	147734364	147748410	CCATATTTAATT	147747247	-8837	147747259	-8849	-1	8.76E-01
				147737339	147734364	147747339	CCATATTTAATT	147747247	-9908	147747259	-9920	-1	8.76E-01
Foxa1	ENSMUSG00000035451	12	-1	58464133	58459134	58474060	ATTTATTTGGTT	58465731	-1598	58465743	-1610	-1	8.41E-01
				58464133	58459134	58474060	ATTTATTTATTT	58470393	-6260	58470405	-6272	-1	9.25E-01
				58464133	58459134	58474060	CTTTATTTATTG	58471106	-6973	58471118	-6985	-1	8.37E-01
				58464133	58459134	58474060	GACTATTTGATT	58472695	-8562	58472707	-8574	1	8.57E-01
				58464133	58459134	58474060	AAATATTACCTT	58472814	-8681	58472826	-8693	1	8.20E-01
Pim1	ENSMUSG00000024014	17	1	29217824	29207896	29222496	AAATGCTTCCTT	29216352	-1472	29216364	-1460	-1	8.13E-01
				29220099	29210099	29222496	AAATGCTTCCTT	29216352	-3747	29216364	-3735	-1	8.13E-01
Cer1	ENSMUSG00000038192	4	-1	82356382	82353295	82365088	ATGTGTTTGCTT	82356459	-77	82356471	-89	-1	8.65E-01
				82356382	82353295	82365088	AAGTGTTTGCCC	82356472	-90	82356484	-102	1	8.14E-01
				82356382	82353295	82365088	CATTCCTTATTT	82362095	-5713	82362107	-5725	1	8.12E-01
				82356382	82353295	82365088	TAATATTTACGT	82362821	-6439	82362833	-6451	-1	8.75E-01
Sox17	ENSMUSG00000025902	1	-1	4483685	4481009	4493685	CATTTTTTACTC	4490405	-6720	4490417	-6732	1	8.66E-01
				4486494	4481495	4496494	CATTTTTTACTC	4490405	-3911	4490417	-3923	1	8.66E-01
				4483685	4481009	4493685	CATTGTGTGTTT	4490371	-6686	4490383	-6698	1	8.54E-01
				4486494	4481495	4496494	CATTGTGTGTTT	4490371	-3877	4490383	-3889	1	8.54E-01
				4483685	4481009	4493685	AATTGTTTGTTG	4489925	-6240	4489937	-6252	-1	8.59E-01
				4486494	4481495	4496494	AATTGTTTGTTG	4489925	-3431	4489937	-3443	-1	8.59E-01

oPOSSUM output: putative target genes with conserved Foxa2 binding motifs

Gene ID	Ensembl ID	Chr	Strand	TSS	Promoter Start	Promoter End	TFBS Sequence	TFBS Start	TFBS Rel. Start	TFBS End	TFBS Rel. End	TFBS Orien-tation	TFBS Score
				4483685	4481009	4493685	AGACATTTACTT	4488874	-5189	4488886	-5201	-1	8.51E-01
				4486494	4481495	4496494	AGACATTTACTT	4488874	-2380	4488886	-2392	-1	8.51E-01
T	ENSMUSG00000062327	17	1	8272597	8262597	8277596	CATTGTTGGCCC	8272283	-314	8272295	-302	1	8.39E-01
				8271820	8262597	8276819	CATTGTTGGCCC	8272283	464	8272295	476	1	8.39E-01
				8272597	8262597	8277596	AAATGTTTGCAC	8272206	-391	8272218	-379	-1	8.69E-01
				8271820	8262597	8276819	AAATGTTTGCAC	8272206	387	8272218	399	-1	8.69E-01
Mlf1	ENSMUSG00000048416	3	1	67462026	67452026	67467025	GTTTGTTTTCTT	67463253	1228	67463265	1240	1	8.05E-01
				67469882	67462615	67472867	GTTTGTTTTCTT	67463253	-6629	67463265	-6617	1	8.05E-01
				67462026	67452026	67467025	CAATCTTTATAT	67464243	2218	67464255	2230	-1	8.31E-01
				67469882	67462615	67472867	CAATCTTTATAT	67464243	-5639	67464255	-5627	-1	8.31E-01
				67462026	67452026	67467025	CAATATTTACTG	67464321	2296	67464333	2308	-1	9.06E-01
				67469882	67462615	67472867	CAATATTTACTG	67464321	-5561	67464333	-5549	-1	9.06E-01
Smoc1	ENSMUSG00000021136	12	1	81945676	81936117	81950675	GAGTATTTTCTT	81943183	-2493	81943195	-2481	-1	8.35E-01
				81945676	81936117	81950675	CTATGTCGACTT	81943262	-2414	81943274	-2402	1	8.31E-01
				81945676	81936117	81950675	AATTATCTGCTG	81946491	816	81946503	828	-1	8.04E-01
				81945676	81936117	81950675	AAGTGTTTGTCC	81947225	1550	81947237	1562	1	8.10E-01
				81945676	81936117	81950675	GTTTATTAATTT	81947739	2064	81947751	2076	1	8.14E-01

Note: genome positions above are based on UCSC Mouse Feb. 2006 mm8, NCBI Build 36--converted to mm9 for Additional File 9
