# Supplementary material for: Microarray analysis of Foxa2 mutant mouse embryos reveals novel gene expression and inductive roles for the gastrula organizer and its derivatives
Source: BMC Genomics. 2008 Oct 30;9:511. doi: 10.1186/1471-2164-9-511 (PMC2605479; doi:10.1186/1471-2164-9-511)
Supplement: Additional file 11 — Supplementary Table 8. oPOSSUM output: putative target genes with conserved Brachyury/T binding motifs. [file 1471-2164-9-511-S11.pdf]

oPOSSUM output: putative target genes with conserved Brachyury/T binding motifs

Gene ID	Ensembl ID	Chr	Strand	TSS	Promoter Start	Promoter End	TFBS Sequence	TFBS Start	TFBS Rel. Start	TFBS End	TFBS Rel. End	TFBS Orien-tation	TFBS Score
Foxa2	ENSMUSG00000037025	2	-1	147738410	147734364	147748410	CTAAGTGTGAG	147737874	537	147737885	526	-1	8.13E-01
				147737339	147734364	147747339	CTAAGTGTGAG	147737874	-535	147737885	-546	-1	8.13E-01
Foxa1	ENSMUSG00000035451	12	-1	58464133	58459134	58474060	GTAGGTGCGAG	58464117	17	58464128	6	-1	8.05E-01
Cer1	ENSMUSG00000038192	4	-1	82356382	82353295	82365088	CCAGTTGTGAA	82357005	-623	82357016	-634	-1	8.22E-01

Note: genome positions above are based on UCSC Mouse Feb. 2006 mm8, NCBI Build 36--converted to mm9 for Additional File 9

oPOSSUM output: putative target genes with conserved Brachyury/T binding motifs
