# Supplementary material for: Microarray analysis of Foxa2 mutant mouse embryos reveals novel gene expression and inductive roles for the gastrula organizer and its derivatives
Source: BMC Genomics. 2008 Oct 30;9:511. doi: 10.1186/1471-2164-9-511 (PMC2605479; doi:10.1186/1471-2164-9-511)
Supplement: Additional file 12 — Supplementary Table 9. Summary of conserved Foxa2 and T binding motif predictions around putative Foxa2 target genes. [file 1471-2164-9-511-S12.pdf]

Summary of conserved Foxa2 and T binding motif predictions around putative Foxa2 target genes.

Gene Symbol	Expression at E7.5	SynoR (Foxa2 and T within 200bp)	oPOSSUM (Foxa2 conserved)	oPOSSUM (T conserved)
Cer1	DE	chr4:82537493-82537542	chr4:82531132-82531144<br>chr4:82531145-82531157<br>chr4:82536768-82536780<br>chr4:82537494-82537506	chr2:147872169-147872180
Foxa1	AME DE		chr12:58648706-58648718<br>chr12:58653368-58653380<br>chr12:58654081-58654093<br>chr12:58655670-58655682<br>chr12:58655789-58655801	chr4:82531678-82531689
Foxa2	ND AME DE	chr2:147757619-147757717	chr2:147871350-147871362<br>chr2:147871369-147871381<br>chr2:147872484-147872496<br>chr2:147872870-147872882<br>chr2:147879230-147879242<br>chr2:147881515-147881527<br>chr2:147881542-147881554	chr12:96938218-96938229
Mlf1	ND		chr3:67179246-67179258<br>chr3:67180236-67180248<br>chr3:67180314-67180326	
Pim1	ND PS		chr17:29626285-29626297	
Smoc1	ND LPM		chr12:82125322-82125334<br>chr12:82125401-82125413<br>chr12:82128630-82128642<br>chr12:82129364-82129376<br>chr12:82129878-82129890	
Sox17	DE		chr1:4488874-4488886<br>chr1:4489925-4489937<br>chr1:4490371-4490383<br>chr1:4490405-4490417	
T	ND AME PS		chr17:8626897-8626909<br>chr17:8626974-8626986	

Note: Genome positions according to USCS Mouse July 2007 (mm9, NCBI Build 37). SynoR (http://synor.dcode.org/) and oPOSSUM (http://www.cisreg.ca/oPOSSUM/) use different position weight matrices to predict Foxa2 and T binding sites (TRANSFAC and JASPAR, respectively). Abbreviations: ND, node; DE, definitive endoderm; AME, anterior mesendoderm; LPM, lateral plate mesoderm; PS, primitive streak.
