# Supplementary material for: Microarray analysis of Foxa2 mutant mouse embryos reveals novel gene expression and inductive roles for the gastrula organizer and its derivatives
Source: BMC Genomics. 2008 Oct 30;9:511. doi: 10.1186/1471-2164-9-511 (PMC2605479; doi:10.1186/1471-2164-9-511)
Supplement: Additional file 13 — Starting material for Foxa2 expression profiling. Details of the numbers and stages of embryos collected for the screen. [file 1471-2164-9-511-S13.pdf]

Starting Material for Foxa2 Expression Profiling

(wild-type 4n B5/EGFP <-> ES cell lines)

stage	wild type (GeneChip 1)	Foxa2 (GeneChip 1)	Foxa2 (GeneChip 2)
Headfold (E7.75)	17	17	16
Neural Plate (E7.6)	7	7	5
Late Streak (E7.5)	19	19	19
Mid-Streak (E7.25)	10	10	8
total	53	53	48
**average age**	7.55	7.55	7.55

stage	wild type (GeneChip 2)
Headfold (E7.75)	7
Neural Plate (E7.6)	7
Late Streak (E7.5)	22
Mid-Streak (E7.25)	14
total	50
**average age**	7.48

Note: Stage-matched ICR wild-type embryos were used. Staging according to Downs and Davies, 1993.
